# Supplementary material for: PIK Your Poison: The Effects of Combining PI3K and CDK Inhibitors against Metastatic Cutaneous Squamous Cell Carcinoma In Vitro
Source: Cancers (Basel). 2024 Jan 15;16(2):370. doi: 10.3390/cancers16020370 (PMC10814950; doi:10.3390/cancers16020370)
Supplement: Supplementary file 1 [file cancers-16-00370-s001.zip › Supplementary Figures.pdf]

Supplementary Figures

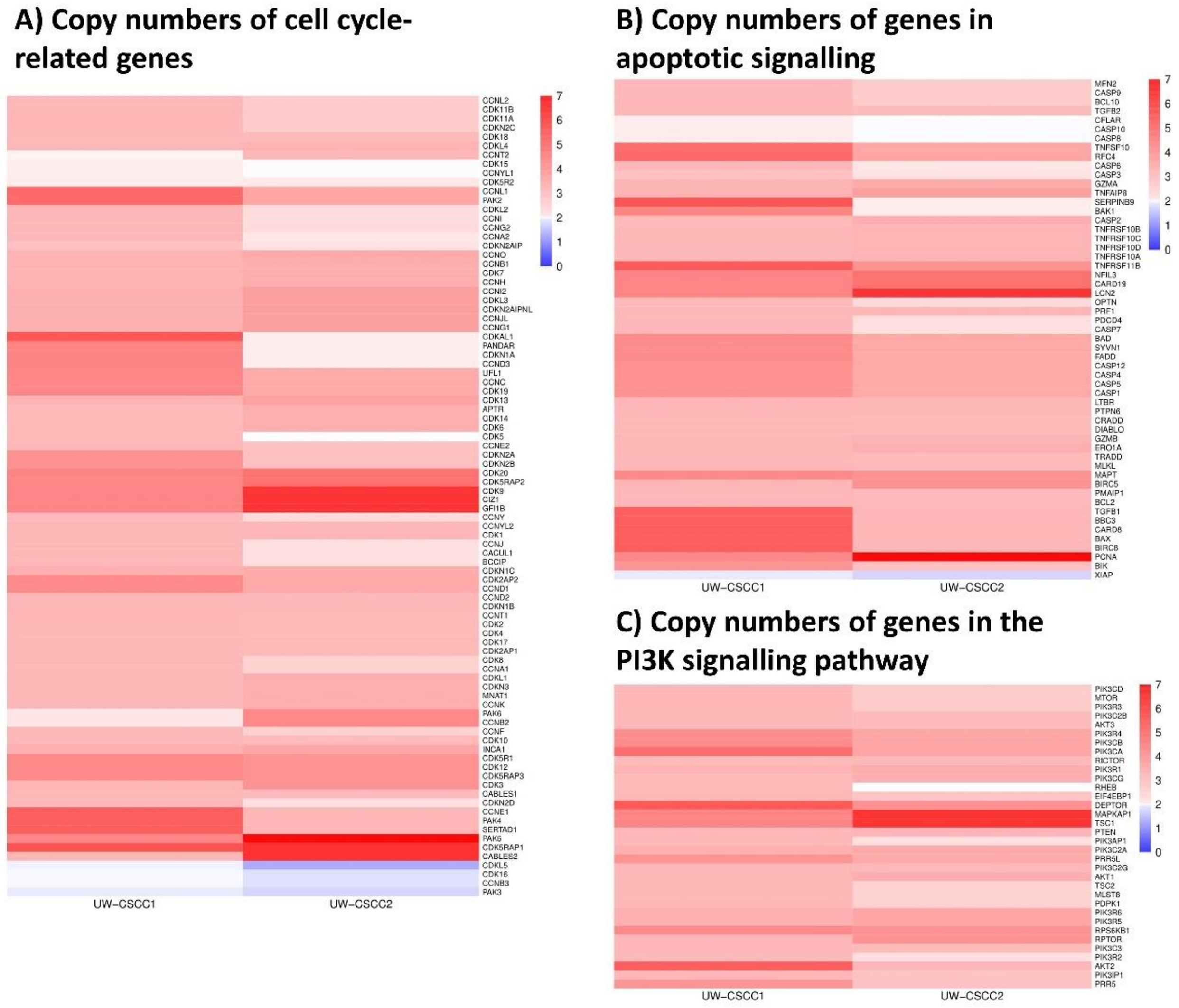

Figure S1. Heatmap of copy number variation (CNV) between UW-CSCC1 and UW-CSCC2 for genes relating to a) cell cycle signalling, b) apoptotic signalling, and c) PI3K/AKT/mTOR signalling. CNV < 2 were considered minor changes, with most genes analysed presenting CNV between 2-7. Data used to generate these figures can be found in Supplementary Data File 2.

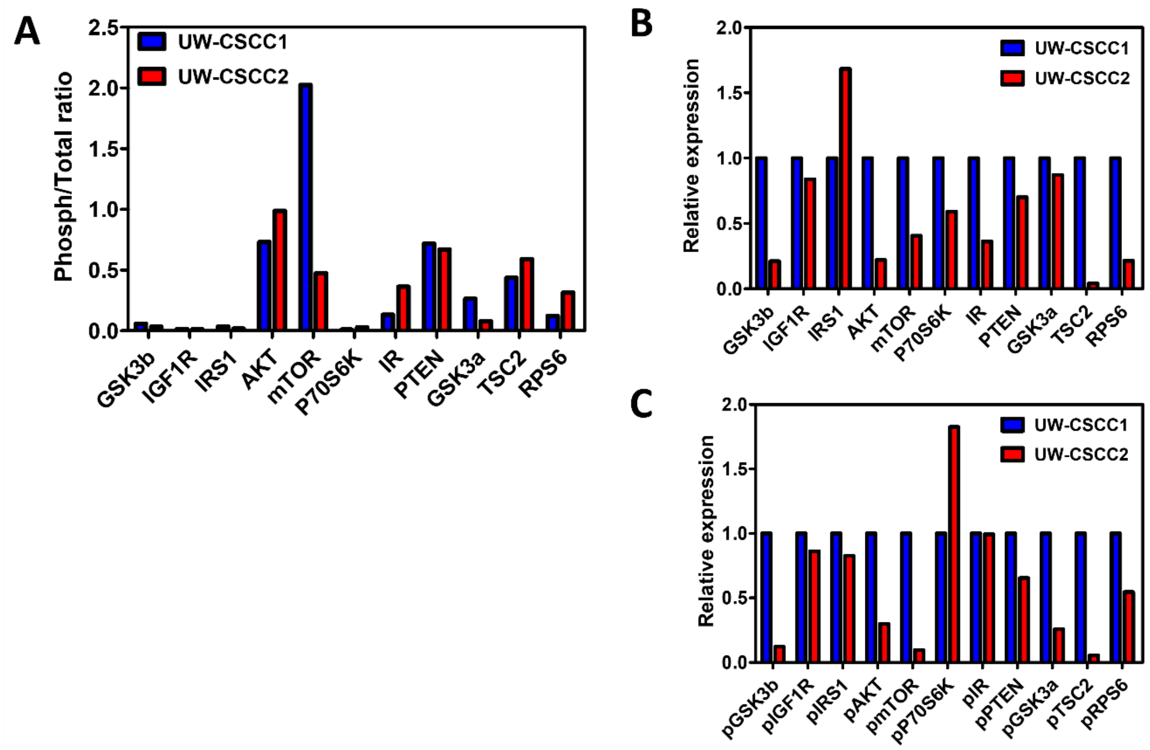

Figure S2. MAGPIX multiplex bead-based analysis of PI3K/AKT/mTOR regulators in UW-CSCC1 (blue) and UW-CSCC2 (red). A) Phosphorylated protein expression as a ratio of total expression. B) Relative expression of total protein. C) Relative expression of phosphorylated protein.

MAGPIX multiplex methodology:

A Bio-Plex MAGPIX system with Bio-Plex Pro-Wash Station (Bio-rad, USA) was used for multiplex analysis of UW-CSCC cell lines. Cell lysates of UW-CSCC1 and UW-CSCC2 were prepared using a lysis buffer (50 mM Tris HCL [pH 7.4], 150 mM NaCl, 1.0 % Triton-X100, 5 mM EDTA, 1 mM PMSF, 1 mM Na_3VO_4). Lysates were analysed using the MILLIPLEX Akt/mTOR Phosphoprotein Magnetic Bead (11-plex) kit (Millipore, USA) according to manufacturer's instructions. Data was generated using Bio-Plex Manager MP, and interpreted using GraphPad Prism V5 (GraphPad Software, USA).

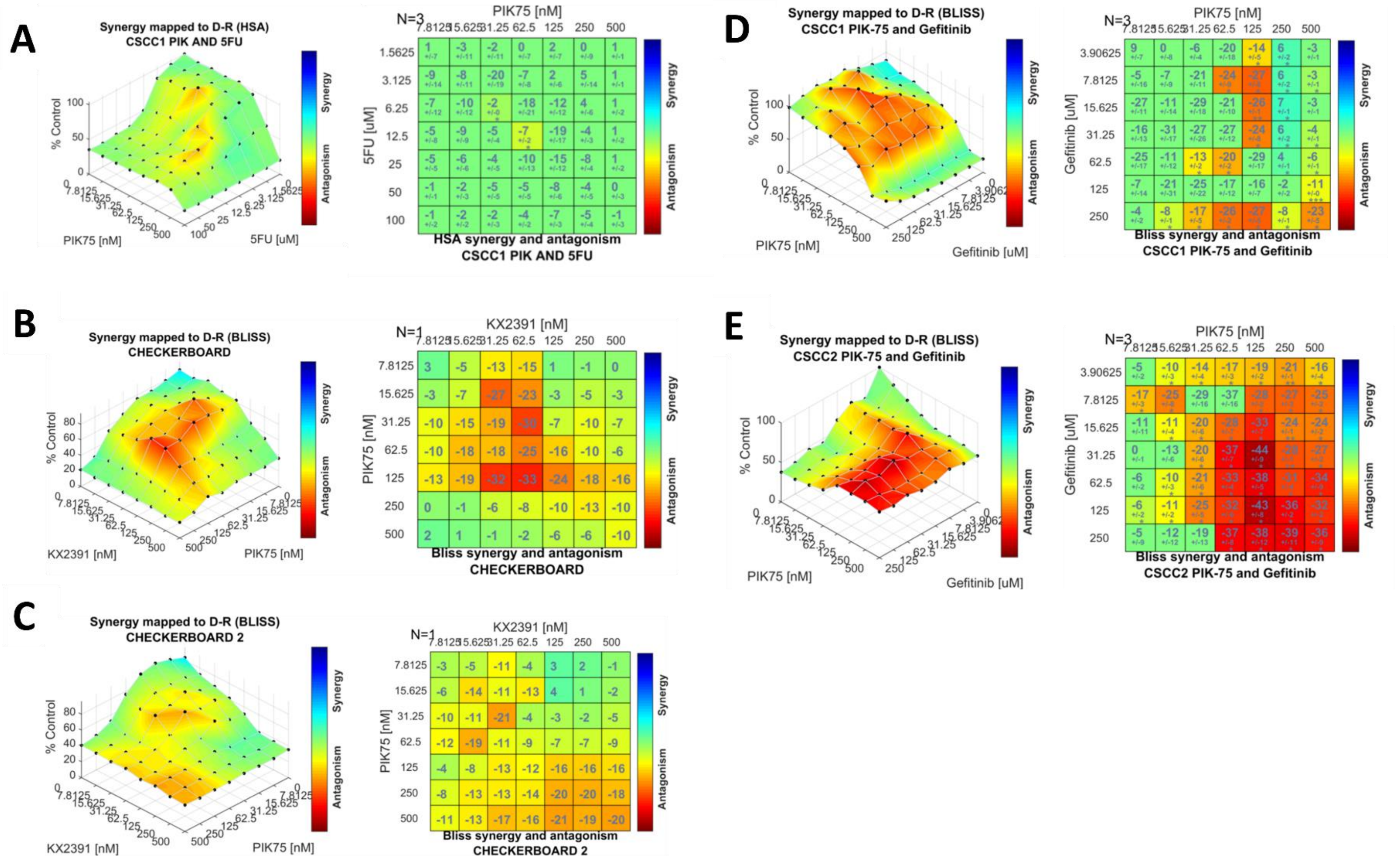

Figure S3. Synergistic score matrix after 72 hours of combination PIK-75 (selective PI3Ki) and other chemotherapeutics on cSCC cell lines, implementing a BLISS analysis model. Raw data from the checkerboard assay were normalised as a percentage of the no drug control and used as input with the drug combination analysis software Combenefit (https://academic.oup.com/bioinformatics/article/32/18/2866/1743073). Images are representative of triplicate observations. A) PIK-75 in combination with 5-fluorouracil (5-FU) [UW-CSCC1]. B-C) PIK-75 in combination with SRC inhibitor KX2391 [UW-CSCC1 and UW-CSCC2, respectively]. D-E) PIK-75 in combination with EGFR inhibitor Gefitinib [UW-CSCC1 and UW-CSCC2, respectively].

(A)

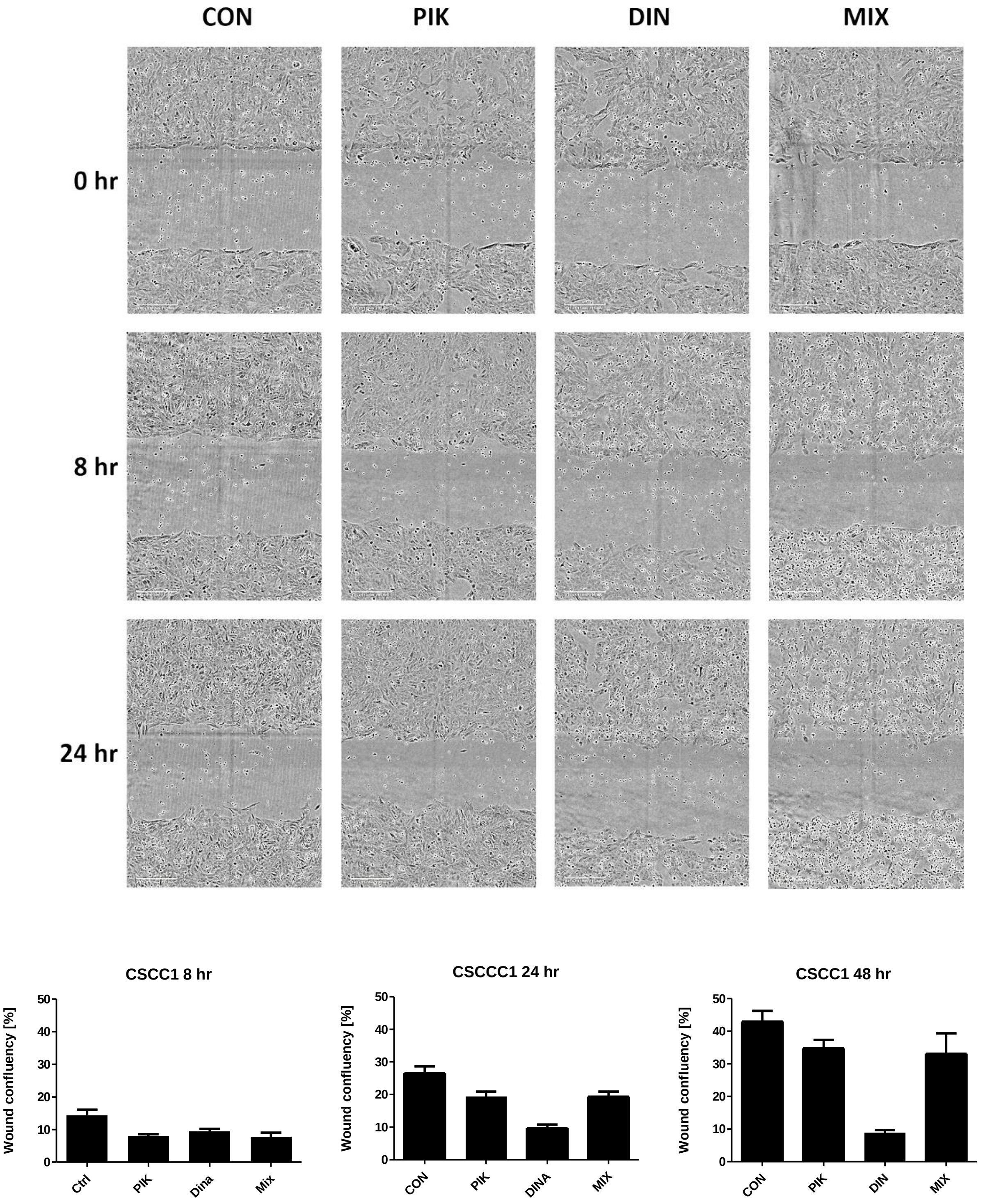
CON
PIK
DIN
MIX
0 hr
8 hr
24 hr
CSCC1 8 hr
Wound confluency [%]
50
40
30
20
10
0
Ctrl
PIK
Dina
Mix
CSCCC1 24 hr
Wound confluency [%]
CON
PIK
DINA
MIX
CSCC1 48 hr
Wound confluency [%]
CON
PIK
DIN
MIX

(B)

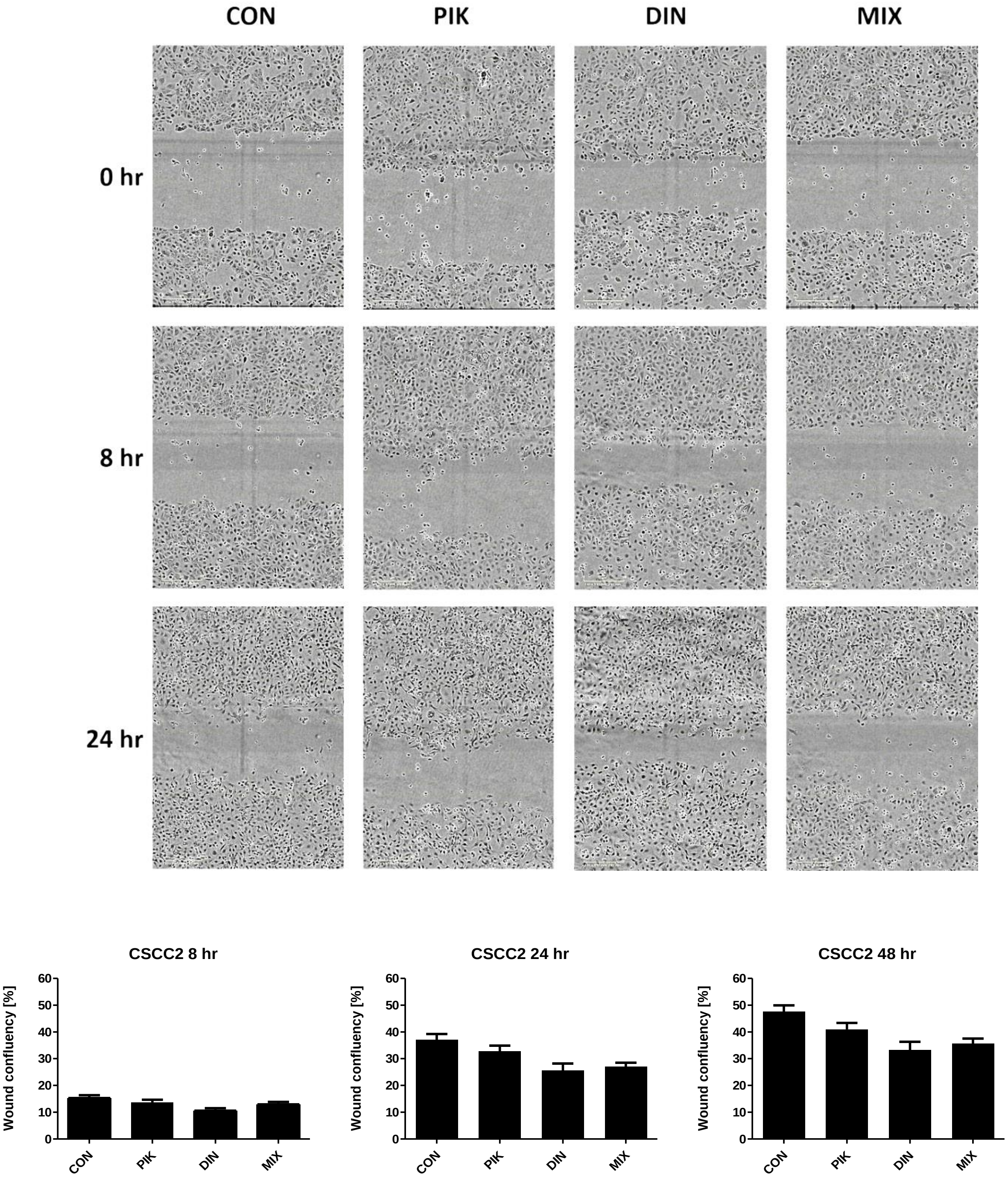

Figure S4. Representative images of wound confluency over 24 hours for UW-CSCC1 (A) and UW-CSCC2 (B) in response to drug treatment. CON, Vehicle control (DMSO); PIK, PIK-75 (both cell lines 62.5 nM), DIN, dinaciclib (UW-CSCC1: 16 nM; UW-CSCC2: 8 nM); MIX, mixture. Standard error of the mean shown, *n* = 10

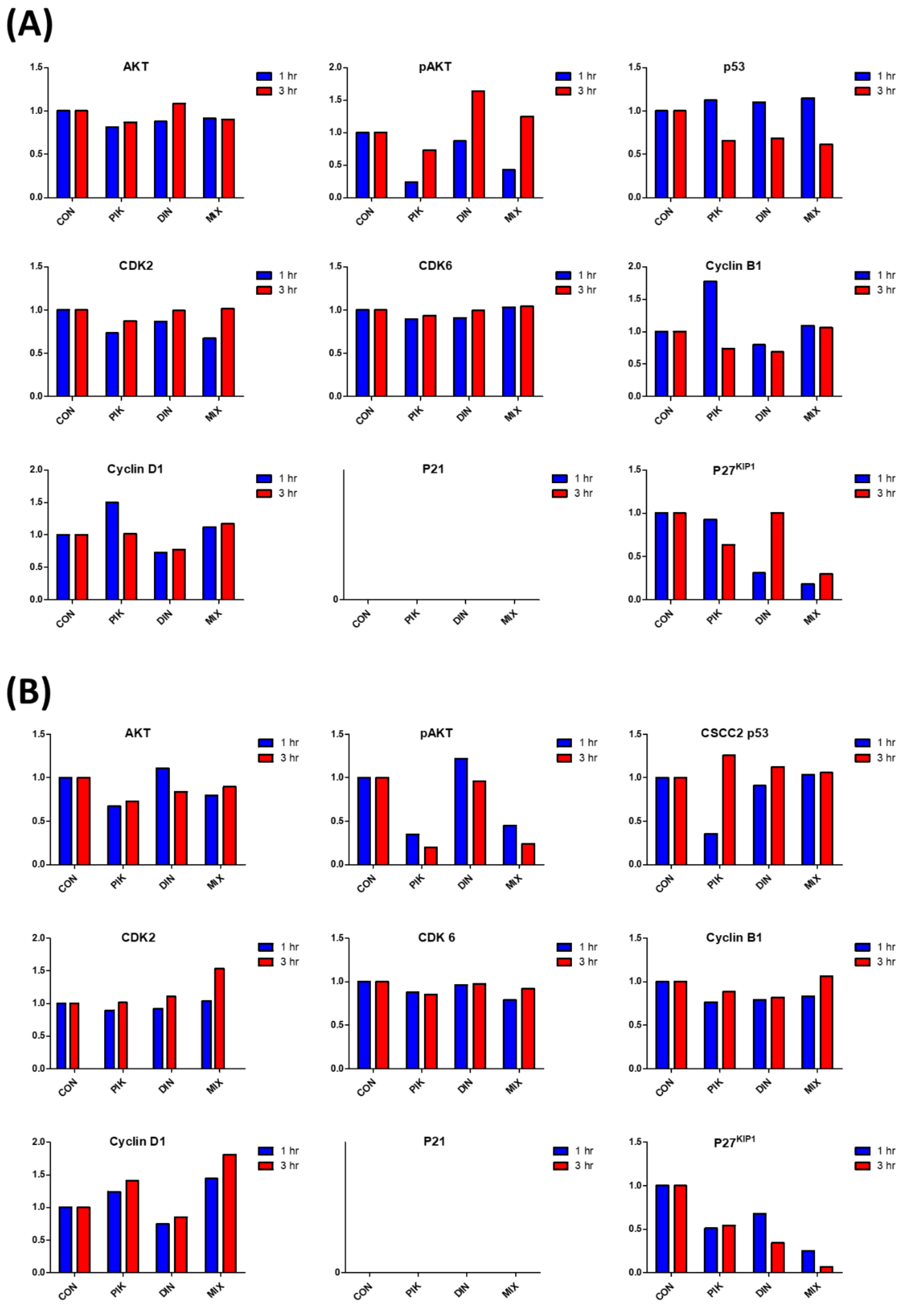

Figure S5. Densitometry analysis of Western blots (Figure 6, results) from control and treated cells at 1 hour (blue) and 3 hours (red) for UW-CSCC1 (A) and UW-CSCC2 (B). Densitometry was performed using the ImageJ analyse lane function, with all samples normalised to their respective GAPDH loading control. P21 produced no observable bands, hence the empty plot.

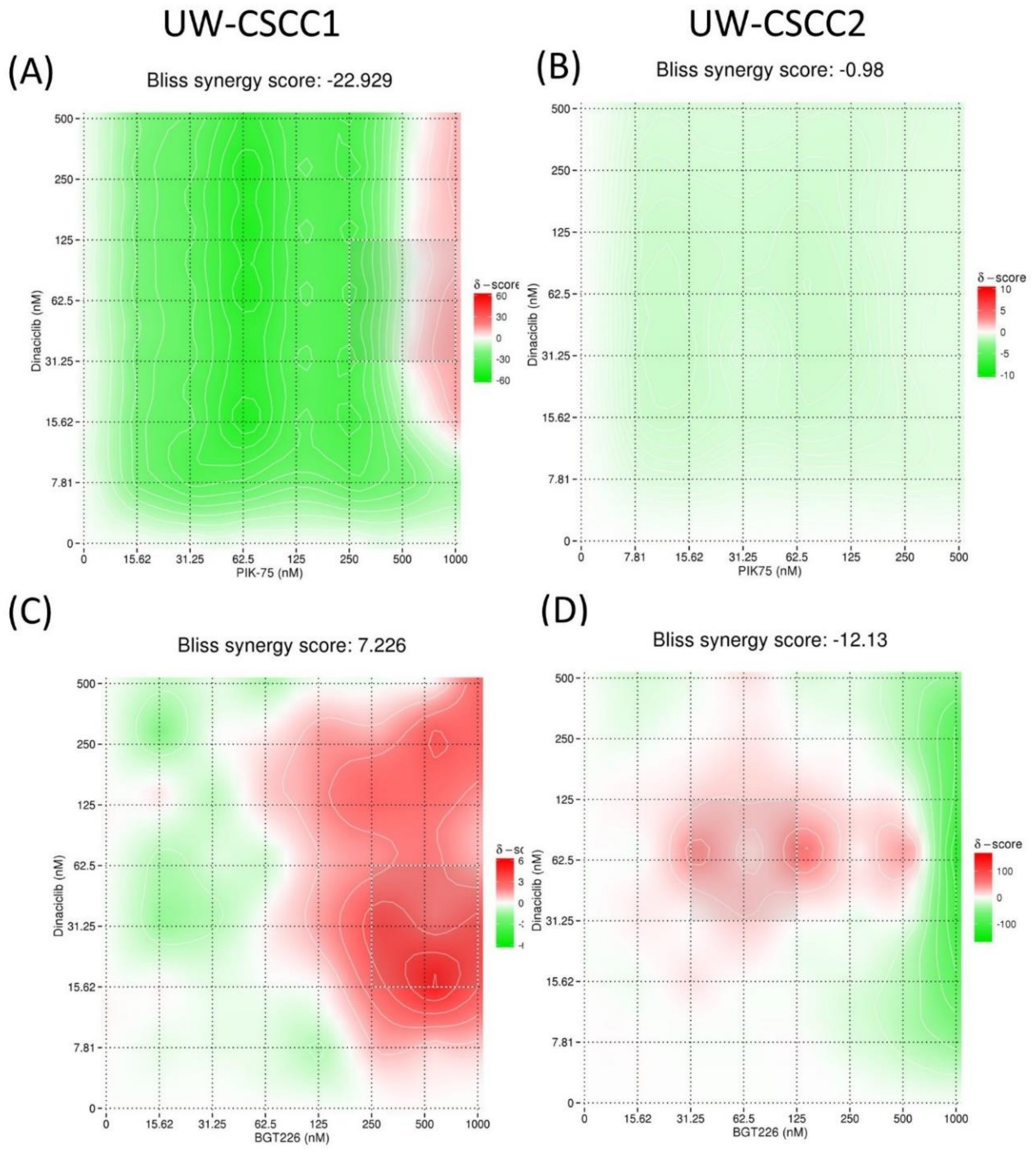

Figure S6: Synergistic effect profile of combination BGT226/ PIK-75 (PI3Ki) and Dinaciclib (CDKi) on spheroids derived from cSCC cell lines UW-CSCC1 and UW-CSCC2. UW-CSCC1 (A, C) and UW-CSCC2 (B, D) were treated with PIK-75 (A, B) or BGT226 (C, D) combined with Dinaciclib (A-D). Synergistic score matrices were generated *at 36 hours post*-treatment, implementing a BLISS analysis model (n=2). Bliss synergy score greater than 10, between 10 and -10, smaller than -10 signifies antagonistic, additive, or synergistic effect, respectively. In contrast to 2D synergy assays (Figure 1&2), the 3D analyses revealed PIK:DIN combinations having very little additivity/synergism and the BGT226:DIN combinations having greater synergy. UW-CSCC1 spheroids exhibited a dose-dependent response to both BGT226 and dinaciclib as single agents. UW-CSCC2 response to BGT:DIN was less obvious, although synergy analysis did reveal mild synergism at some concentration combinations.

3D drug viability methodology:

To determine synergistic concentrations in a 3D context, a 3D checkerboard assay was performed. Cells were seeded at 4,000 cells per well in round-bottomed, ultra-low attachment 96-well plates (Costar® Corning Incorporated, USA) in their relevant media. The cells were centrifuged at 209 x g for 3 min to facilitate their aggregation and incubated in a normoxic incubator (~21 % O_2, 37 °C). After 72 hours, cells were treated with a dilution of the PI3K inhibitors (PIK-75 or BGT226) [0-1,000 nM] against a dilution series of dinaciclib [0-500 nM]. Incucyte® Cytotox Green fluorescence intensity was used to determine the percentage effect of the drug mixtures relative to the control. Data was analysed as described in section 2.7.
