# Supplementary material for: PIK Your Poison: The Effects of Combining PI3K and CDK Inhibitors against Metastatic Cutaneous Squamous Cell Carcinoma In Vitro
Source: Cancers (Basel). 2024 Jan 15;16(2):370. doi: 10.3390/cancers16020370 (PMC10814950; doi:10.3390/cancers16020370)
Supplement: Supplementary file 1 [file cancers-16-00370-s001.zip › Supplementary Tables.pdf]

Supplementary Tables

Table S1. Primary and secondary antibodies used in Western blotting of cell lysates. All antibody dilutions were performed in TBST containing 2% skim milk powder.

**Primary Antibodies**			
Antibody	***Dilution***	***Manufacturer***	***Catalogue #***
Mouse anti-human GAPDH [D4C6R]	1:5000	Cell Signalling	97166S
Rabbit anti-human AKT [11E7]	1:1000	Cell Signalling	4685S
Rabbit anti-human phospho-AKT [Ser473; D9E]	1:1000	Cell Signalling	4060S
Rabbit anti-human p53	1:1000	Cell Signalling	9282S
Rabbit anti-human Cdk2 [E304]		Abcam	Ab32147
Rabbit anti-human Cdk6 [EPR4515]		Abcam	Ab124821
Rabbit anti-human Cyclin B1 [EPR17060]		Abcam	Ab181593
Rabbit anti-human Cyclin D1 [EPR2241]		Abcam	Ab134175
Rabbit anti-human p21 [EPR3993]		Abcam	Ab109199
Rabbit anti-human p27 KIP 1 [Y236]		Abcam	Ab32034

**Secondary Antibodies**			
Antibody	***Dilution***	***Manufacturer***	***Catalogue #***
Horseradish peroxidase-conjugated anti-mouse IgG	1:2000	Abcam	Ab205719
Horseradish peroxidase-conjugated anti-rabbit IgG	1:2000	Cell Signalling	7074S

Table S2. Recurrent (30%) coding mutations in metastatic cSCC and their representation in the UW-CSCC cell lines. Blank cells represent recurrent mutations not observed in the UW-CSCC cell lines.

Pathway	Recurrent alterations	UW-CSCC1		UW-CSCC2	
	(Frequency %, n = 25)	Alteration	Impact	Alteration	Impact
Cell cycle regulation	*TP53* (96)	missense<br>missense	p.Tyr220Ser<br>p.Thr125Pro	stop gained<br>missense variant	p.Arg342Ter<br>p.Glu285Lys
	CDKN2A (80)			stop gained	p.Arg58Ter
	PAK5 (68)		p.Tyr620Asn		
	CCNB3 (56)			missense	p.Glu1029Asp
	CDK5RAP2 (52)				
	PAK4 (44)		p.Pro52Leu		
	CDK12 (40)	synonymous<br>synonymous	p.Leu947=<br>p.Ser1329=		
	CIZ1 (40)				
	CDK14 (40)				
	CACUL1 (36)				
	RB1 (36)	inframe insertion	p.Gly89_Lys928delinsPheSer		
	CDK13 (32)				
PI3K-AKT-mTOR	*PIK3CG* (64)	missense	p.Pro200Leu	missense	p.Ser517Thr
	PIK3R5 (60)	missense	p.Phe551Leu		
	TSC2 (60)				
	PIK3C2B (52)	missense	p.Pro282Ser		
	MTOR (52)				
	PIK3R6 (52)	frameshift<br>frameshift<br>missense<br>complex substitution	p.Ile534ArgfsTer12<br>p.Met529PhefsTer21<br>p.Arg328Gln<br>p.Asp327Asn		
	RICTOR (48)				
	PIK3C2G (44)				
	PIK3AP1 (44)				
	RPTOR (40)				
	AKT3 (40)	missense	p.Glu338Lys	synonymous	p.Phe27=
	PIK3C2A (40)			missense	p.Phe127Leu
	PIK3CB (32)				
	PIKR1 (32)				
Apoptosis	*PRF1* (32)				
	CASP8 (32)				

Table S3. Drug combination experimental values (from representative experiments).

A) UW-CSCC1 – PIK-75:Dinaciclib treatment

				Dose-reduction Index	
**PIK-75 (nM)**	**Dinaciclib (nM)**	**Fa**	**CI**	**PIK-75**	**Dinaciclib**
55	4.25	0.008065	0.999	1.928	2.083
110	8.5	0.816992	1.016	1.919	2.022
220	17	0.961652	1.688	1.158	1.212
440	34	0.98715	2.995	0.654	0.682
880	68	0.99999	2.777	0.715	0.725

CI = combination index, Fa = effect

B) UW-CSCC2 – PIK-75:Dinaciclib treatment

				Dose-reduction Index	
**PIK-75 (nM)**	**Dinaciclib (nM)**	**Fa**	**CI**	**PIK-75**	**Dinaciclib**
14	4.25	0.092054	0.73	1.488	17.251
28	8.5	0.501674	0.873	1.279	10.96
56	17	0.920443	1.02	1.138	7.071
112	34	0.959426	1.748	0.673	3.809
224	68	0.99999	0.634	2.415	4.552

CI = combination index, Fa = effect

C) UW-CSCC1 – BGT226:Dinaciclib treatment

				Dose-reduction Index	
**BGT226 (nM)**	**Dinaciclib (nM)**	**Fa**	**CI**	**BGT226**	**Dinaciclib**
50	4.2517	0.0831694	1.136	1.188	3.391
100	8.5034	0.217762	1.780	0.795	1.917
200	17.0068	0.958022	1.447	1.245	1.552

400	34.0136	0.998295	1.596	1.443	1.107
800	68.0272	0.99999	1.395	2.736	0.972

CI = combination index, Fa = effect

D) UW-CSCC2 – BGT226:Dinaciclib treatment

				**Dose-reduction Index**	
**BGT226 (nM)**	**Dinaciclib (nM)**	**Fa**	**CI**	**BGT226**	**Dinaciclib**
50	4.25026	0.473709	2.125	0.527	4.400
100	8.50051	0.479666	4.224	0.265	2.207
200	17.001	0.670393	7.007	0.162	1.224
400	34.002	0.944735	8.507	0.138	0.810
800	68.0041	0.99999	2.454	0.601	1.265

CI = combination index, Fa = effect
