# Supplementary figures and images for: PIK Your Poison: The Effects of Combining PI3K and CDK Inhibitors against Metastatic Cutaneous Squamous Cell Carcinoma In Vitro (part 2 of 2)
Source: Cancers (Basel). 2024 Jan 15;16(2):370. doi: 10.3390/cancers16020370 (PMC10814950; doi:10.3390/cancers16020370)

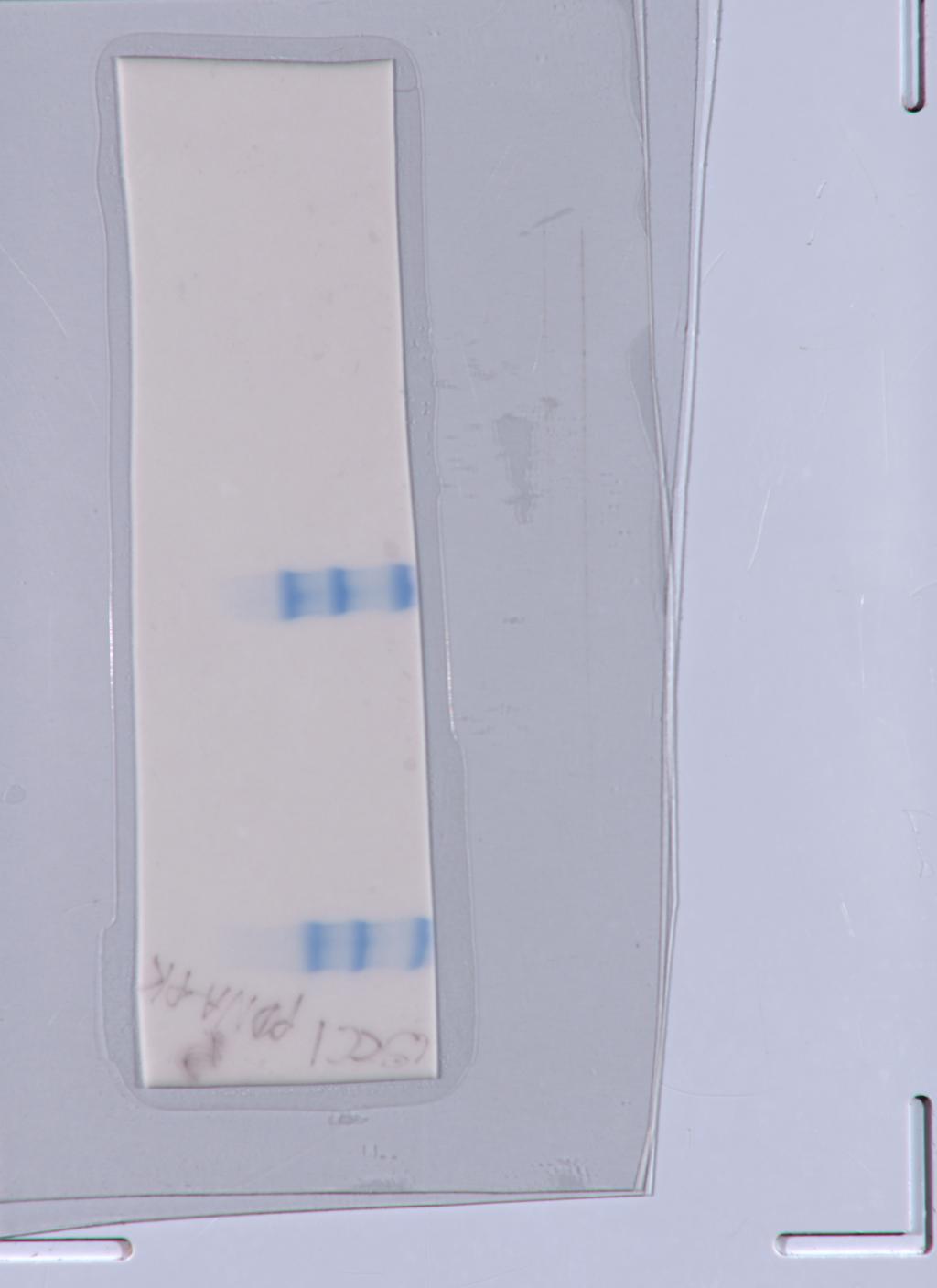

Supplement: Supplementary file 1 [file cancers-16-00370-s001.zip › JPpDNA cs1 30-5 10mf 2022.05.30_14.56.51_Ch-Marker.jpg]

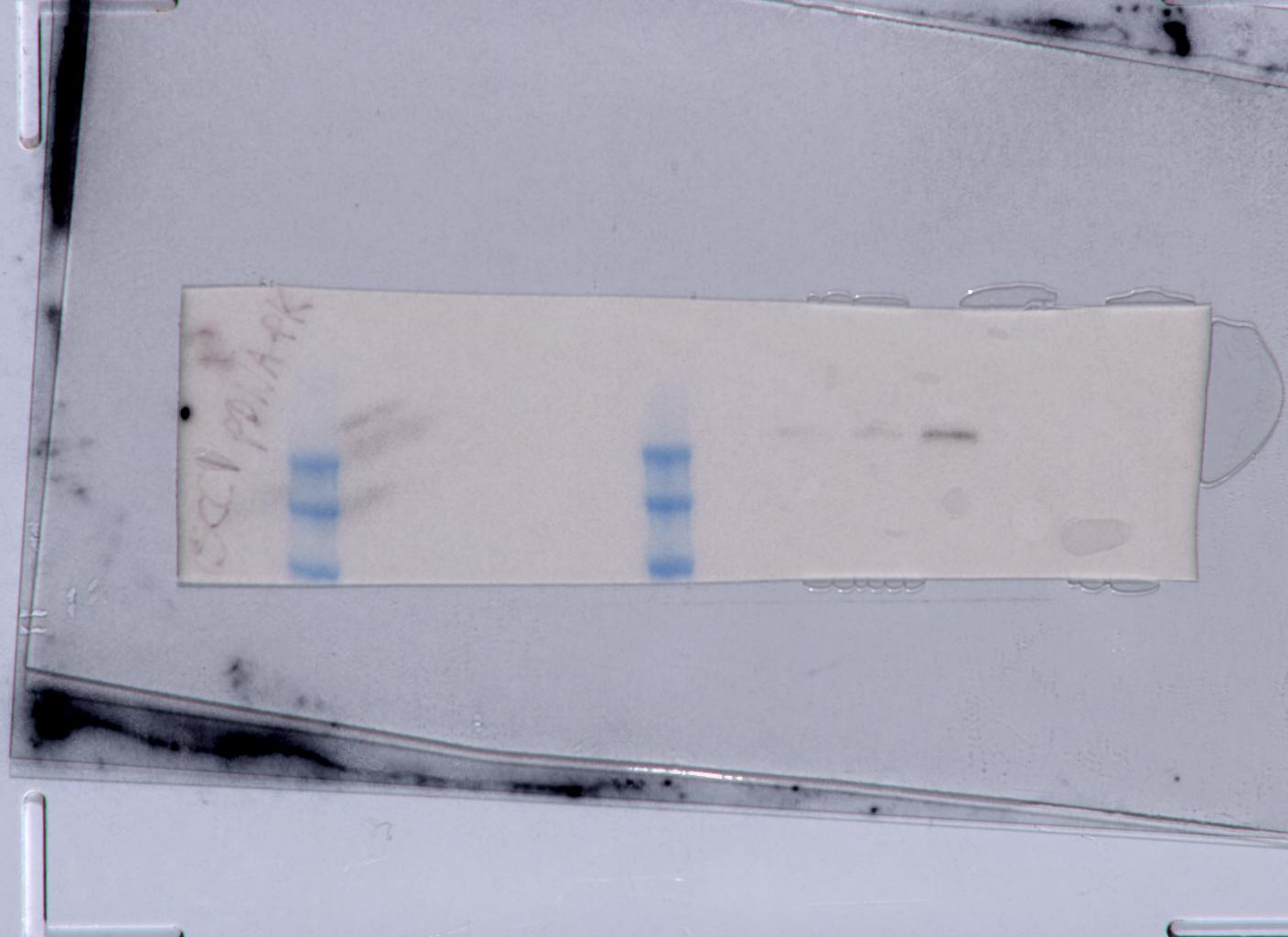

Supplement: Supplementary file 1 [file cancers-16-00370-s001.zip › JPpDNA cs1 30-5 2mfe 2022.05.30_14.51.30_Ch+Marker.jpg]

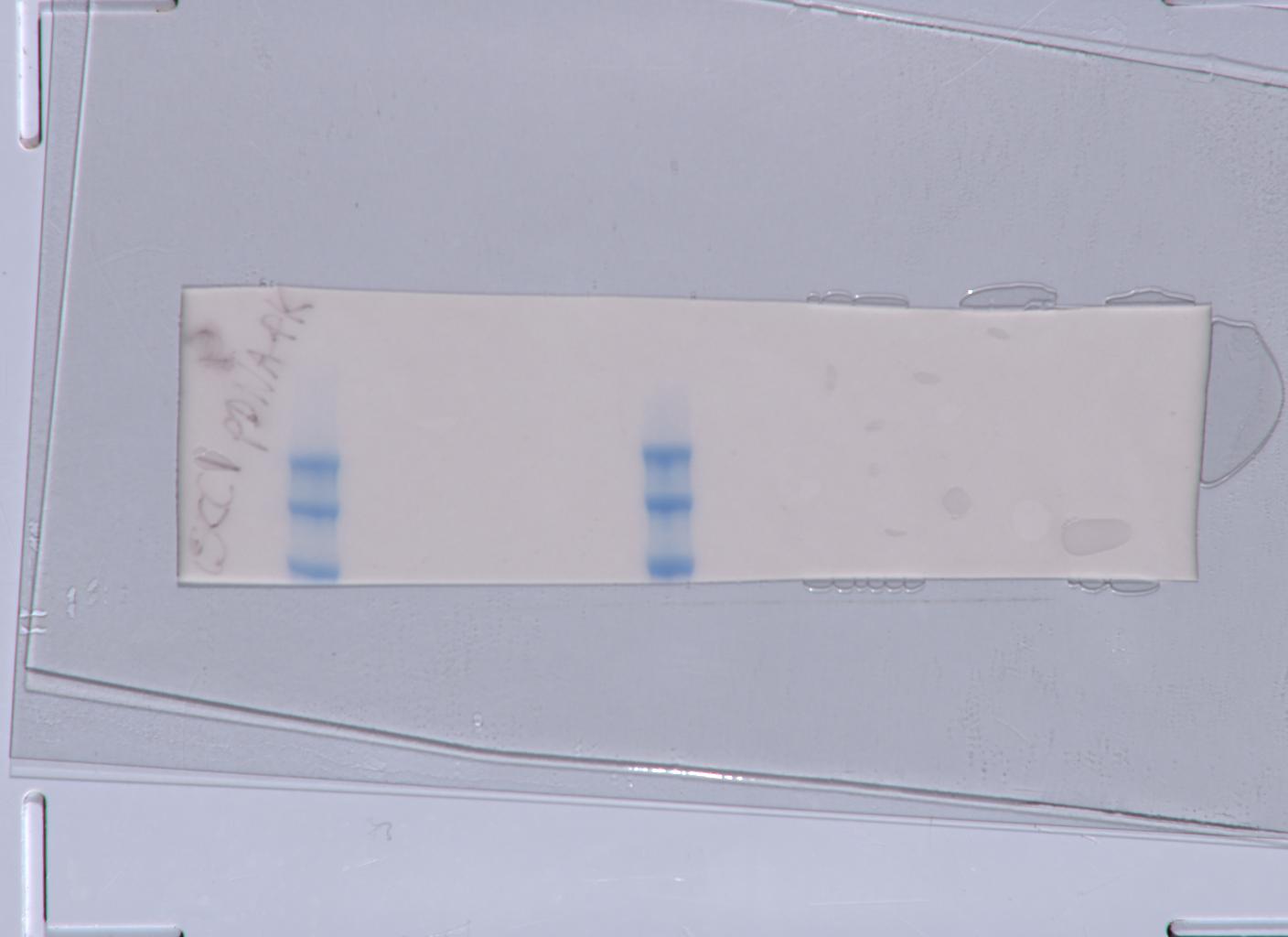

Supplement: Supplementary file 1 [file cancers-16-00370-s001.zip › JPpDNA cs1 30-5 2mfe 2022.05.30_14.51.30_Ch-Marker.jpg]

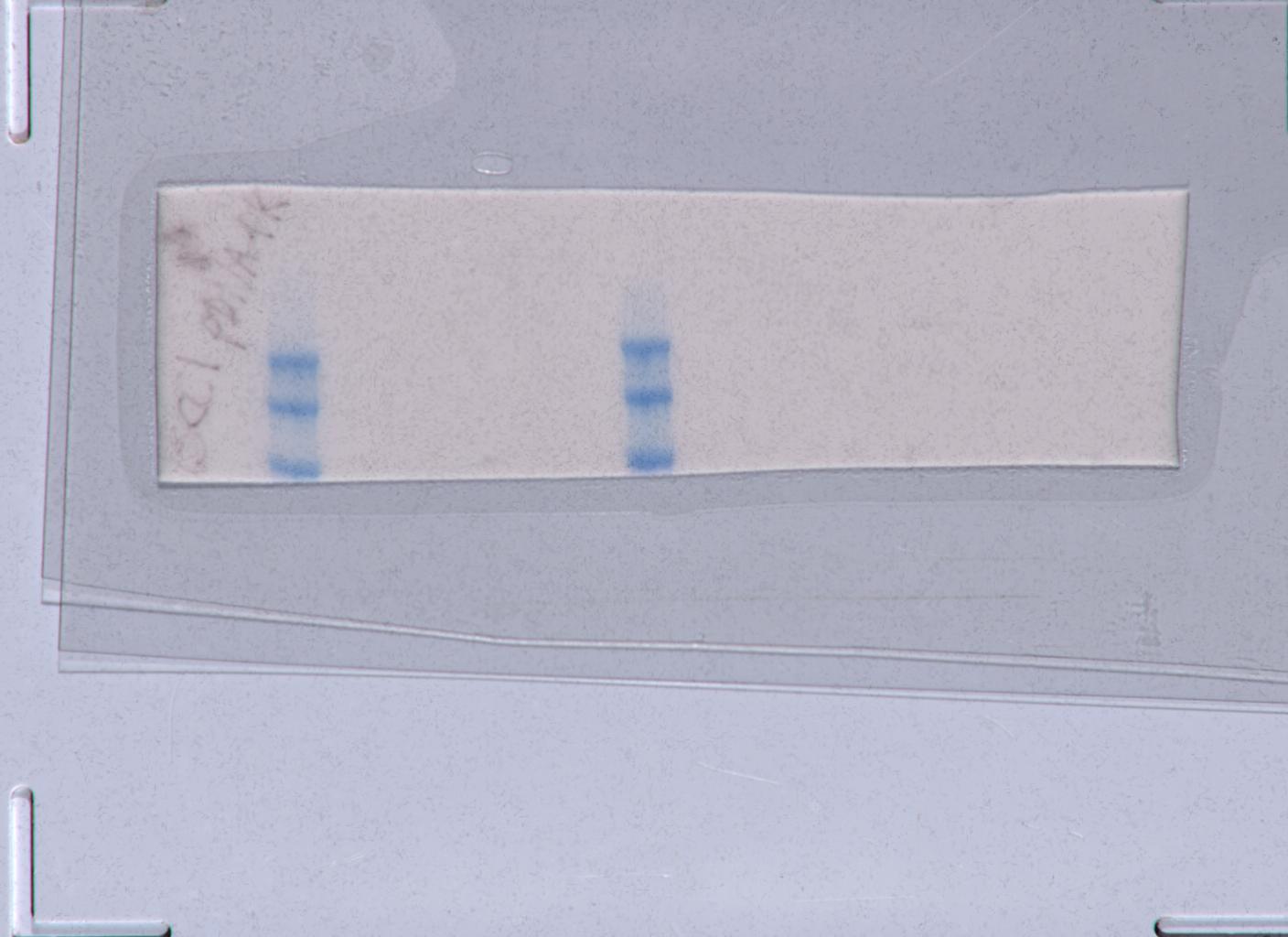

Supplement: Supplementary file 1 [file cancers-16-00370-s001.zip › JPpDNAPK cs1 30-5 3m 2022.05.30_14.26.06_Ch+Marker.jpg]

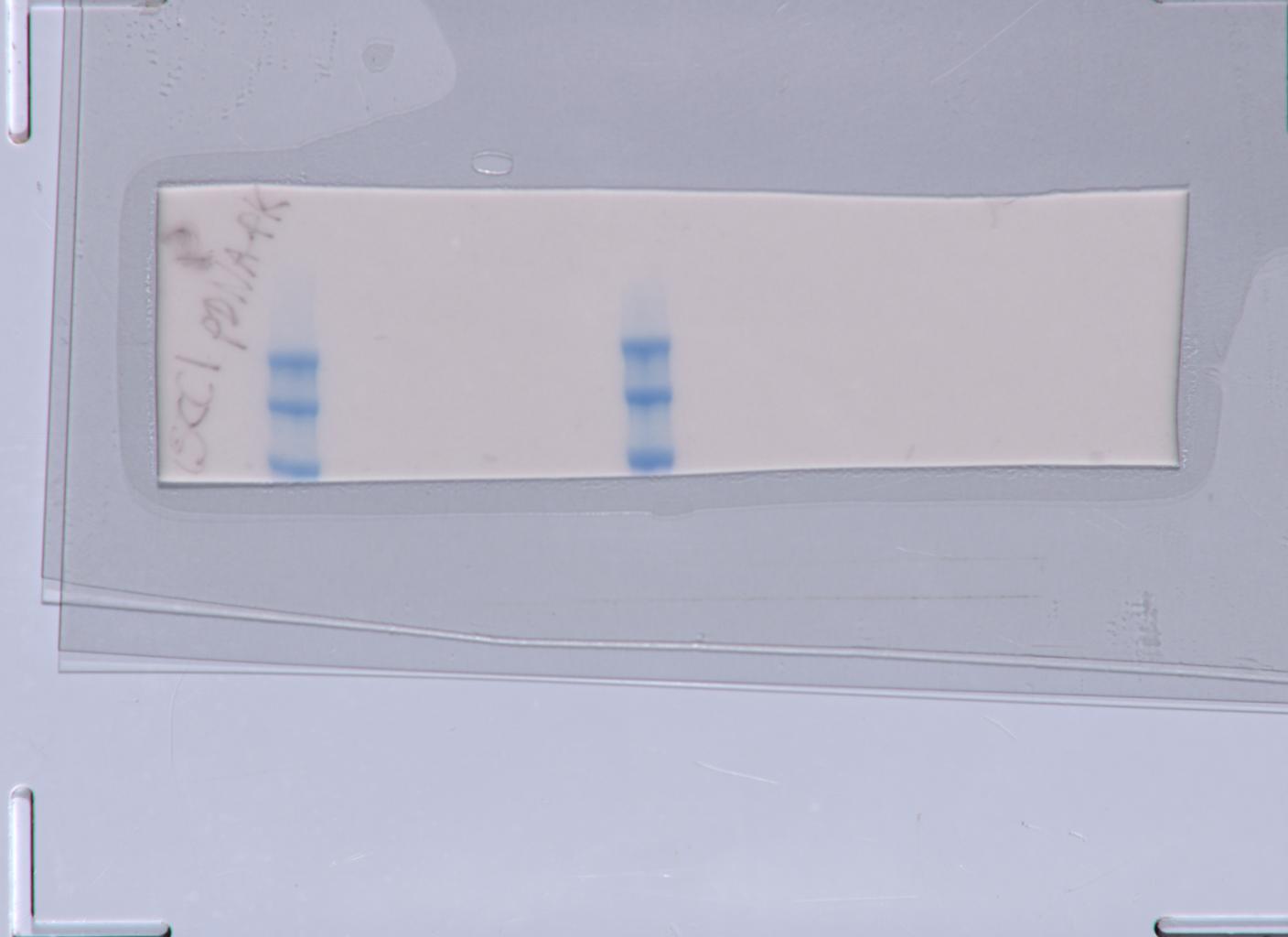

Supplement: Supplementary file 1 [file cancers-16-00370-s001.zip › JPpDNAPK cs1 30-5 3m 2022.05.30_14.26.06_Ch-Marker.jpg]

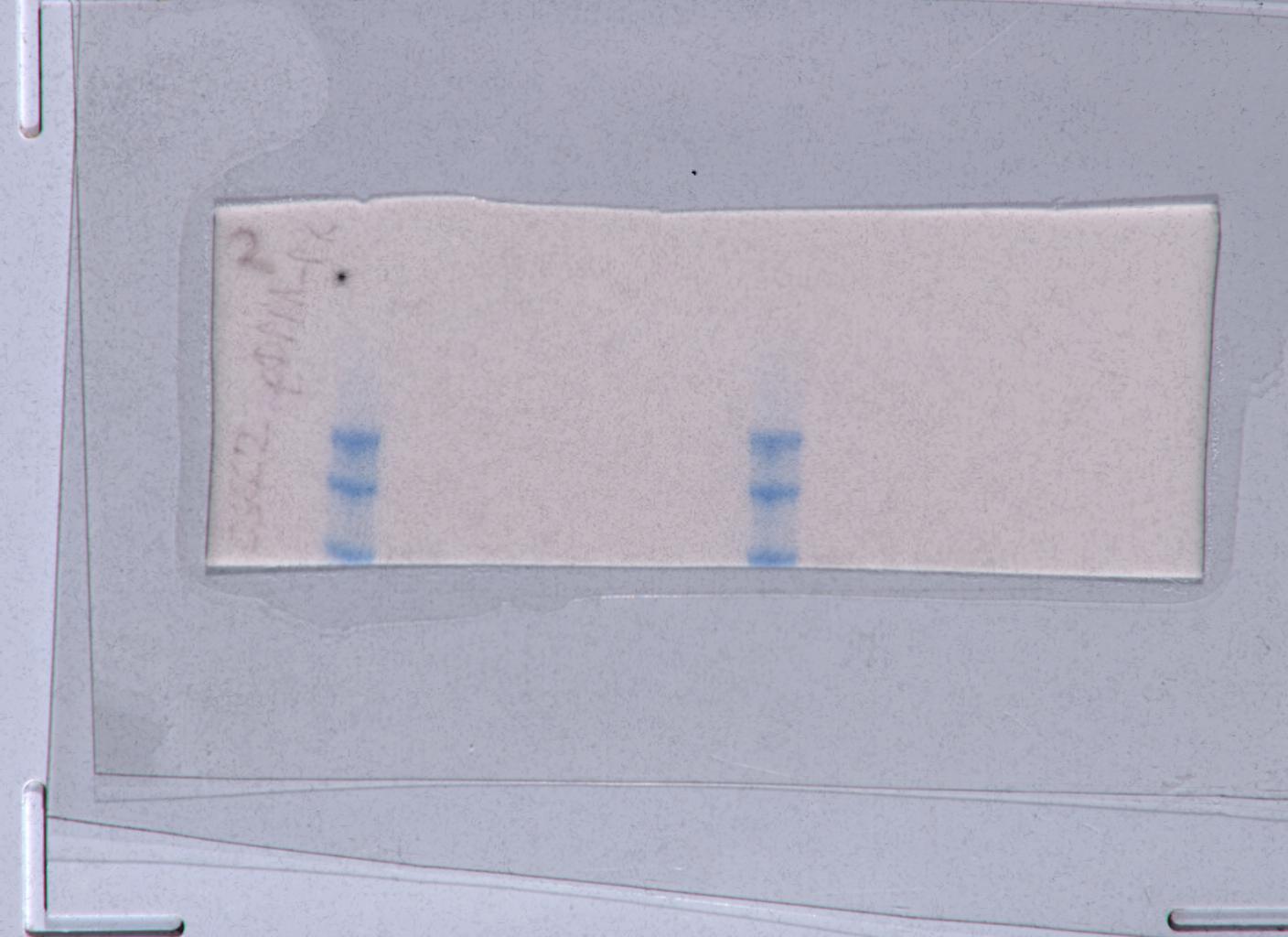

Supplement: Supplementary file 1 [file cancers-16-00370-s001.zip › JPpDNAPK cs2 30-5 3m 2022.05.30_14.32.22_Ch+Marker.jpg]

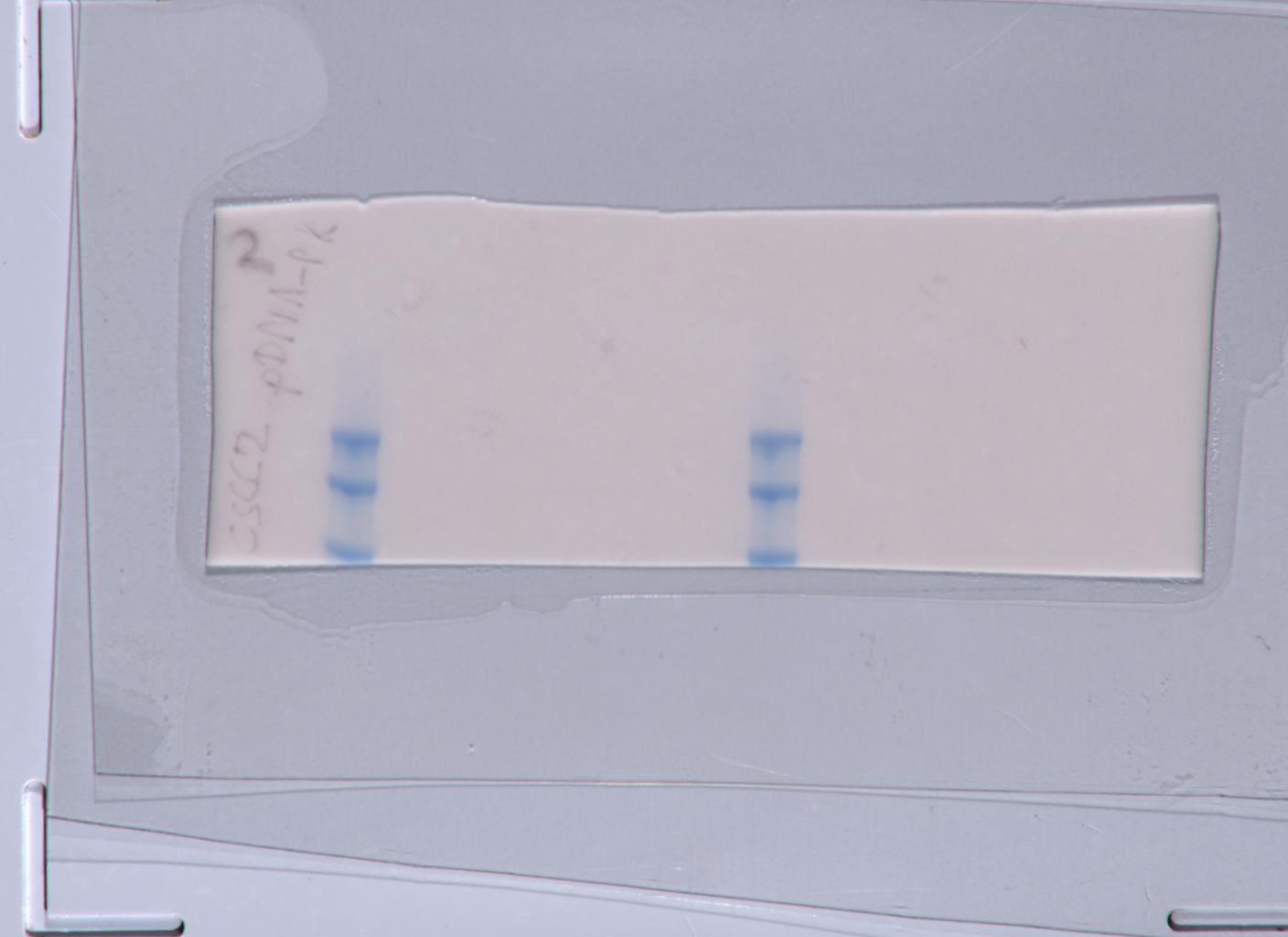

Supplement: Supplementary file 1 [file cancers-16-00370-s001.zip › JPpDNAPK cs2 30-5 3m 2022.05.30_14.32.22_Ch-Marker.jpg]

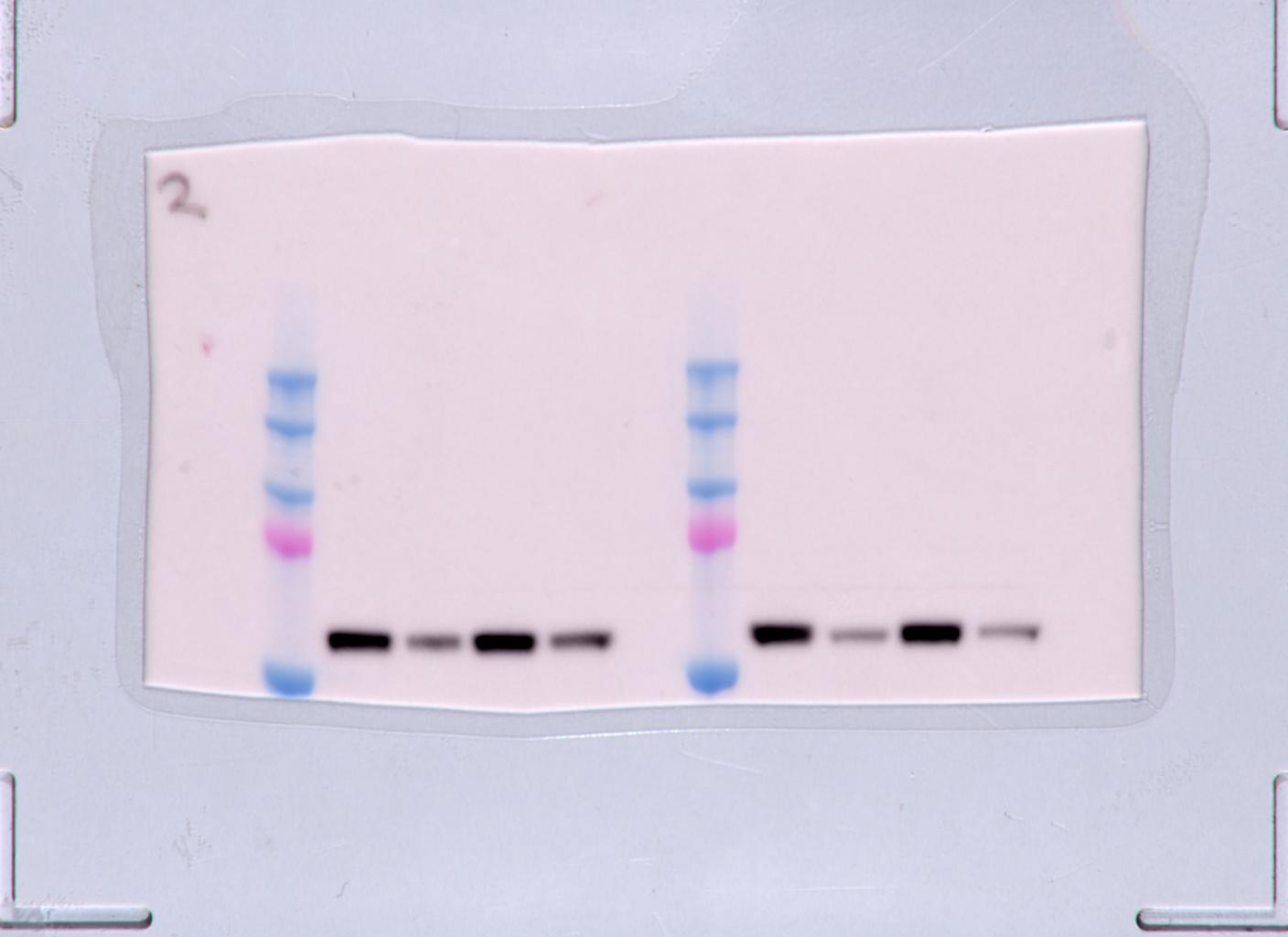

Supplement: Supplementary file 1 [file cancers-16-00370-s001.zip › pAKT cscc2 31-3-22 J 2022.03.31_15.52.39_Ch+Marker.jpg]

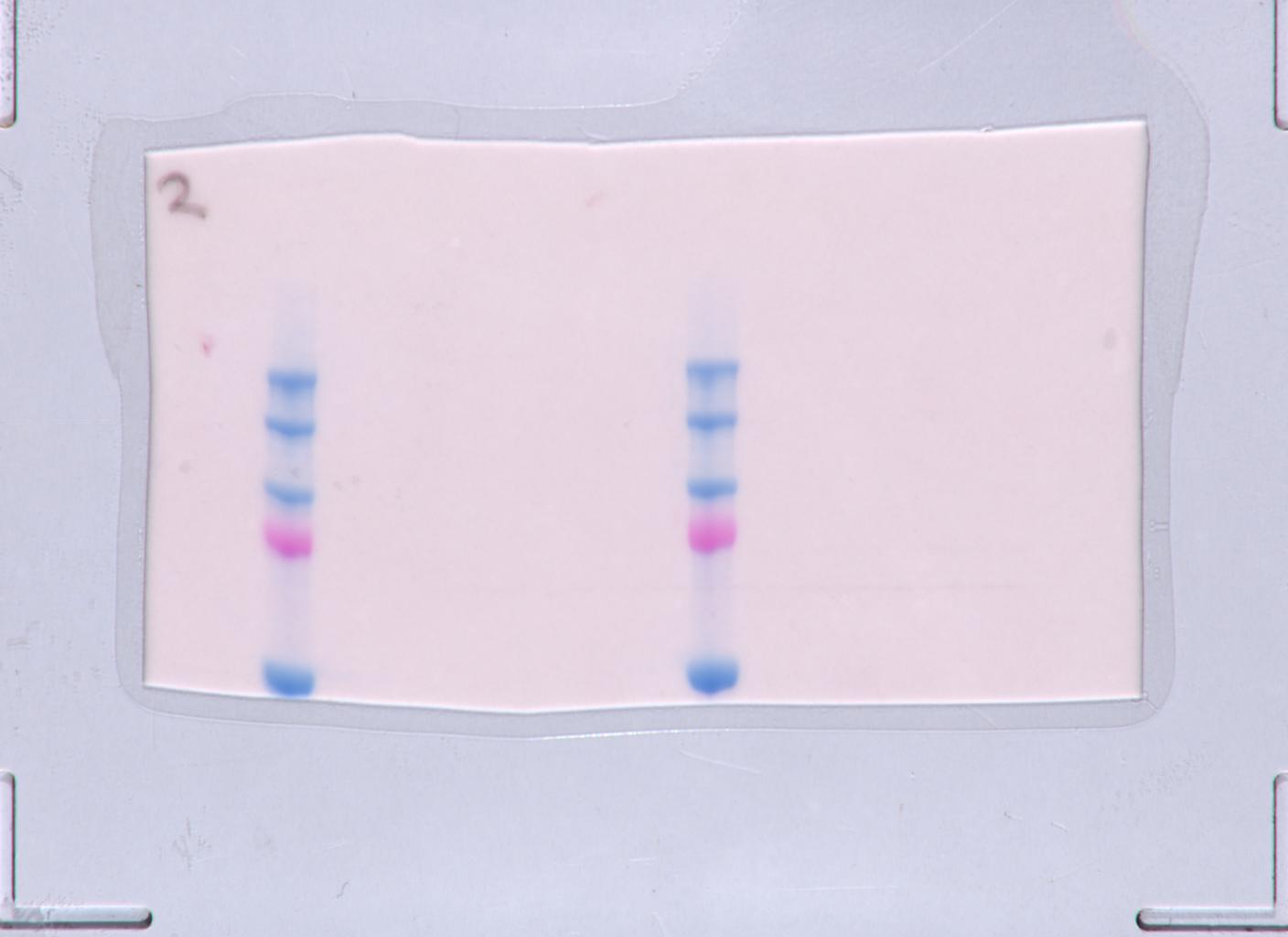

Supplement: Supplementary file 1 [file cancers-16-00370-s001.zip › pAKT cscc2 31-3-22 J 2022.03.31_15.52.39_Ch-Marker.jpg]
