# Supplementary figures and images for: PIK Your Poison: The Effects of Combining PI3K and CDK Inhibitors against Metastatic Cutaneous Squamous Cell Carcinoma In Vitro (part 1 of 2)
Source: Cancers (Basel). 2024 Jan 15;16(2):370. doi: 10.3390/cancers16020370 (PMC10814950; doi:10.3390/cancers16020370)

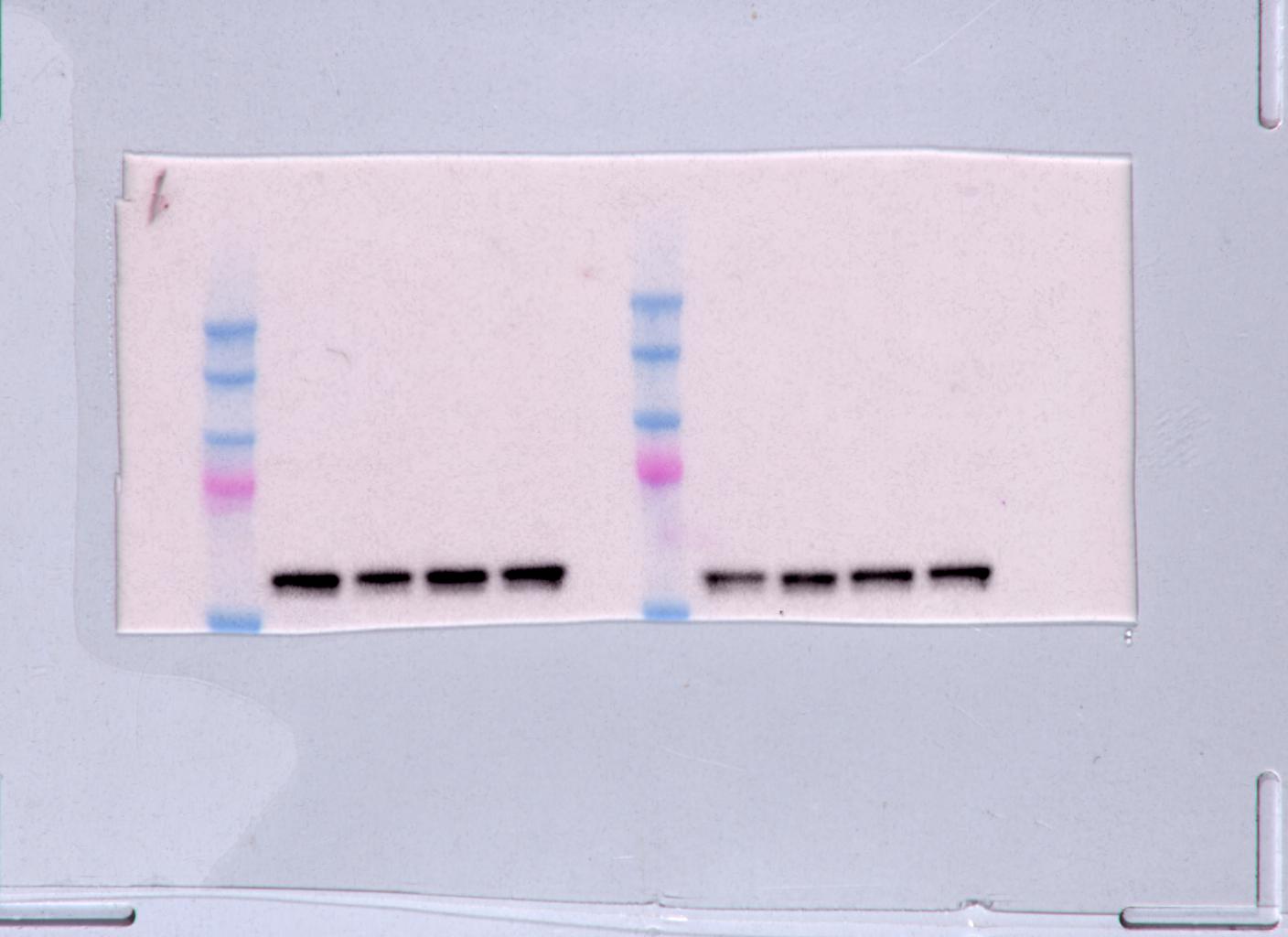

Supplement: Supplementary file 1 [file cancers-16-00370-s001.zip › AKT cscc2 31-3-22 JP 2022.03.31_15.47.06_Ch+Marker.jpg]

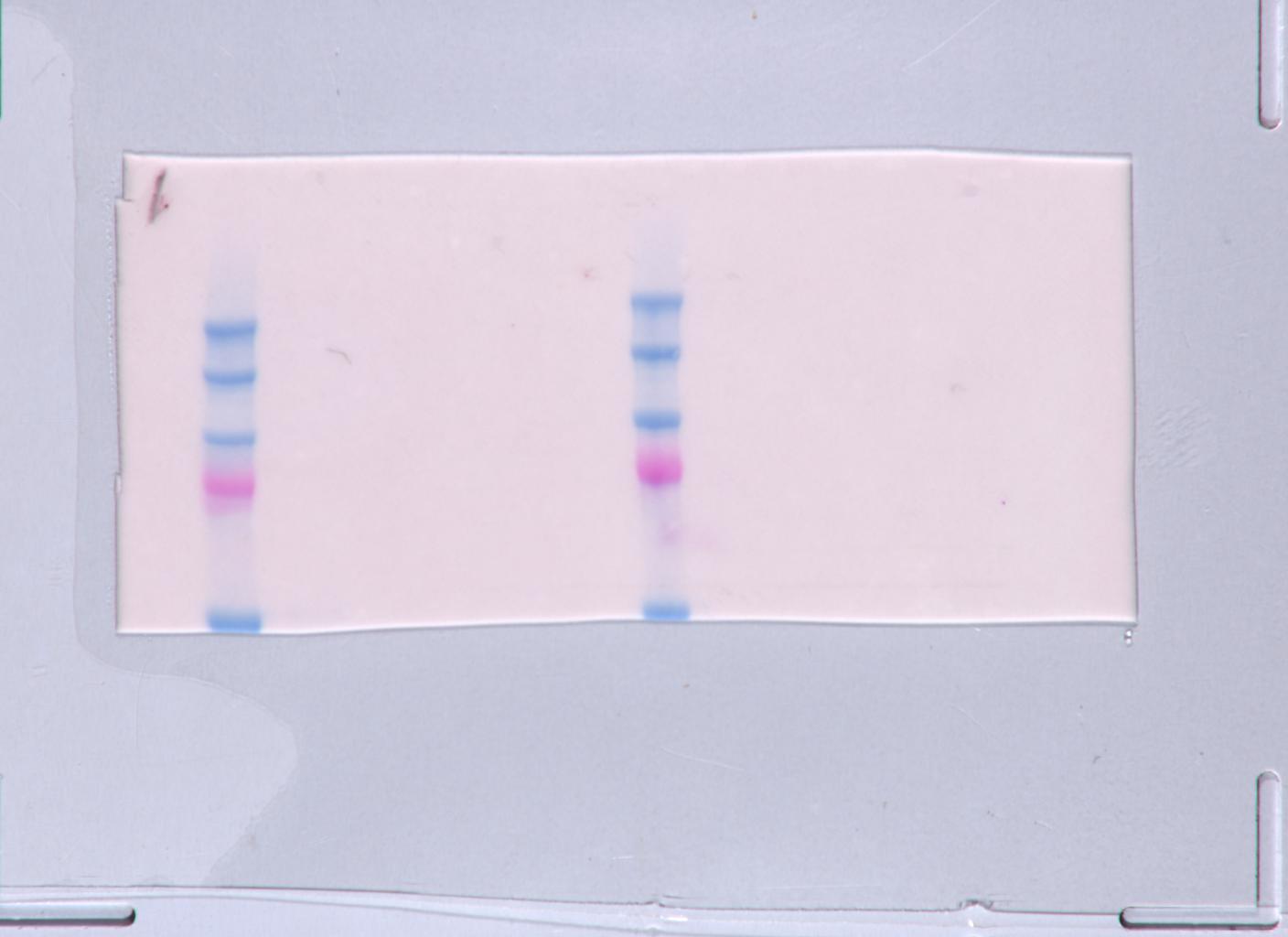

Supplement: Supplementary file 1 [file cancers-16-00370-s001.zip › AKT cscc2 31-3-22 JP 2022.03.31_15.47.06_Ch-Marker.jpg]

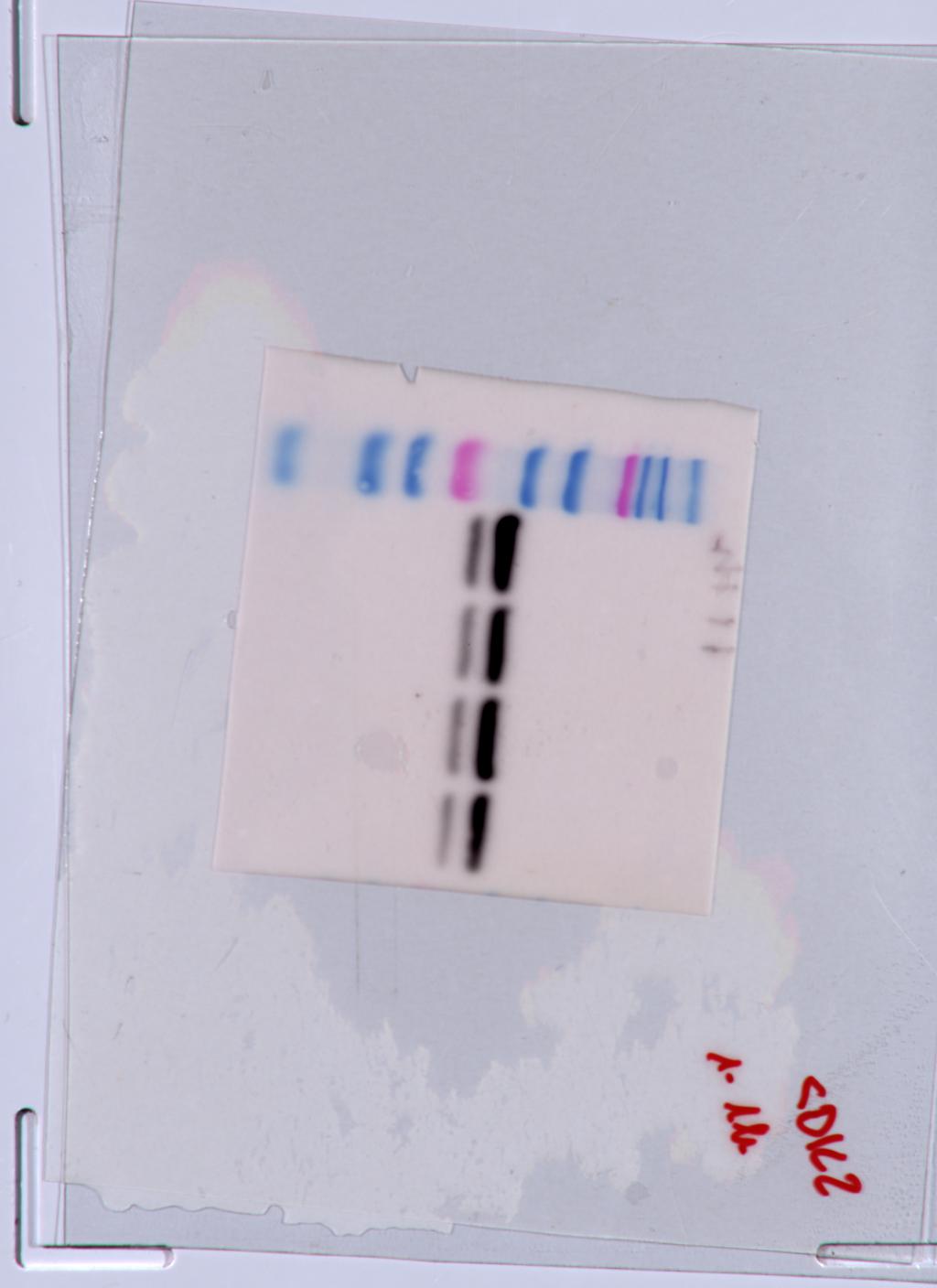

Supplement: Supplementary file 1 [file cancers-16-00370-s001.zip › BGJP_ 1_1h_CPDM_CDK2 2022.12.09_11.55.33_Ch+Marker.jpg]

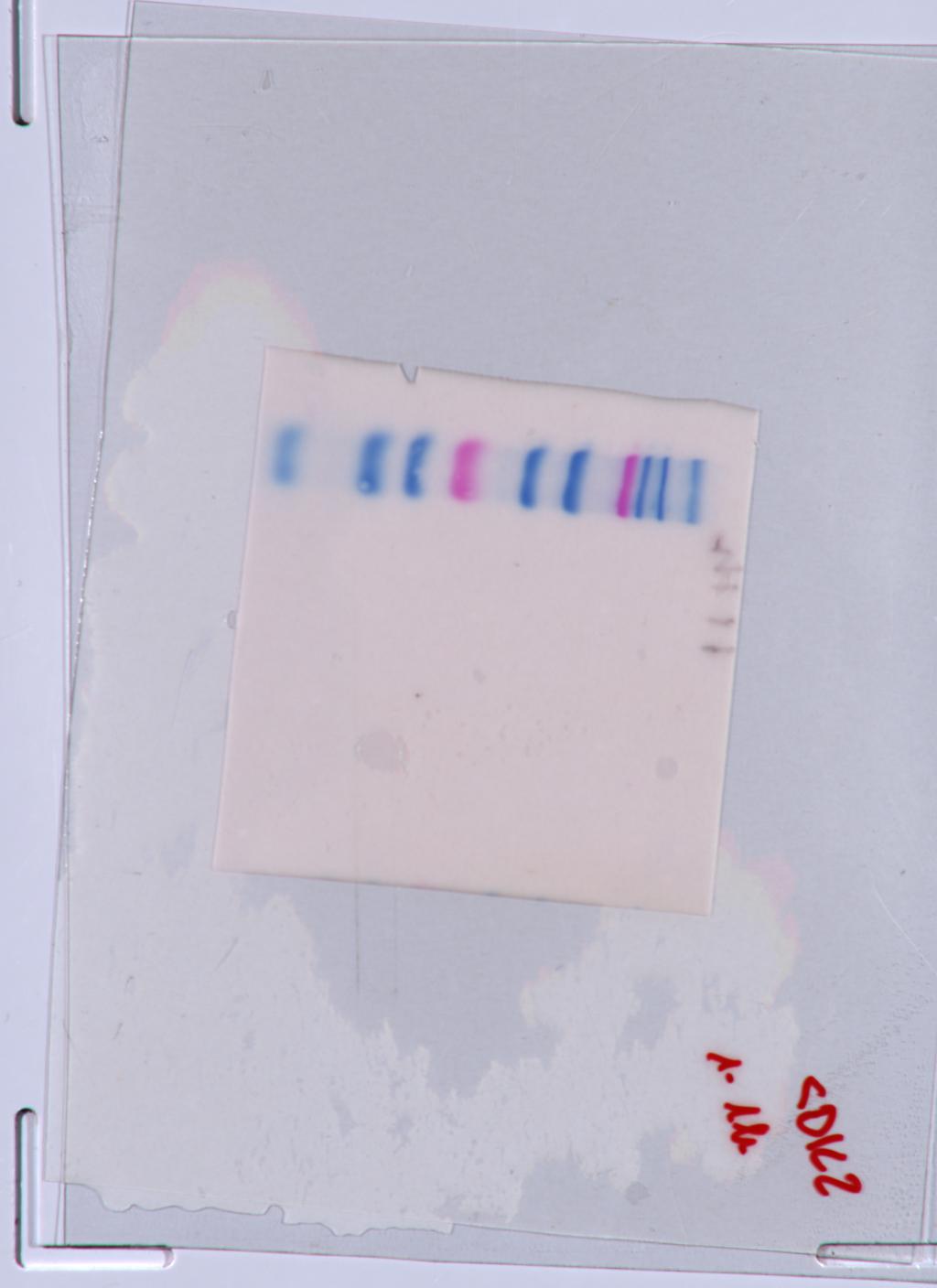

Supplement: Supplementary file 1 [file cancers-16-00370-s001.zip › BGJP_ 1_1h_CPDM_CDK2 2022.12.09_11.55.33_Ch-Marker.jpg]

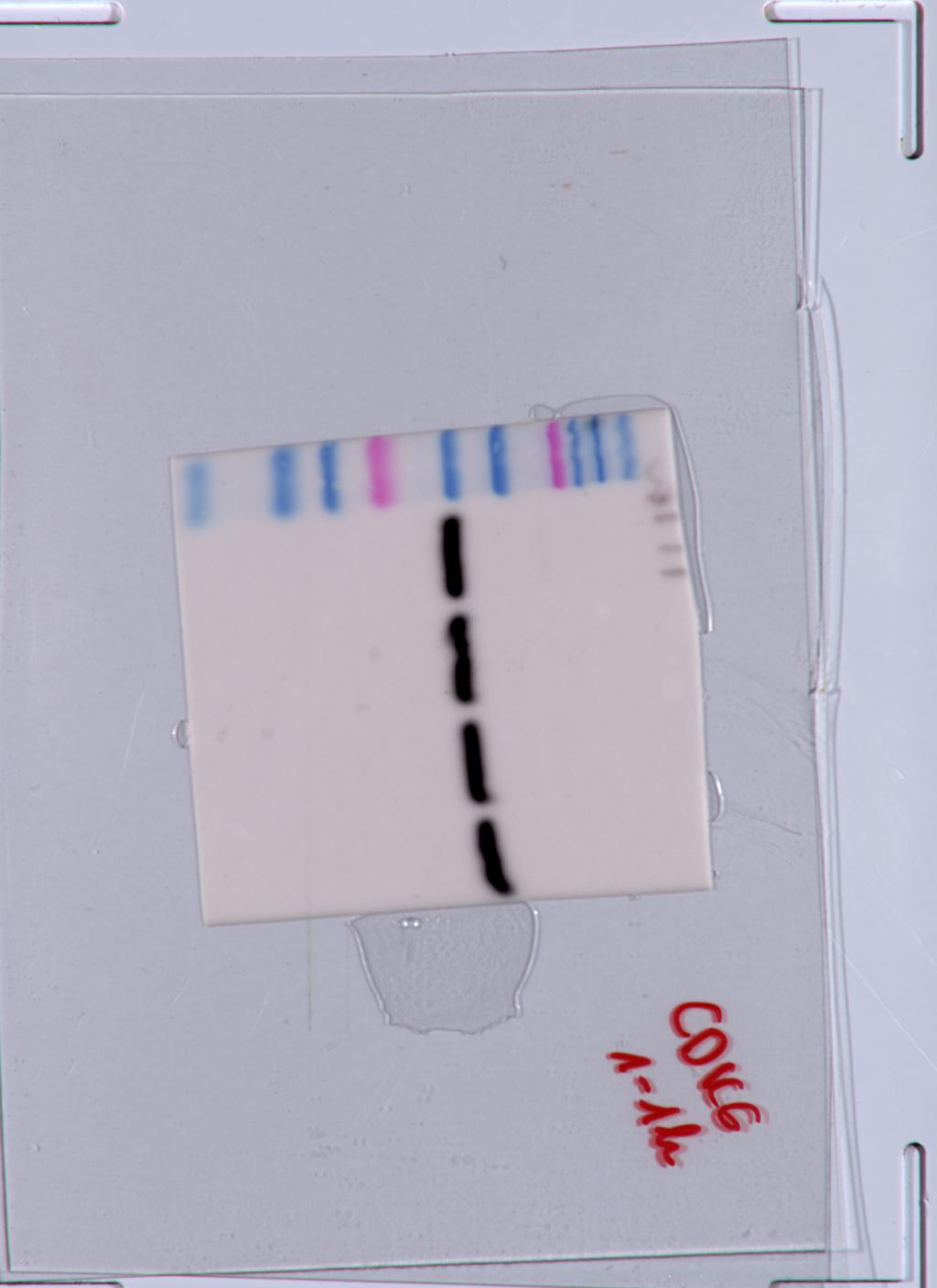

Supplement: Supplementary file 1 [file cancers-16-00370-s001.zip › BGJP_ 1_1h_CPDM_CDK6 2022.12.09_12.12.53_Ch+Marker.jpg]

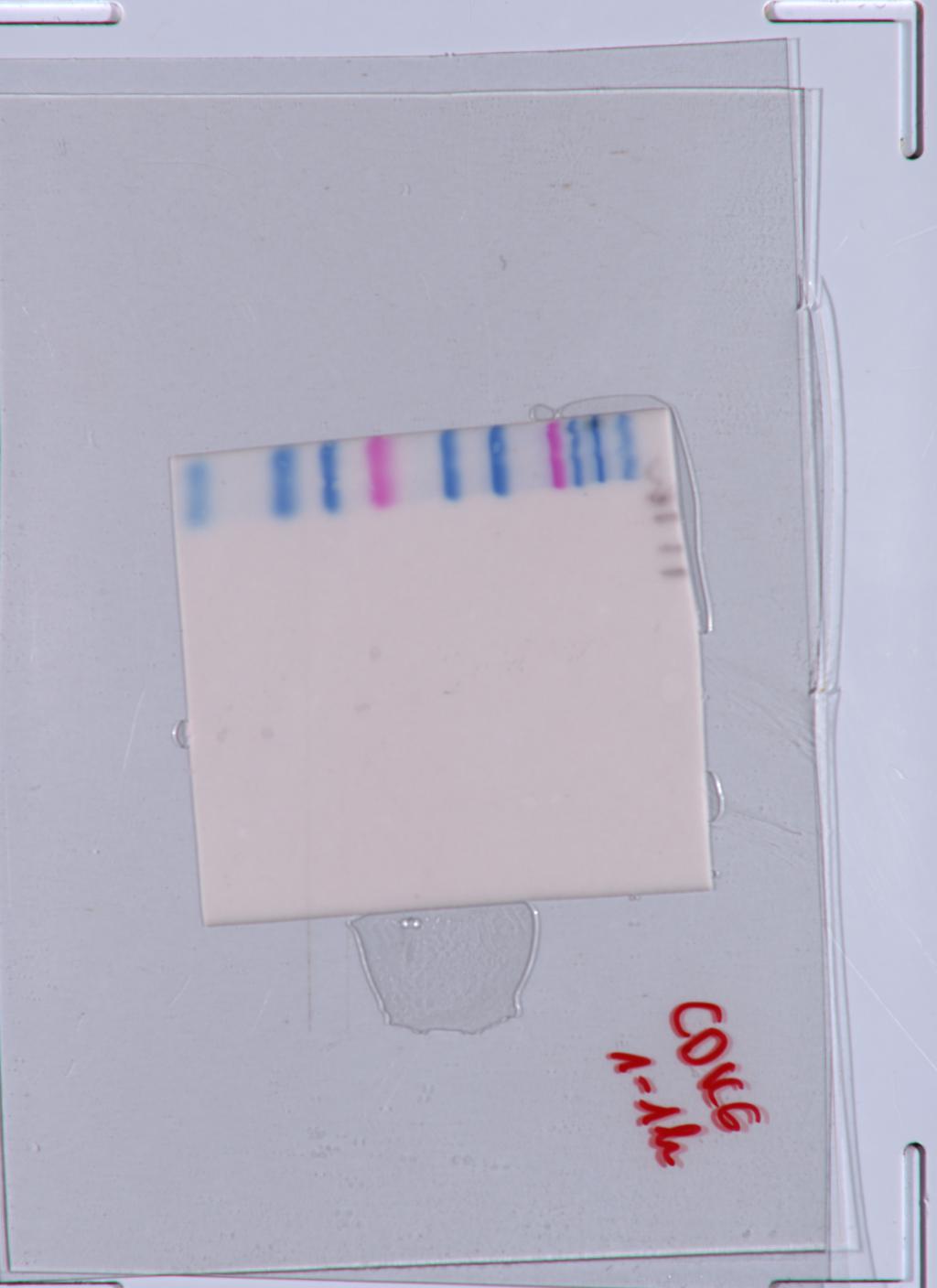

Supplement: Supplementary file 1 [file cancers-16-00370-s001.zip › BGJP_ 1_1h_CPDM_CDK6 2022.12.09_12.12.53_Ch-Marker.jpg]

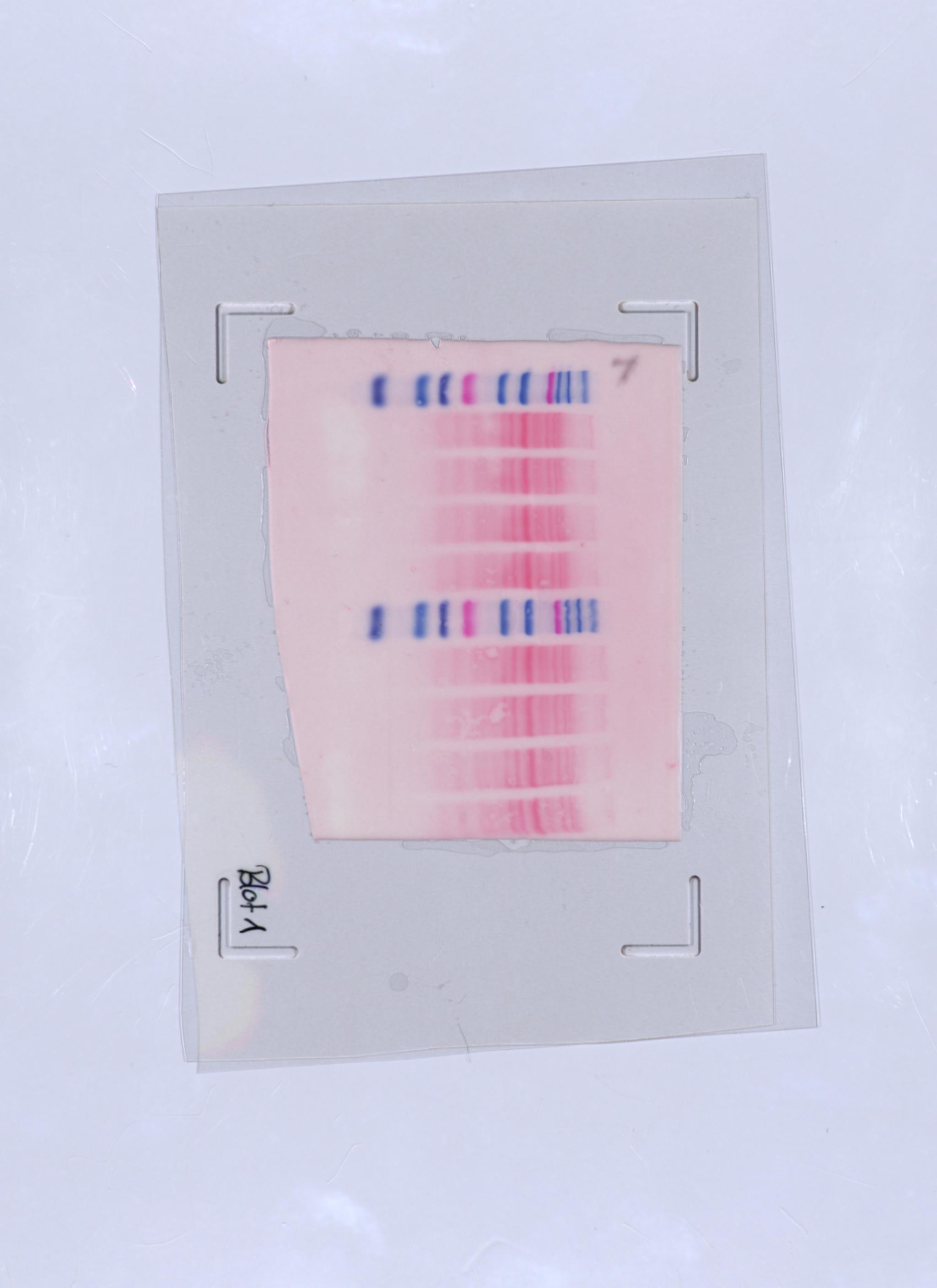

Supplement: Supplementary file 1 [file cancers-16-00370-s001.zip › BGJP_ 1_1h_CPDM_Ponc 2022.12.08_14.44.43_Co.jpg]

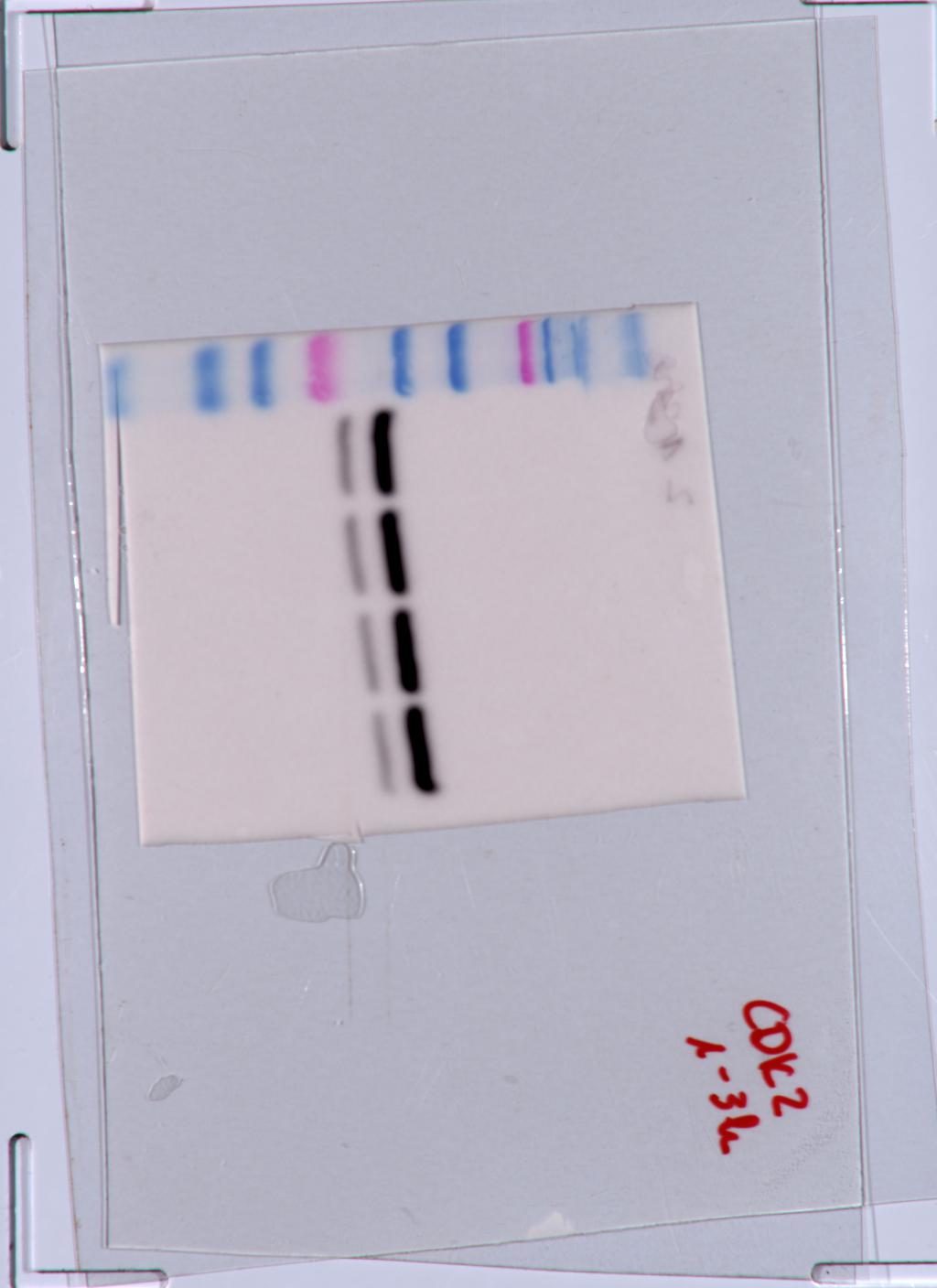

Supplement: Supplementary file 1 [file cancers-16-00370-s001.zip › BGJP_ 1_3h_CPDM_CDK2 2022.12.09_12.01.00_Ch+Marker.jpg]

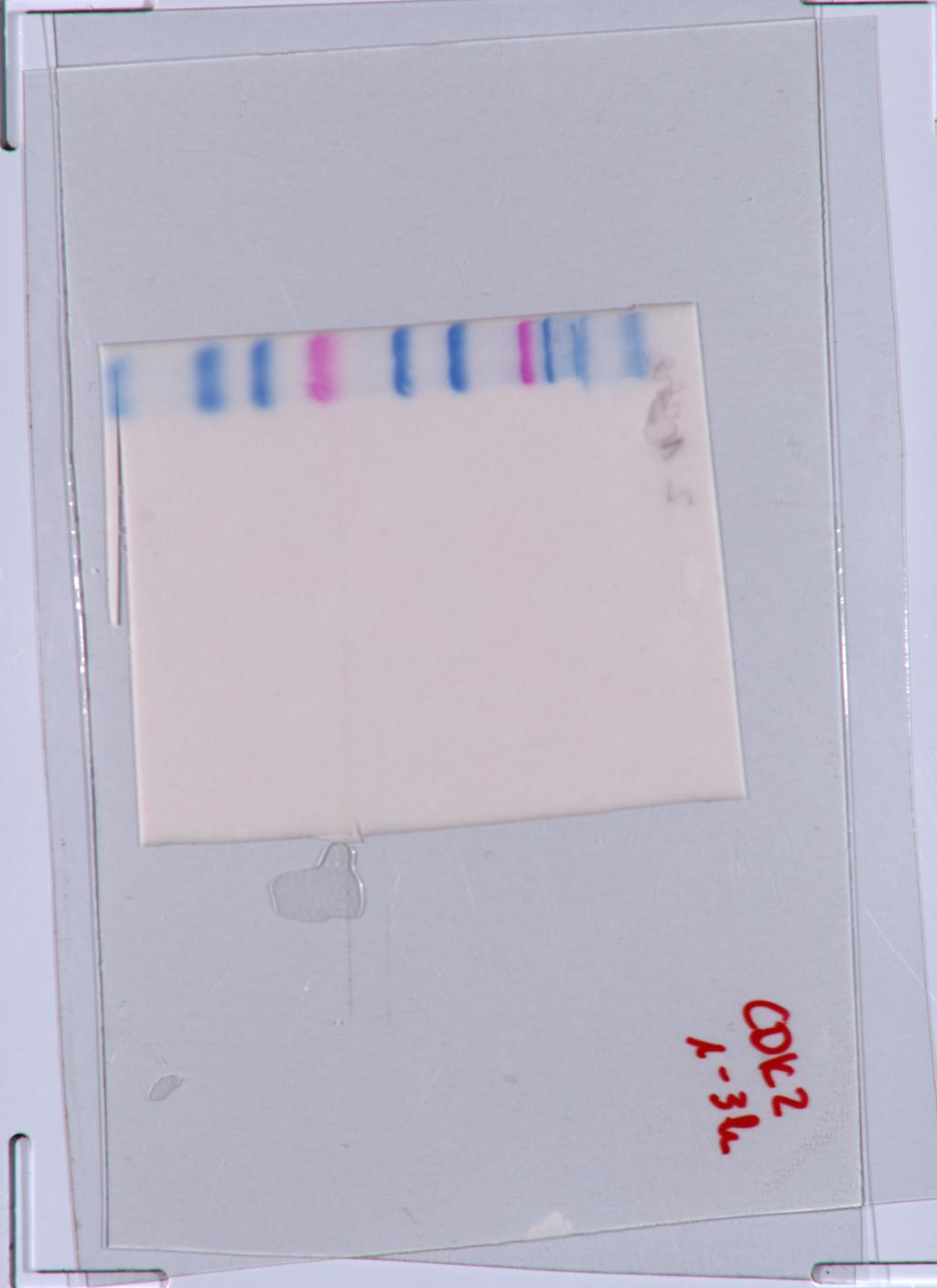

Supplement: Supplementary file 1 [file cancers-16-00370-s001.zip › BGJP_ 1_3h_CPDM_CDK2 2022.12.09_12.01.00_Ch-Marker.jpg]

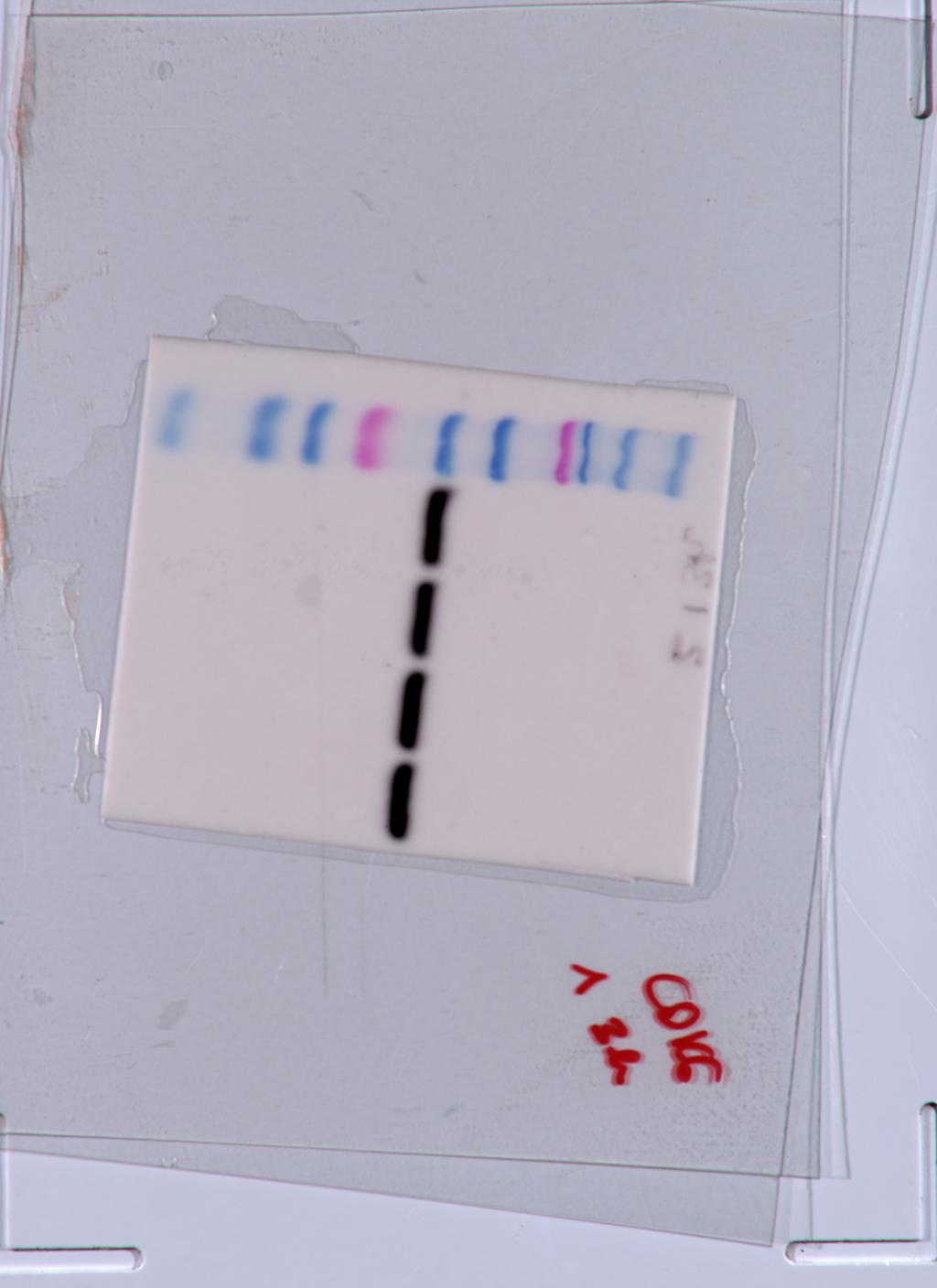

Supplement: Supplementary file 1 [file cancers-16-00370-s001.zip › BGJP_ 1_3h_CPDM_CDK6 2022.12.09_12.05.13_Ch+Marker.jpg]

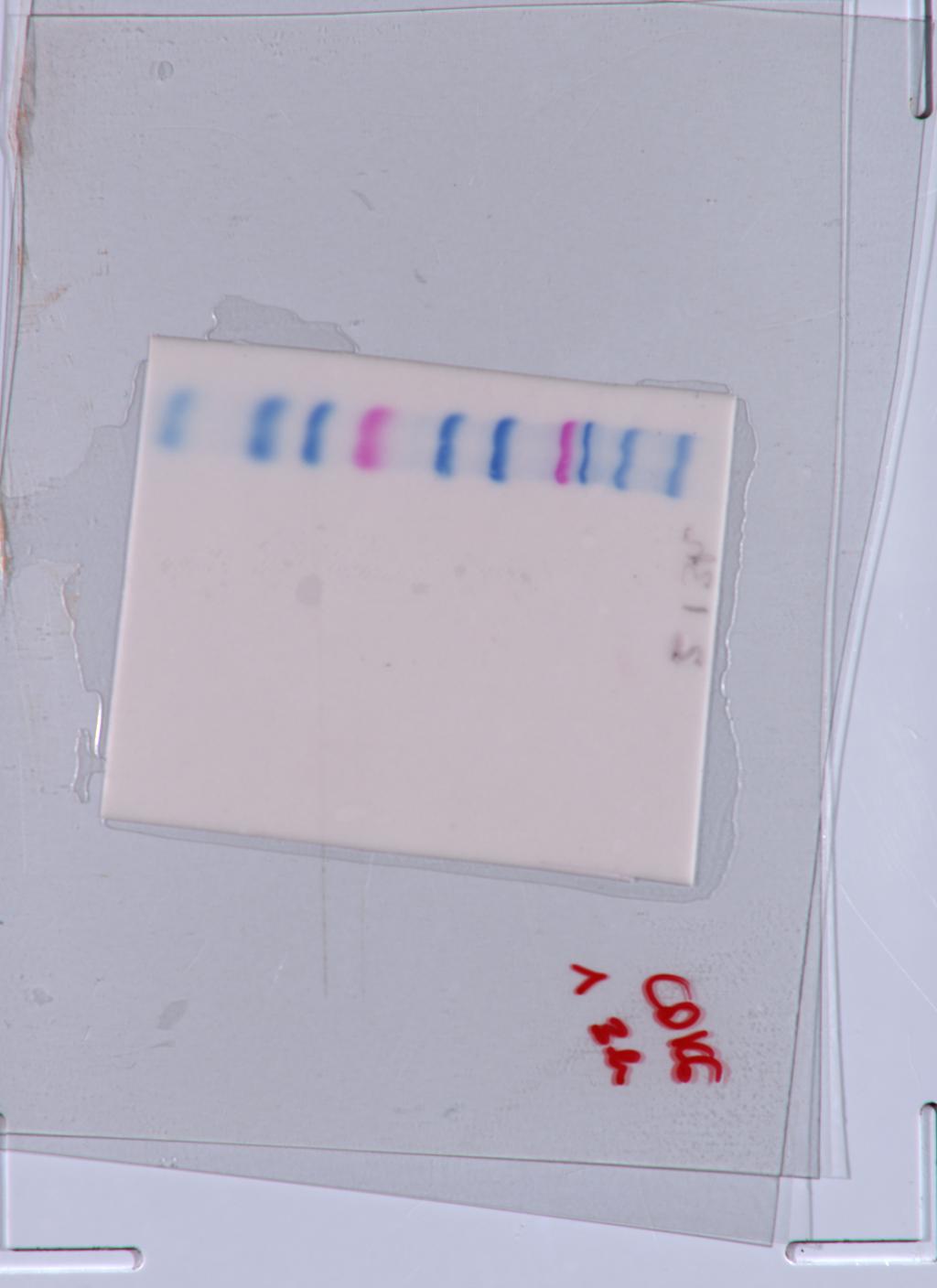

Supplement: Supplementary file 1 [file cancers-16-00370-s001.zip › BGJP_ 1_3h_CPDM_CDK6 2022.12.09_12.05.13_Ch-Marker.jpg]

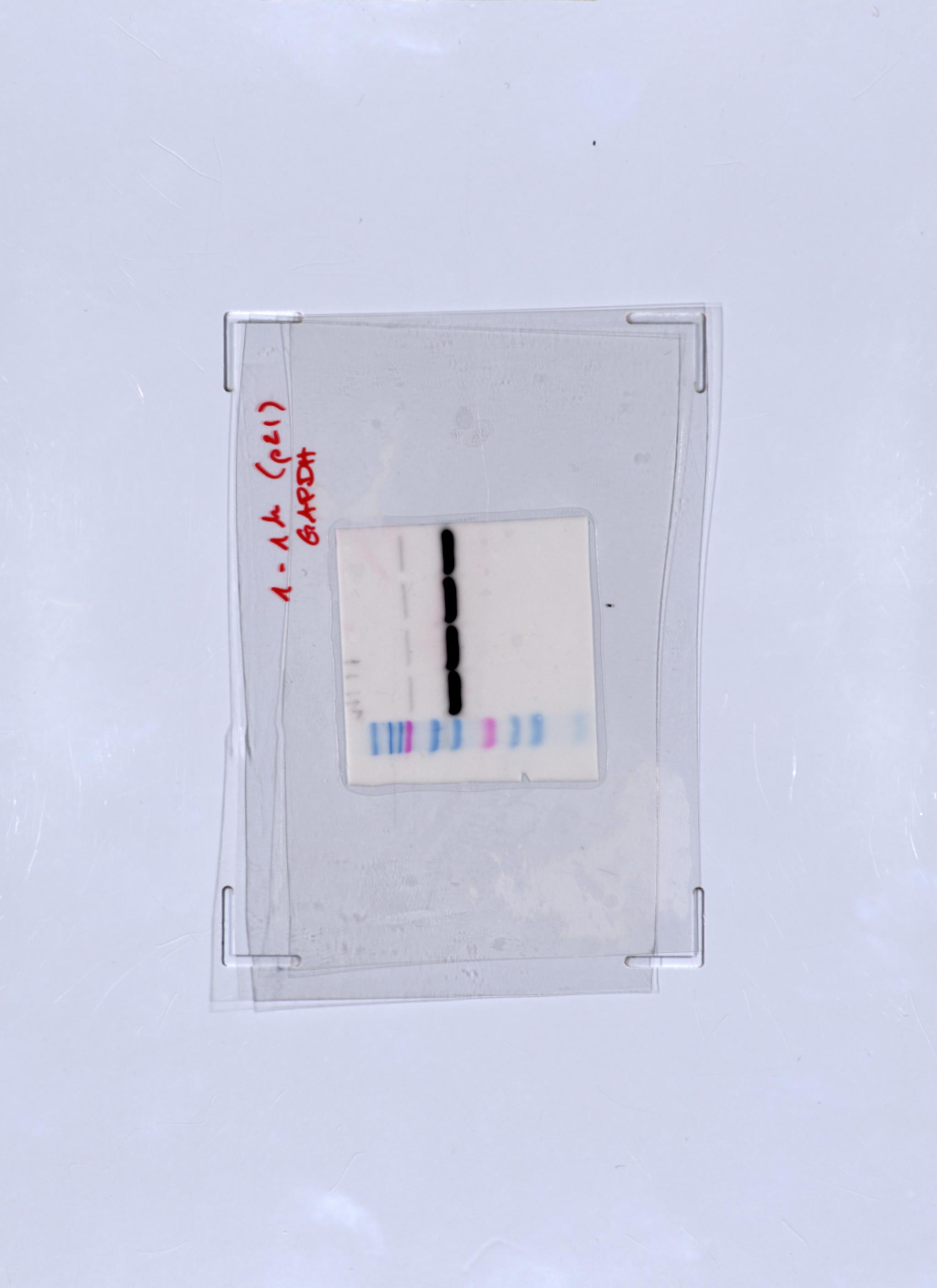

Supplement: Supplementary file 1 [file cancers-16-00370-s001.zip › BGJP_CPDM_1_1h_21GAP 2022.12.14_13.37.34_Ch+Marker.jpg]

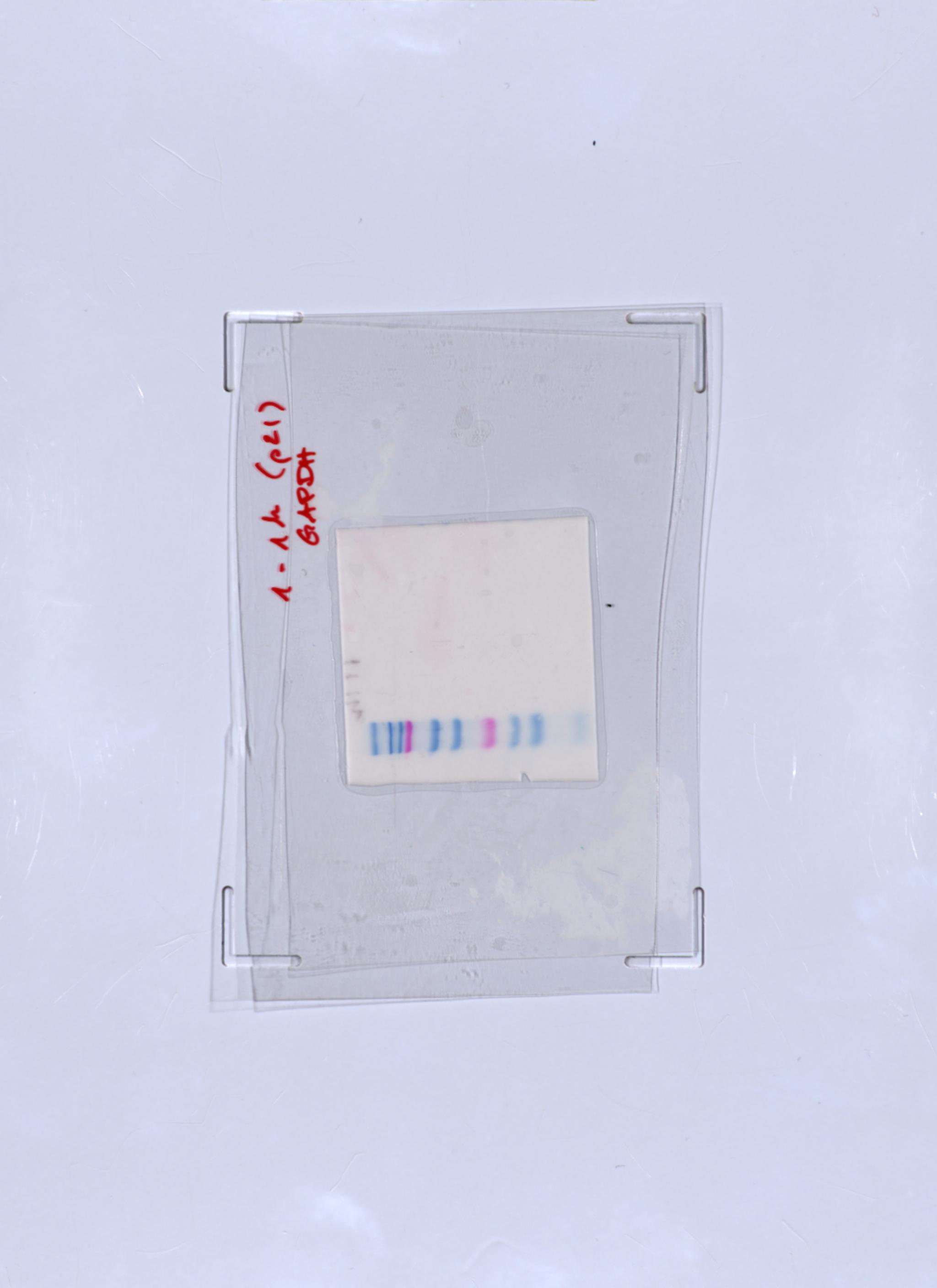

Supplement: Supplementary file 1 [file cancers-16-00370-s001.zip › BGJP_CPDM_1_1h_21GAP 2022.12.14_13.37.34_Ch-Marker.jpg]

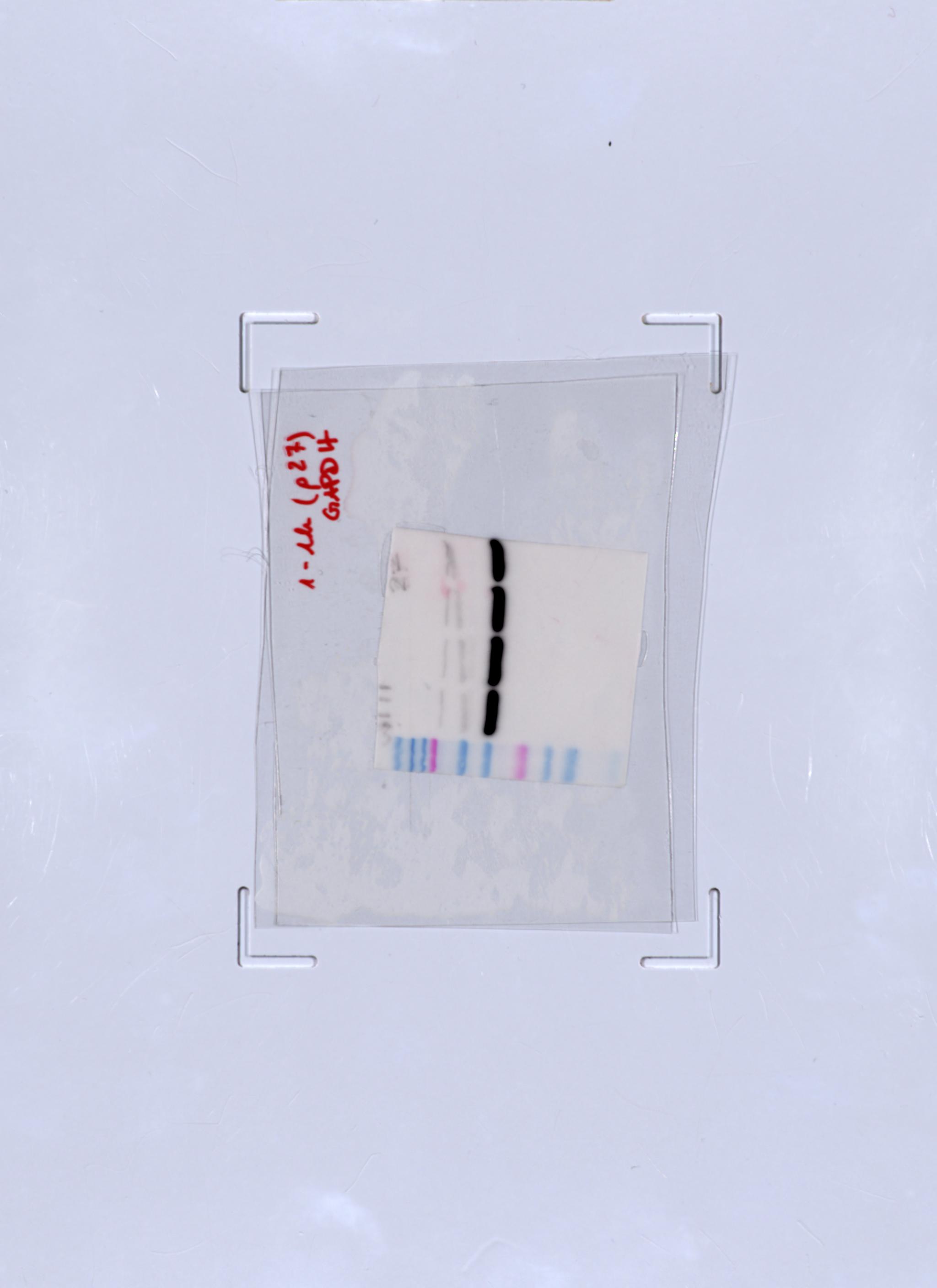

Supplement: Supplementary file 1 [file cancers-16-00370-s001.zip › BGJP_CPDM_1_1h_27GAP 2022.12.14_13.19.32_Ch+Marker.jpg]

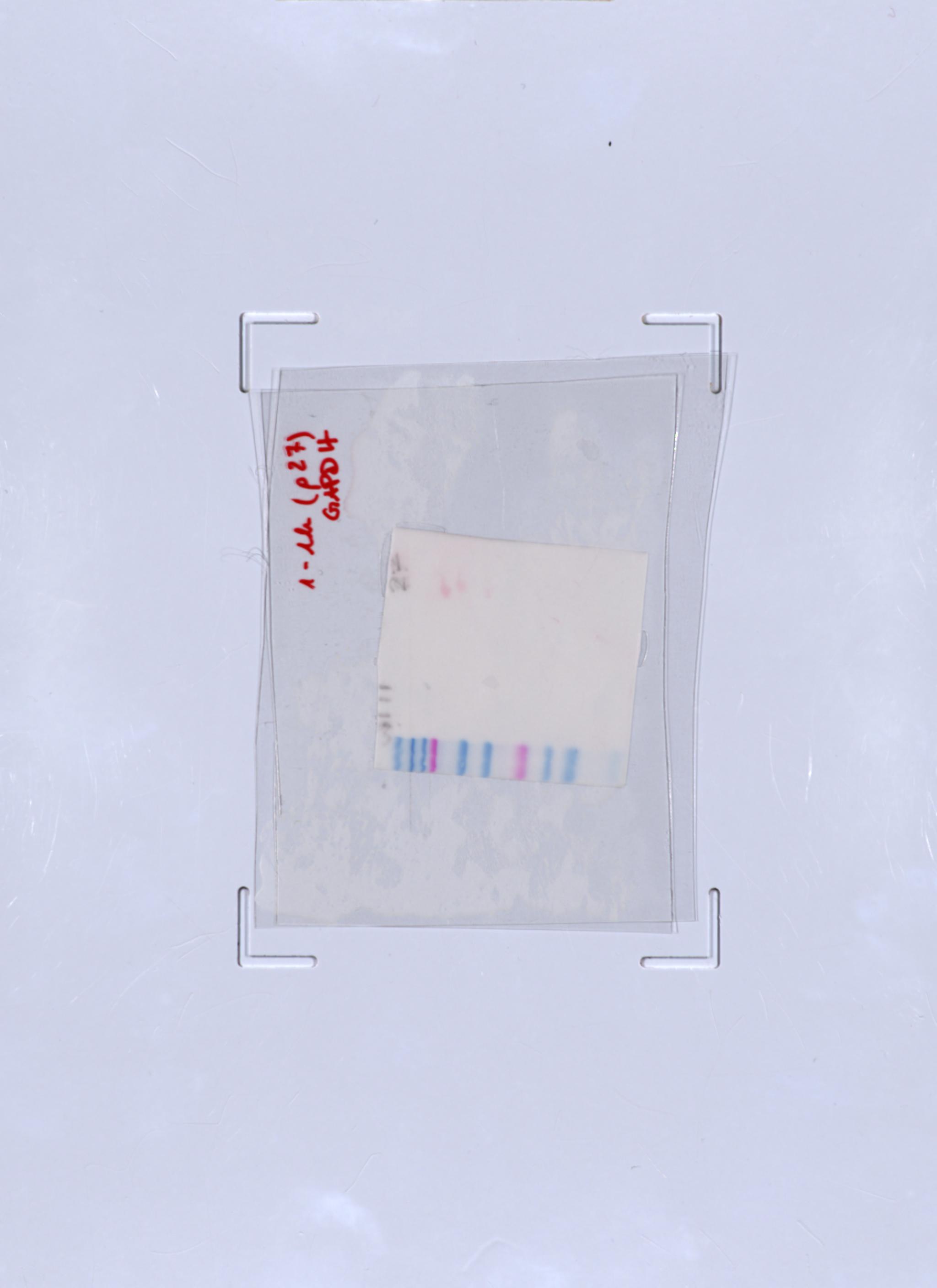

Supplement: Supplementary file 1 [file cancers-16-00370-s001.zip › BGJP_CPDM_1_1h_27GAP 2022.12.14_13.19.32_Ch-Marker.jpg]

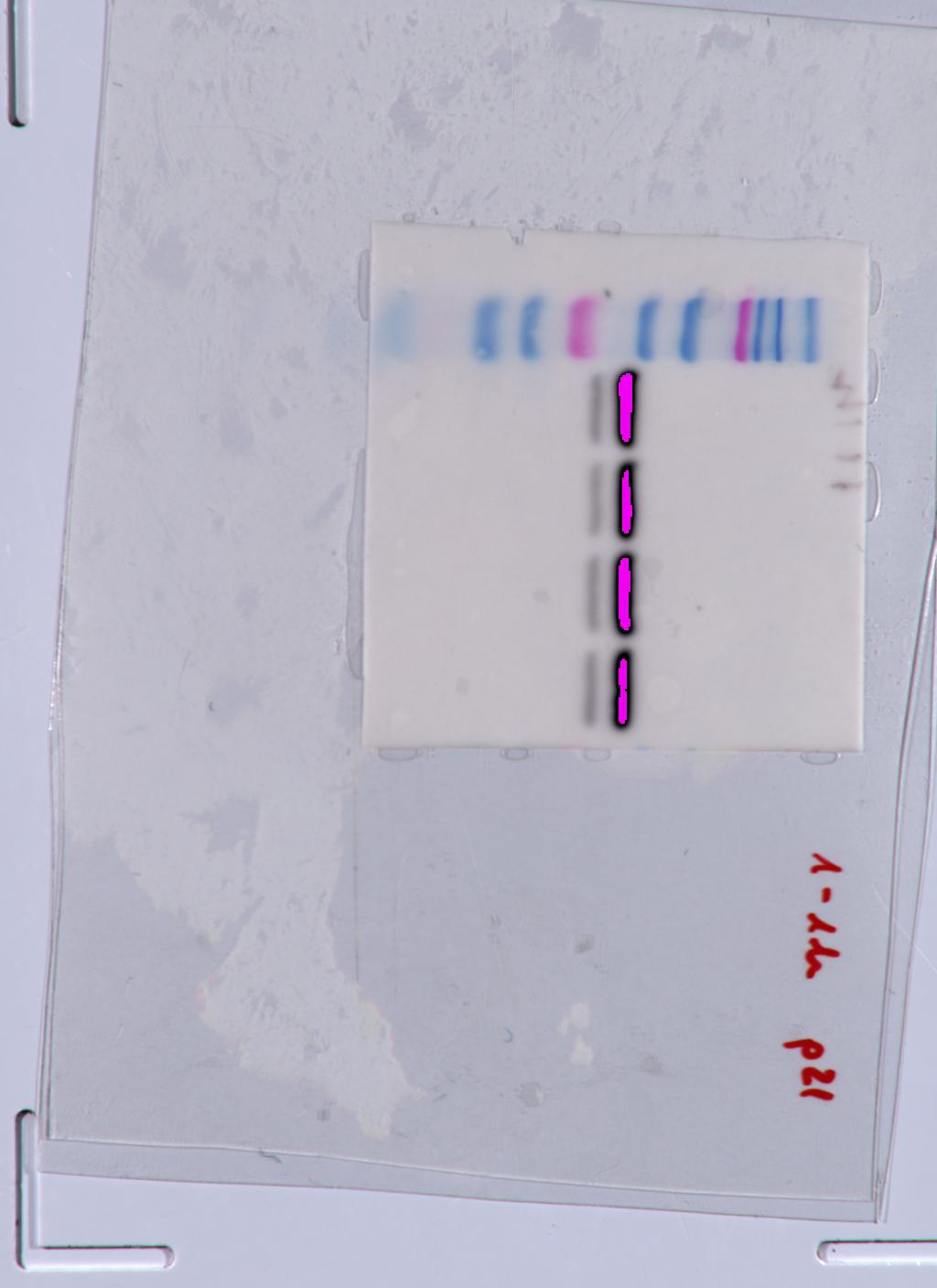

Supplement: Supplementary file 1 [file cancers-16-00370-s001.zip › BGJP_CPDM_1_1h_p21 2022.12.12_15.00.25_Ch+Marker.jpg]

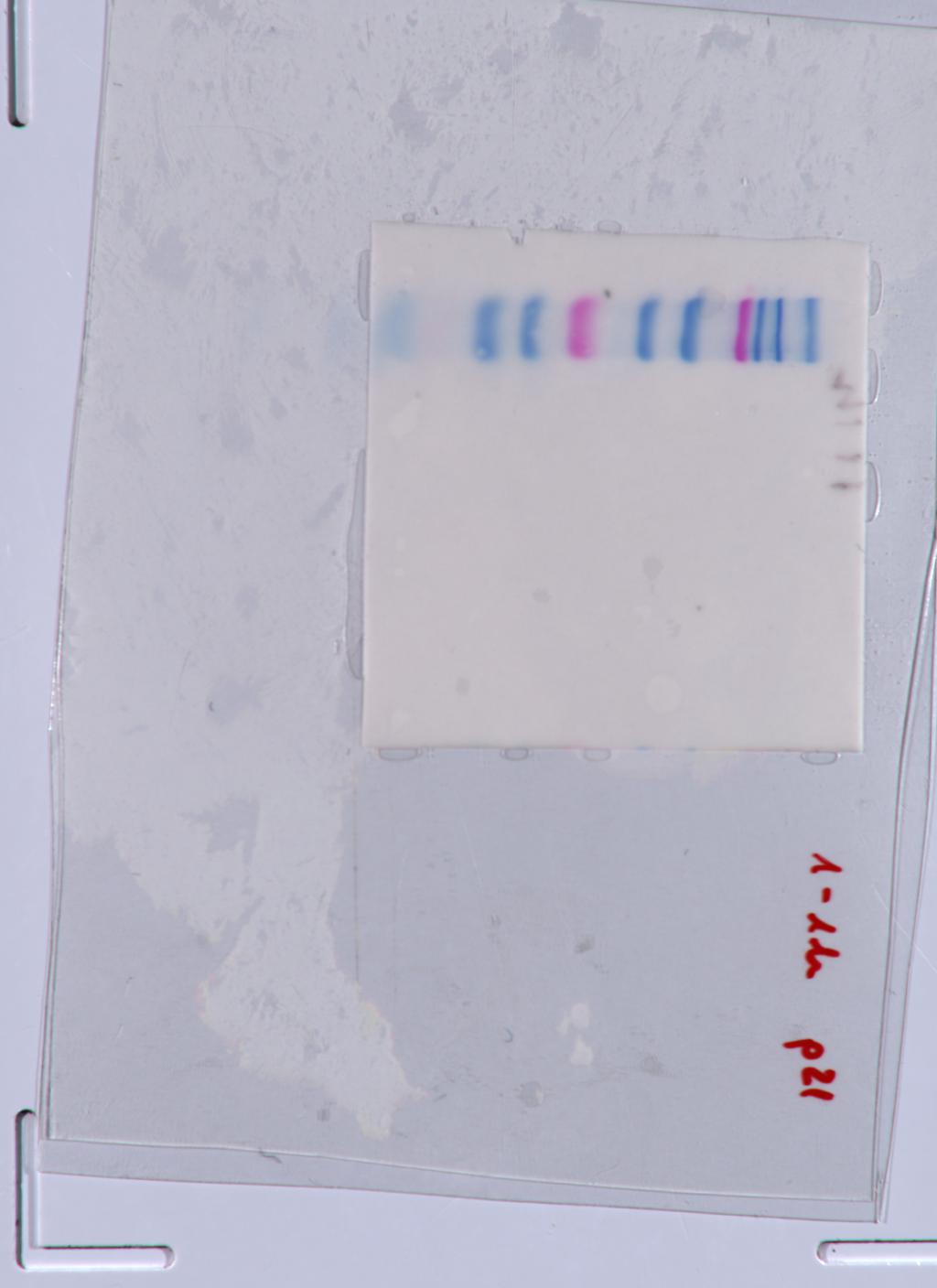

Supplement: Supplementary file 1 [file cancers-16-00370-s001.zip › BGJP_CPDM_1_1h_p21 2022.12.12_15.00.25_Ch-Marker.jpg]

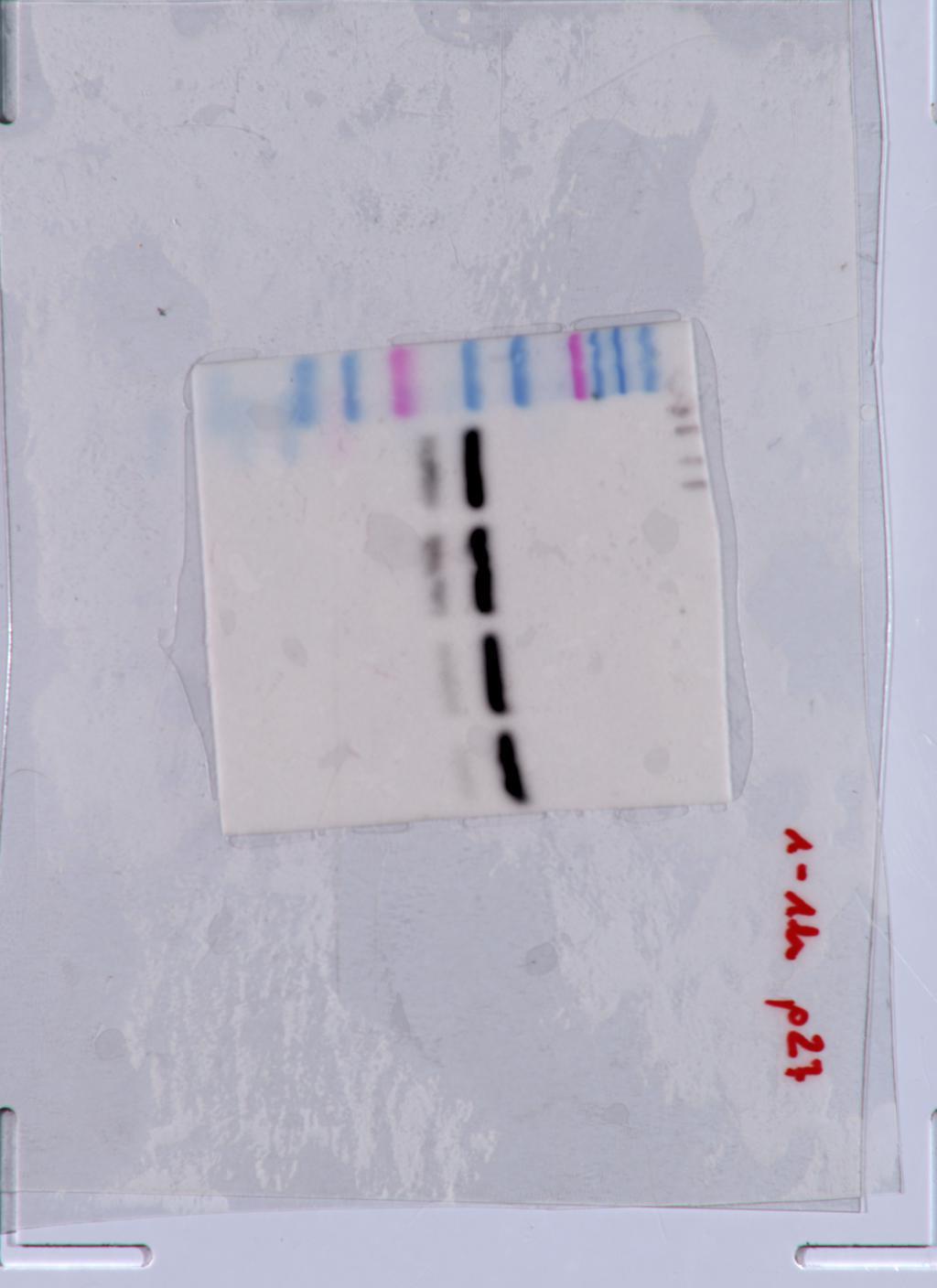

Supplement: Supplementary file 1 [file cancers-16-00370-s001.zip › BGJP_CPDM_1_1h_p27 2022.12.12_15.23.26_Ch+Marker.jpg]

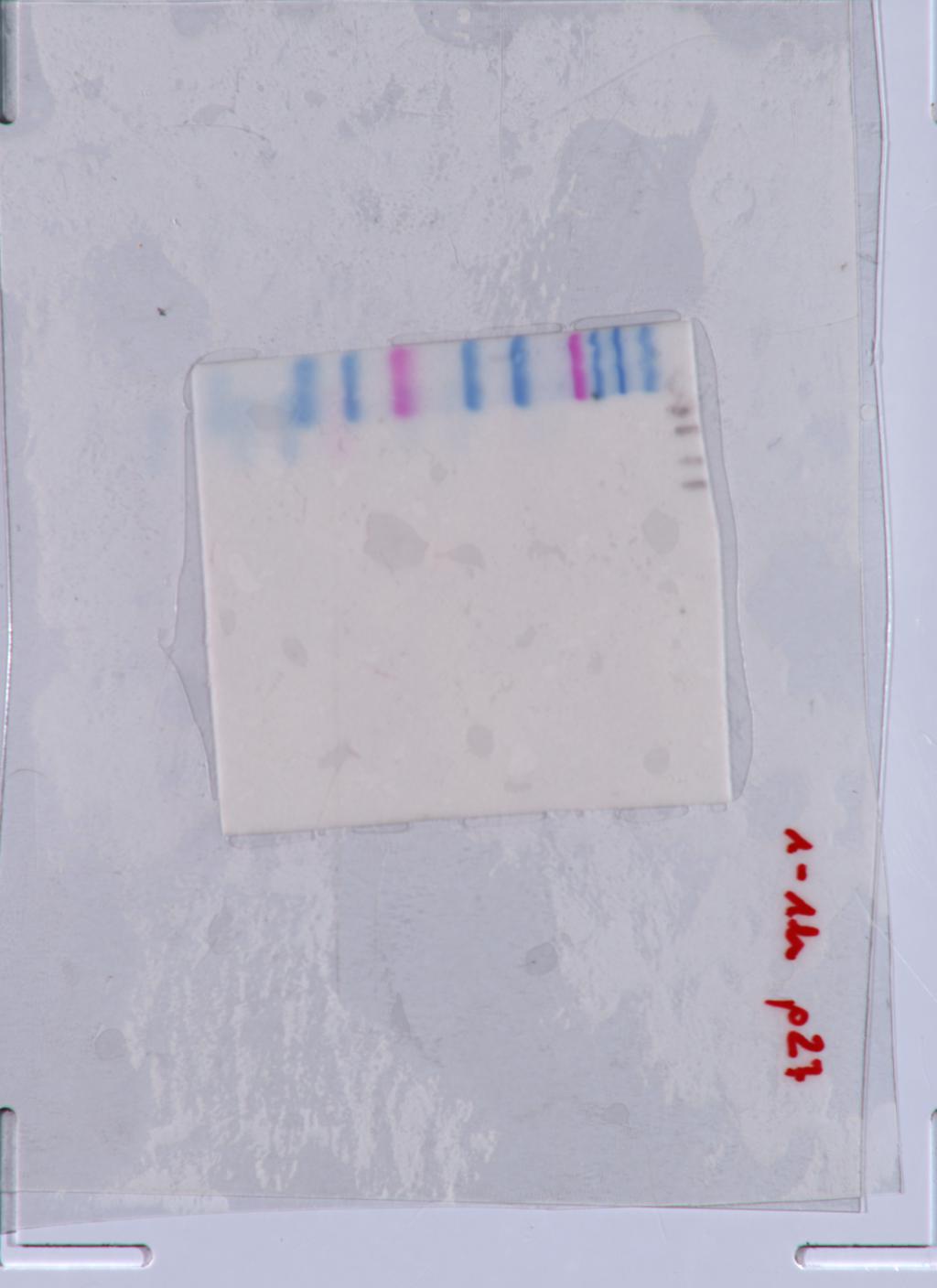

Supplement: Supplementary file 1 [file cancers-16-00370-s001.zip › BGJP_CPDM_1_1h_p27 2022.12.12_15.23.26_Ch-Marker.jpg]

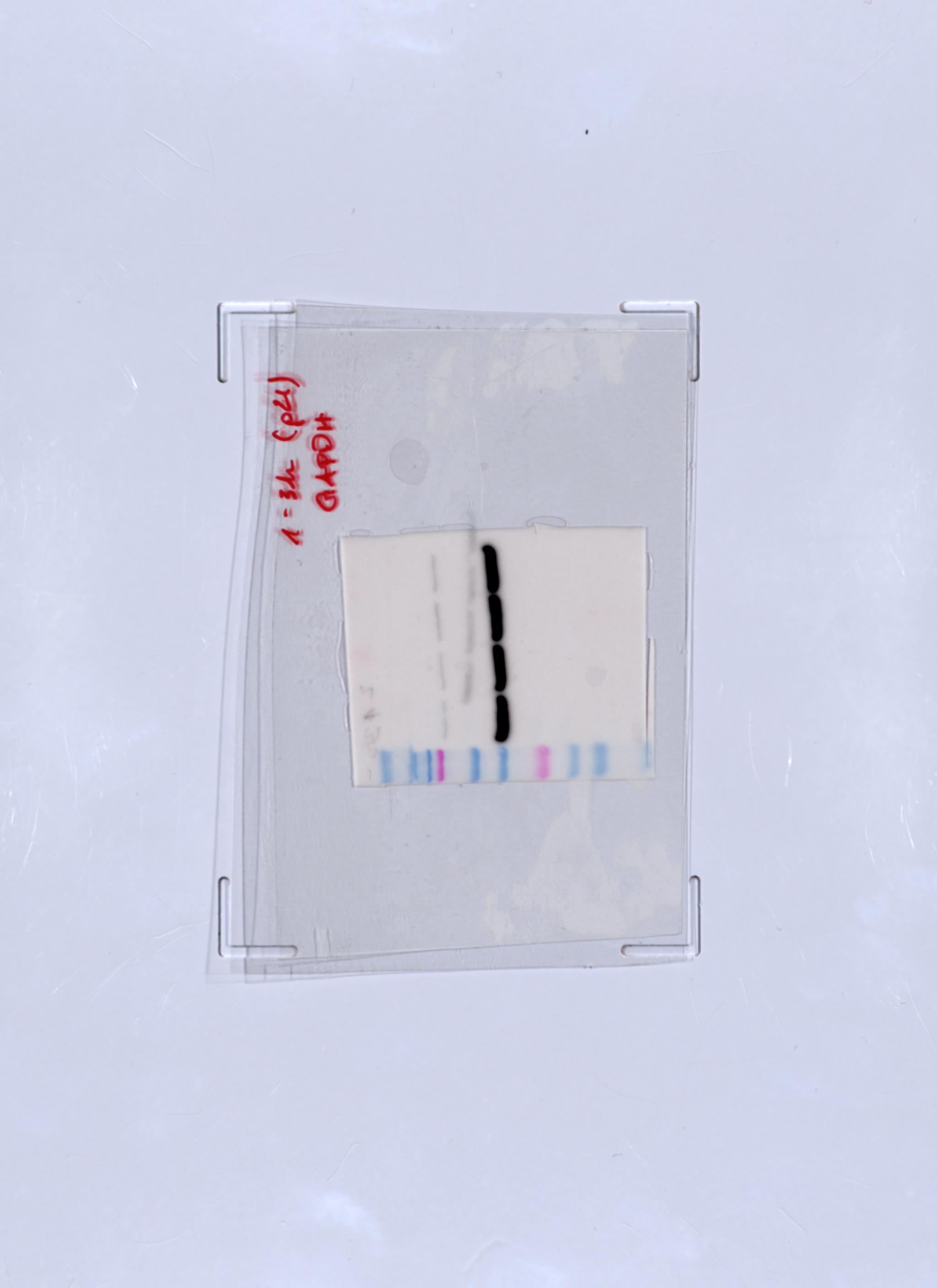

Supplement: Supplementary file 1 [file cancers-16-00370-s001.zip › BGJP_CPDM_1_3h_21GAP 2022.12.14_13.54.32_Ch+Marker.jpg]

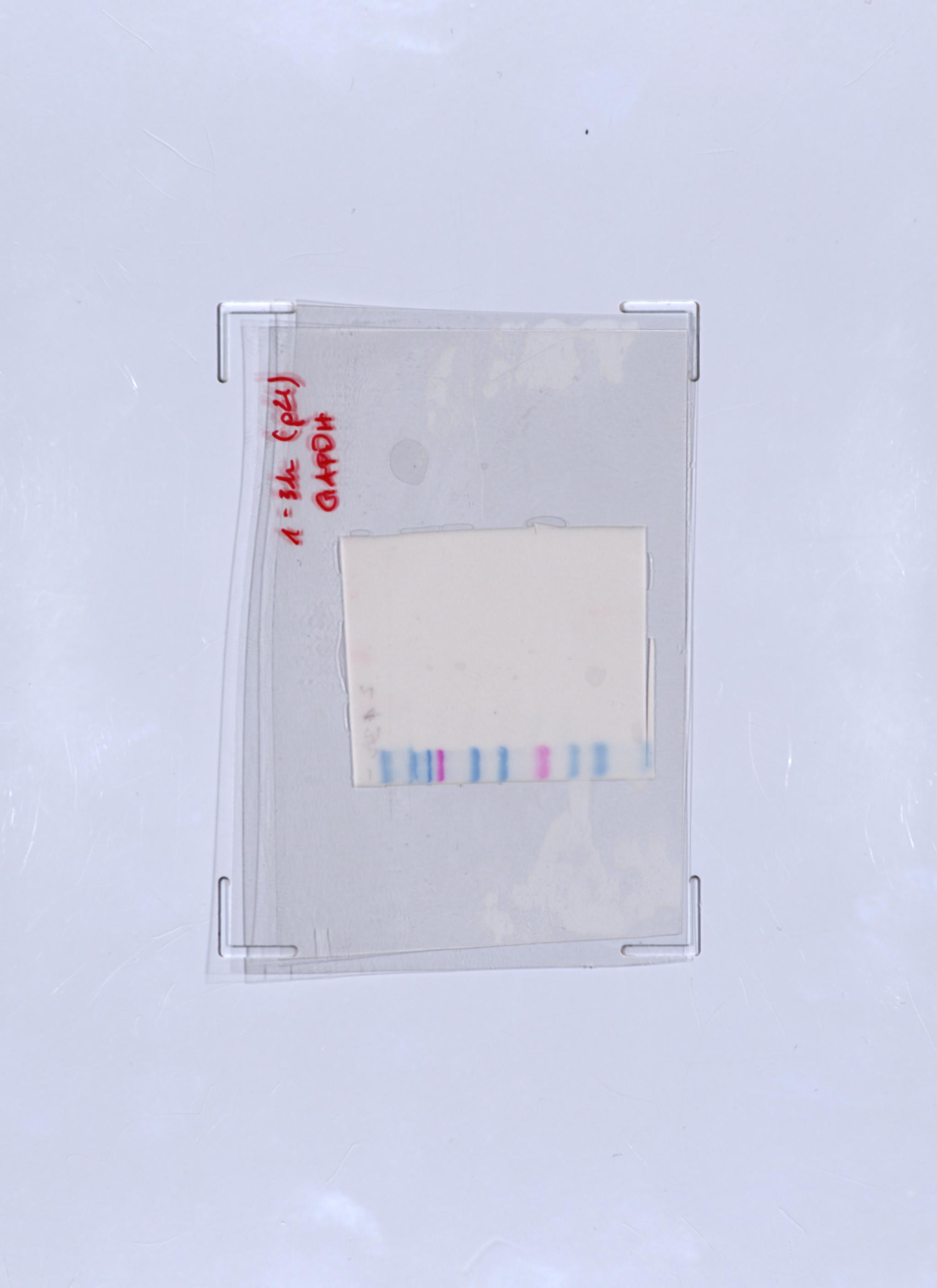

Supplement: Supplementary file 1 [file cancers-16-00370-s001.zip › BGJP_CPDM_1_3h_21GAP 2022.12.14_13.54.32_Ch-Marker.jpg]

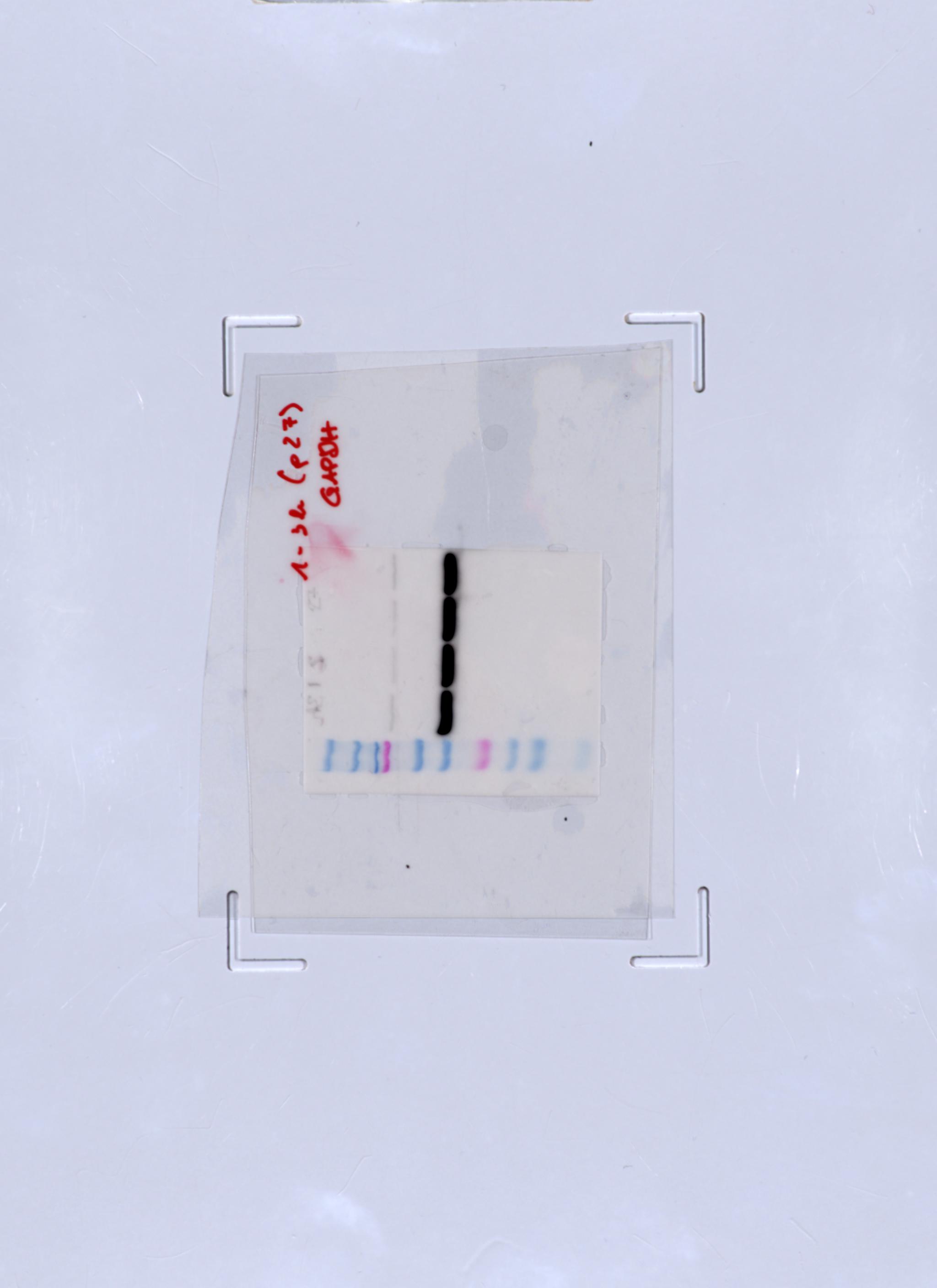

Supplement: Supplementary file 1 [file cancers-16-00370-s001.zip › BGJP_CPDM_1_3h_27GAP 2022.12.14_13.04.44_Ch+Marker.jpg]

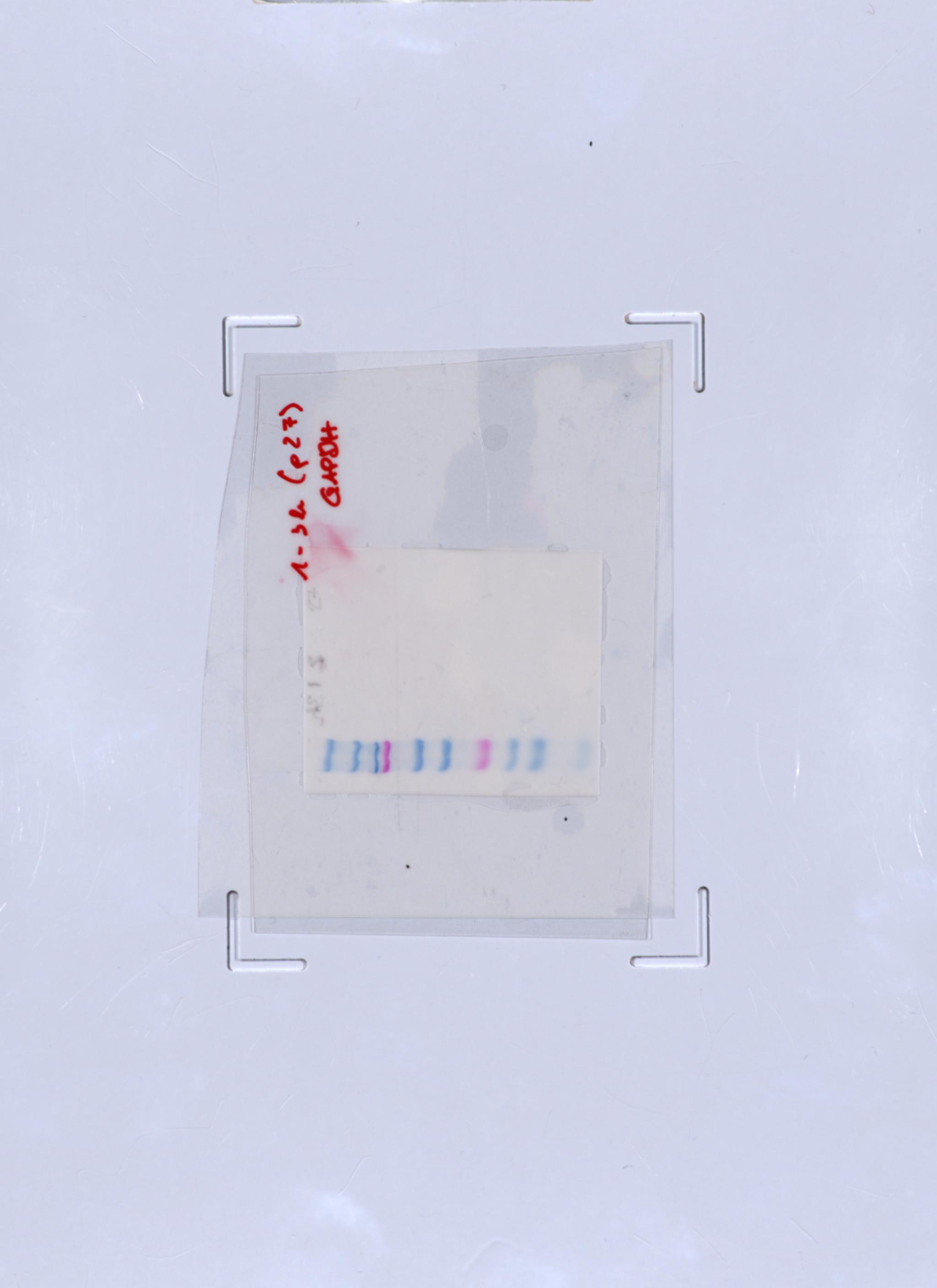

Supplement: Supplementary file 1 [file cancers-16-00370-s001.zip › BGJP_CPDM_1_3h_27GAP 2022.12.14_13.04.44_Ch-Marker.jpg]

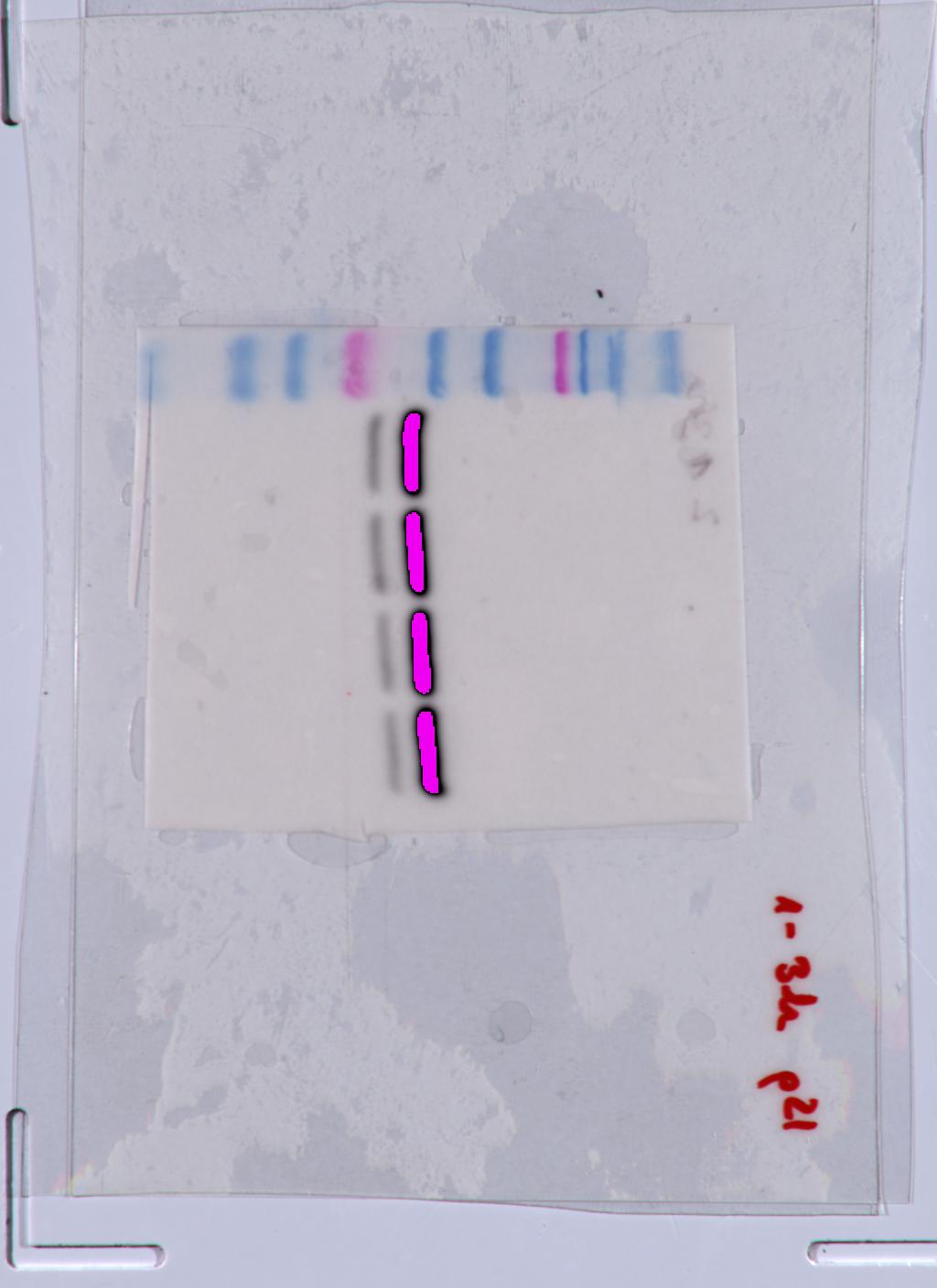

Supplement: Supplementary file 1 [file cancers-16-00370-s001.zip › BGJP_CPDM_1_3h_p21 2022.12.12_15.12.02_Ch+Marker.jpg]

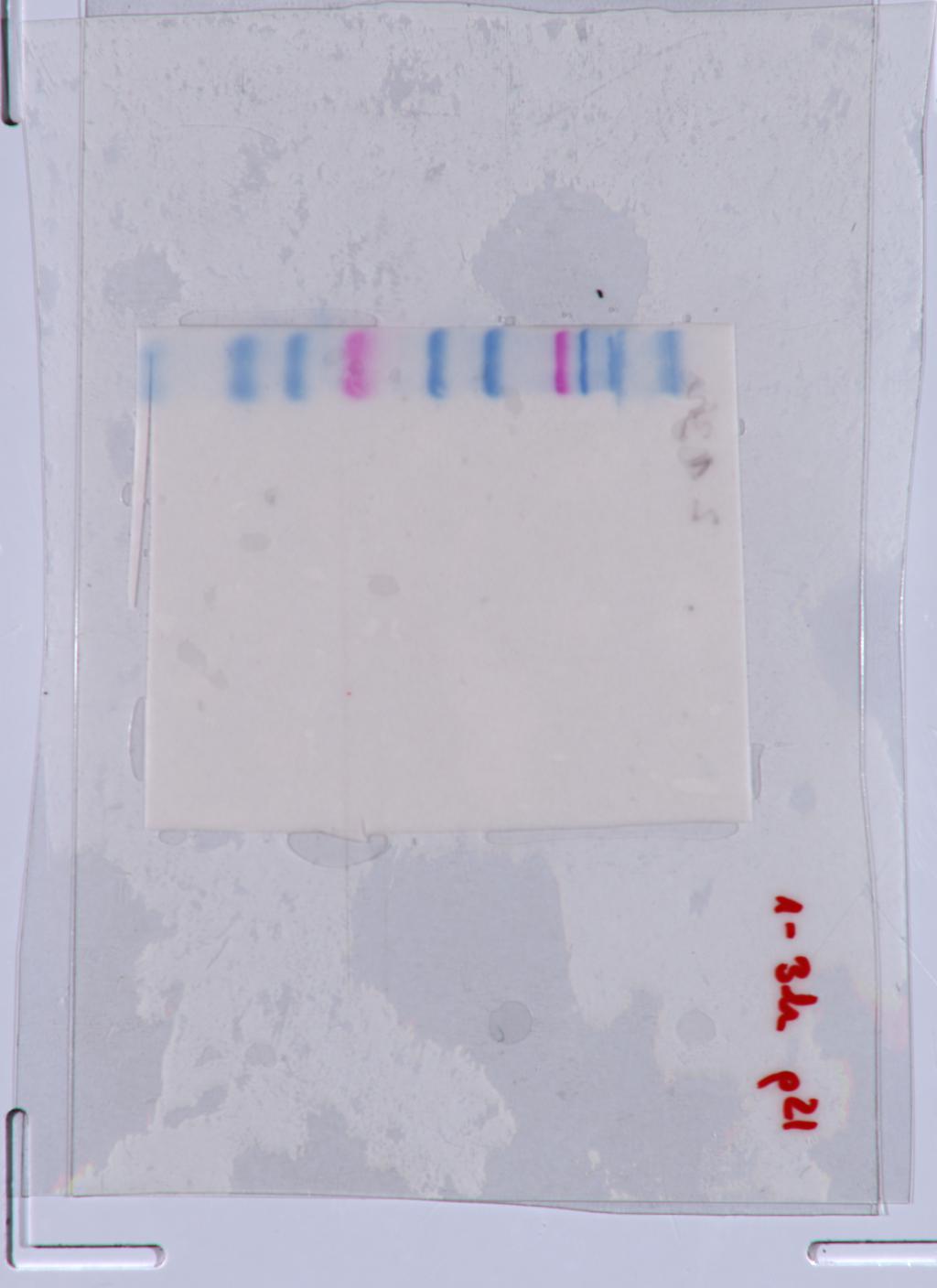

Supplement: Supplementary file 1 [file cancers-16-00370-s001.zip › BGJP_CPDM_1_3h_p21 2022.12.12_15.12.02_Ch-Marker.jpg]

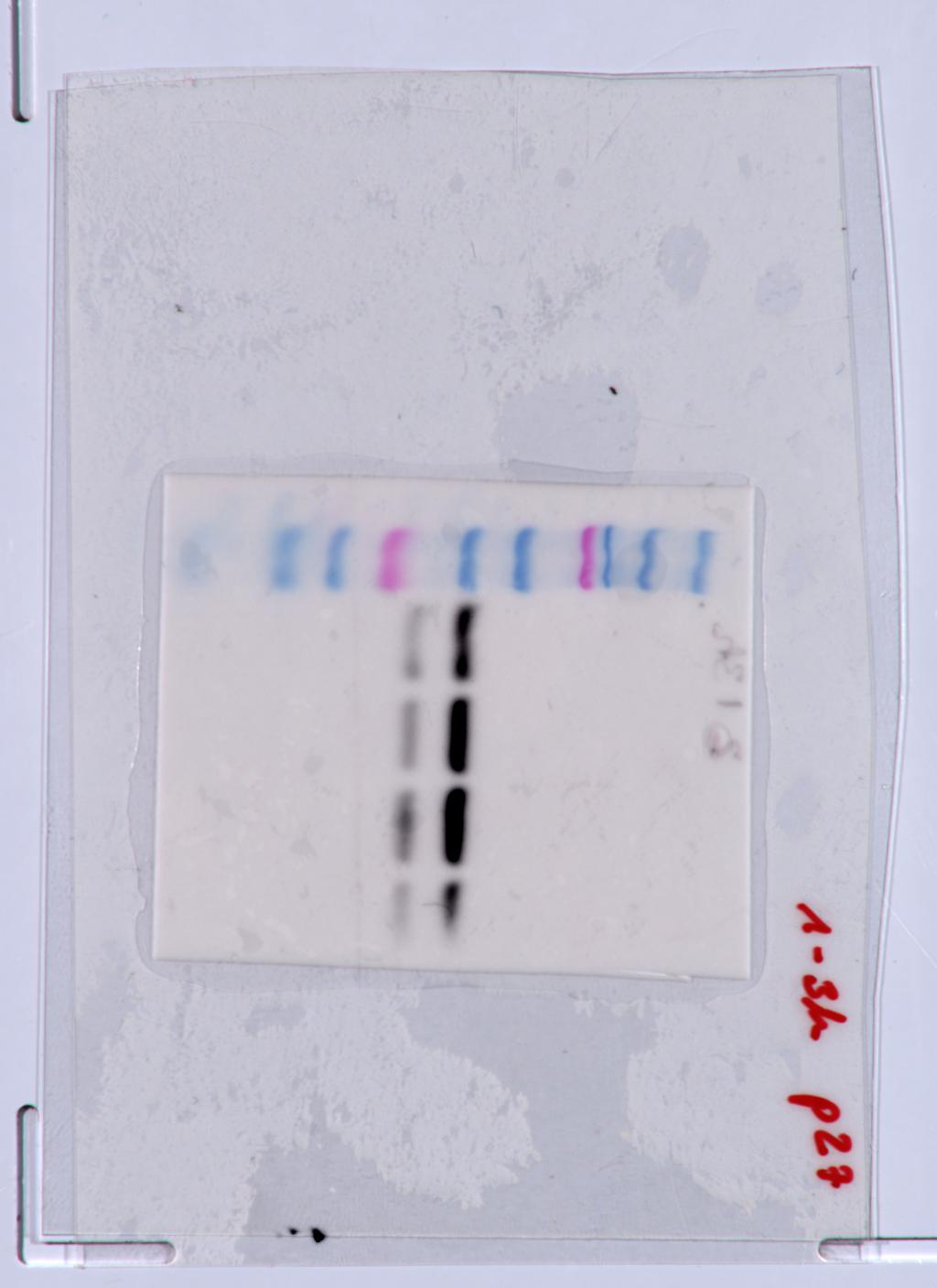

Supplement: Supplementary file 1 [file cancers-16-00370-s001.zip › BGJP_CPDM_1_3h_p27 2022.12.12_15.38.41_Ch+Marker.jpg]

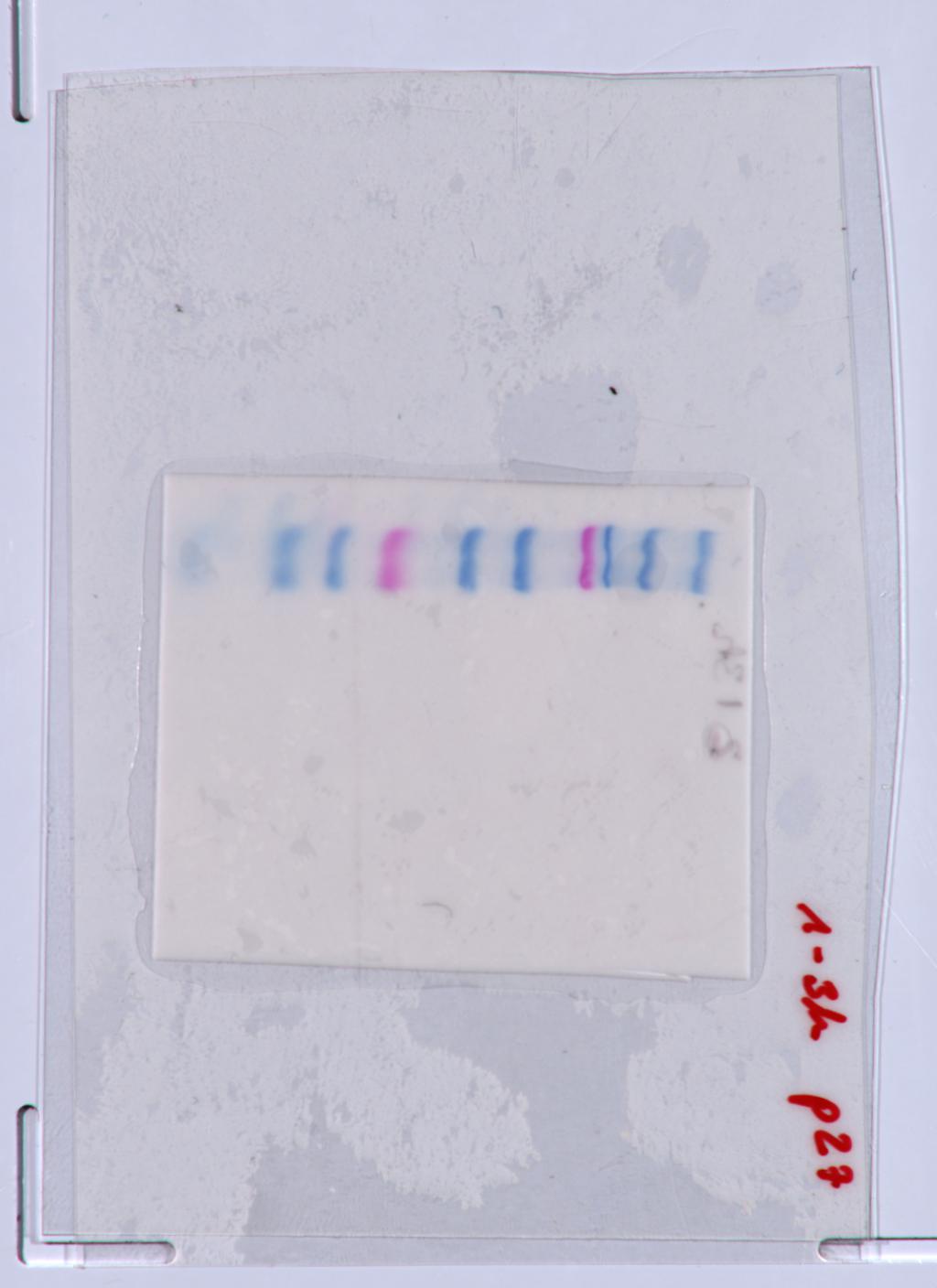

Supplement: Supplementary file 1 [file cancers-16-00370-s001.zip › BGJP_CPDM_1_3h_p27 2022.12.12_15.38.41_Ch-Marker.jpg]

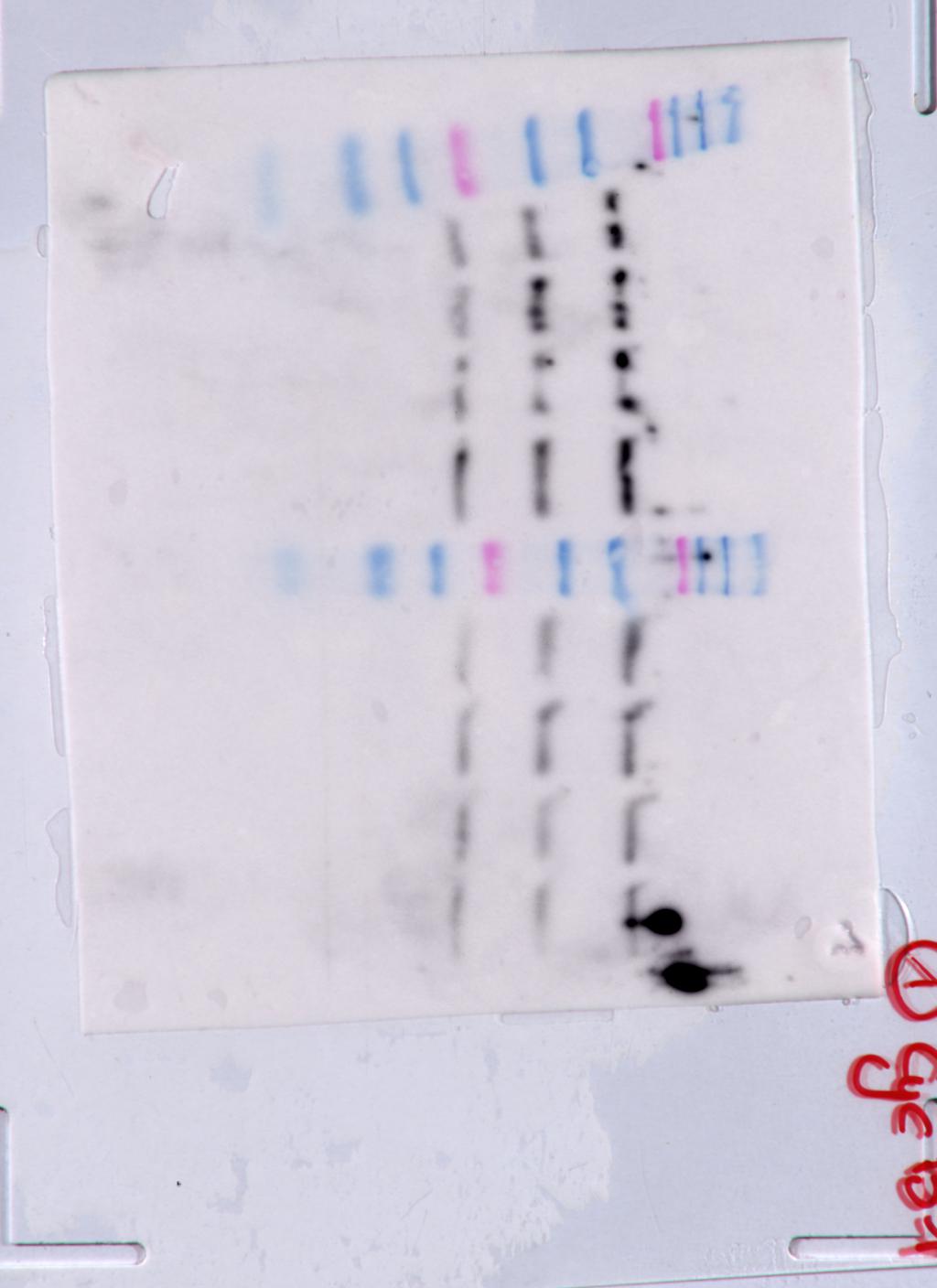

Supplement: Supplementary file 1 [file cancers-16-00370-s001.zip › BGJP_CPDM_1_CycB1 2022.12.18_12.20.39_Ch+Marker.jpg]

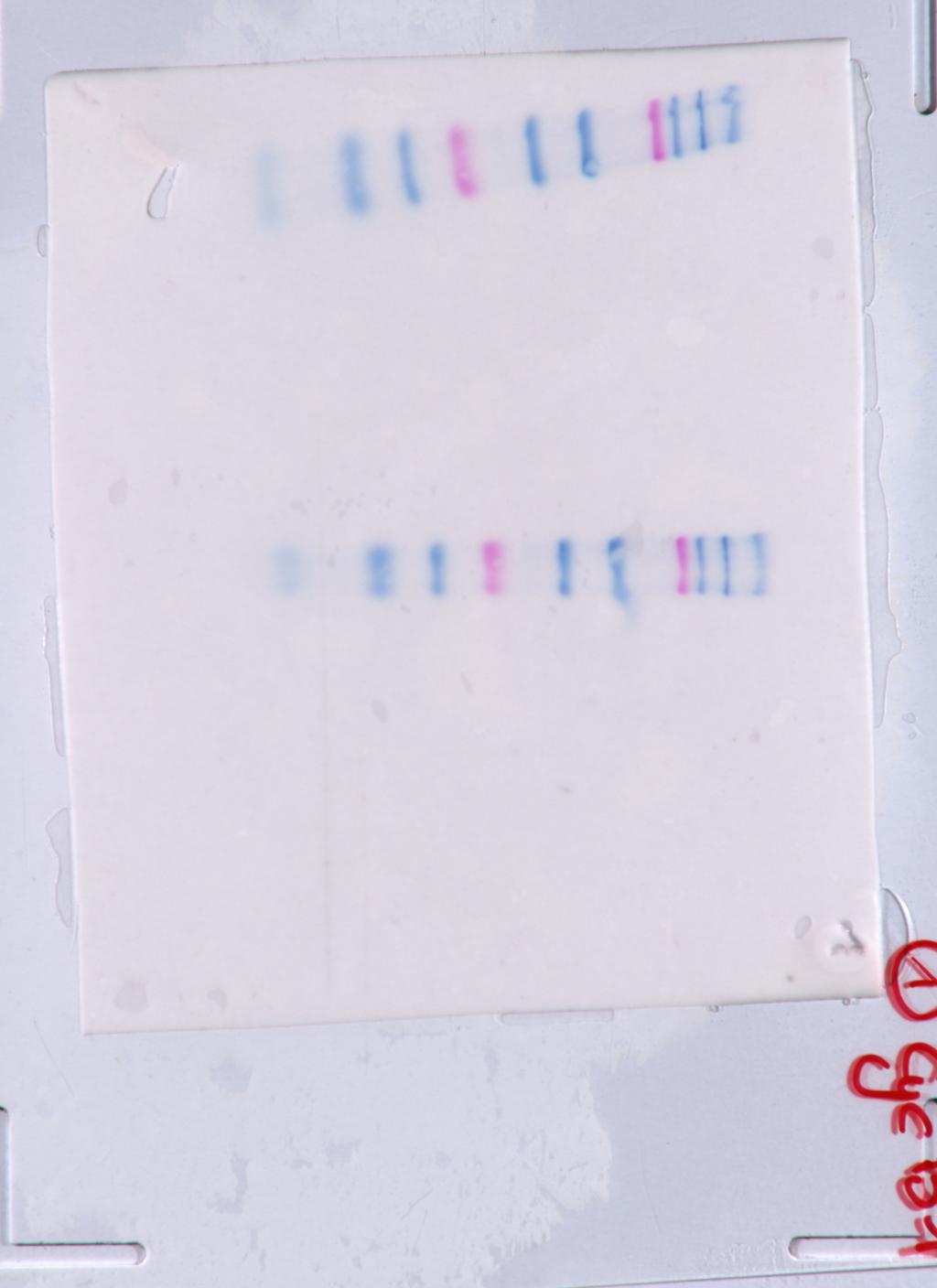

Supplement: Supplementary file 1 [file cancers-16-00370-s001.zip › BGJP_CPDM_1_CycB1 2022.12.18_12.20.39_Ch-Marker.jpg]

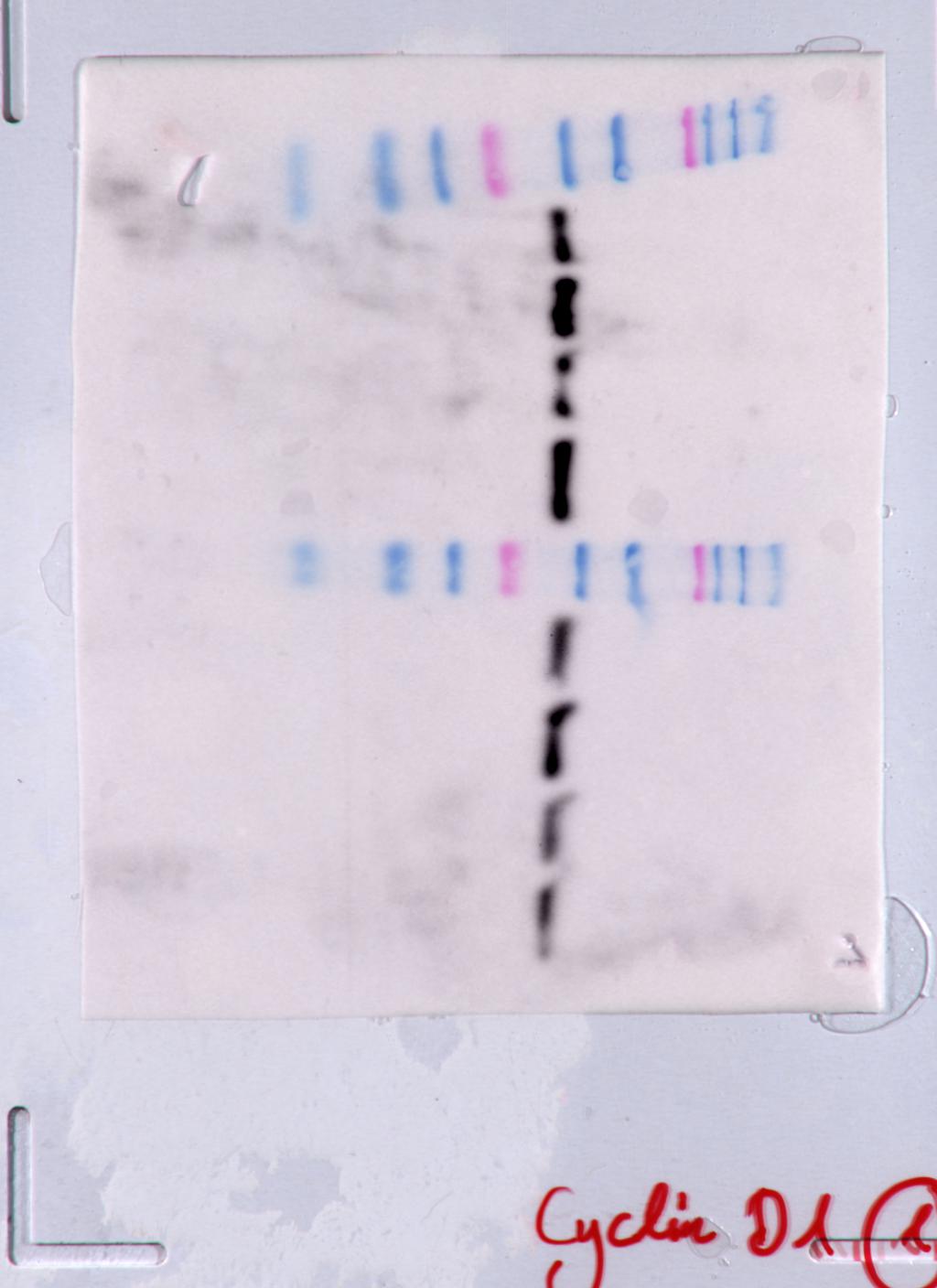

Supplement: Supplementary file 1 [file cancers-16-00370-s001.zip › BGJP_CPDM_1_CycD1 2022.12.17_14.17.58_Ch+Marker.jpg]

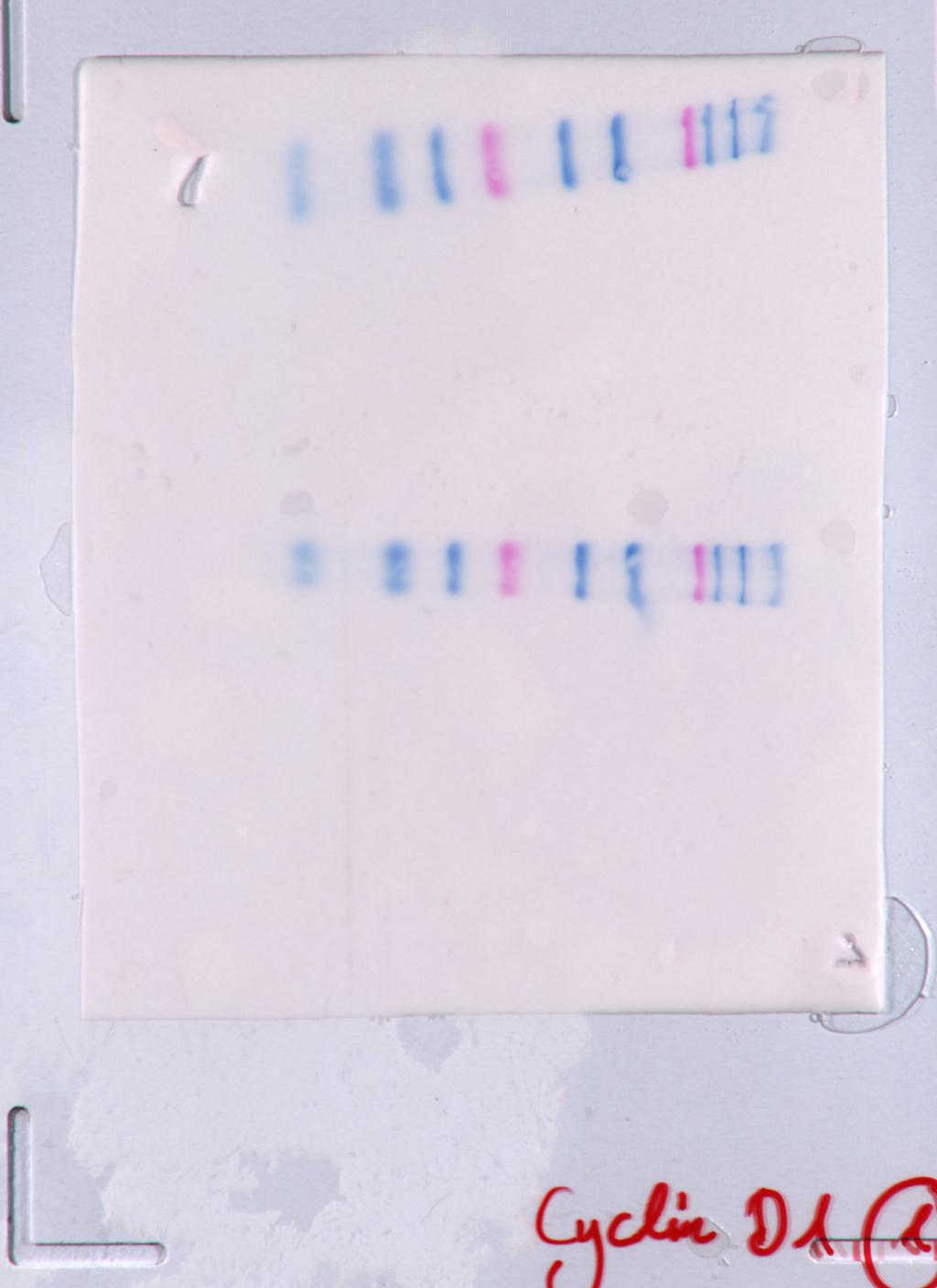

Supplement: Supplementary file 1 [file cancers-16-00370-s001.zip › BGJP_CPDM_1_CycD1 2022.12.17_14.17.58_Ch-Marker.jpg]

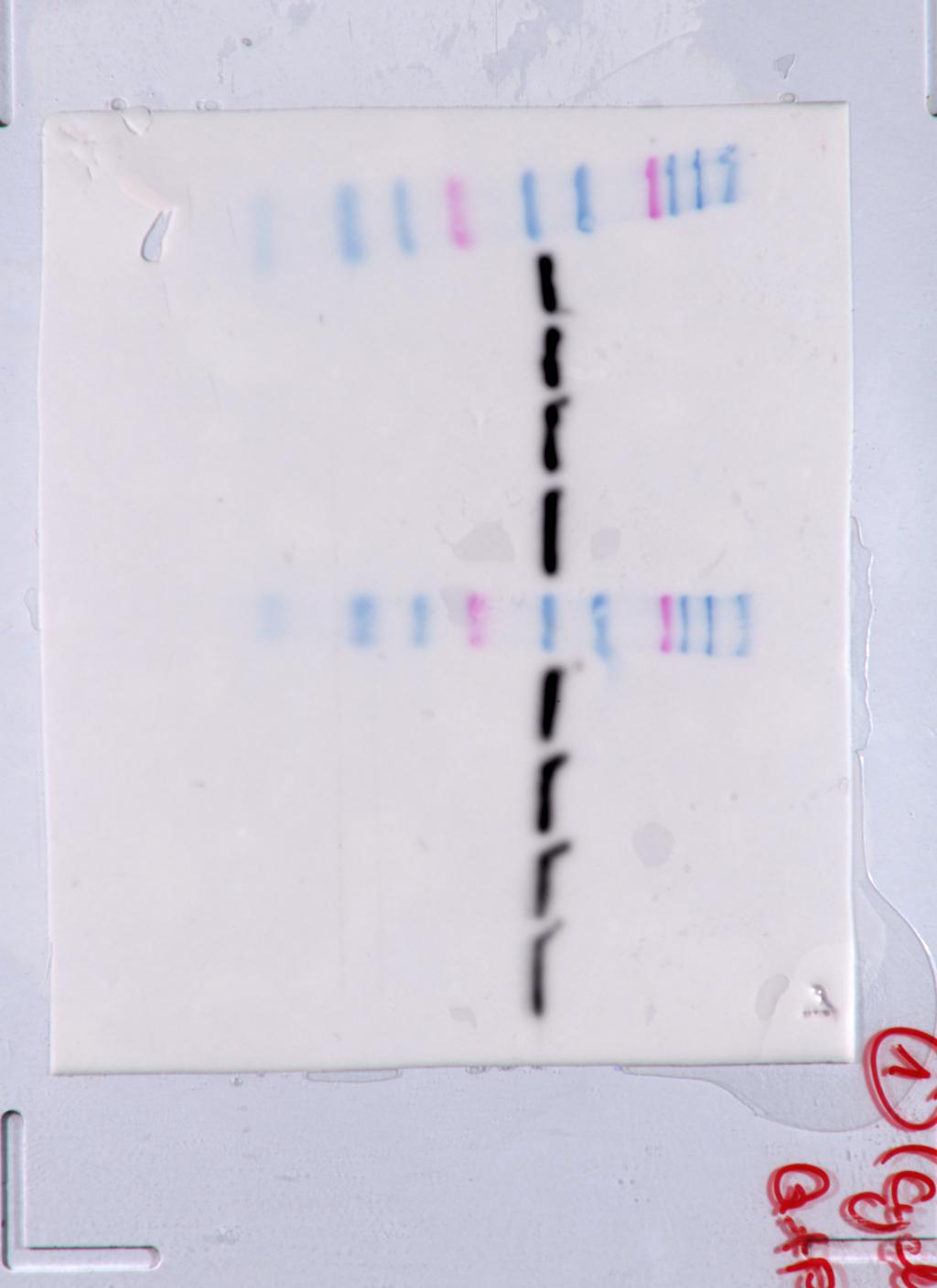

Supplement: Supplementary file 1 [file cancers-16-00370-s001.zip › BGJP_CPDM_1_Cycl_GAP 2022.12.19_14.33.53_Ch+Marker.jpg]

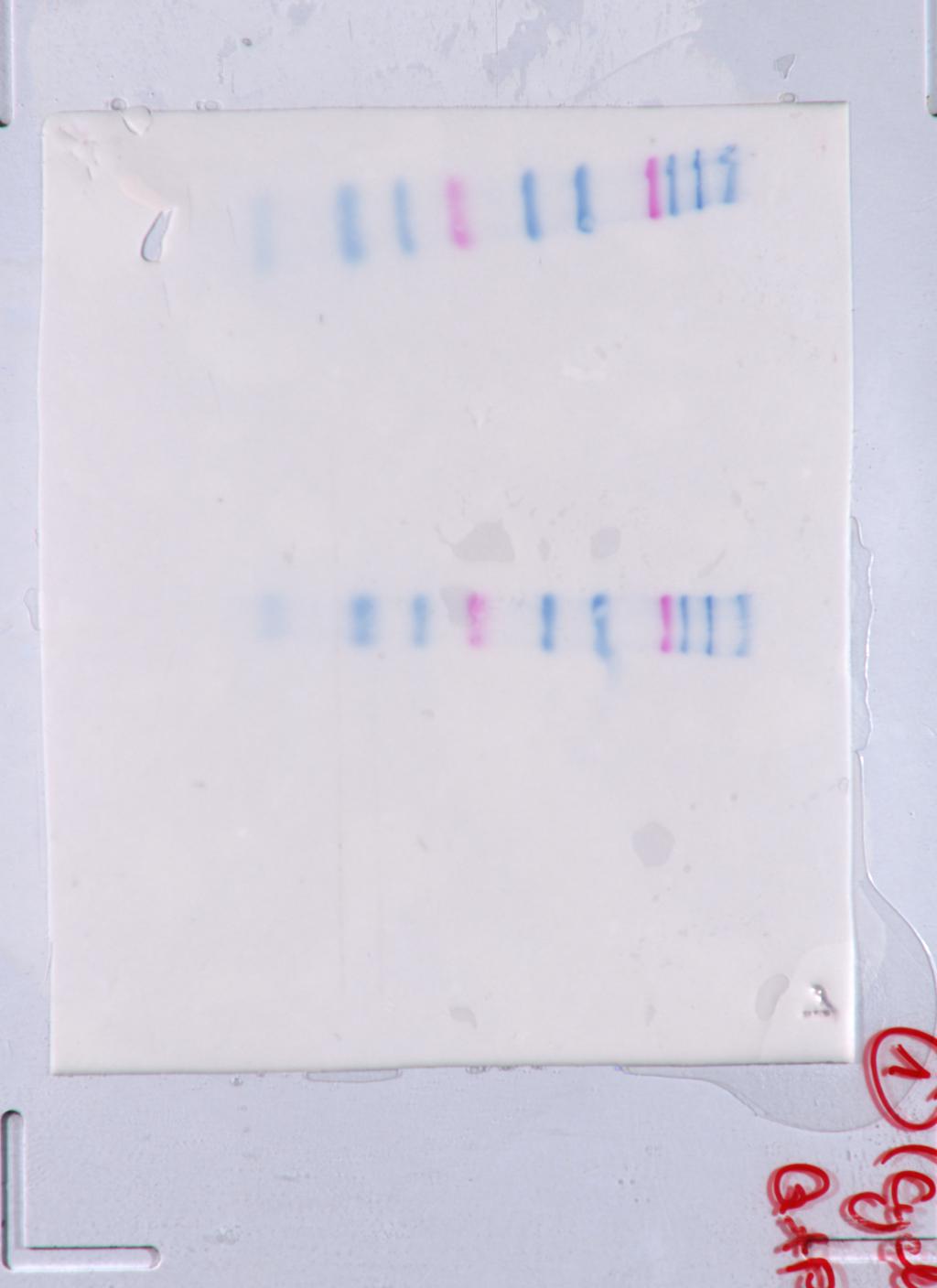

Supplement: Supplementary file 1 [file cancers-16-00370-s001.zip › BGJP_CPDM_1_Cycl_GAP 2022.12.19_14.33.53_Ch-Marker.jpg]

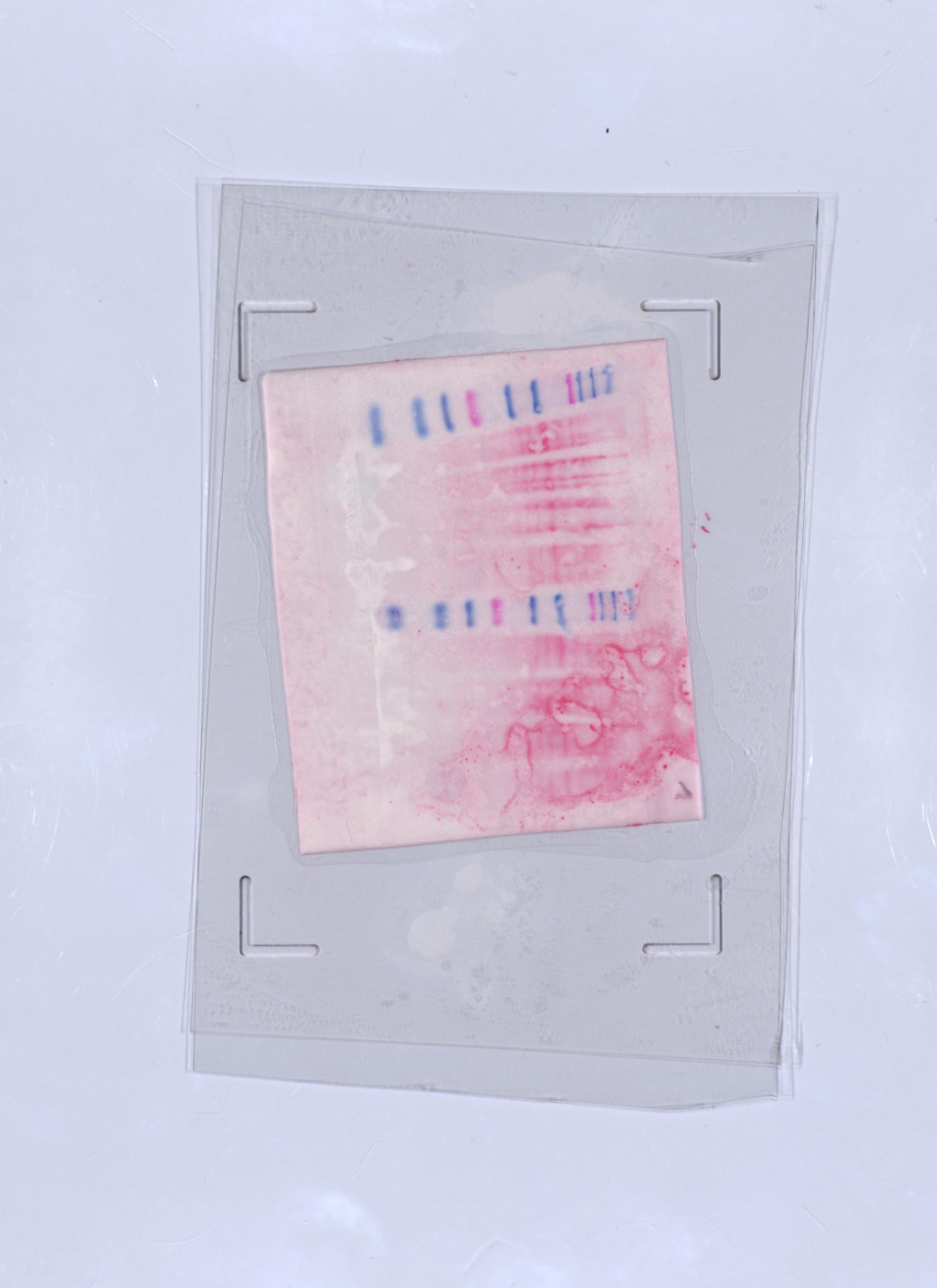

Supplement: Supplementary file 1 [file cancers-16-00370-s001.zip › BGJP_CPDM_1_G3_Ponc 2022.12.14_12.51.03_Co.jpg]

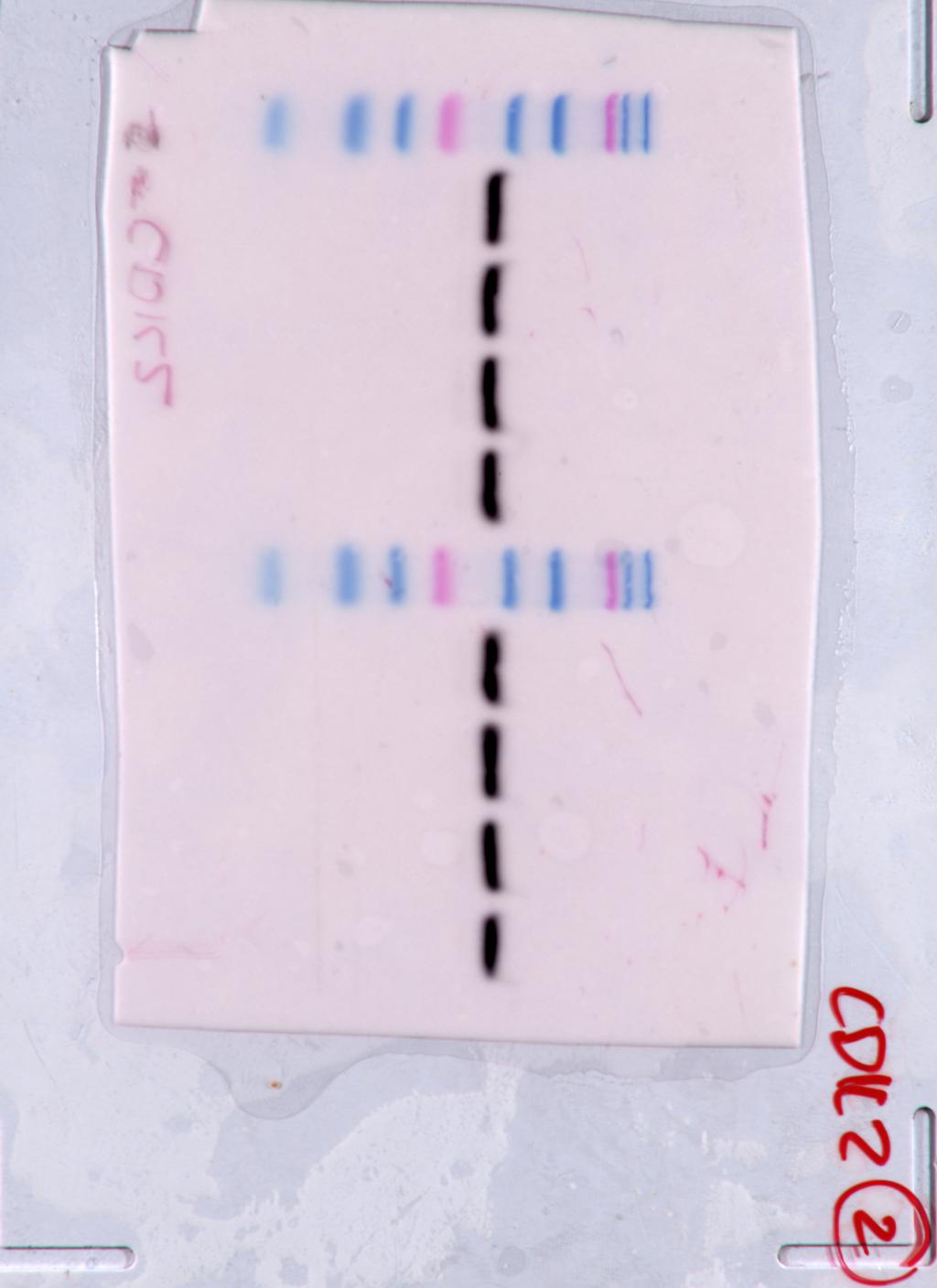

Supplement: Supplementary file 1 [file cancers-16-00370-s001.zip › BGJP_CPDM_2_CDK2 2022.12.17_14.44.15_Ch+Marker.jpg]

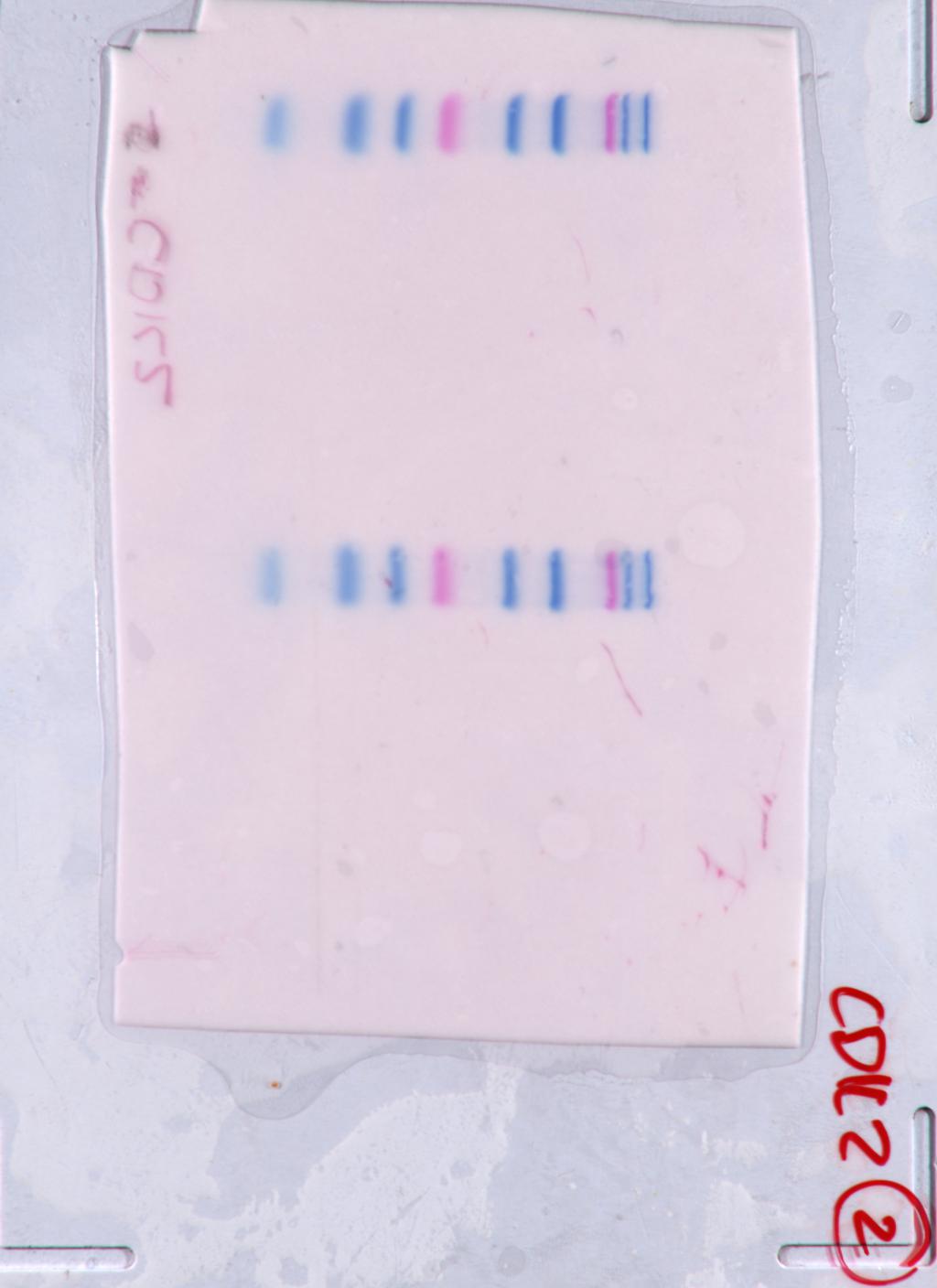

Supplement: Supplementary file 1 [file cancers-16-00370-s001.zip › BGJP_CPDM_2_CDK2 2022.12.17_14.44.15_Ch-Marker.jpg]

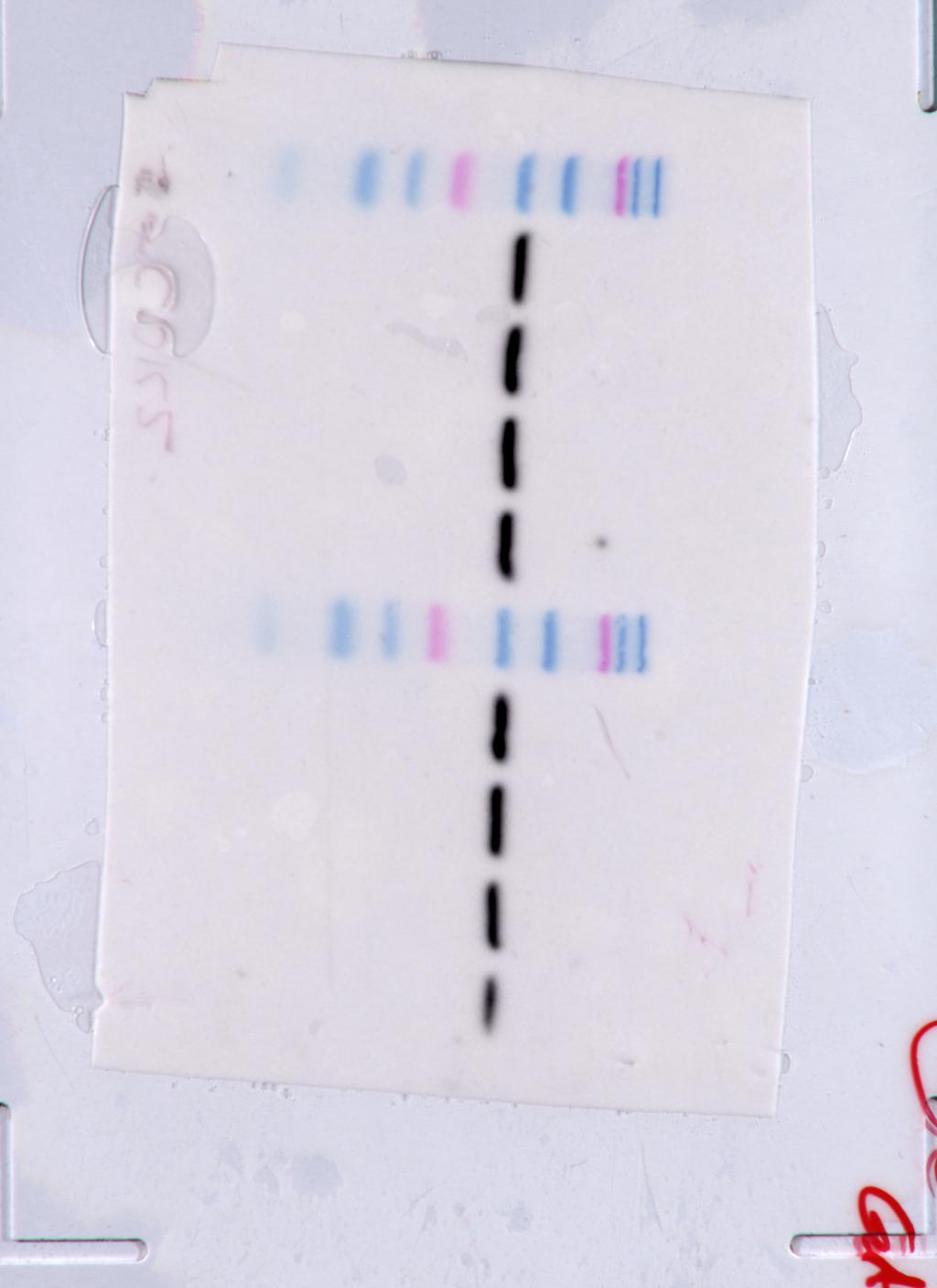

Supplement: Supplementary file 1 [file cancers-16-00370-s001.zip › BGJP_CPDM_2_CDK2_GAP 2022.12.19_13.56.18_Ch+Marker.jpg]

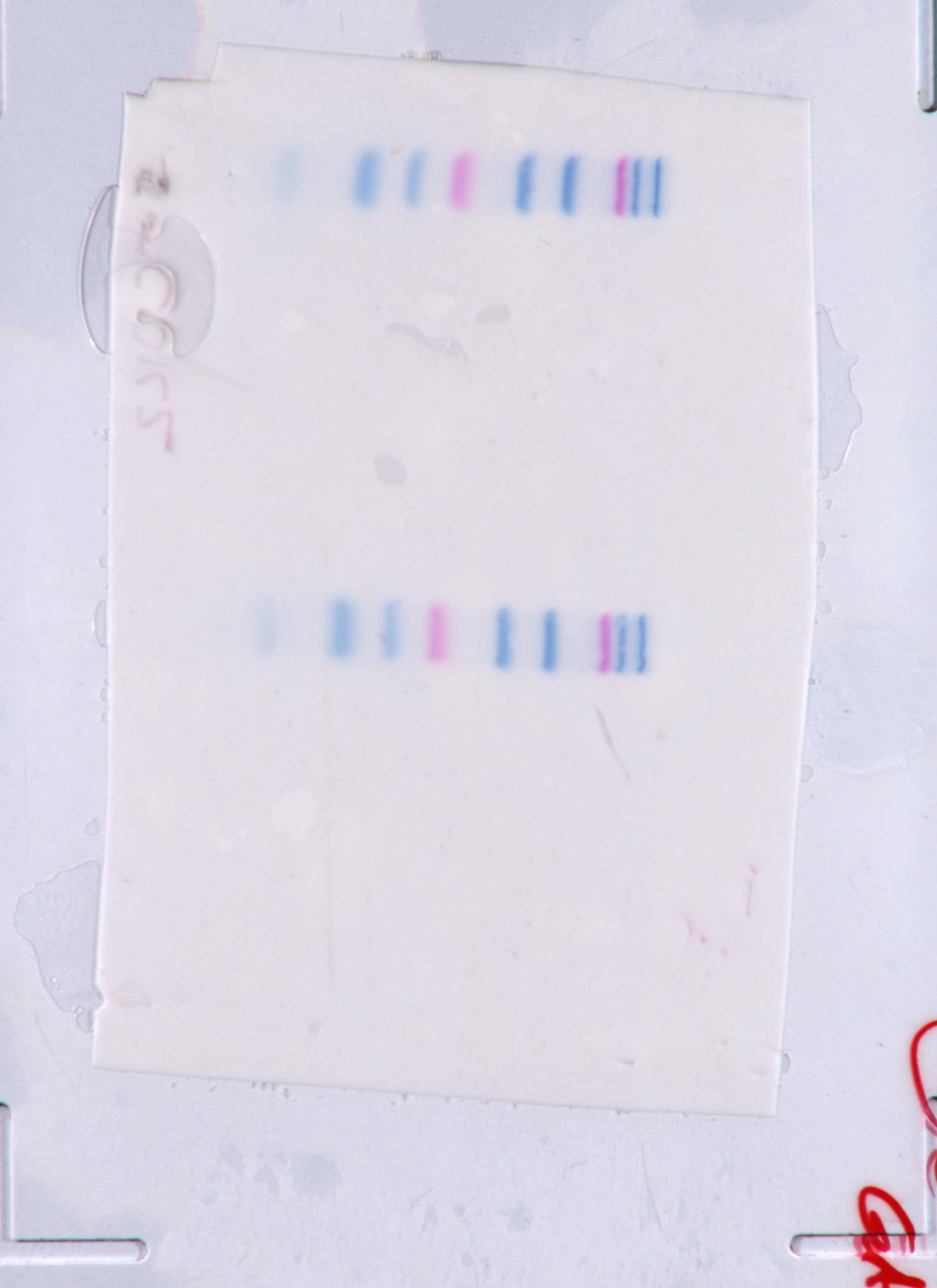

Supplement: Supplementary file 1 [file cancers-16-00370-s001.zip › BGJP_CPDM_2_CDK2_GAP 2022.12.19_13.56.18_Ch-Marker.jpg]

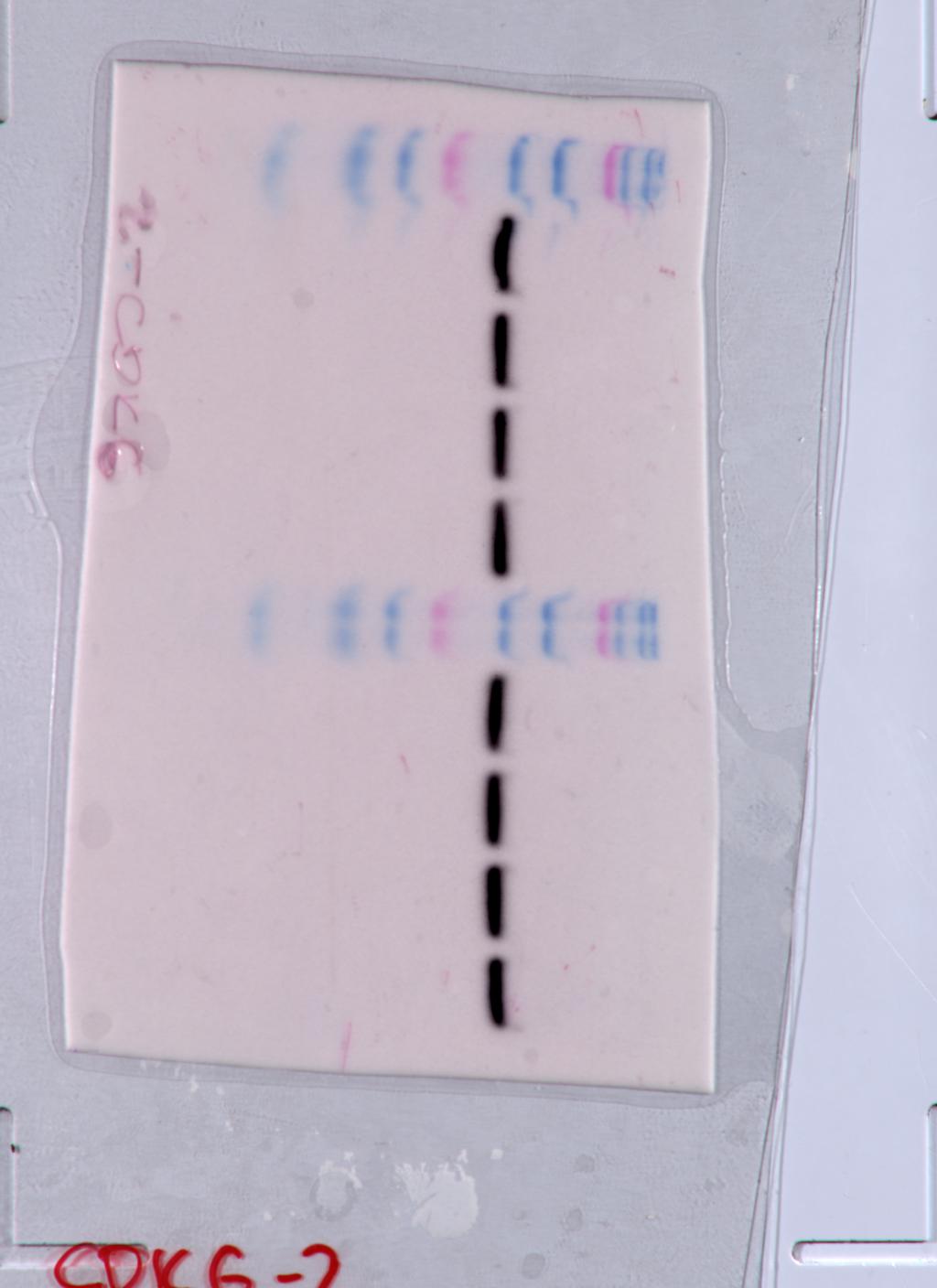

Supplement: Supplementary file 1 [file cancers-16-00370-s001.zip › BGJP_CPDM_2_CDK6 2022.12.17_14.55.39_Ch+Marker.jpg]

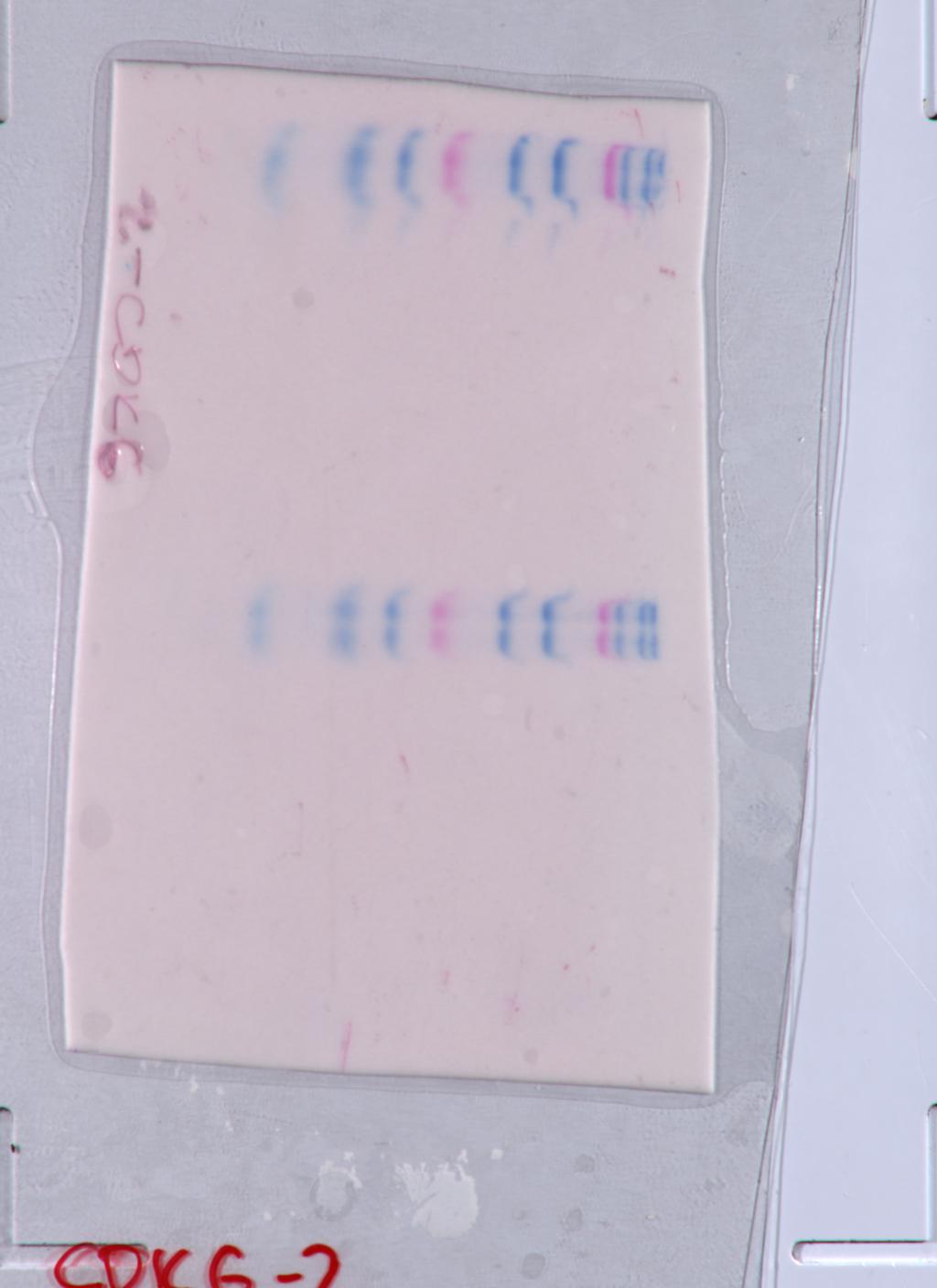

Supplement: Supplementary file 1 [file cancers-16-00370-s001.zip › BGJP_CPDM_2_CDK6 2022.12.17_14.55.39_Ch-Marker.jpg]

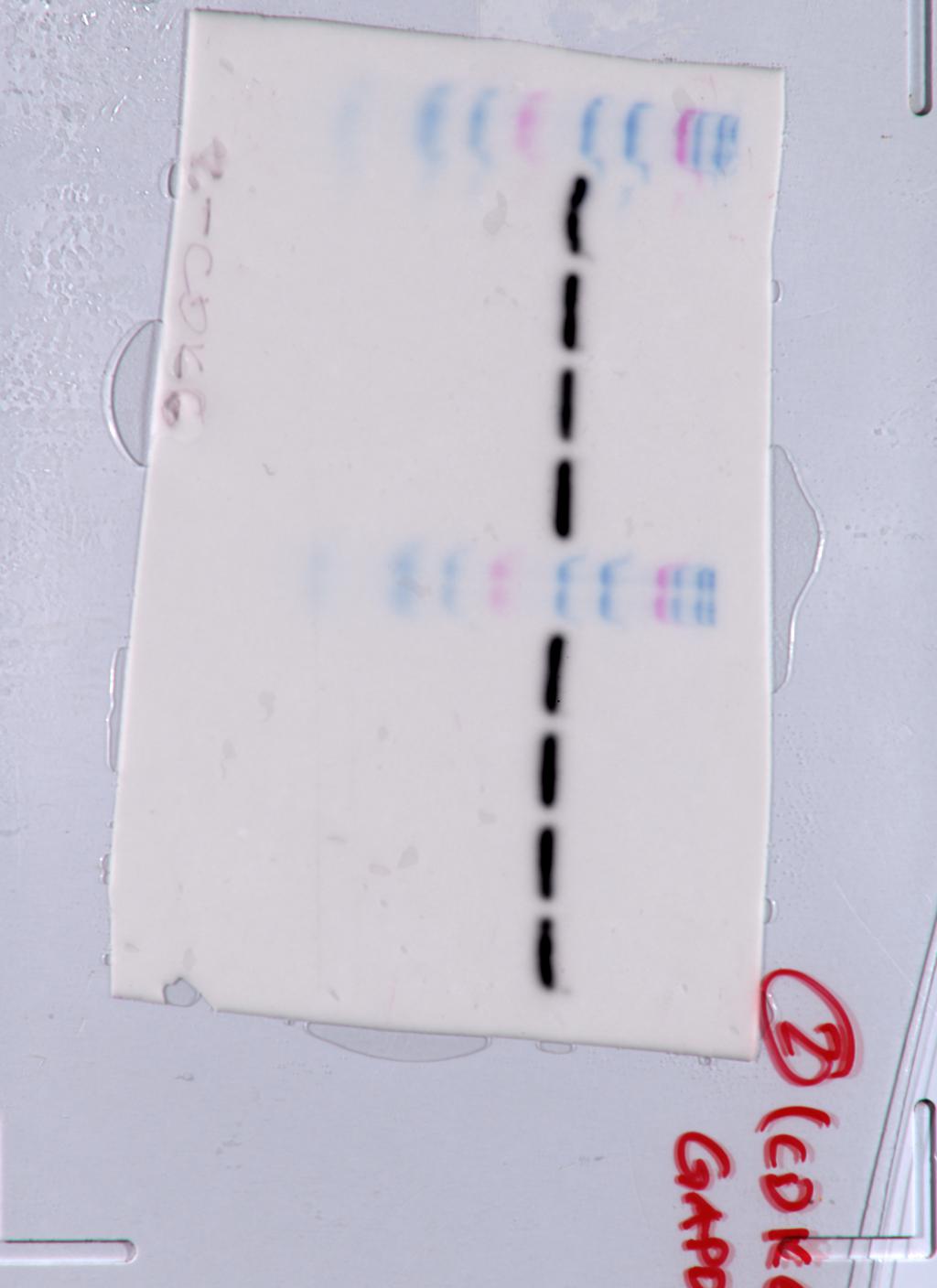

Supplement: Supplementary file 1 [file cancers-16-00370-s001.zip › BGJP_CPDM_2_CDK6_GAP 2022.12.19_14.08.26_Ch+Marker.jpg]

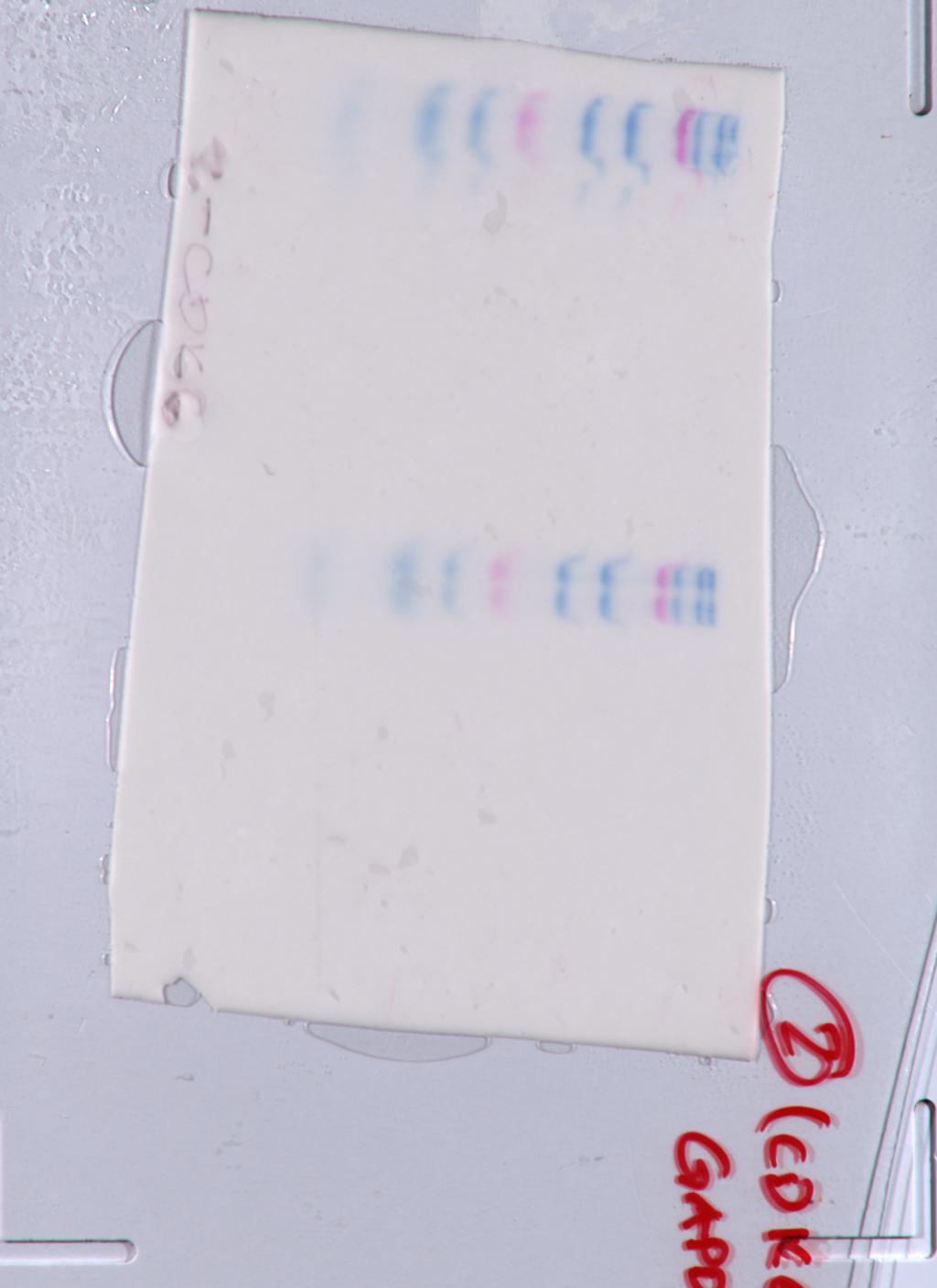

Supplement: Supplementary file 1 [file cancers-16-00370-s001.zip › BGJP_CPDM_2_CDK6_GAP 2022.12.19_14.08.26_Ch-Marker.jpg]

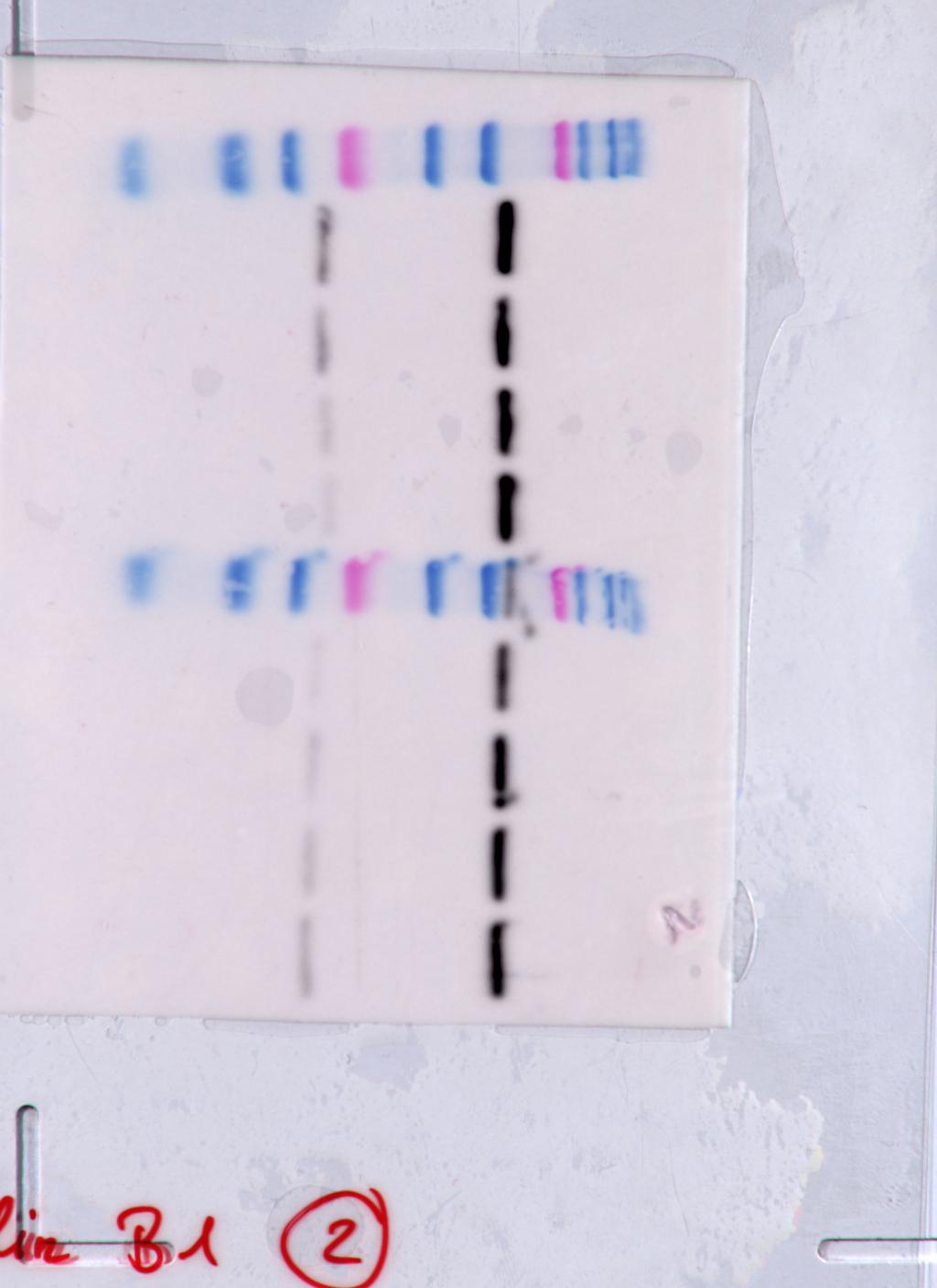

Supplement: Supplementary file 1 [file cancers-16-00370-s001.zip › BGJP_CPDM_2_CycB1 2022.12.17_14.31.09_Ch+Marker.jpg]

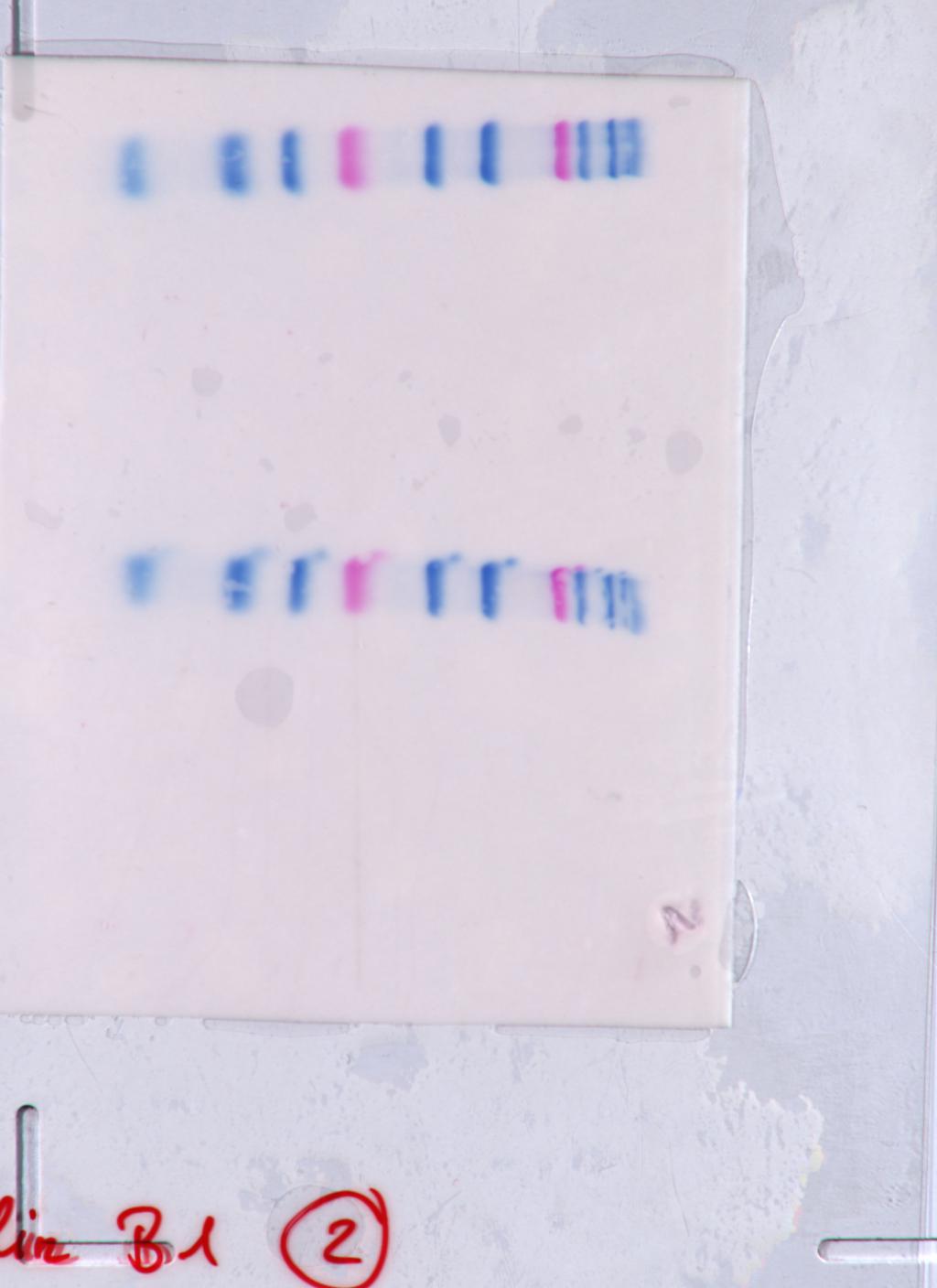

Supplement: Supplementary file 1 [file cancers-16-00370-s001.zip › BGJP_CPDM_2_CycB1 2022.12.17_14.31.09_Ch-Marker.jpg]

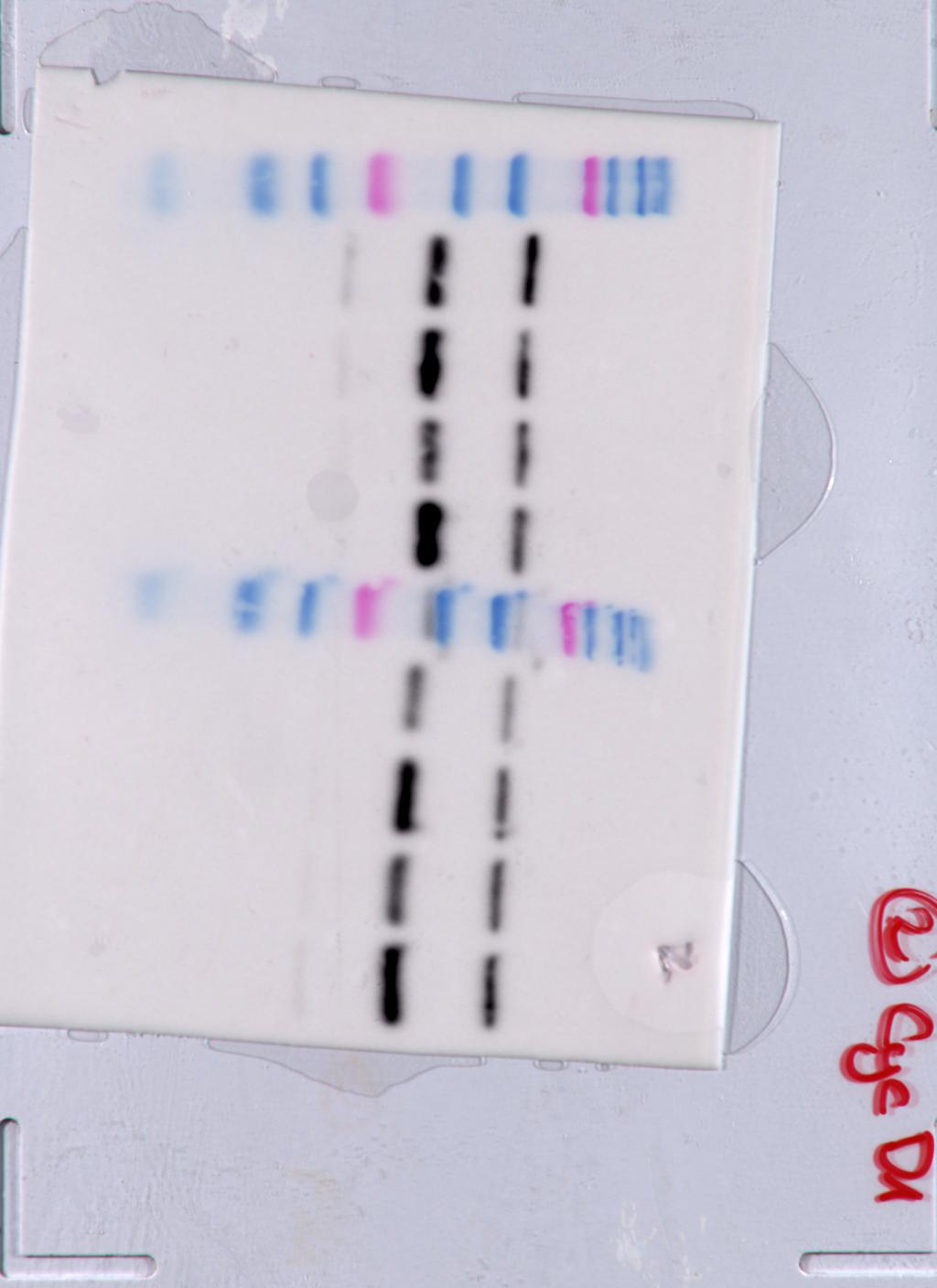

Supplement: Supplementary file 1 [file cancers-16-00370-s001.zip › BGJP_CPDM_2_CycD1 2022.12.18_12.33.36_Ch+Marker.jpg]

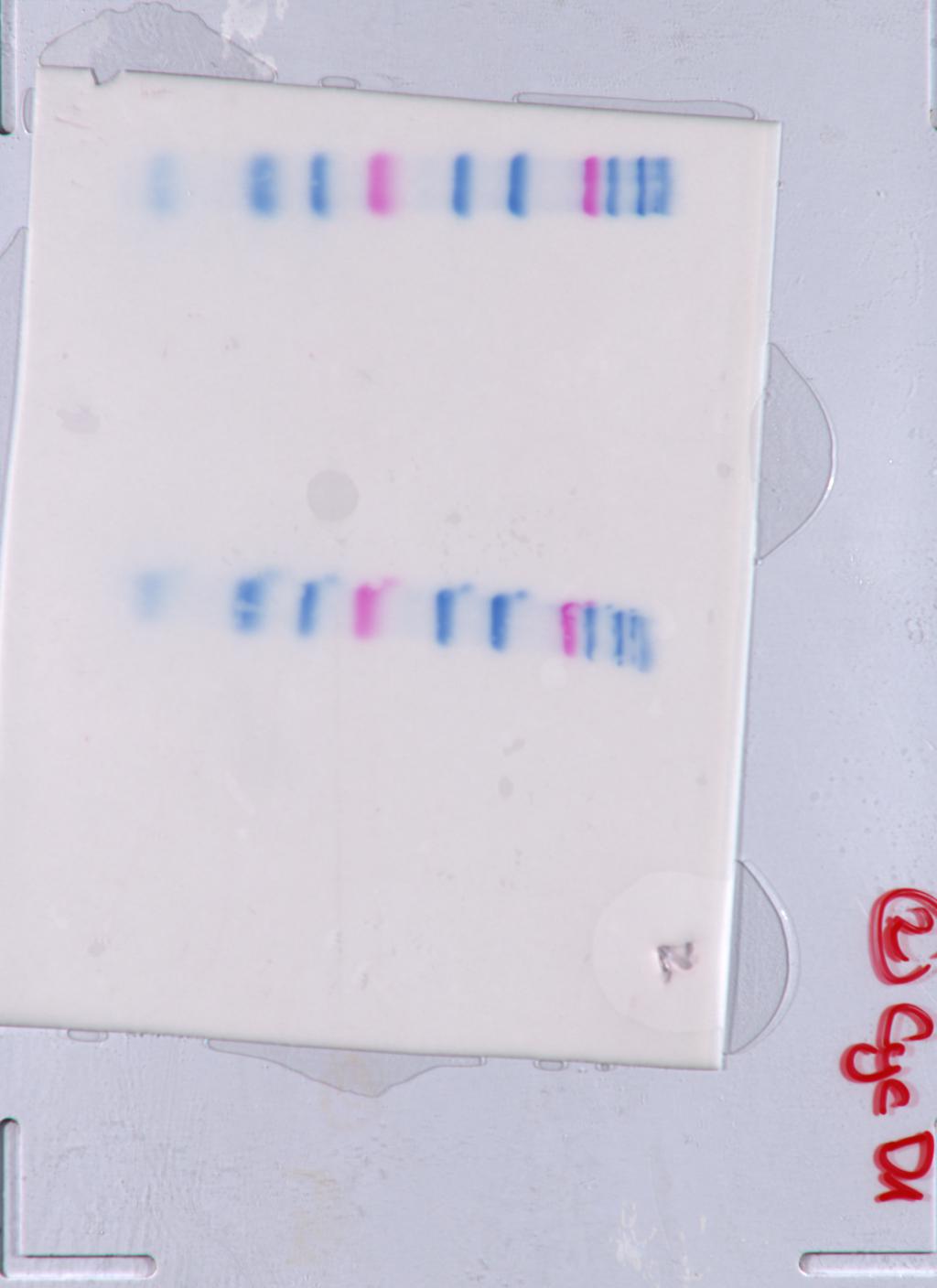

Supplement: Supplementary file 1 [file cancers-16-00370-s001.zip › BGJP_CPDM_2_CycD1 2022.12.18_12.33.36_Ch-Marker.jpg]

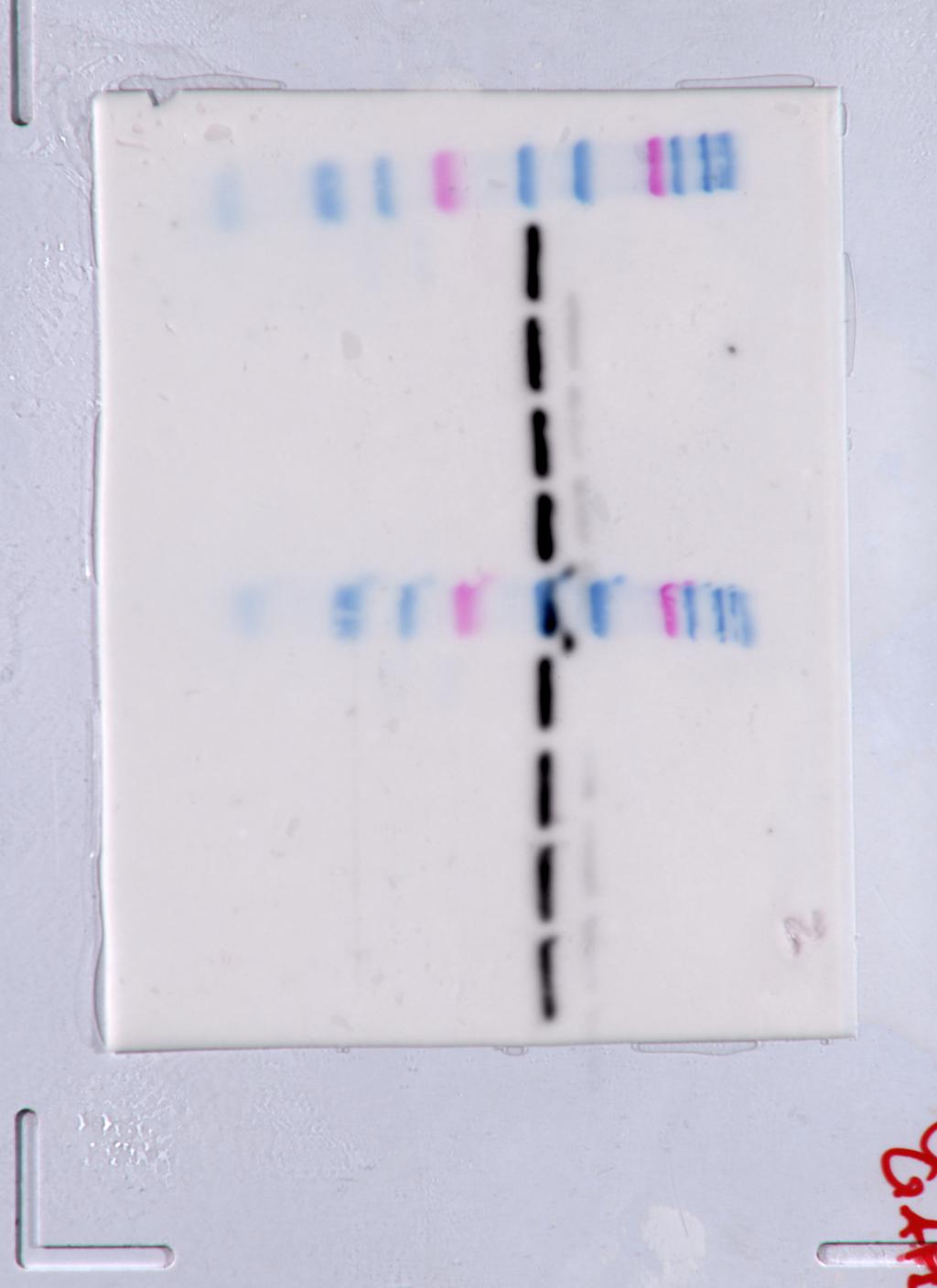

Supplement: Supplementary file 1 [file cancers-16-00370-s001.zip › BGJP_CPDM_2_Cycl_GAP 2022.12.19_14.20.11_Ch+Marker.jpg]

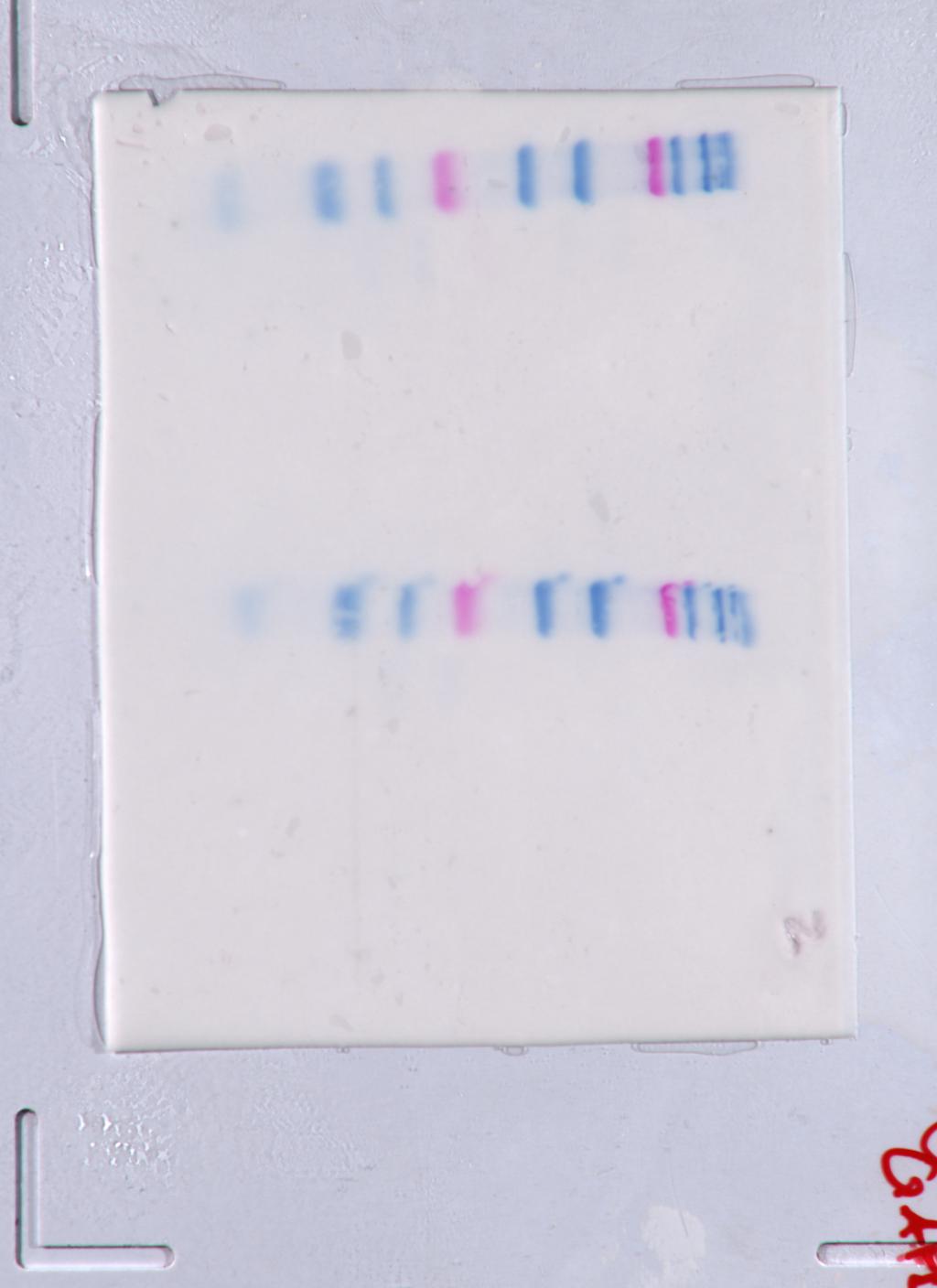

Supplement: Supplementary file 1 [file cancers-16-00370-s001.zip › BGJP_CPDM_2_Cycl_GAP 2022.12.19_14.20.11_Ch-Marker.jpg]

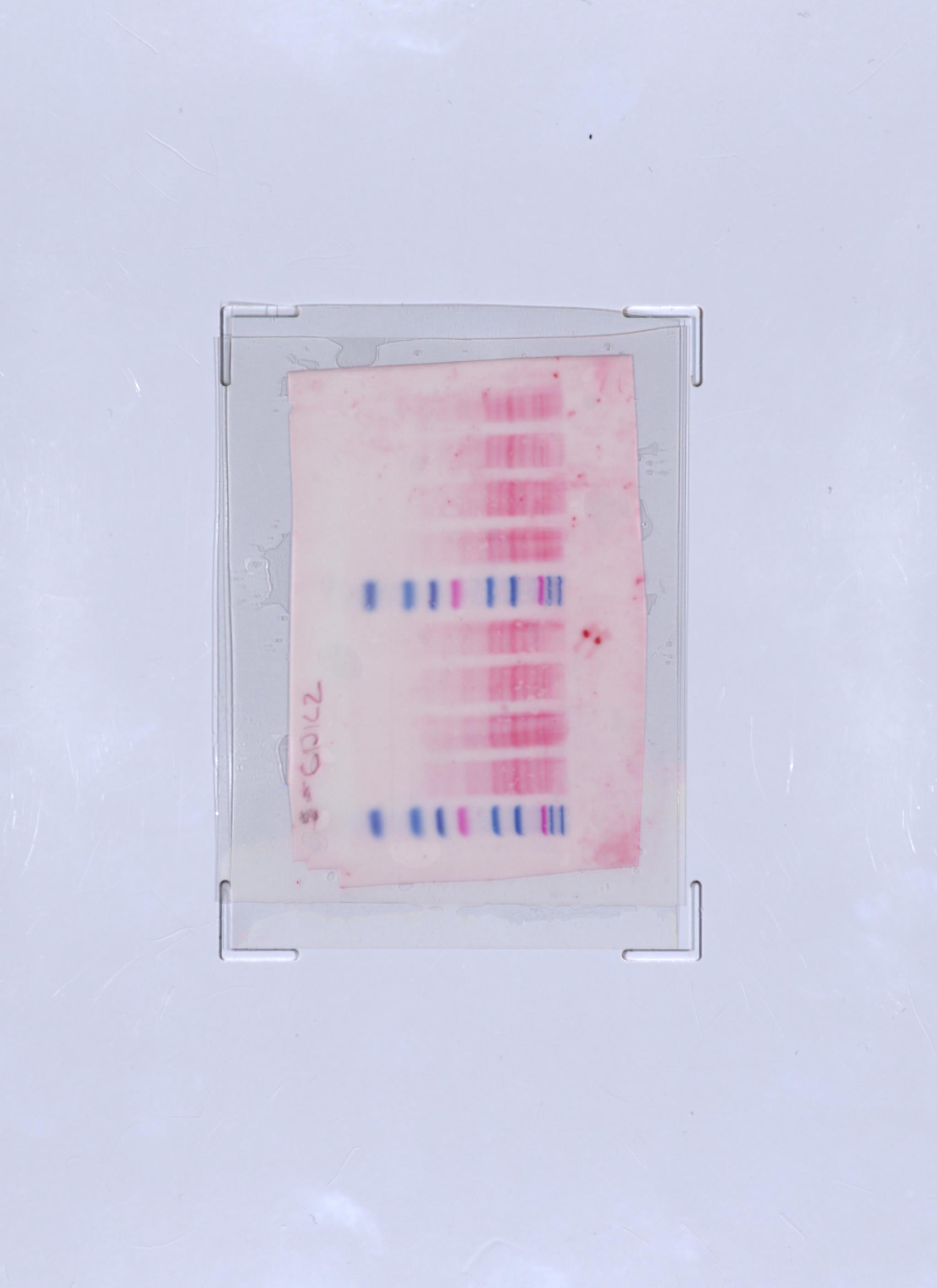

Supplement: Supplementary file 1 [file cancers-16-00370-s001.zip › BGJP_CPDM_2_G1_Ponc 2022.12.13_14.31.02_Co.jpg]

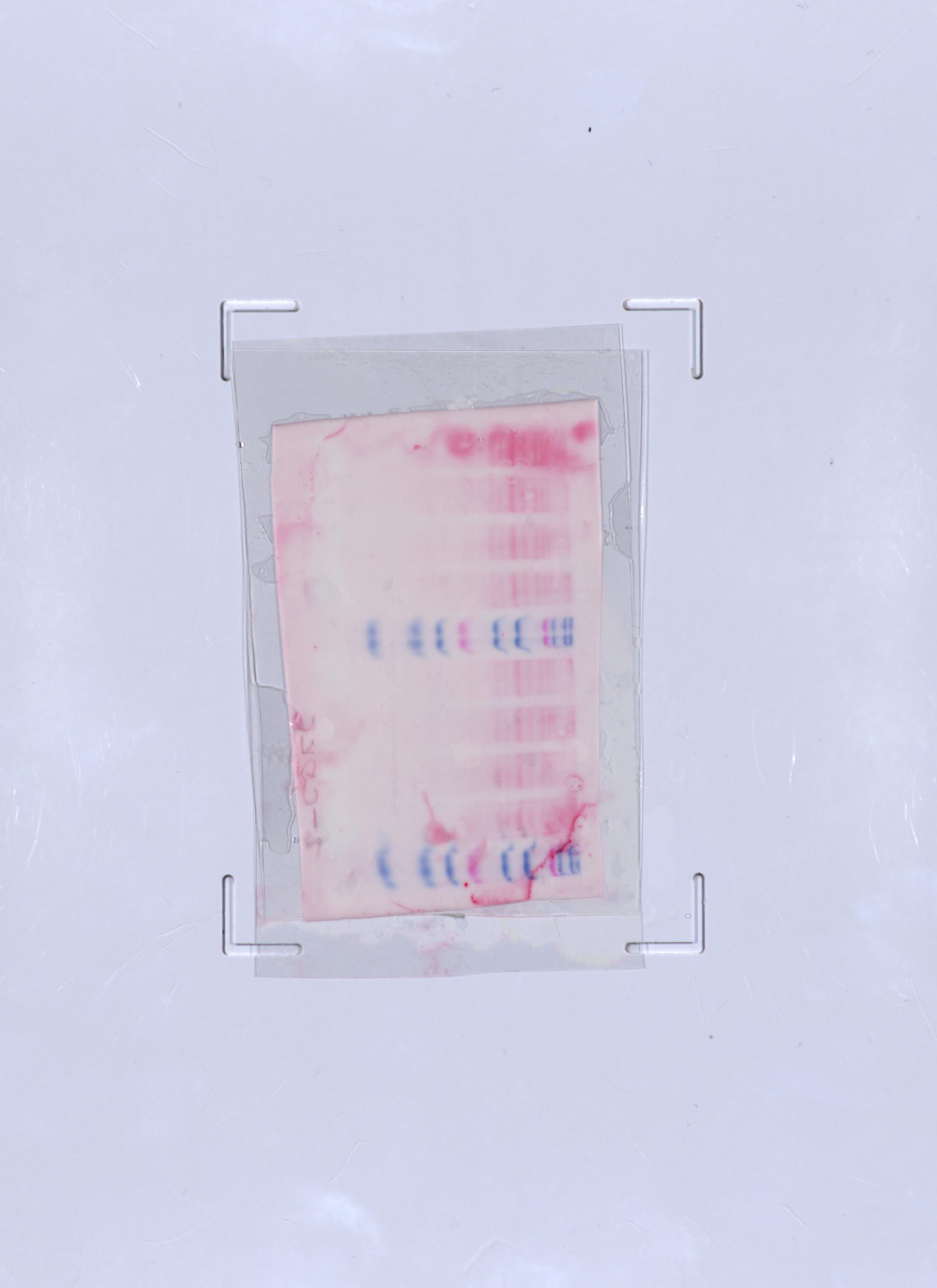

Supplement: Supplementary file 1 [file cancers-16-00370-s001.zip › BGJP_CPDM_2_G2_Ponc 2022.12.13_14.32.55_Co.jpg]

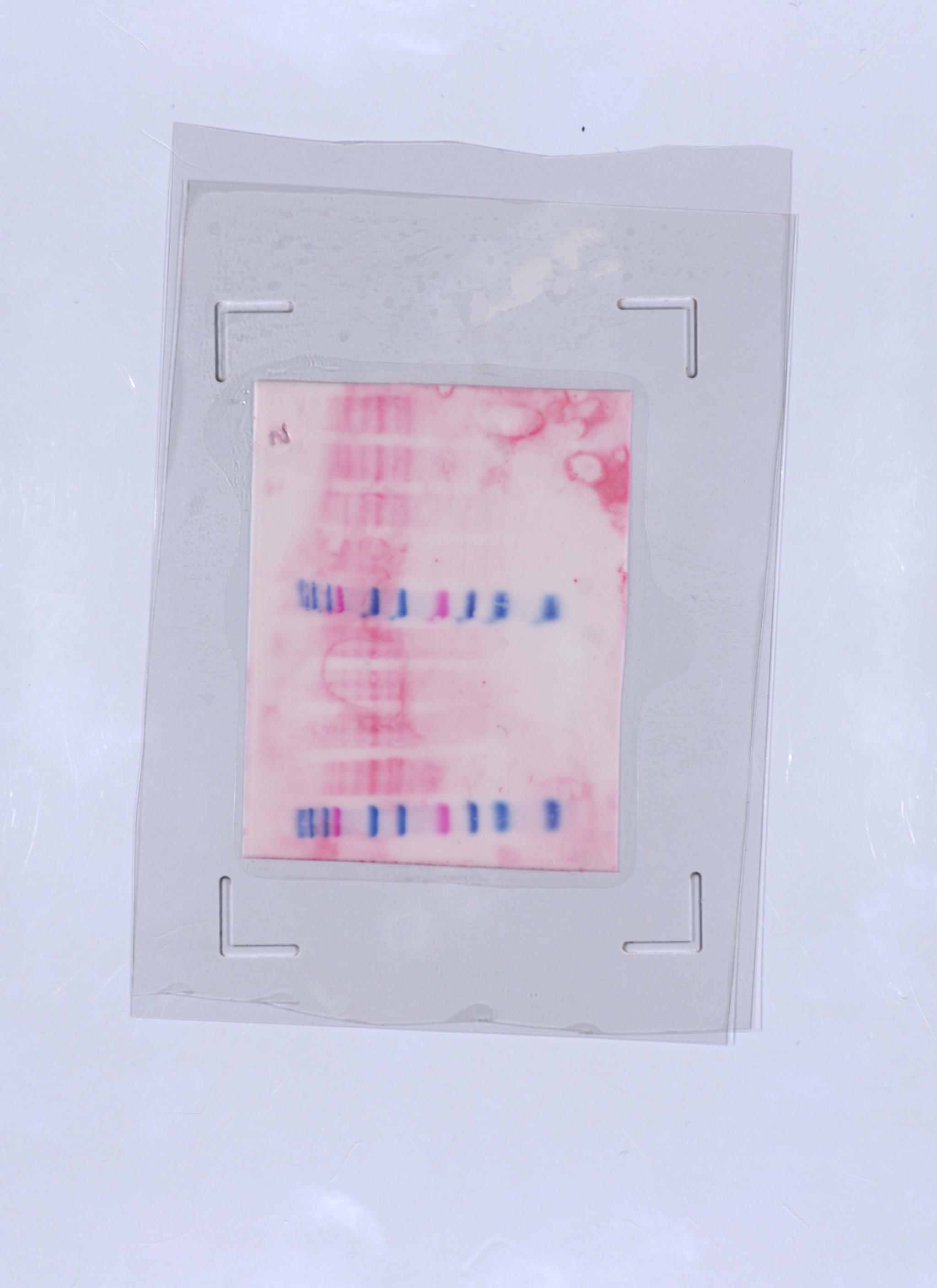

Supplement: Supplementary file 1 [file cancers-16-00370-s001.zip › BGJP_CPDM_2_G3_Ponc 2022.12.14_12.53.07_Co.jpg]

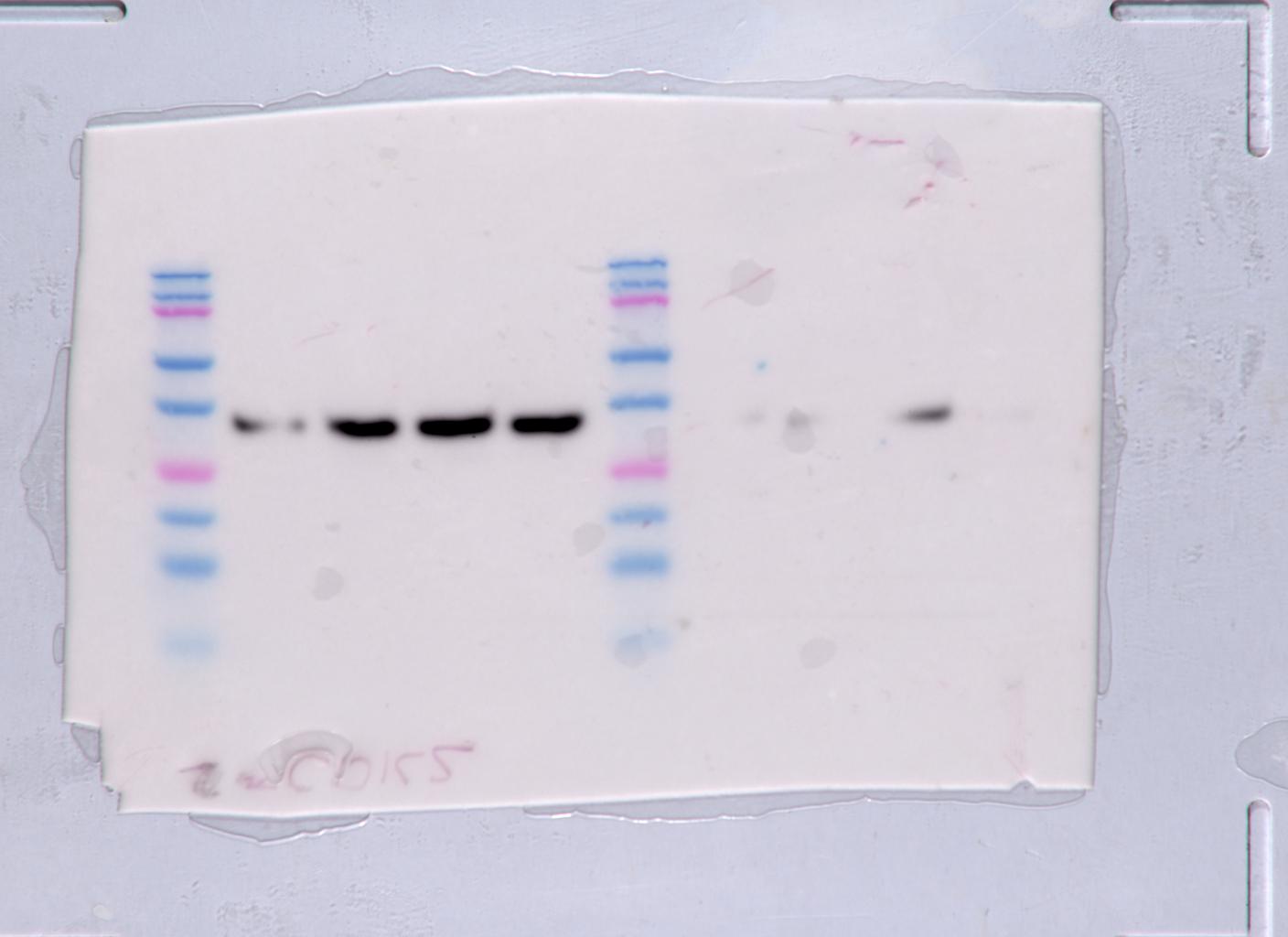

Supplement: Supplementary file 1 [file cancers-16-00370-s001.zip › BGJP_CPDM_2_p21 2022.12.18_12.46.17_Ch+Marker.jpg]

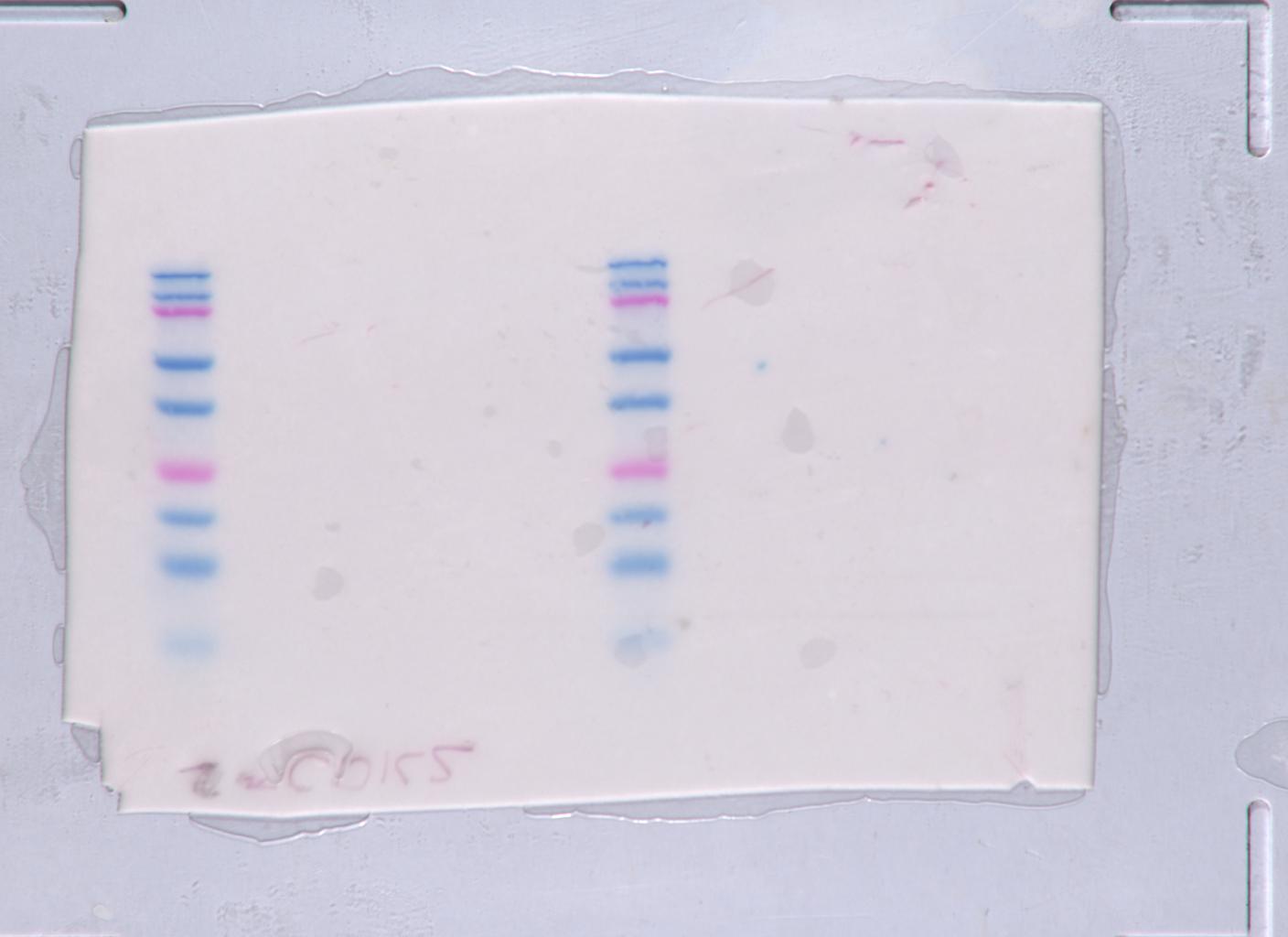

Supplement: Supplementary file 1 [file cancers-16-00370-s001.zip › BGJP_CPDM_2_p21 2022.12.18_12.46.17_Ch-Marker.jpg]

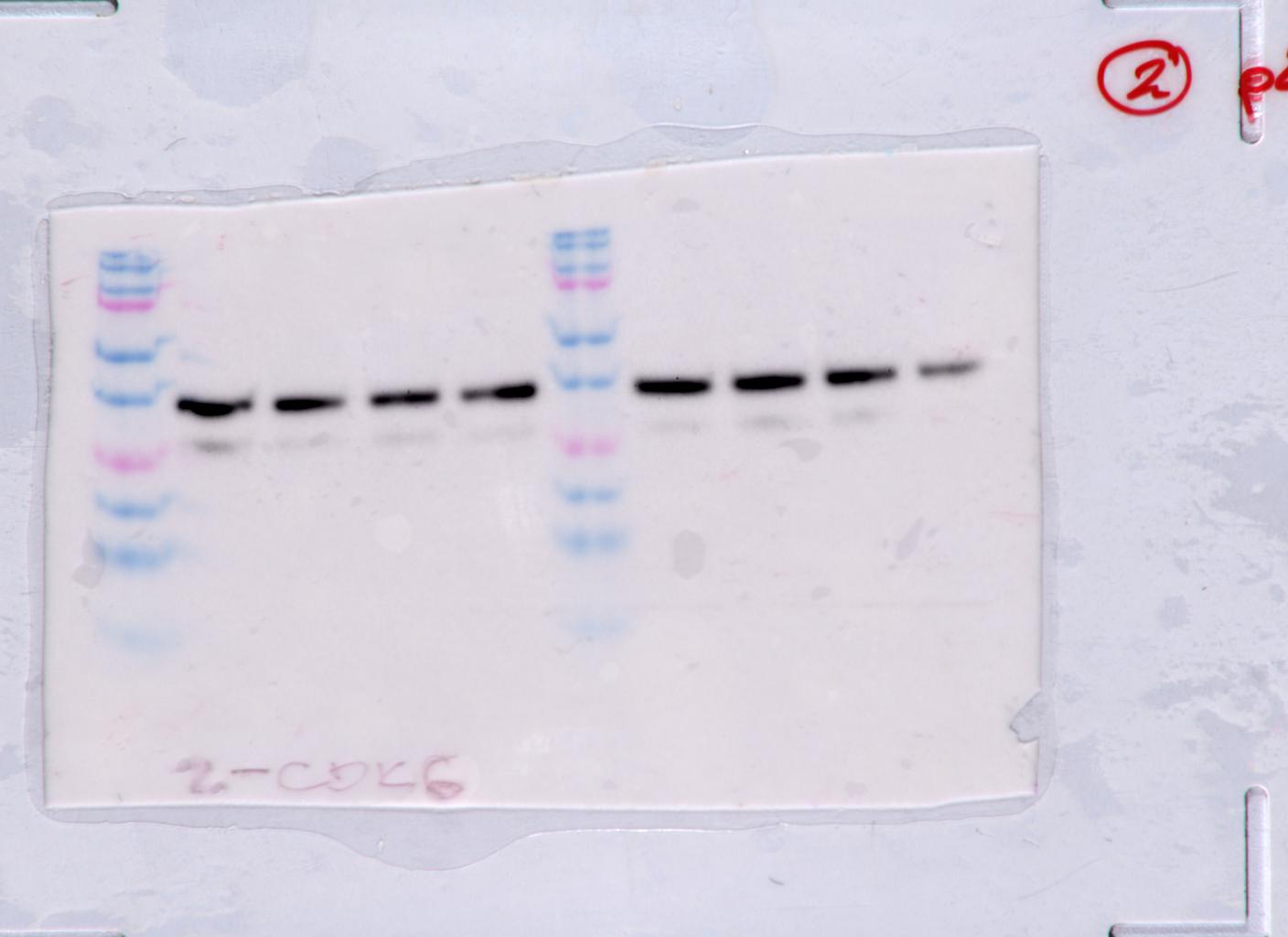

Supplement: Supplementary file 1 [file cancers-16-00370-s001.zip › BGJP_CPDM_2_p27 2022.12.18_12.58.42_Ch+Marker.jpg]

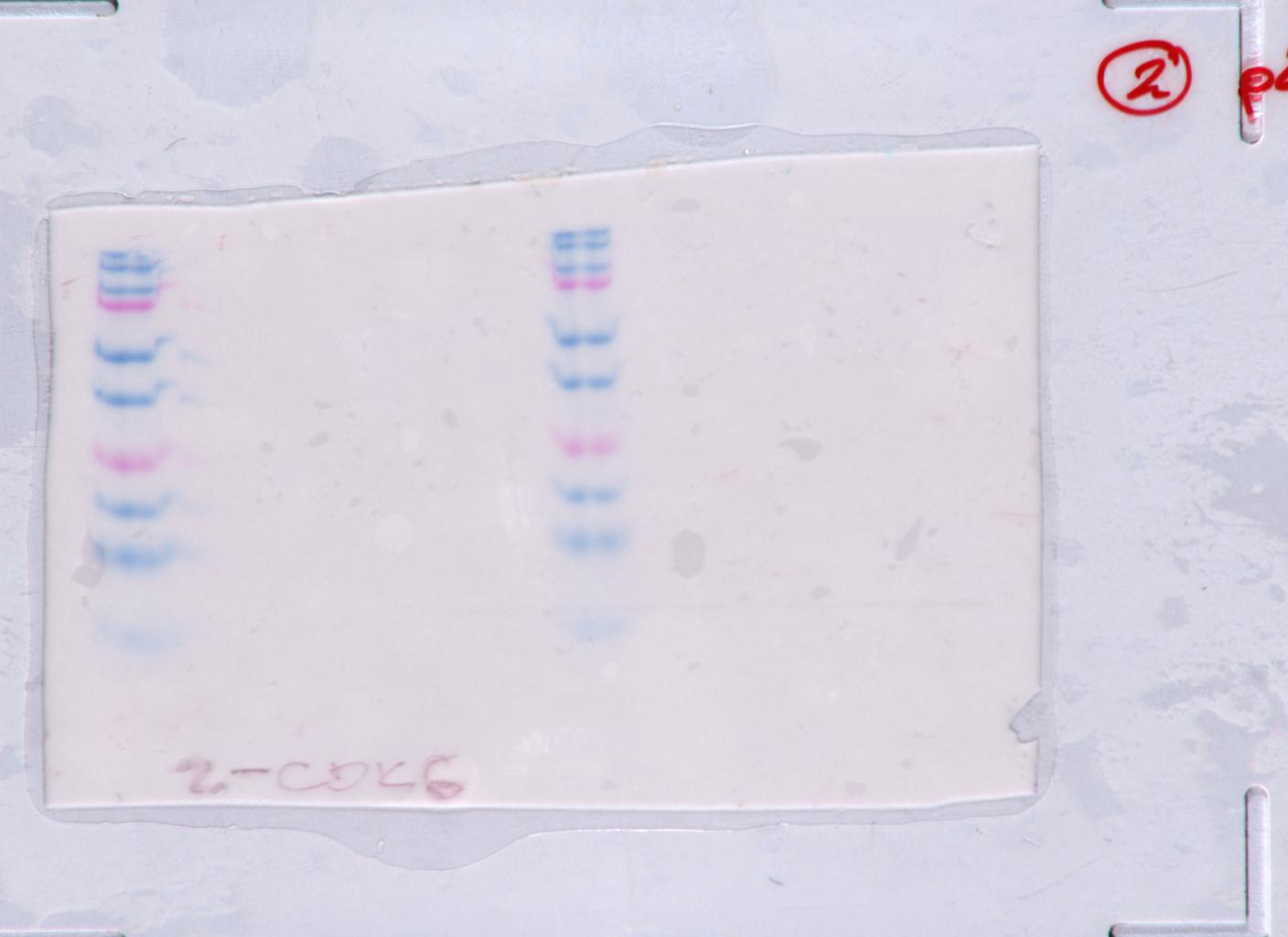

Supplement: Supplementary file 1 [file cancers-16-00370-s001.zip › BGJP_CPDM_2_p27 2022.12.18_12.58.42_Ch-Marker.jpg]

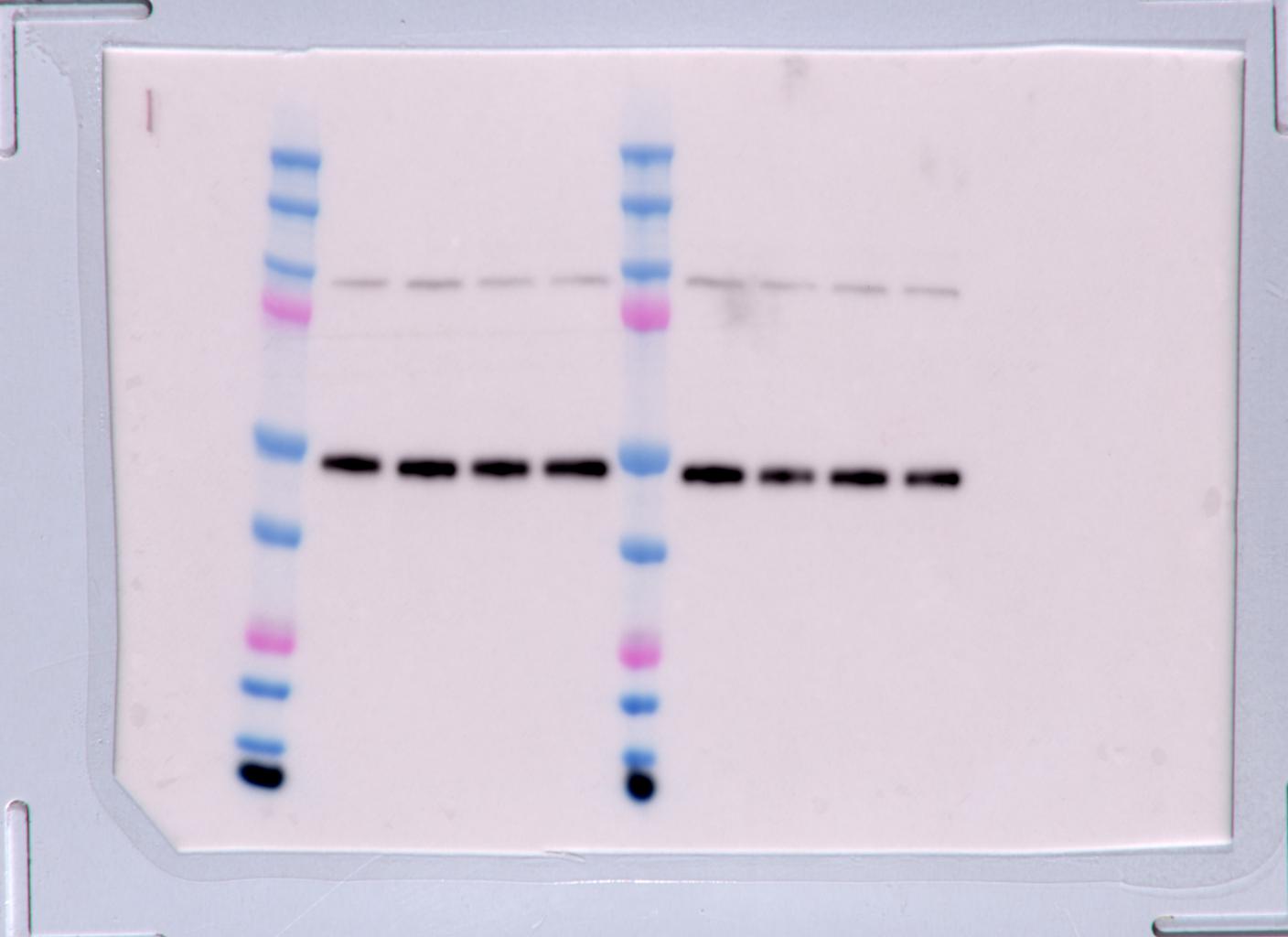

Supplement: Supplementary file 1 [file cancers-16-00370-s001.zip › CSCC1 p53 10.5.22 jp 2022.05.10_15.57.17_Ch+Marker.jpg]

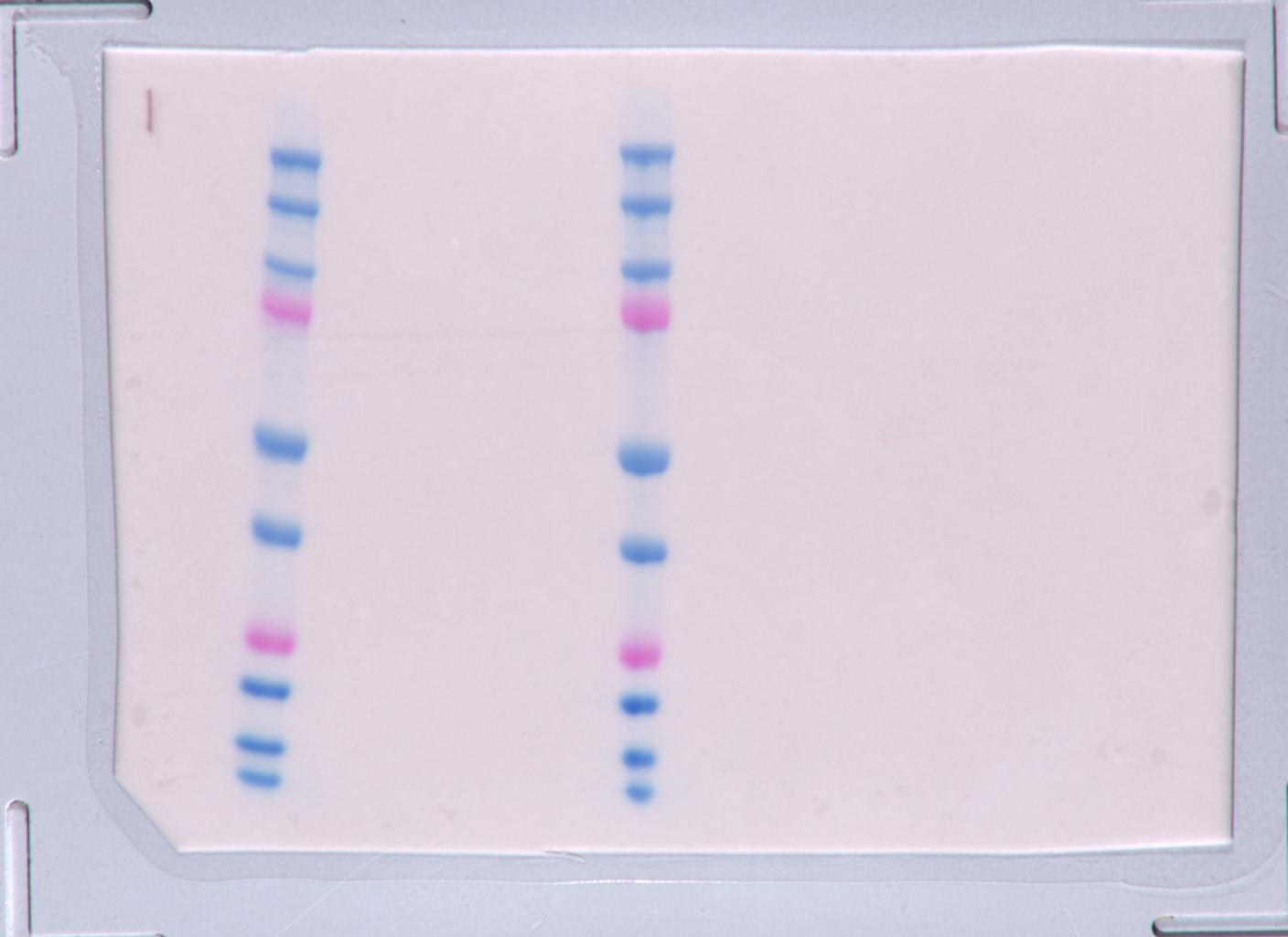

Supplement: Supplementary file 1 [file cancers-16-00370-s001.zip › CSCC1 p53 10.5.22 jp 2022.05.10_15.57.17_Ch-Marker.jpg]

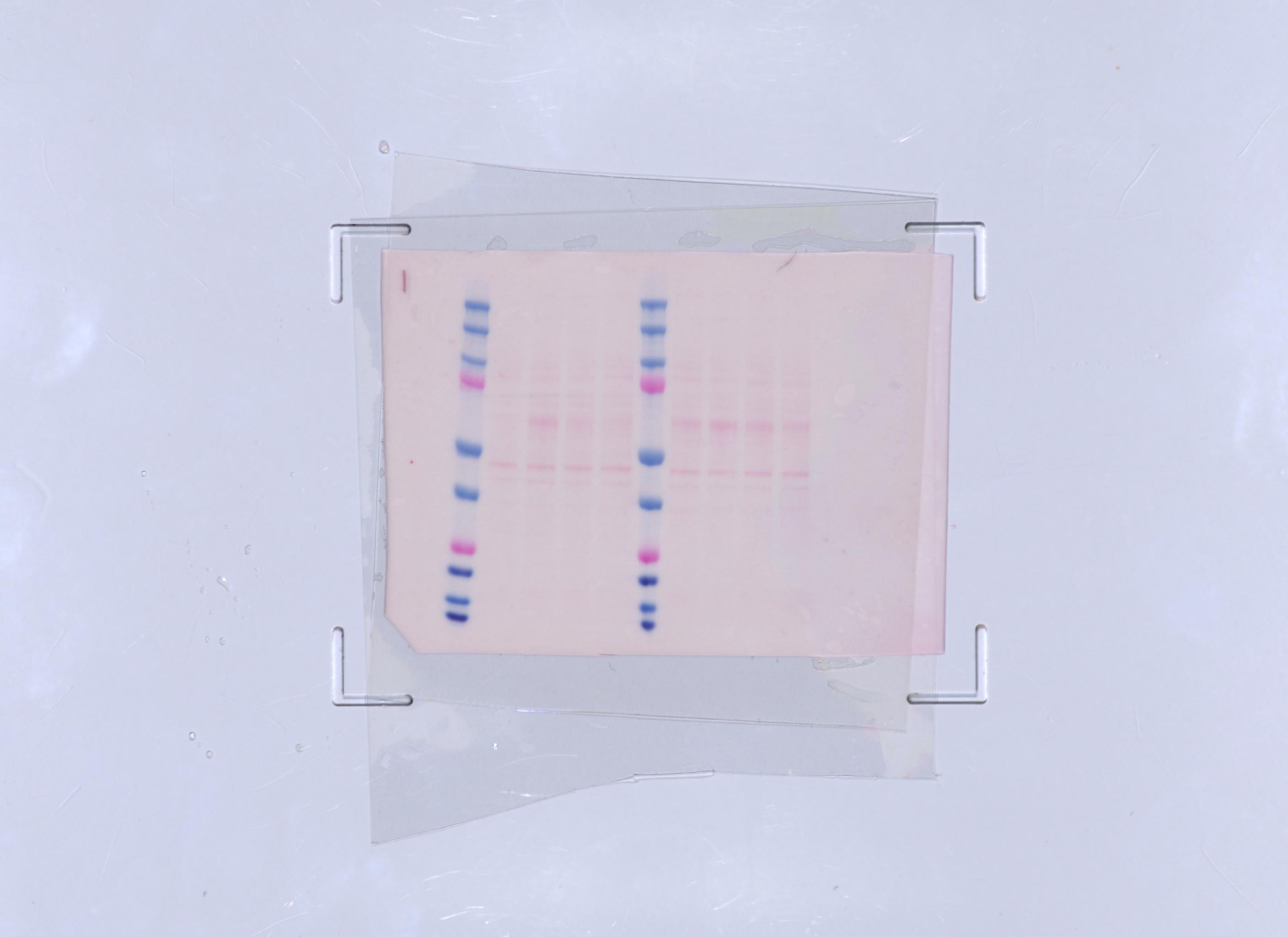

Supplement: Supplementary file 1 [file cancers-16-00370-s001.zip › CSCC1 p53 pon 9-5-22 2022.05.09_15.55.34_Co.jpg]

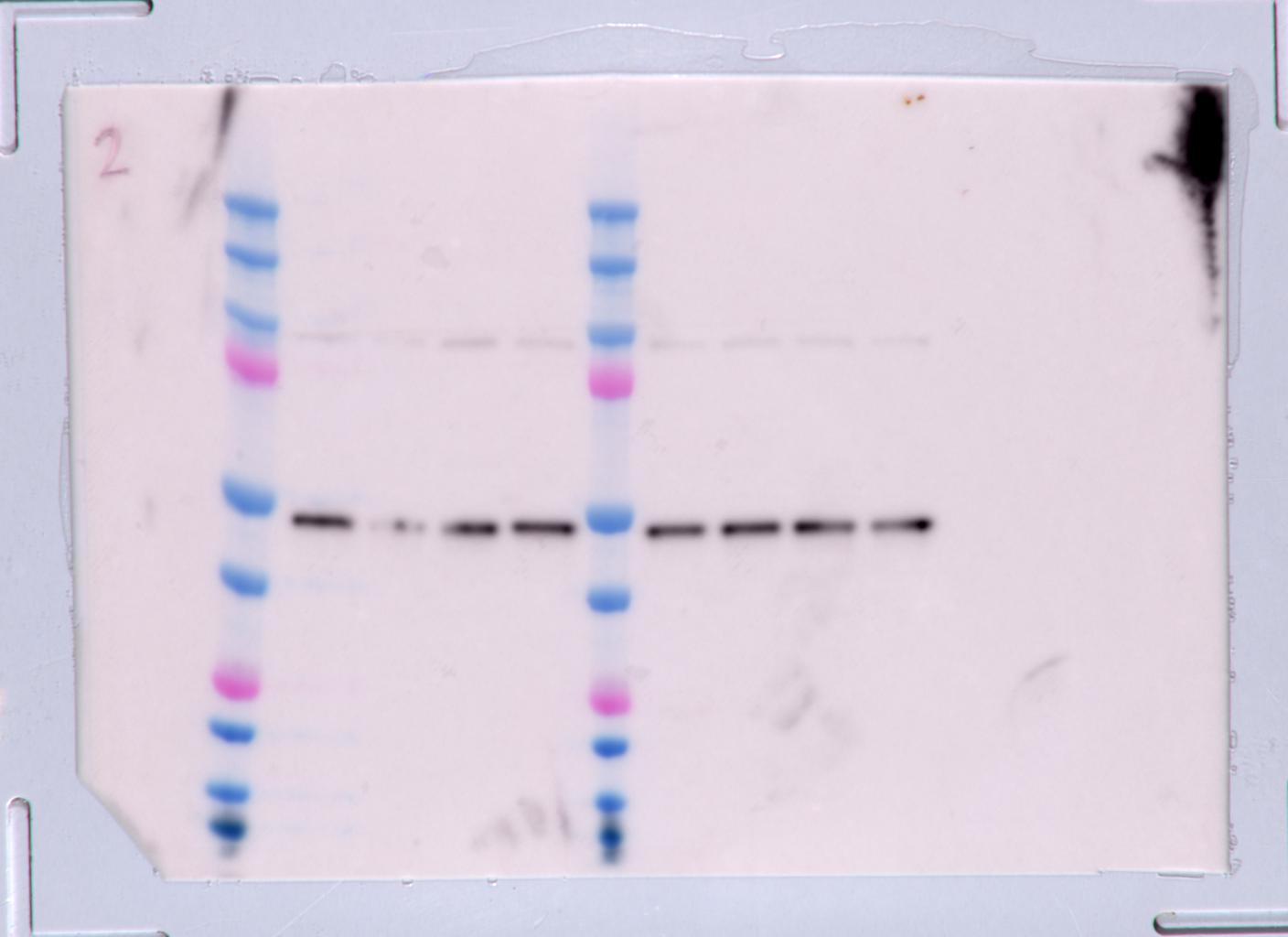

Supplement: Supplementary file 1 [file cancers-16-00370-s001.zip › CSCC2 p53 10.5.22 jp 2022.05.10_16.10.35_Ch+Marker.jpg]

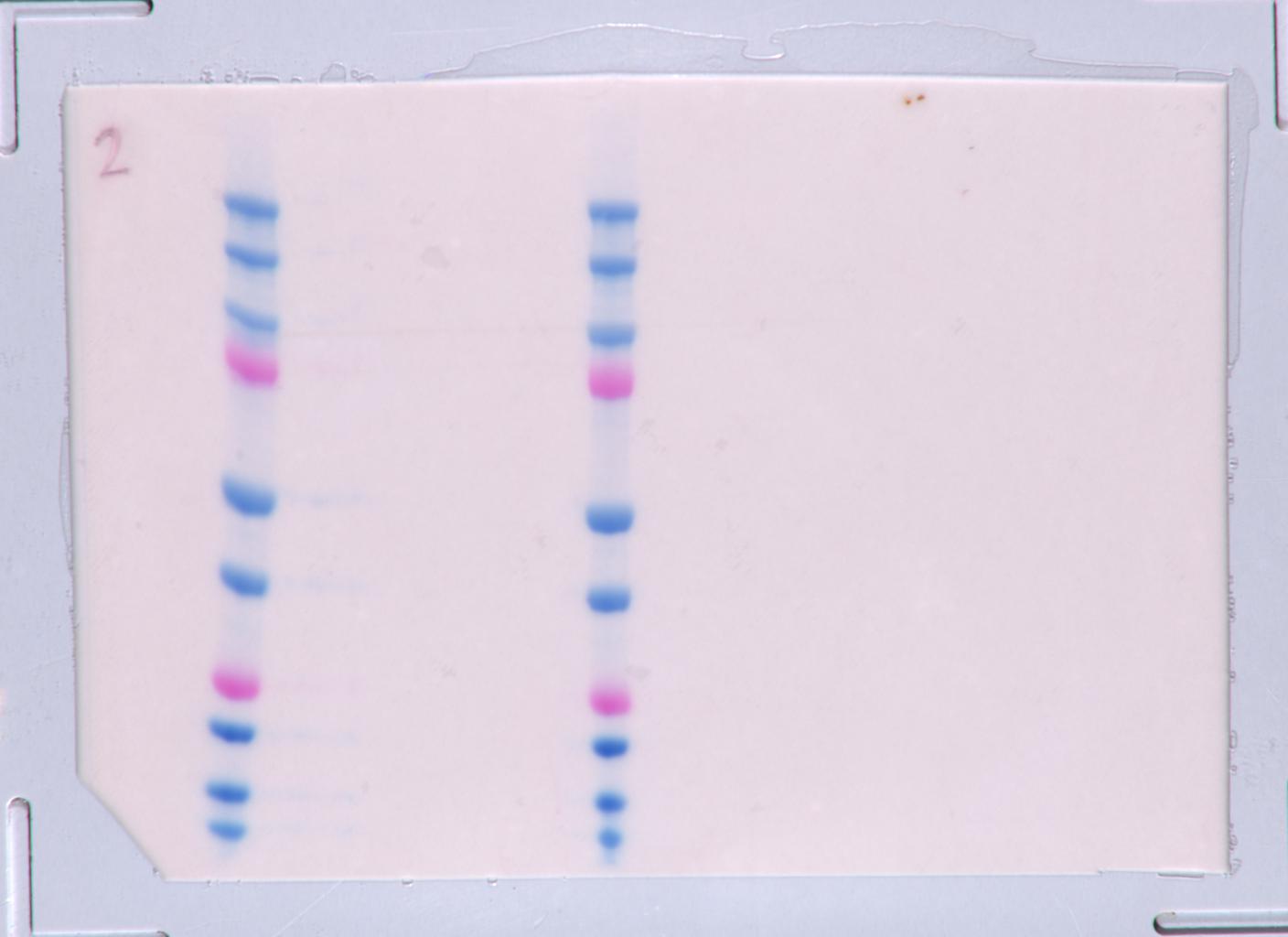

Supplement: Supplementary file 1 [file cancers-16-00370-s001.zip › CSCC2 p53 10.5.22 jp 2022.05.10_16.10.35_Ch-Marker.jpg]

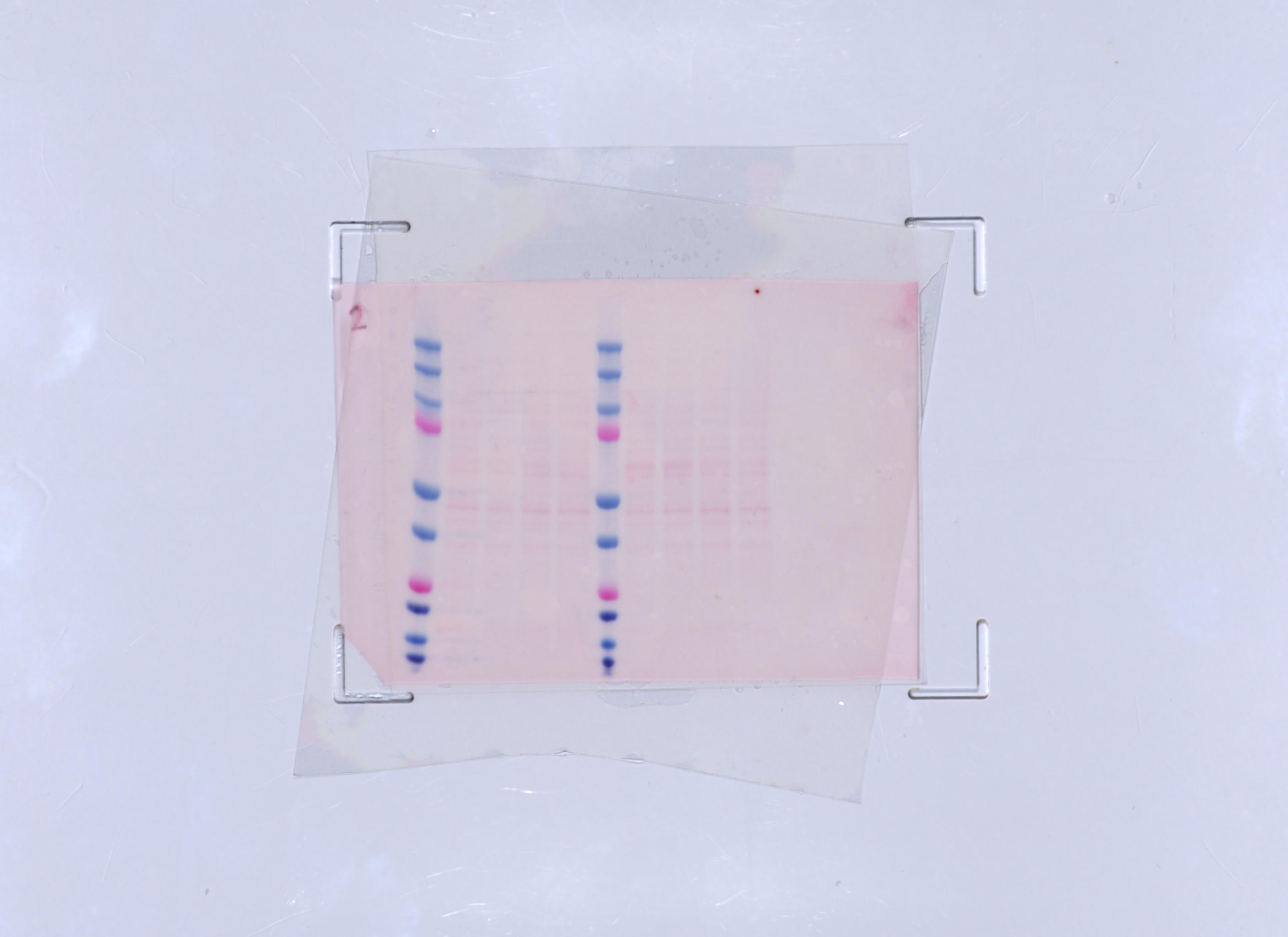

Supplement: Supplementary file 1 [file cancers-16-00370-s001.zip › CSCC2 p53 pon 9-5-22 2022.05.09_15.57.54_Co.jpg]

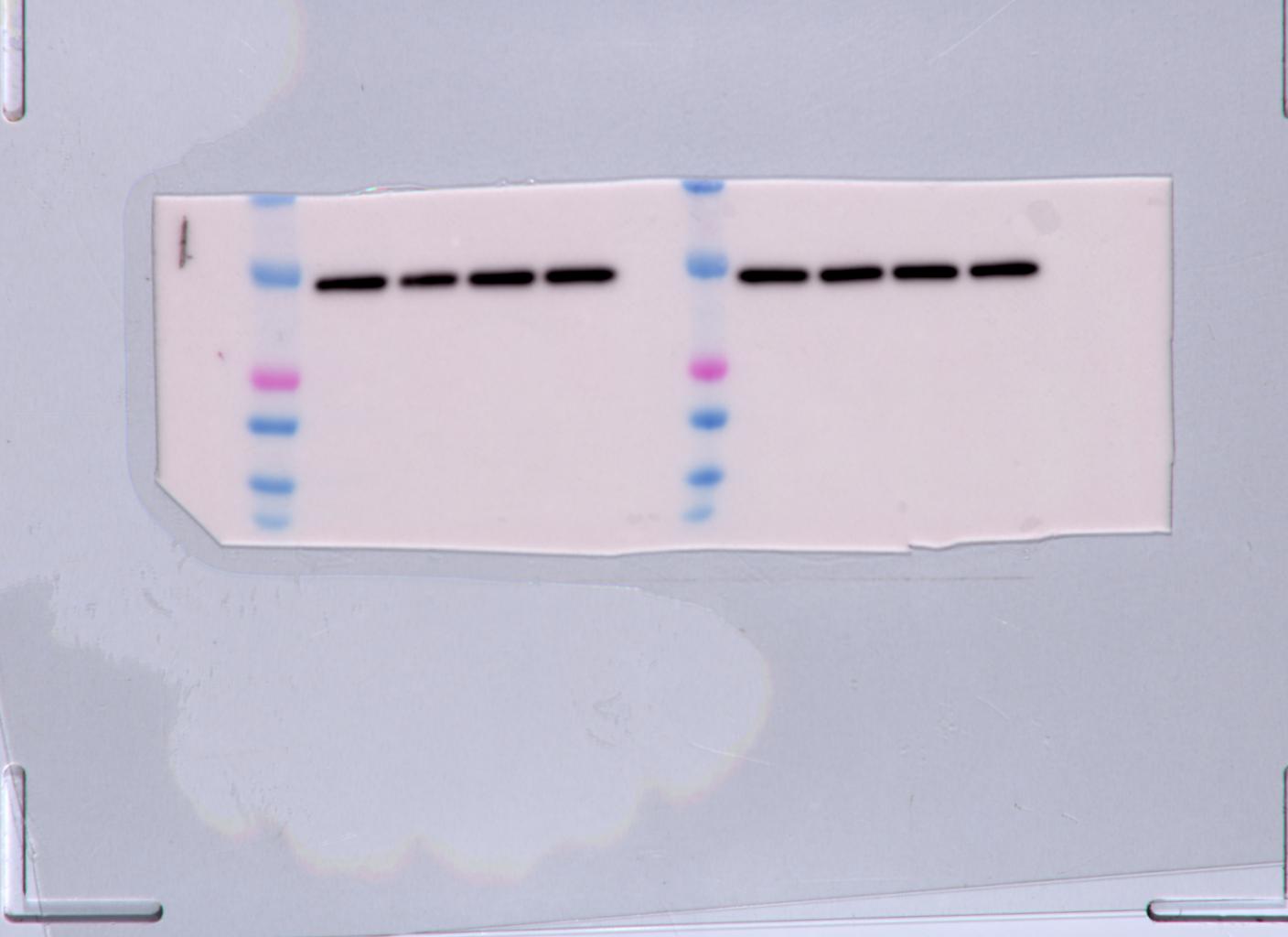

Supplement: Supplementary file 1 [file cancers-16-00370-s001.zip › GAPDH1 cscc2 31-3-22 2022.03.31_15.57.36_Ch+Marker.jpg]

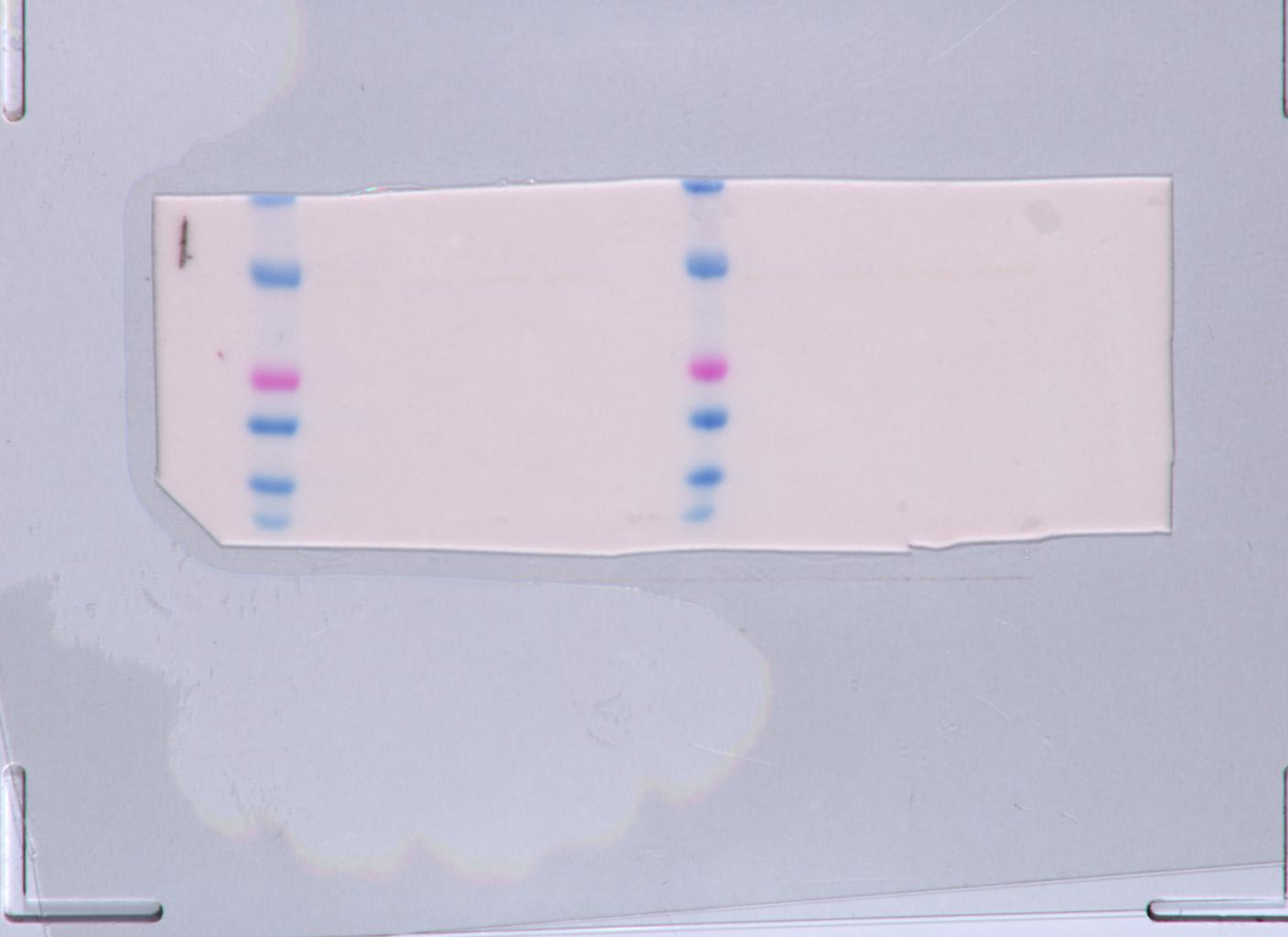

Supplement: Supplementary file 1 [file cancers-16-00370-s001.zip › GAPDH1 cscc2 31-3-22 2022.03.31_15.57.36_Ch-Marker.jpg]

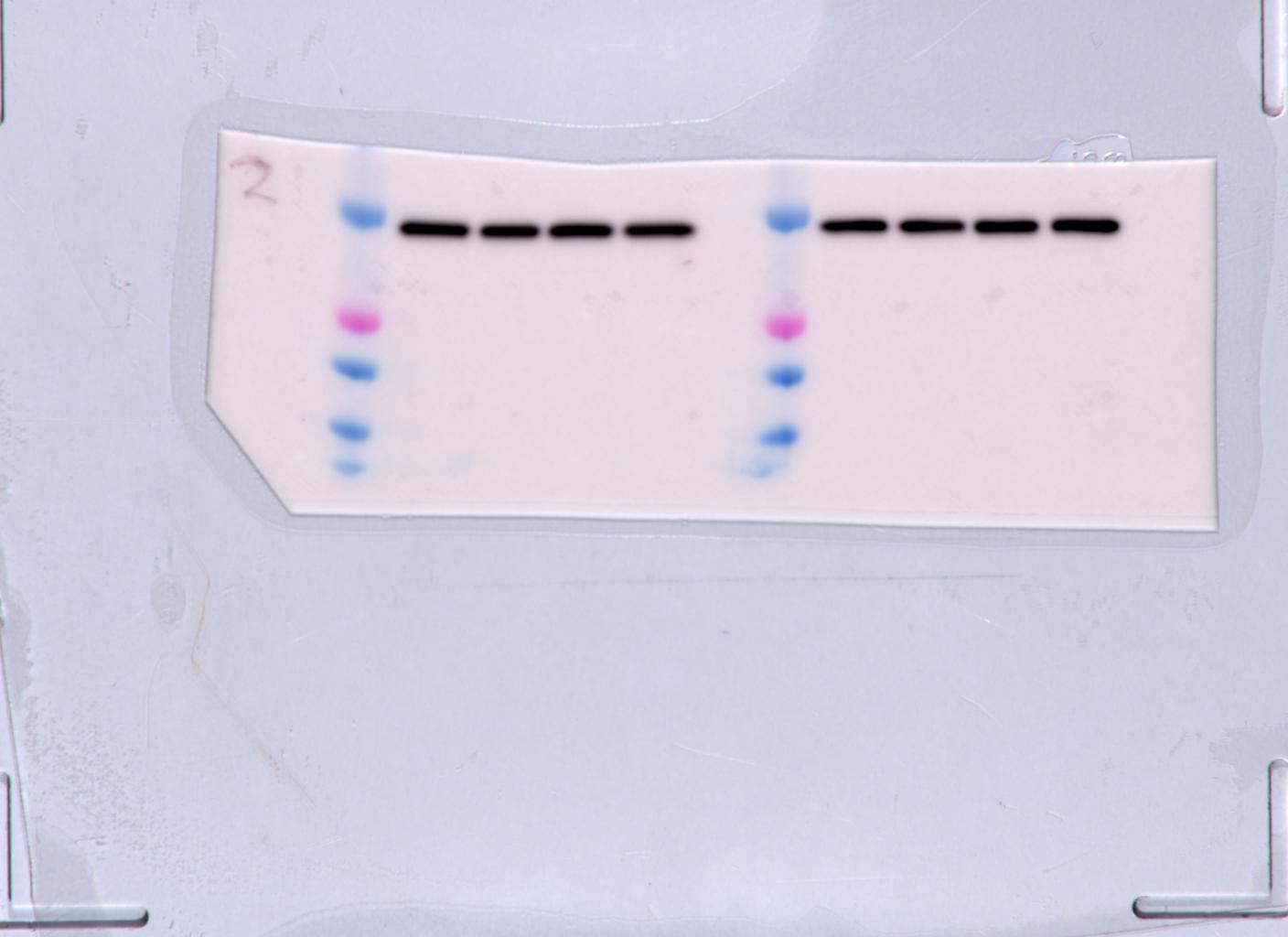

Supplement: Supplementary file 1 [file cancers-16-00370-s001.zip › GAPDH2 cscc2 31-3-22 2022.03.31_15.59.19_Ch+Marker.jpg]

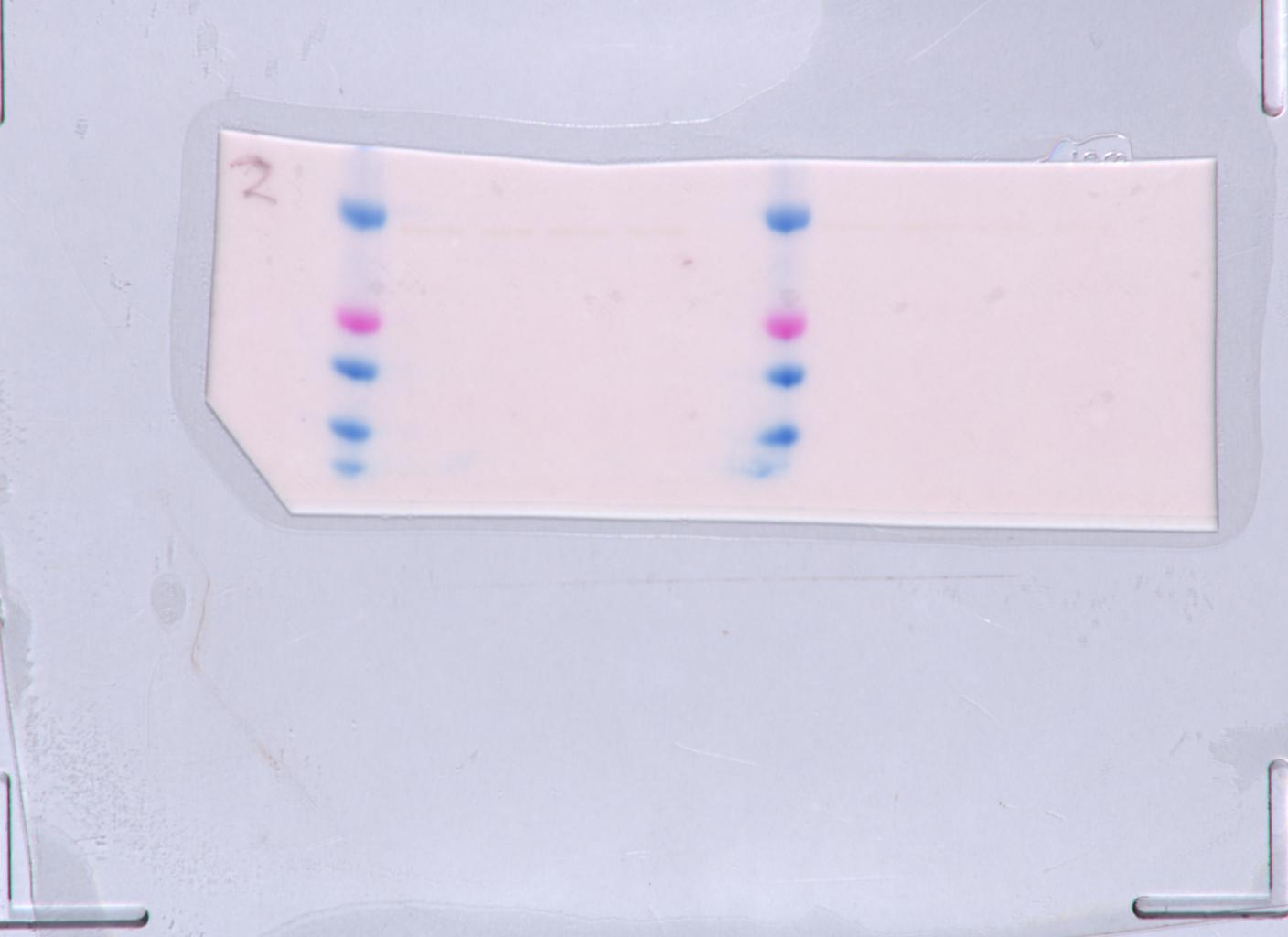

Supplement: Supplementary file 1 [file cancers-16-00370-s001.zip › GAPDH2 cscc2 31-3-22 2022.03.31_15.59.19_Ch-Marker.jpg]

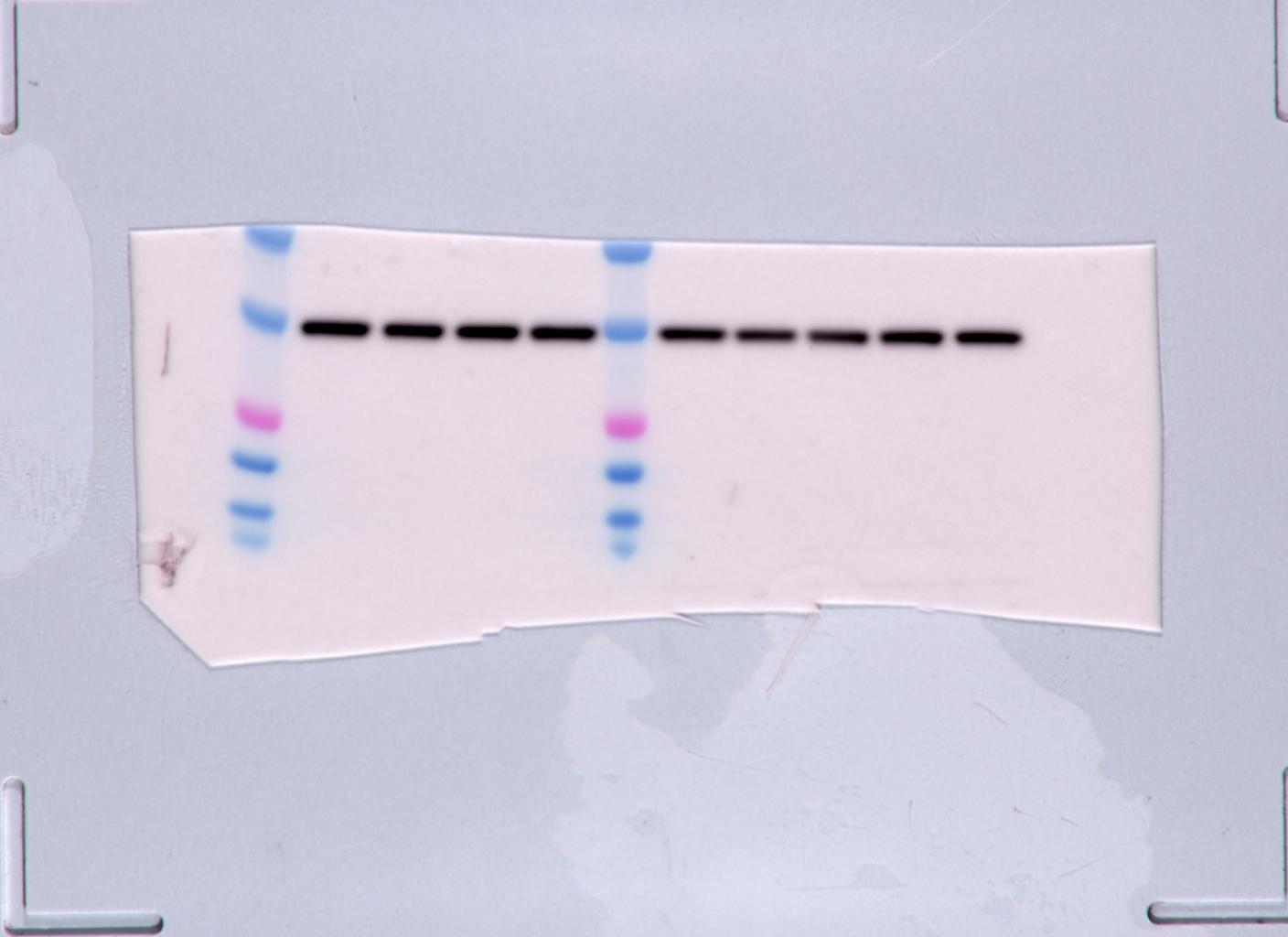

Supplement: Supplementary file 1 [file cancers-16-00370-s001.zip › JP ACDC GEL1 GAPDH 2022.03.28_15.04.24_Ch+Marker.jpg]

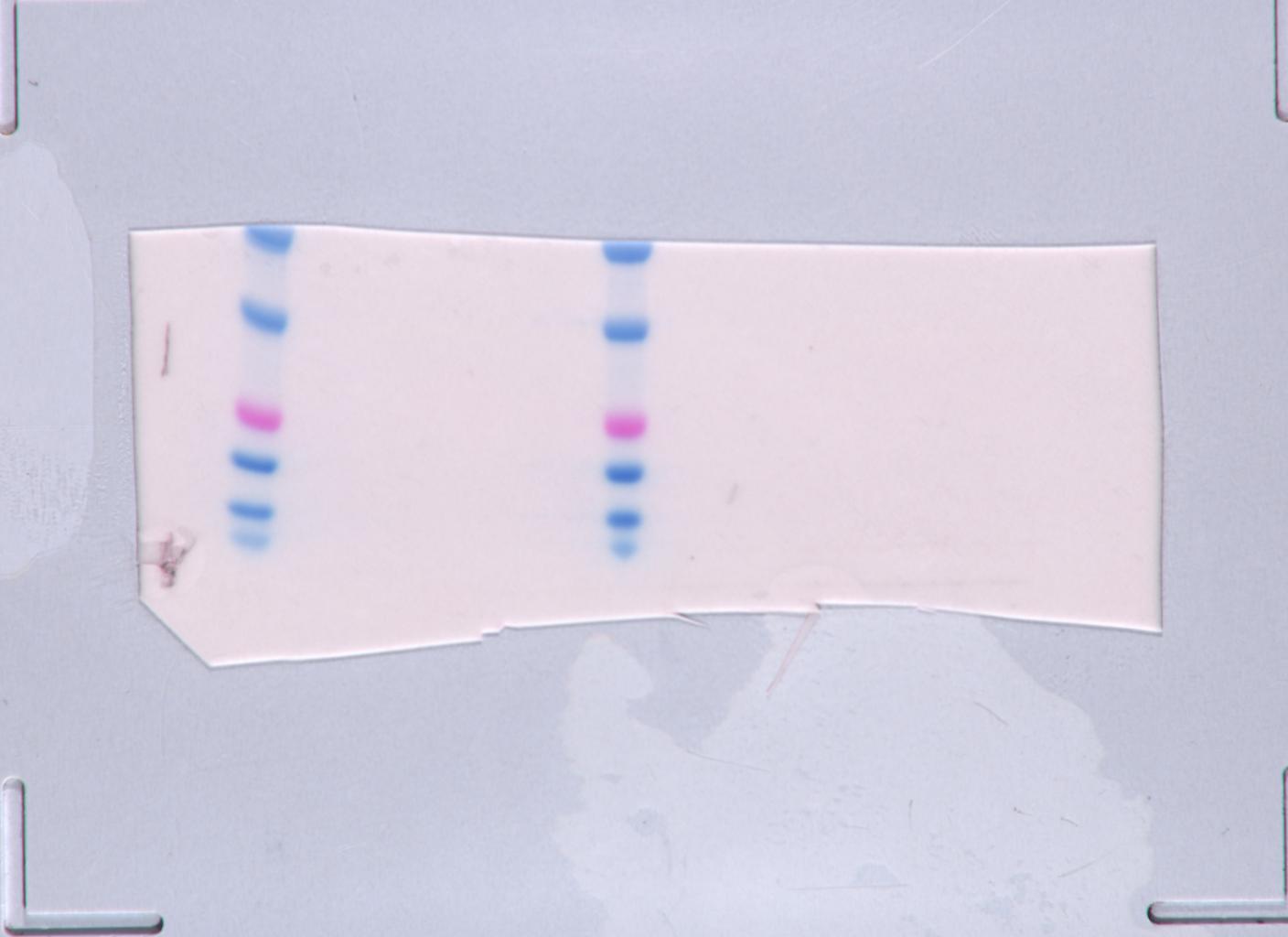

Supplement: Supplementary file 1 [file cancers-16-00370-s001.zip › JP ACDC GEL1 GAPDH 2022.03.28_15.04.24_Ch-Marker.jpg]

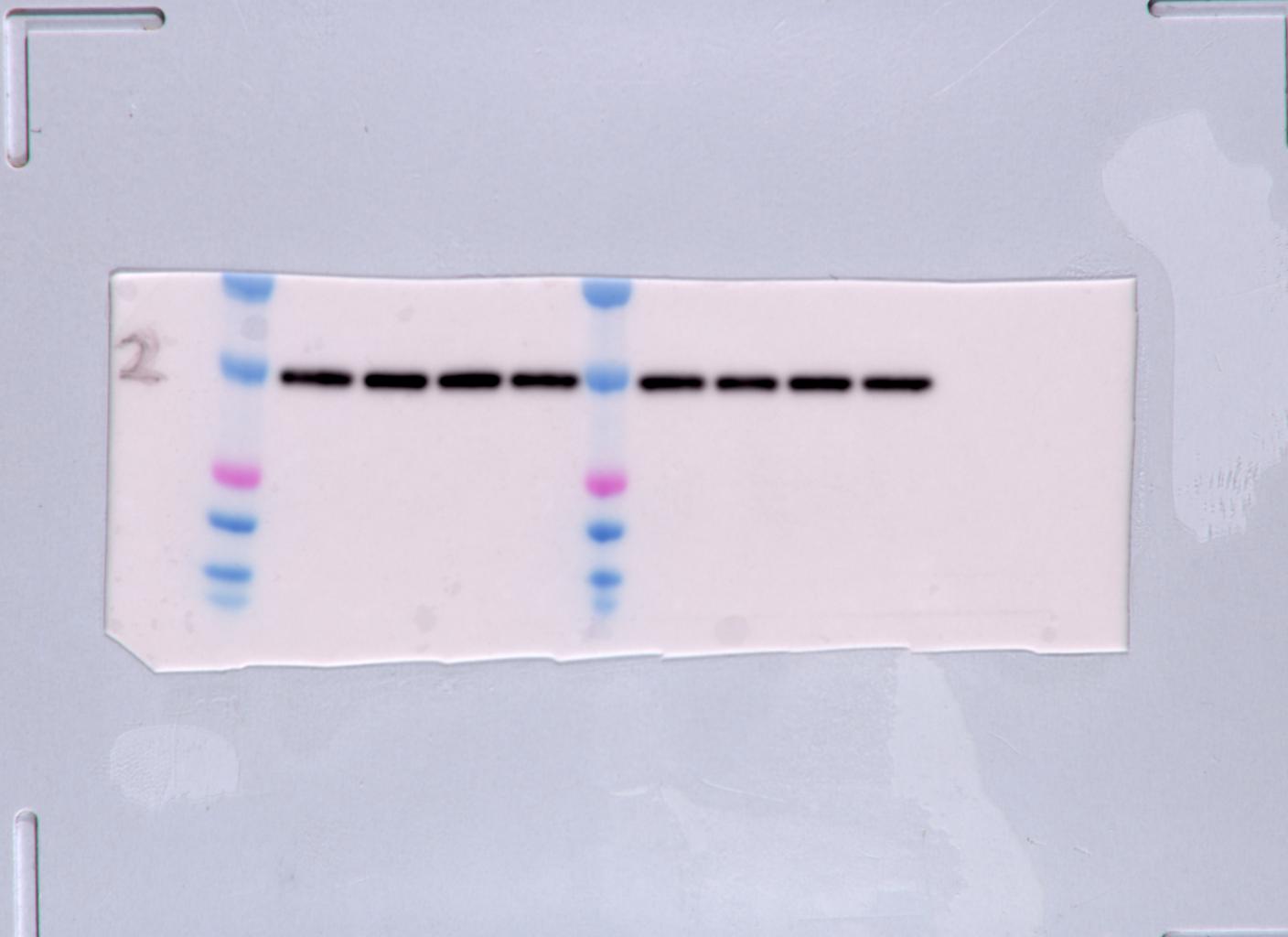

Supplement: Supplementary file 1 [file cancers-16-00370-s001.zip › JP ACDC GEL2 GAPDH 2022.03.28_15.07.00_Ch+Marker.jpg]

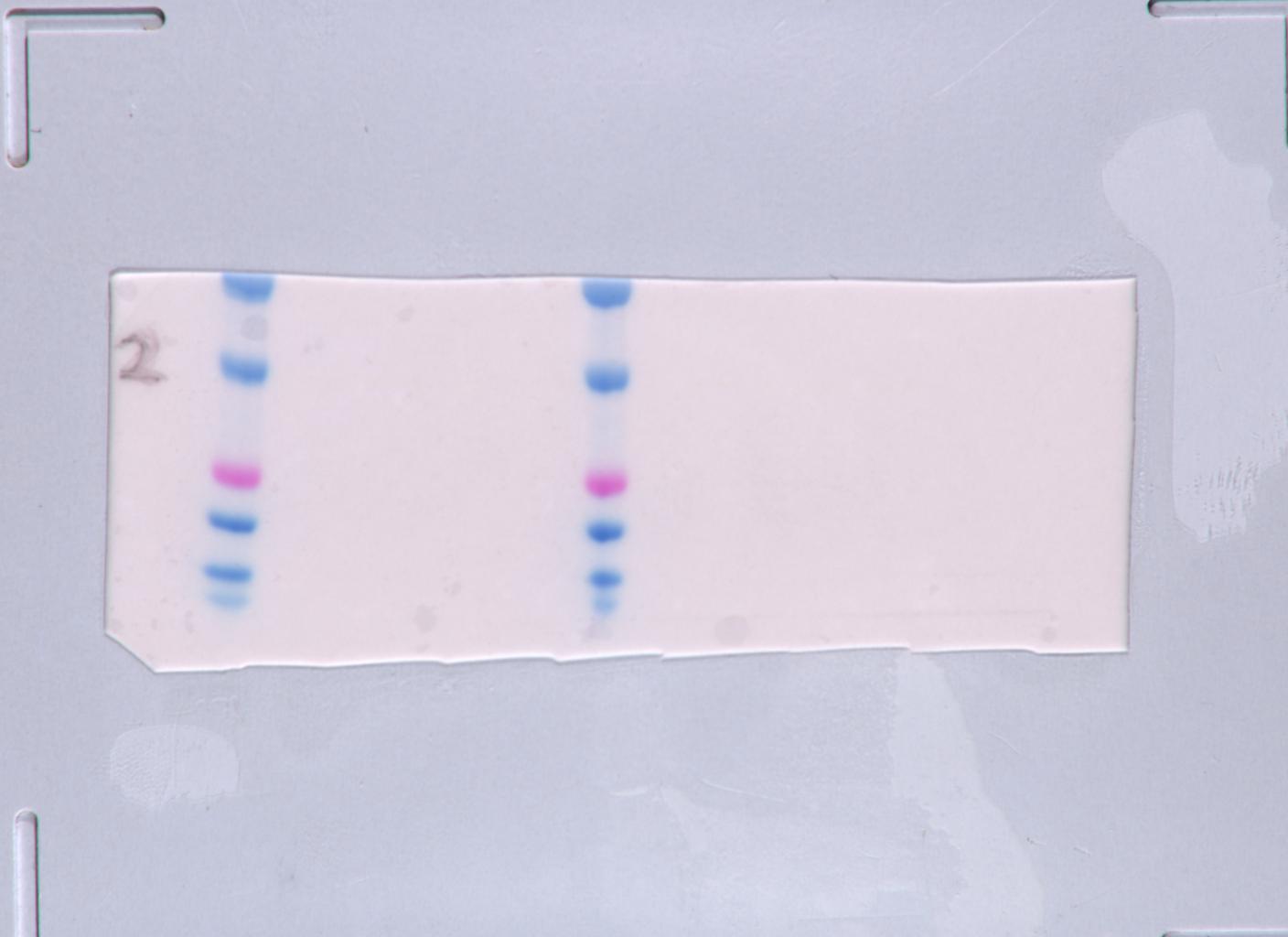

Supplement: Supplementary file 1 [file cancers-16-00370-s001.zip › JP ACDC GEL2 GAPDH 2022.03.28_15.07.00_Ch-Marker.jpg]

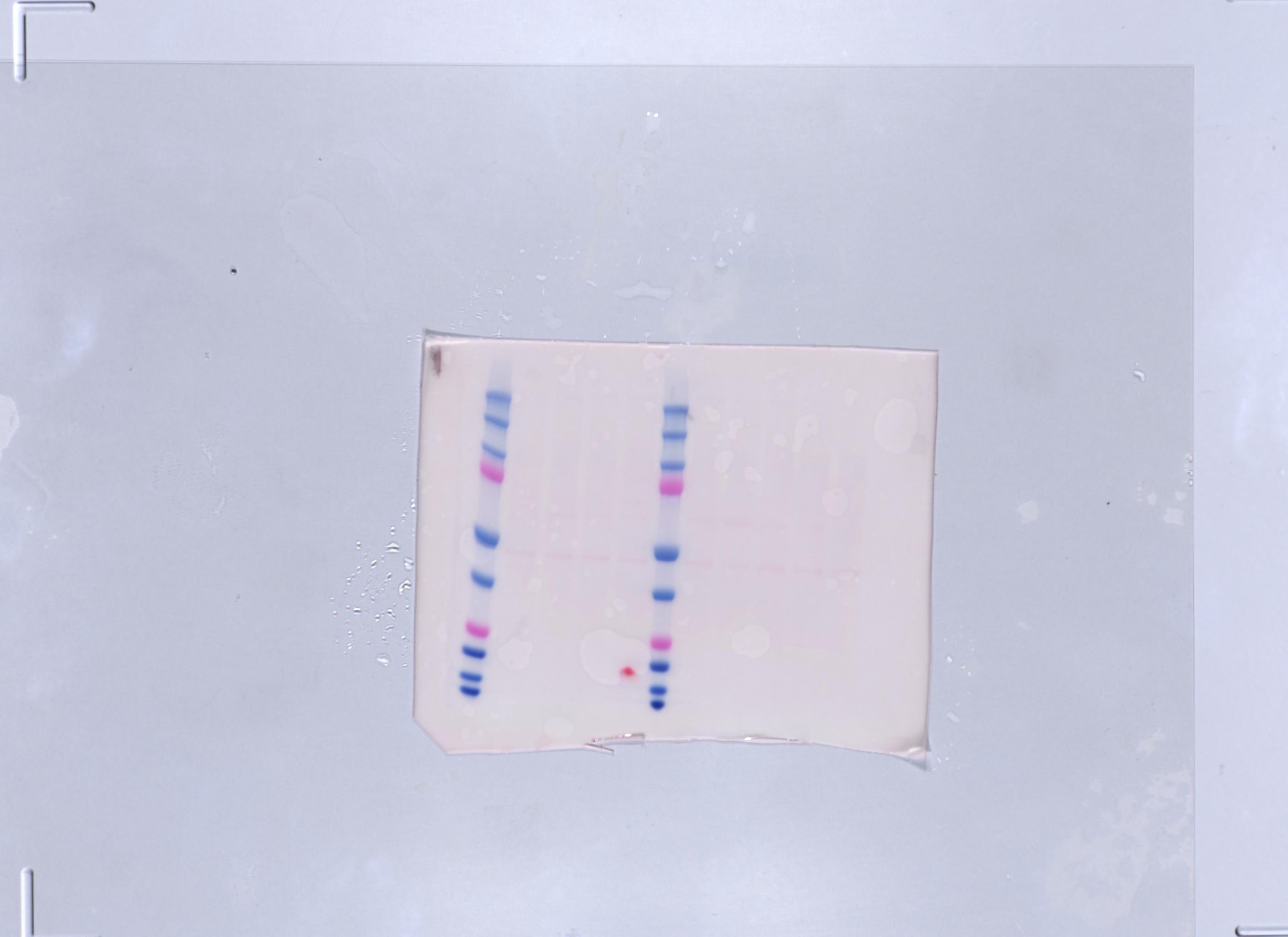

Supplement: Supplementary file 1 [file cancers-16-00370-s001.zip › JP Combo gel1 poncea 2022.03.24_14.47.08_Co.jpg]

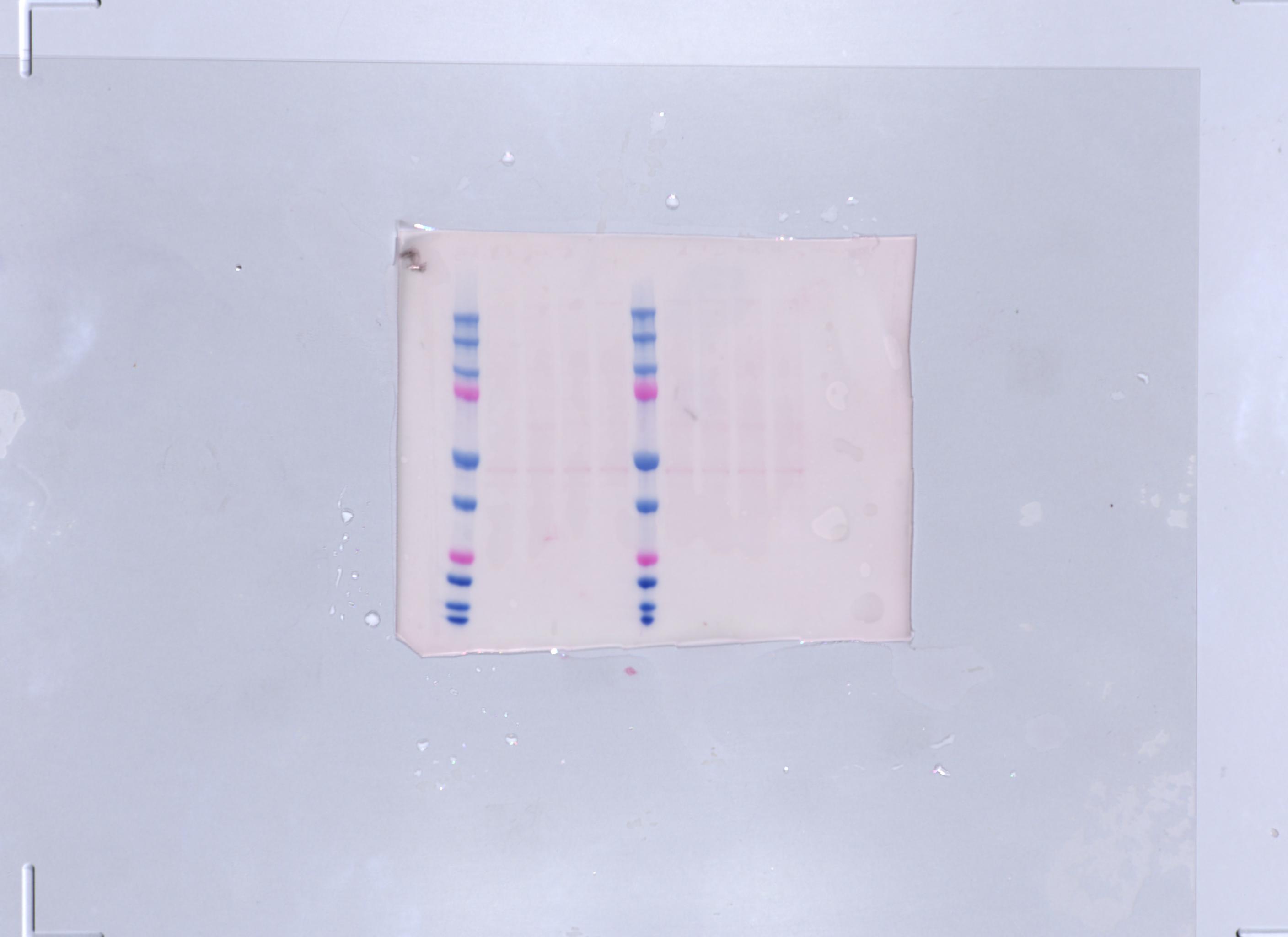

Supplement: Supplementary file 1 [file cancers-16-00370-s001.zip › JP Combo gel2 poncea 2022.03.24_14.48.53_Co.jpg]

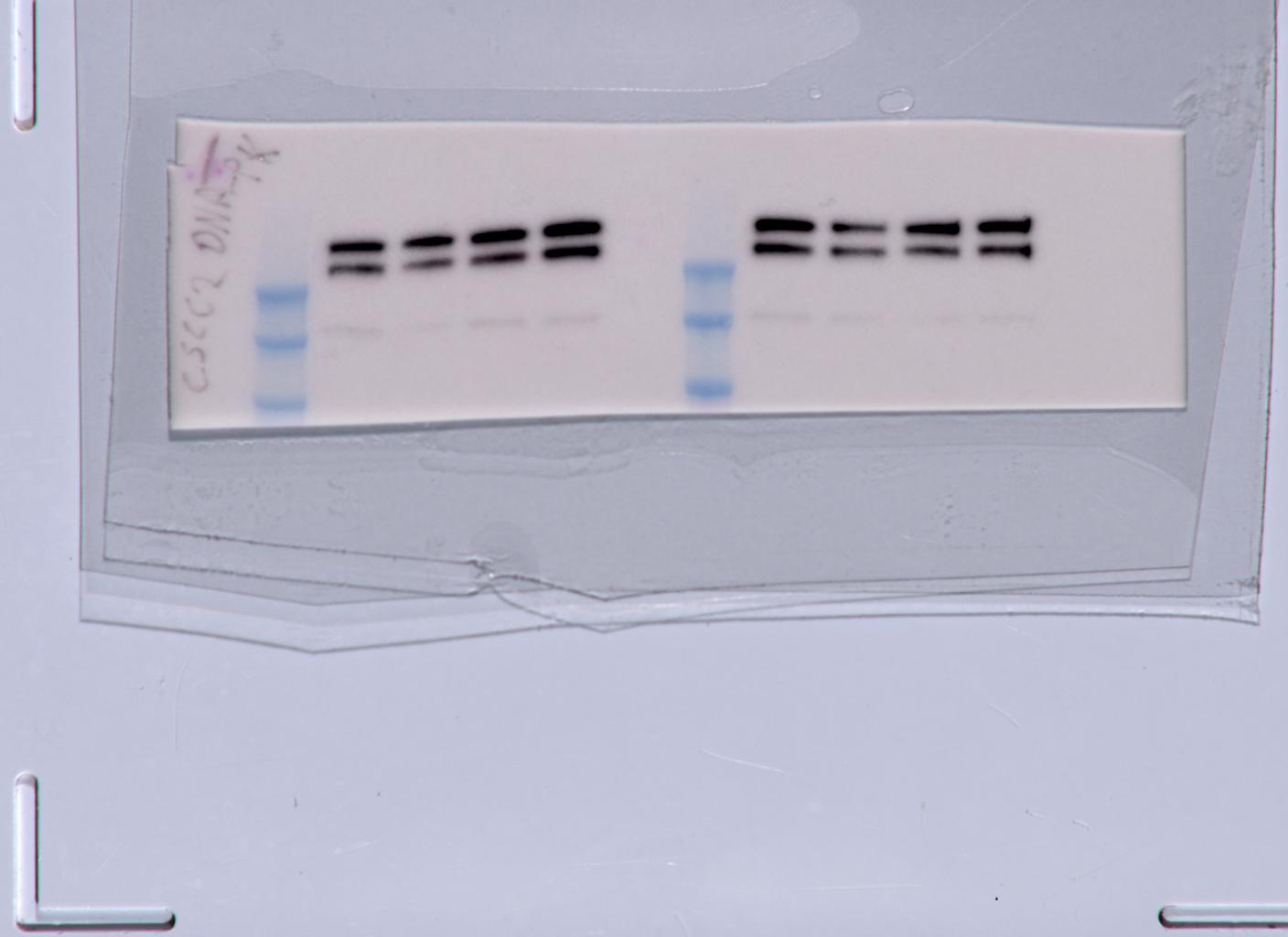

Supplement: Supplementary file 1 [file cancers-16-00370-s001.zip › JP DNAPK cs2 30-5 3m 2022.05.30_14.21.28_Ch+Marker.jpg]

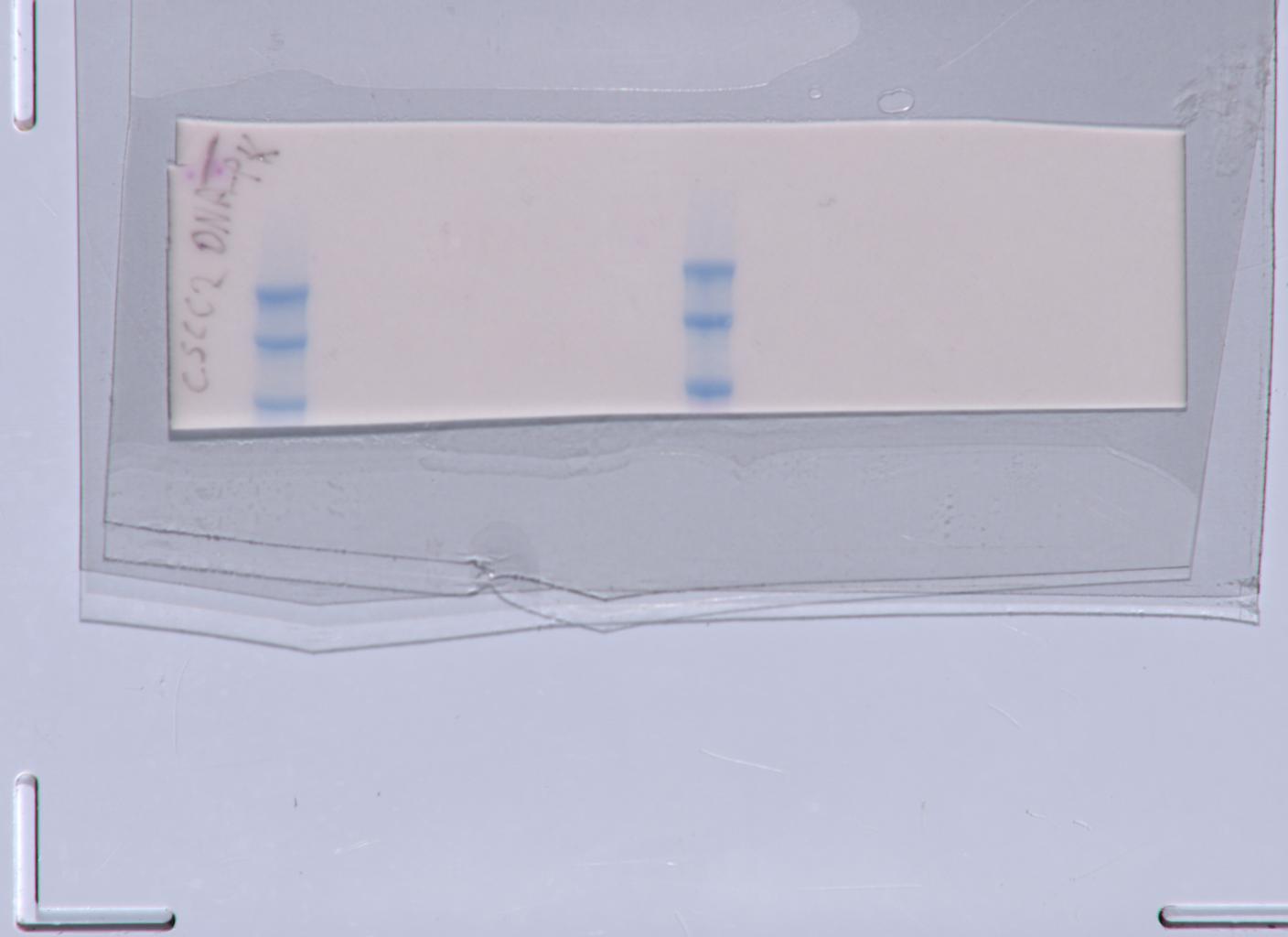

Supplement: Supplementary file 1 [file cancers-16-00370-s001.zip › JP DNAPK cs2 30-5 3m 2022.05.30_14.21.28_Ch-Marker.jpg]

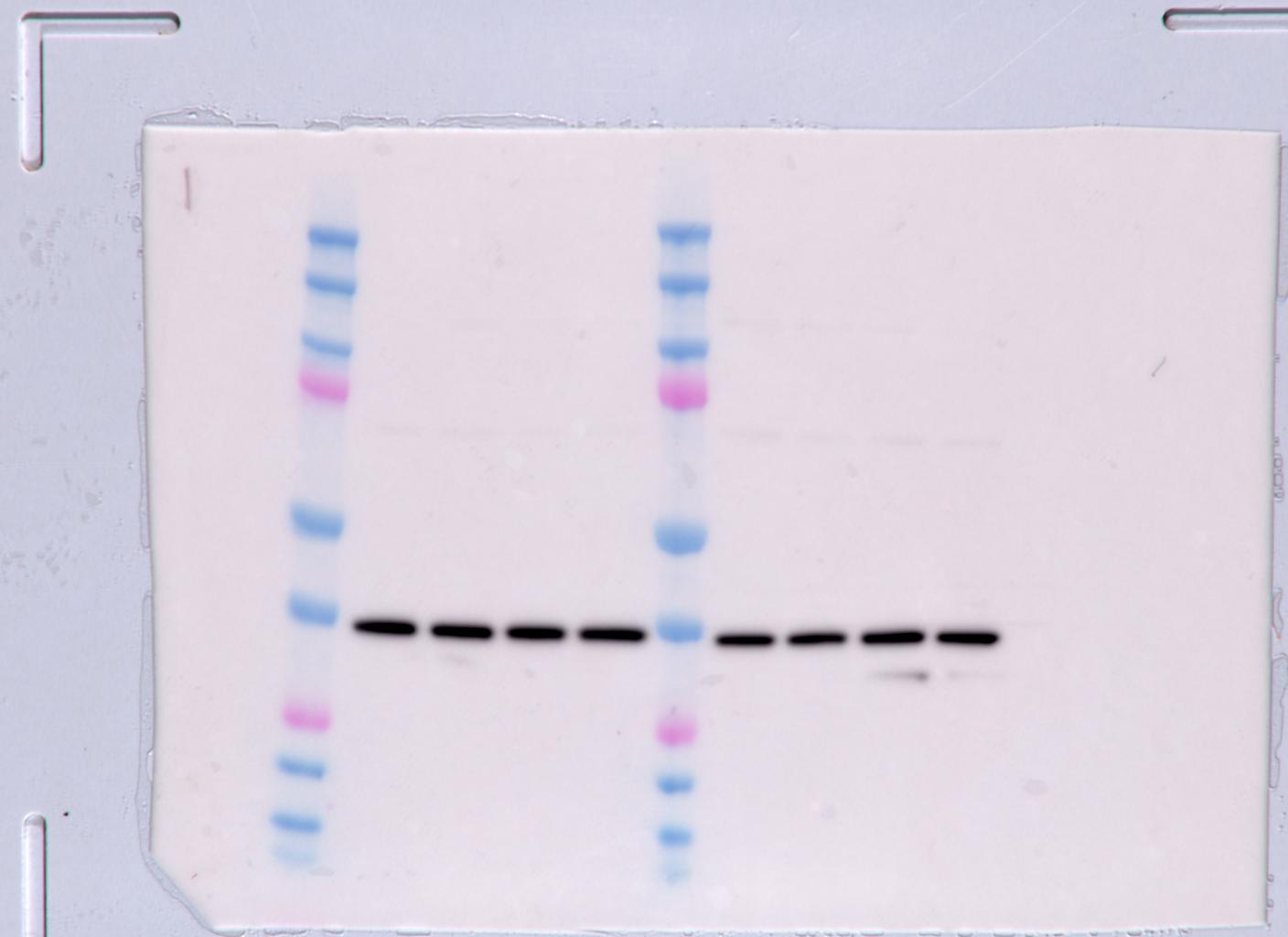

Supplement: Supplementary file 1 [file cancers-16-00370-s001.zip › JP GAPDH1 18-5-22 2022.05.19_12.57.27_Ch+Marker.jpg]

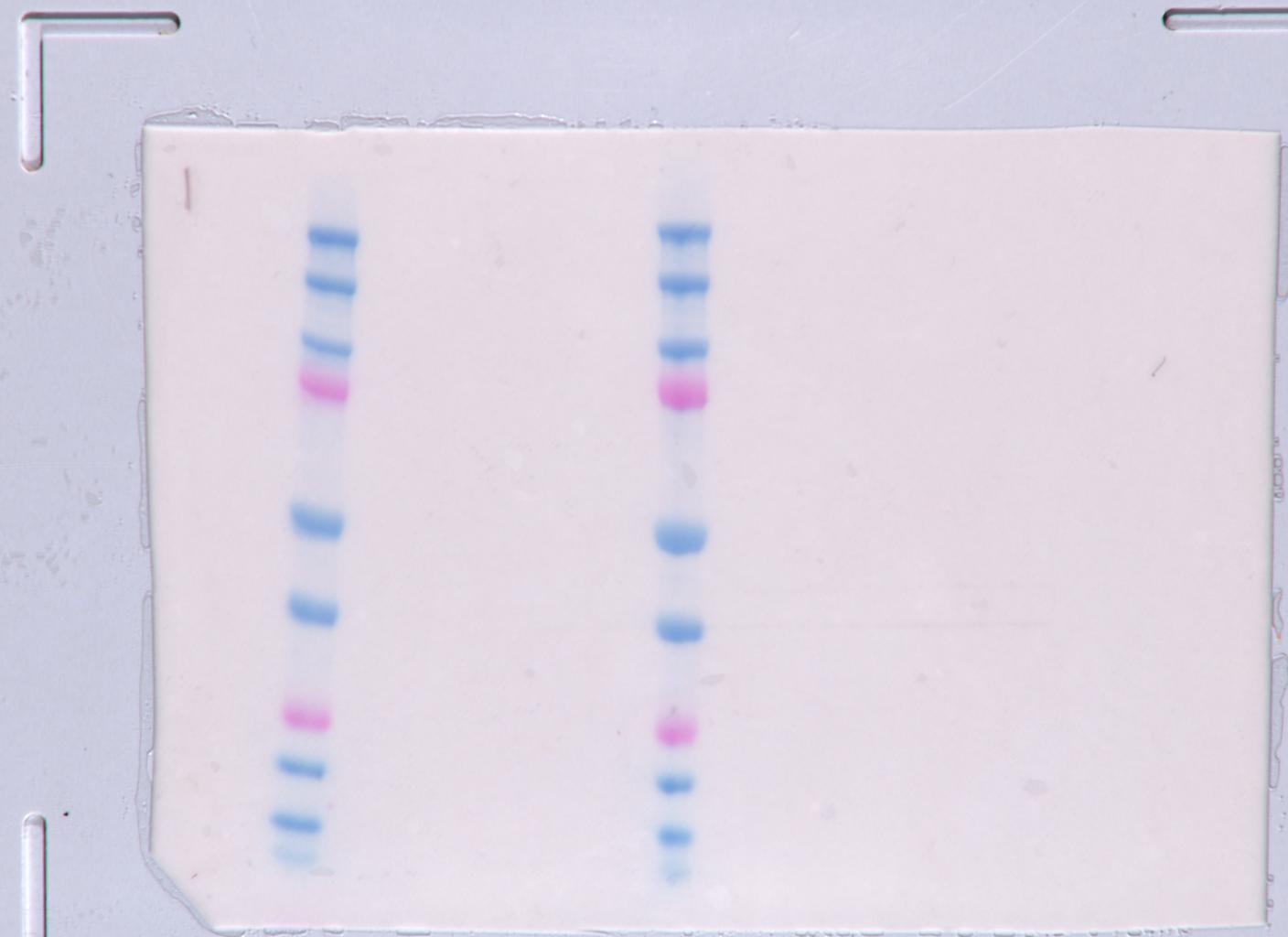

Supplement: Supplementary file 1 [file cancers-16-00370-s001.zip › JP GAPDH1 18-5-22 2022.05.19_12.57.27_Ch-Marker.jpg]

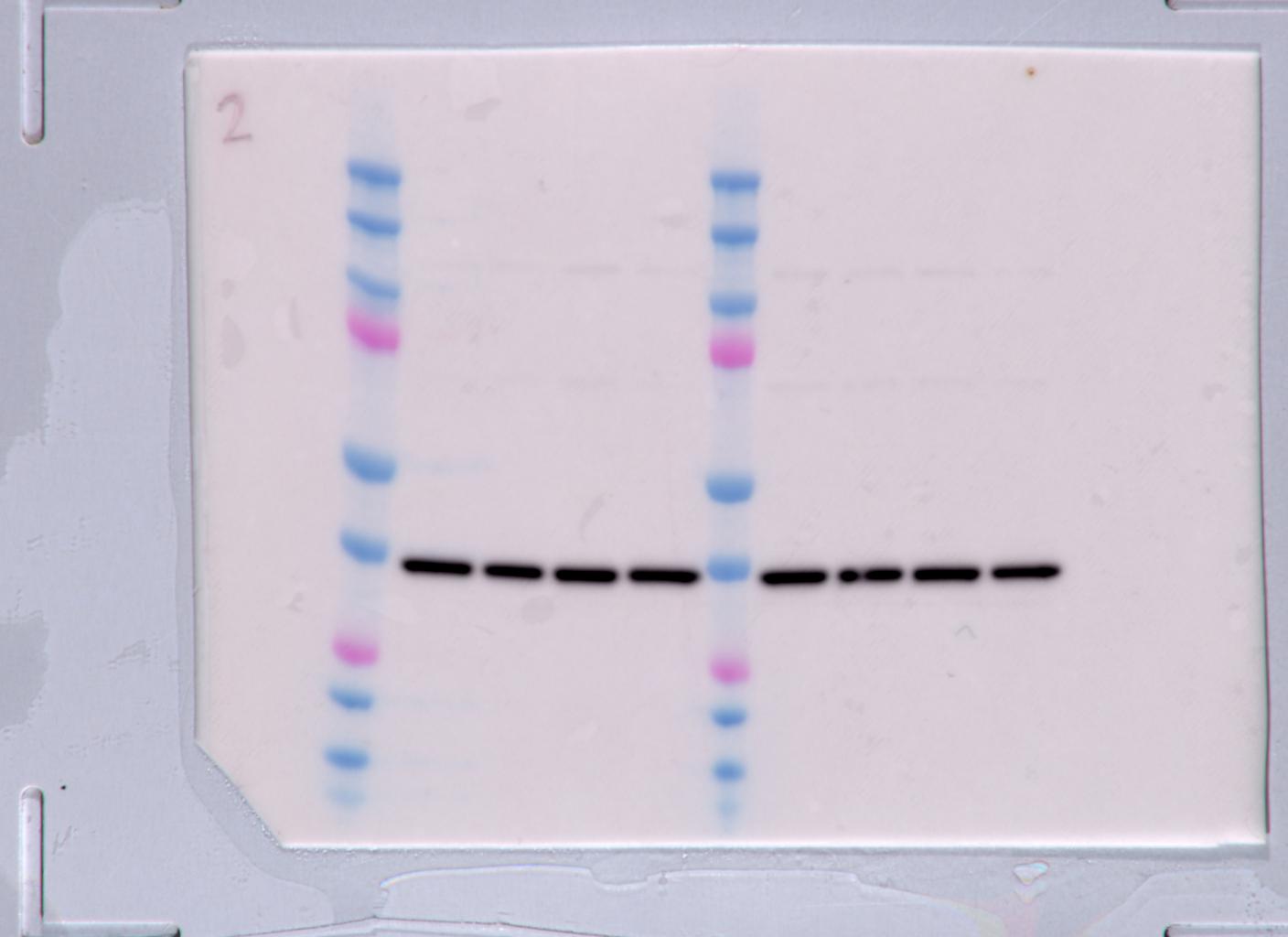

Supplement: Supplementary file 1 [file cancers-16-00370-s001.zip › JP GAPDH2 18-5-22 2022.05.19_13.00.58_Ch+Marker.jpg]

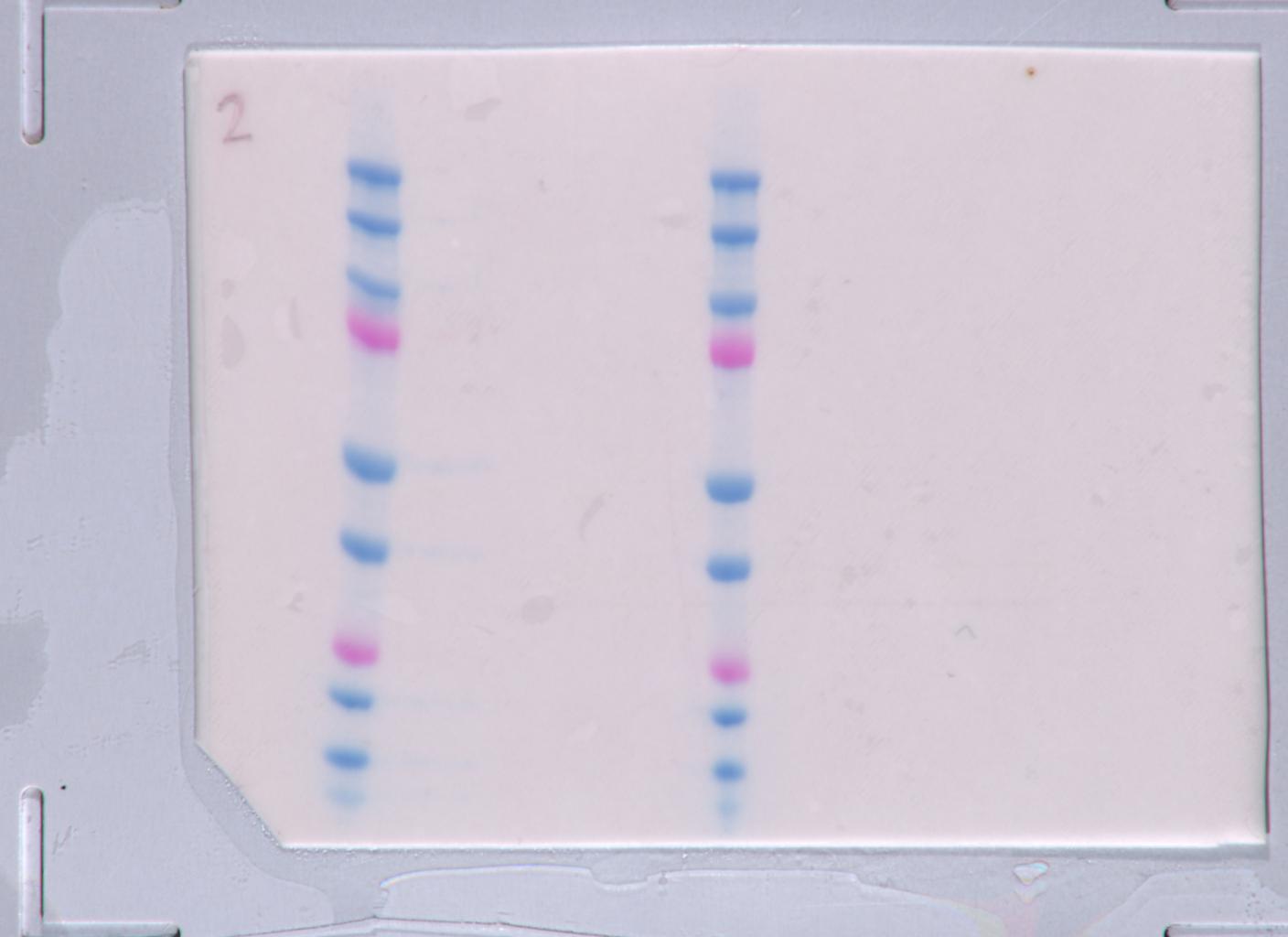

Supplement: Supplementary file 1 [file cancers-16-00370-s001.zip › JP GAPDH2 18-5-22 2022.05.19_13.00.58_Ch-Marker.jpg]

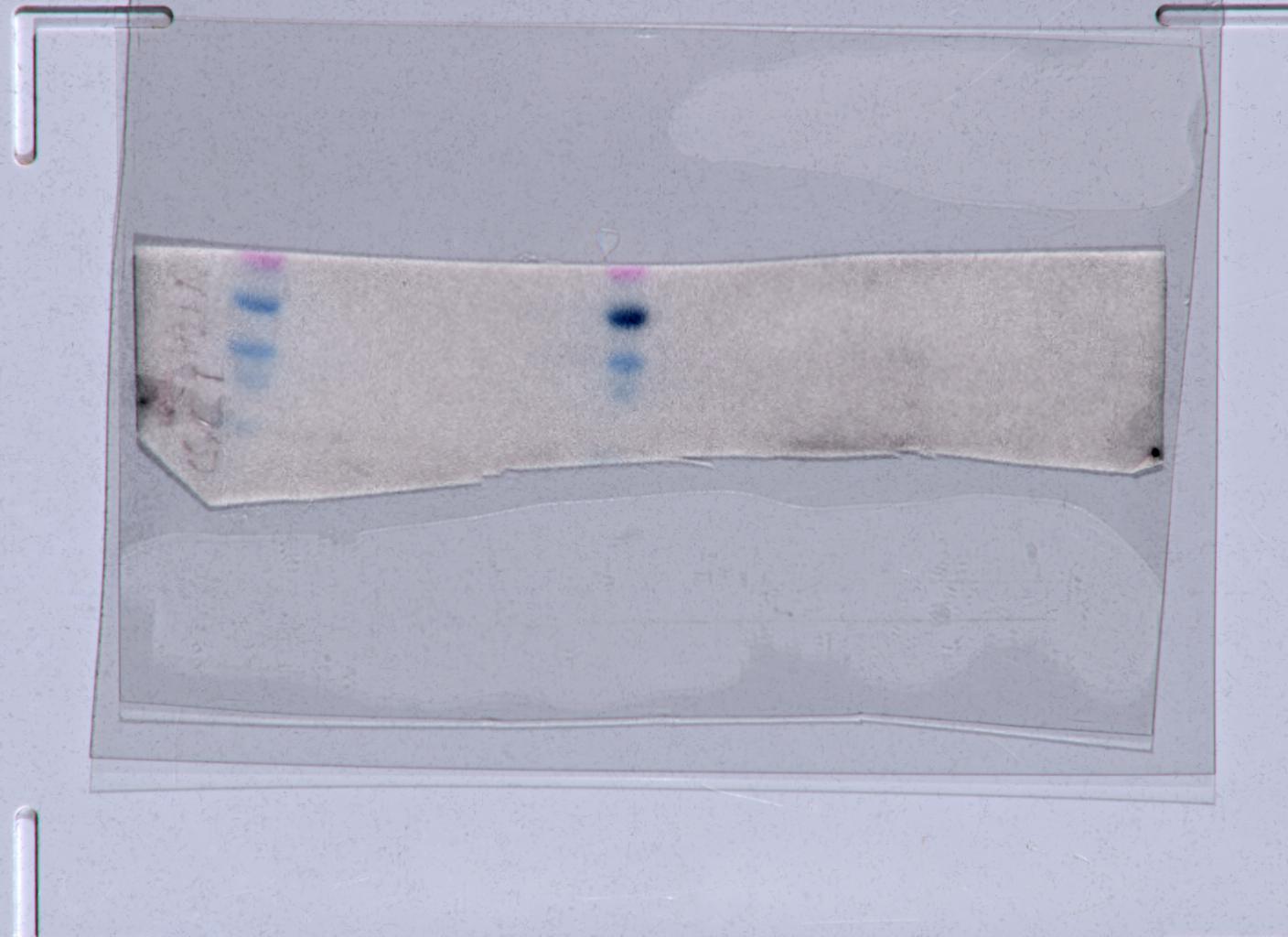

Supplement: Supplementary file 1 [file cancers-16-00370-s001.zip › JP h2ax cs1 30-5 3m 2022.05.30_14.37.34_Ch+Marker.jpg]

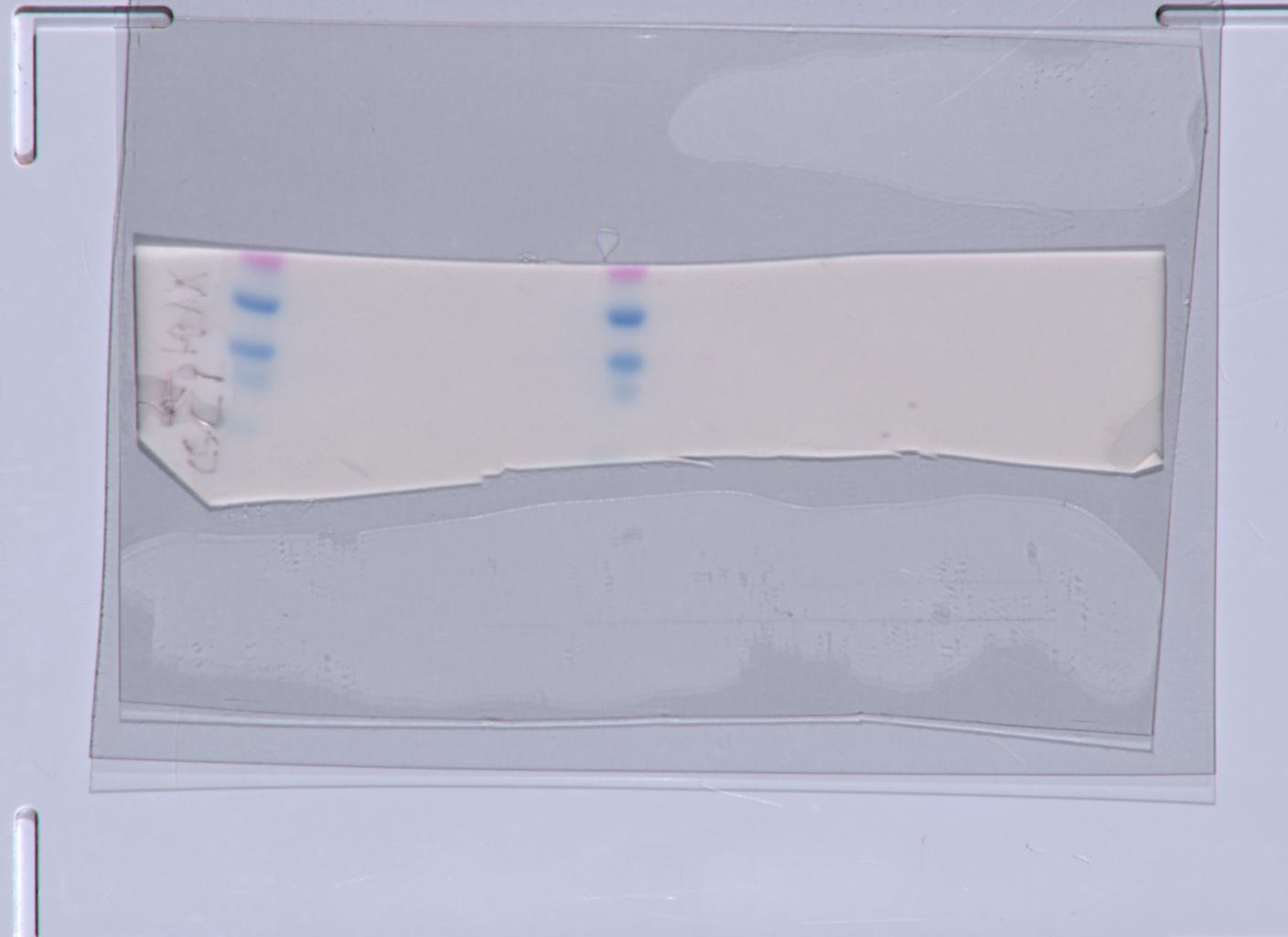

Supplement: Supplementary file 1 [file cancers-16-00370-s001.zip › JP h2ax cs1 30-5 3m 2022.05.30_14.37.34_Ch-Marker.jpg]

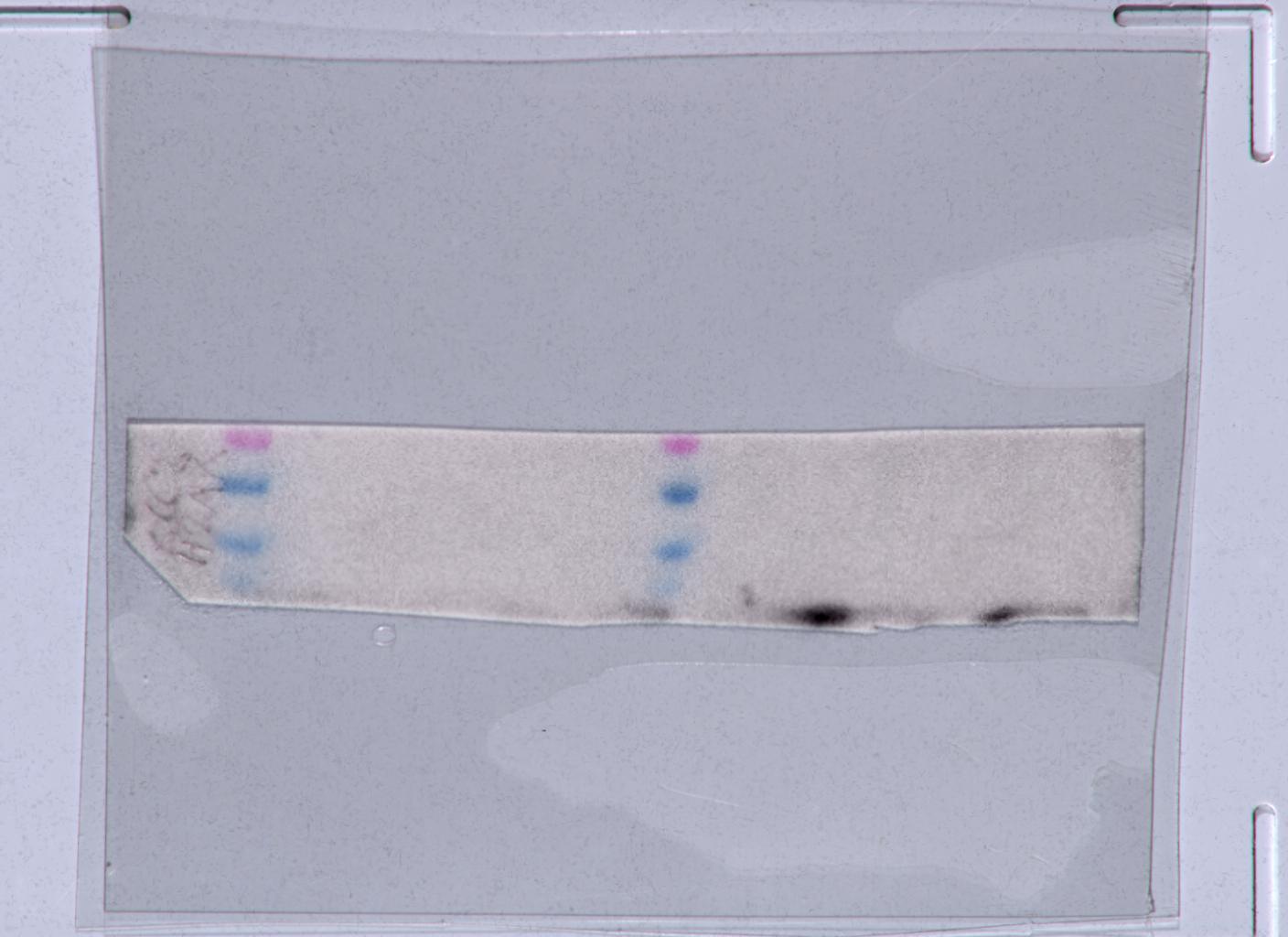

Supplement: Supplementary file 1 [file cancers-16-00370-s001.zip › JP h2ax cs2 30-5 3m 2022.05.30_14.42.20_Ch+Marker.jpg]

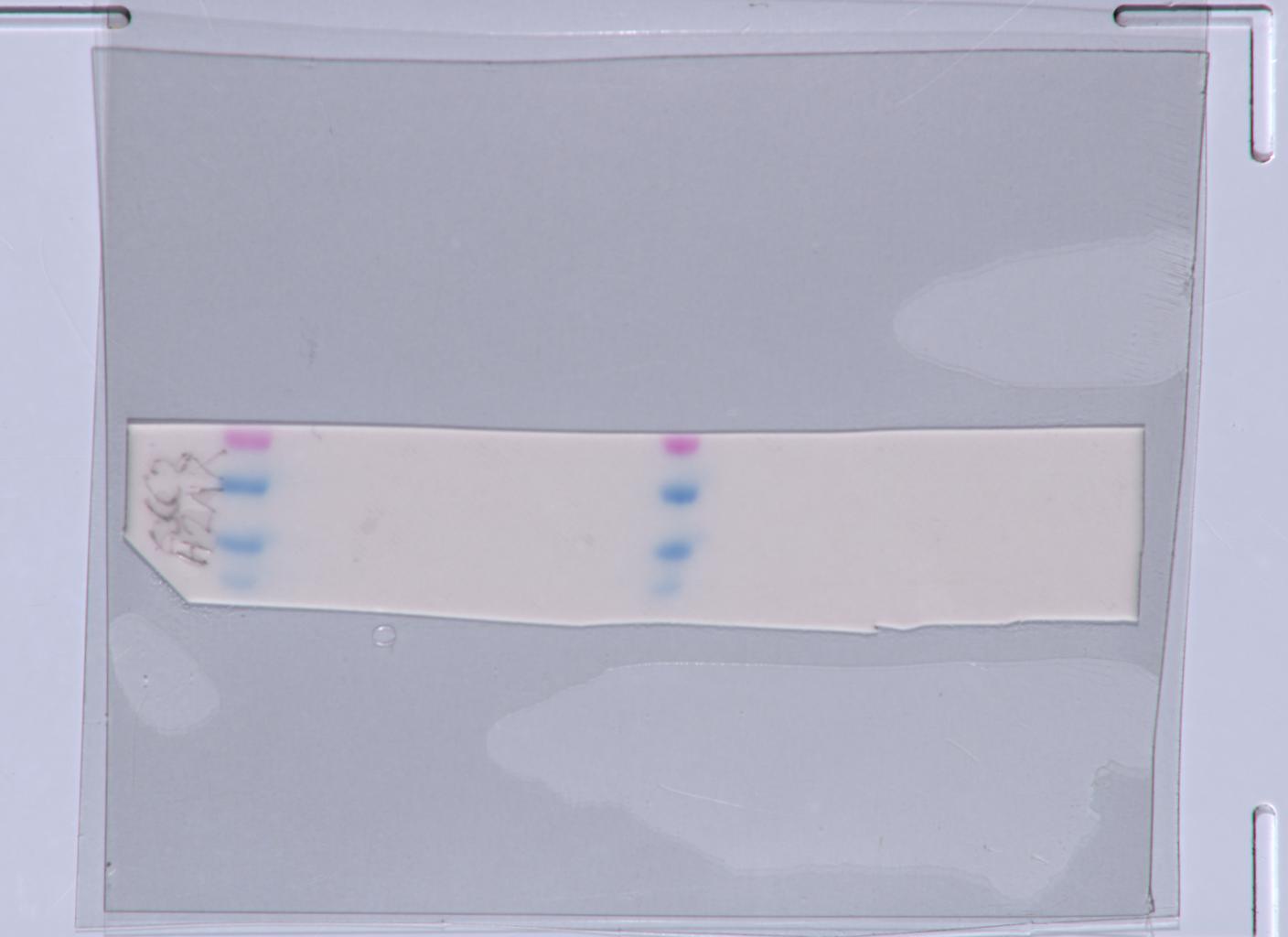

Supplement: Supplementary file 1 [file cancers-16-00370-s001.zip › JP h2ax cs2 30-5 3m 2022.05.30_14.42.20_Ch-Marker.jpg]

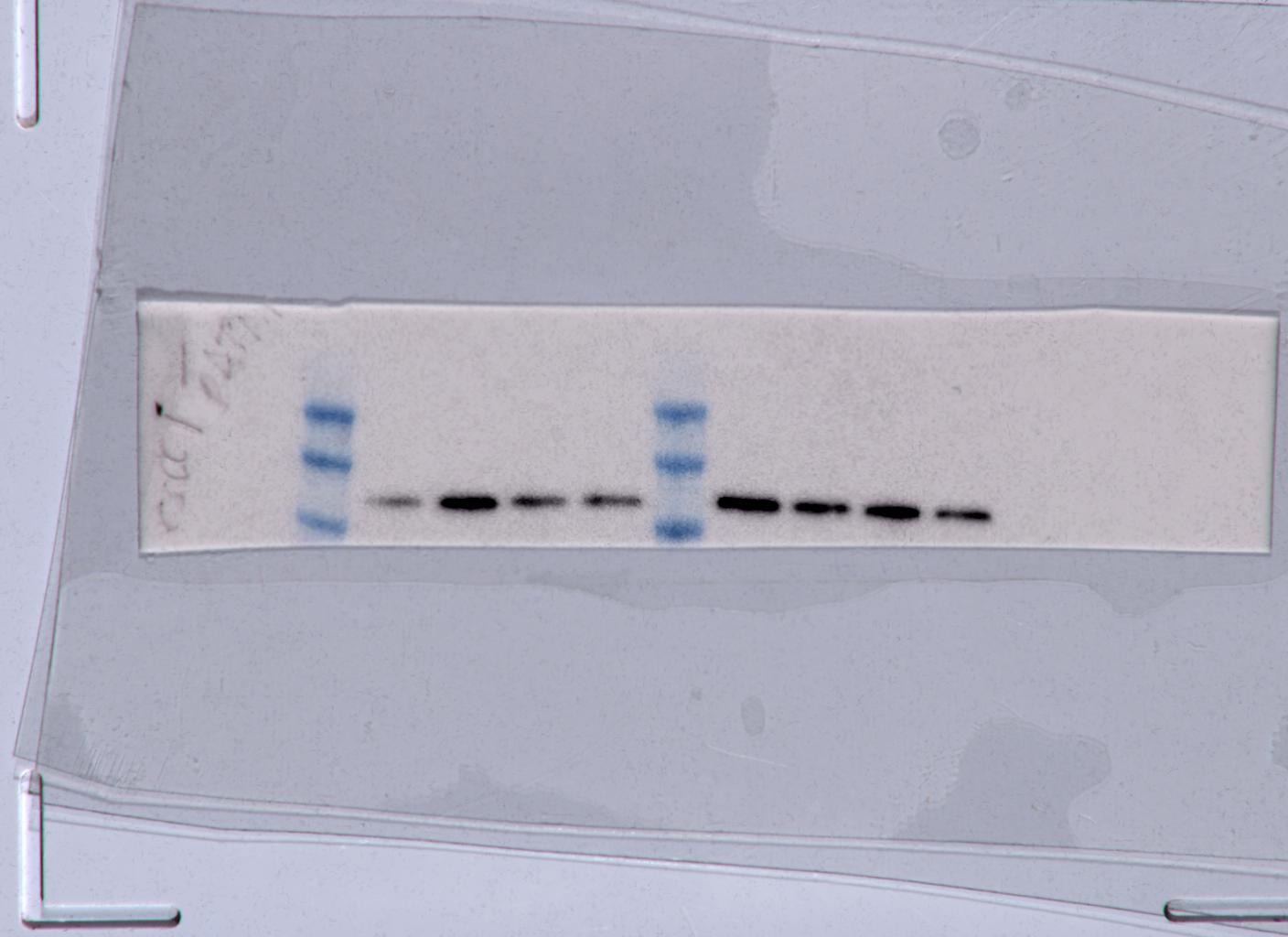

Supplement: Supplementary file 1 [file cancers-16-00370-s001.zip › JP pATM cs1 30-5 2mi 2022.05.30_11.04.45_Ch+Marker.jpg]

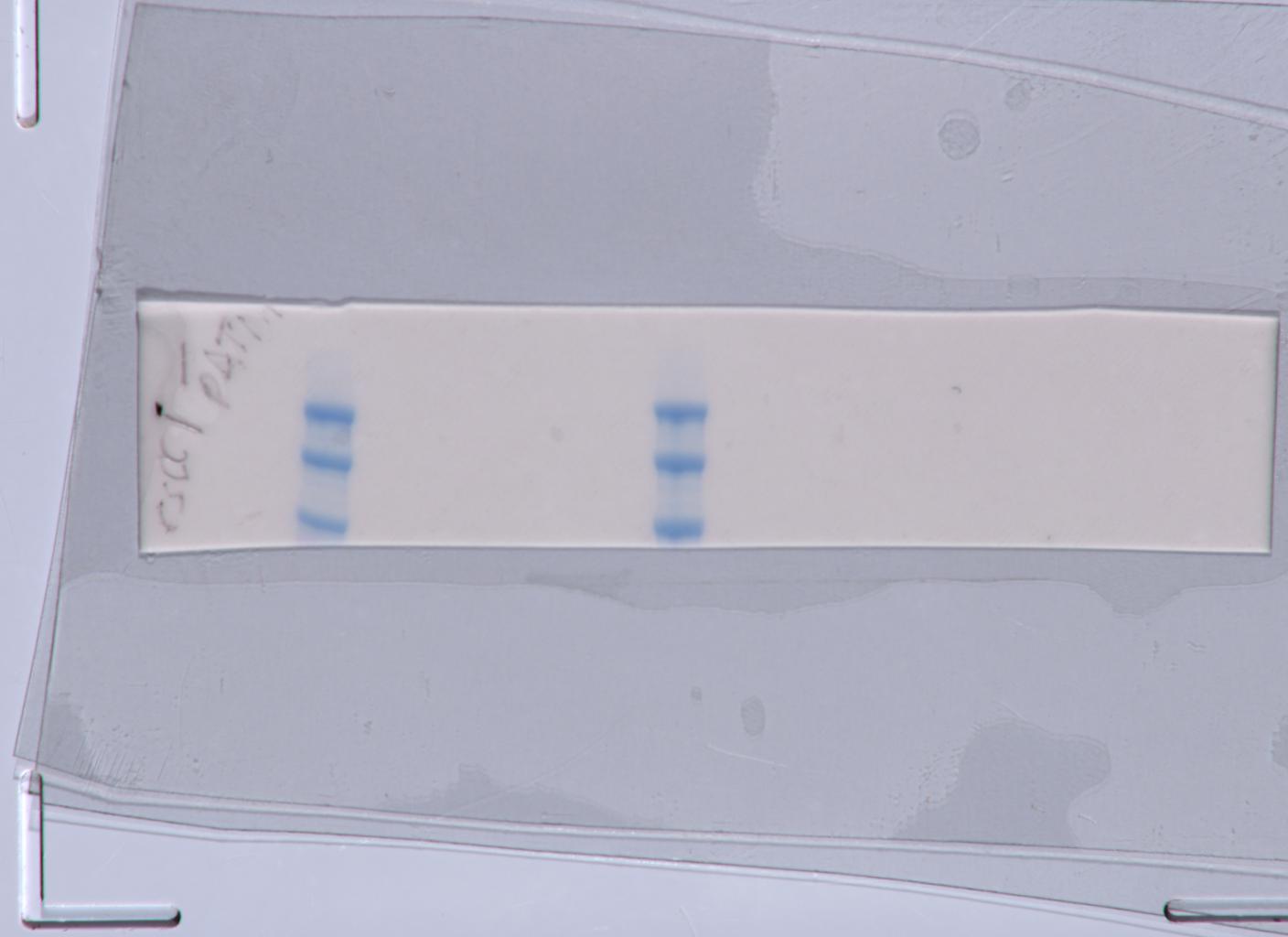

Supplement: Supplementary file 1 [file cancers-16-00370-s001.zip › JP pATM cs1 30-5 2mi 2022.05.30_11.04.45_Ch-Marker.jpg]

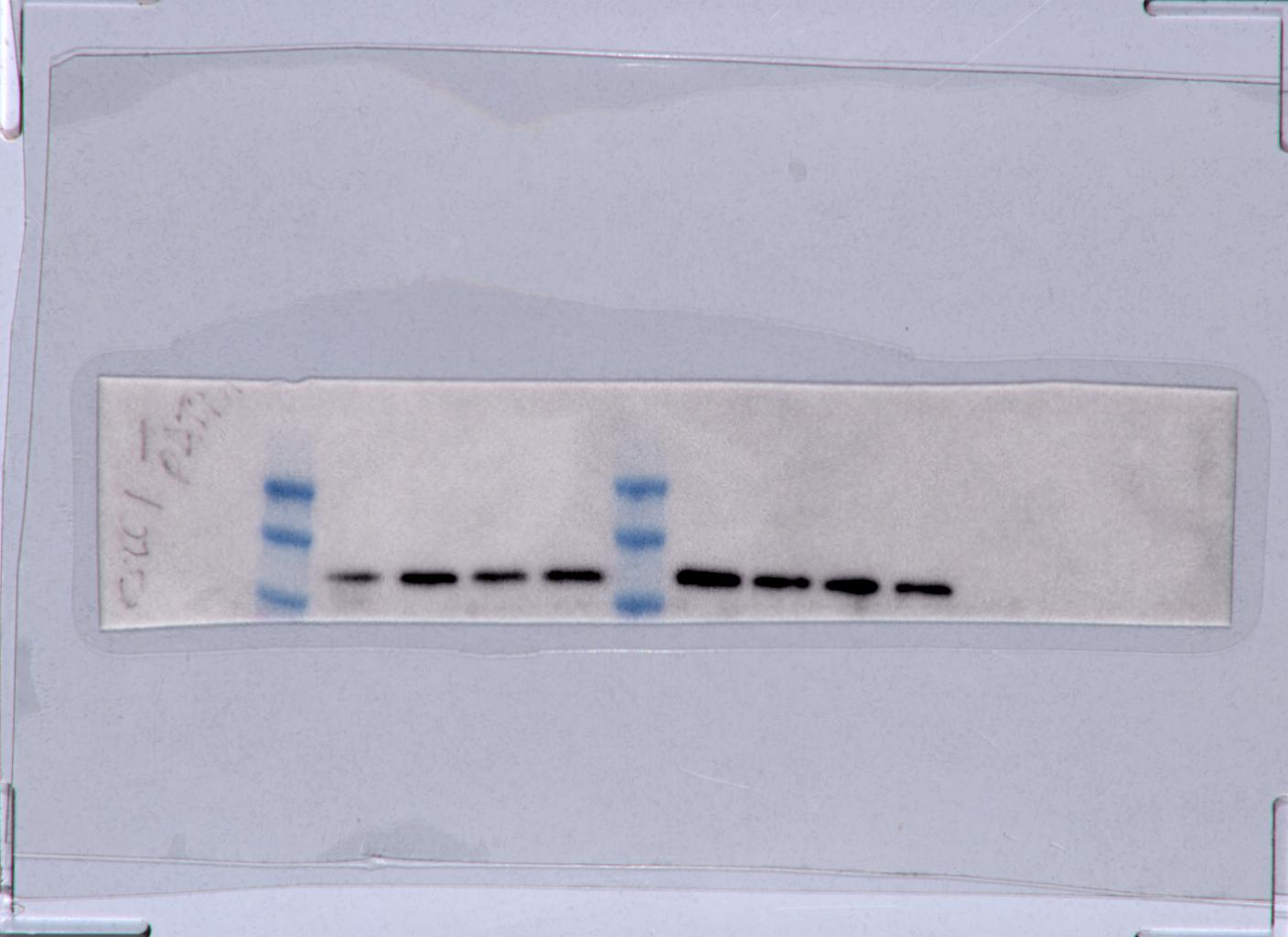

Supplement: Supplementary file 1 [file cancers-16-00370-s001.zip › JP pATM cs1 30-5 3mi 2022.05.30_14.05.44_Ch+Marker.jpg]

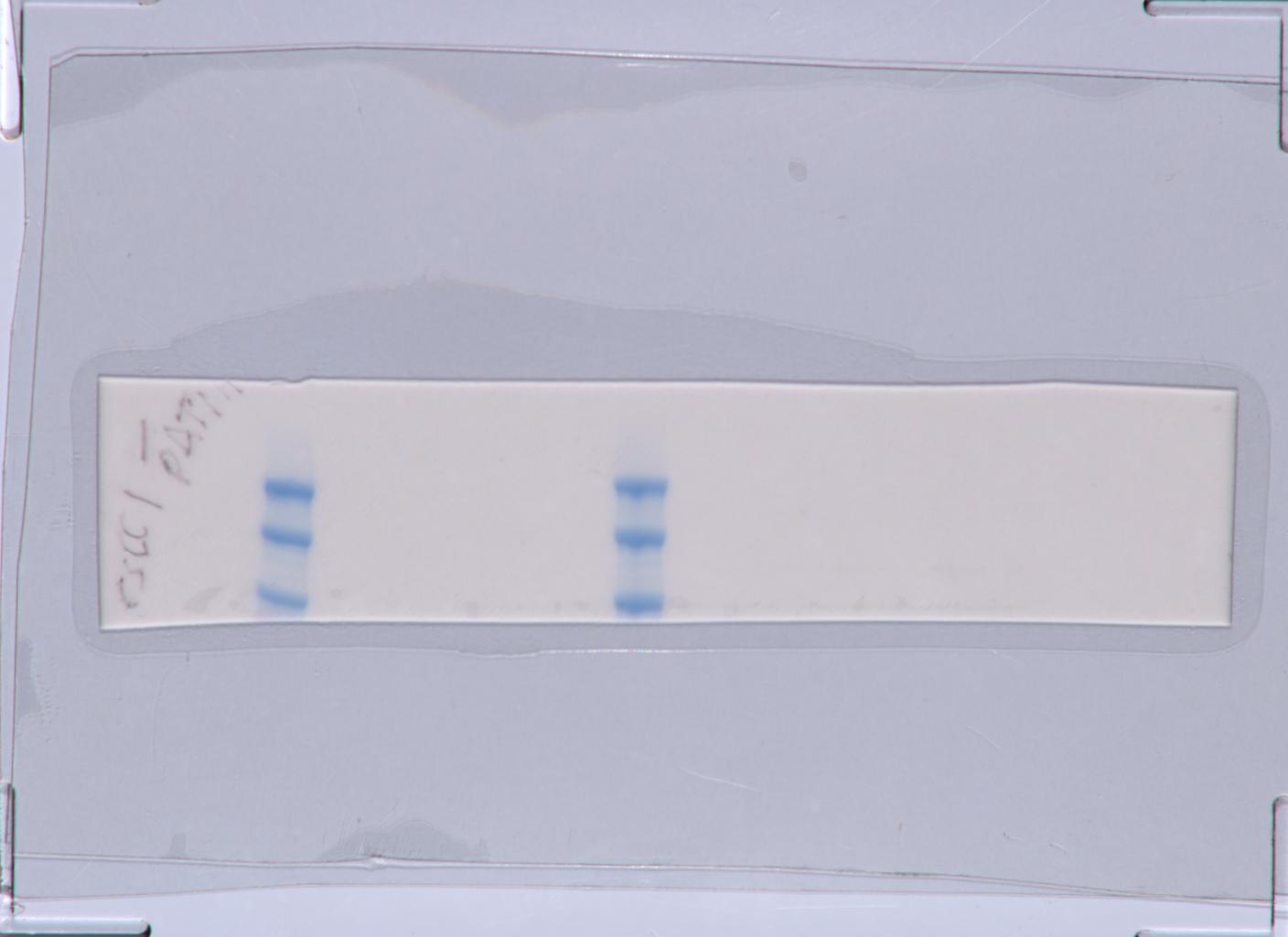

Supplement: Supplementary file 1 [file cancers-16-00370-s001.zip › JP pATM cs1 30-5 3mi 2022.05.30_14.05.44_Ch-Marker.jpg]

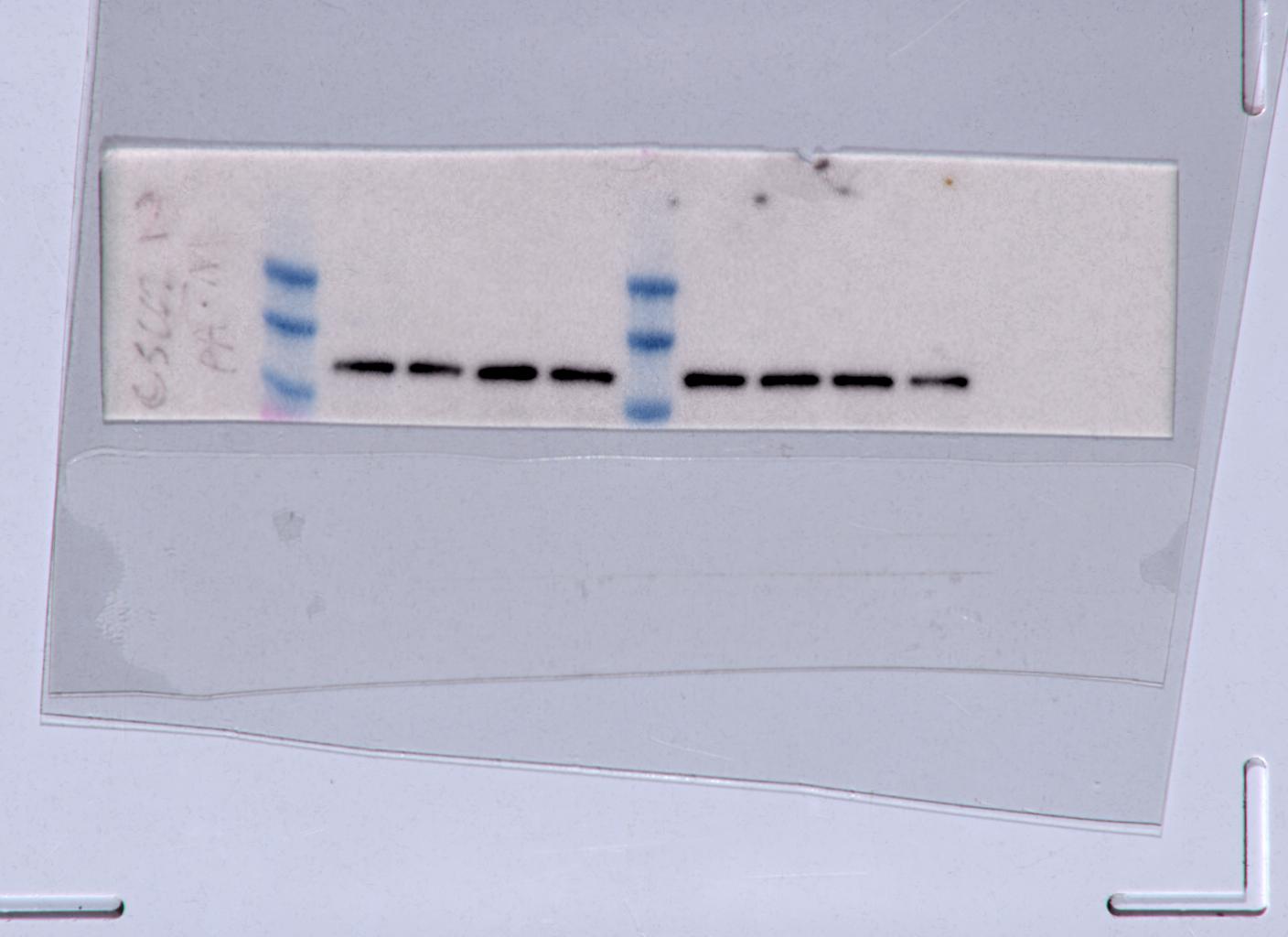

Supplement: Supplementary file 1 [file cancers-16-00370-s001.zip › JP pATM cs2 30-5 2mi 2022.05.30_11.08.17_Ch+Marker.jpg]

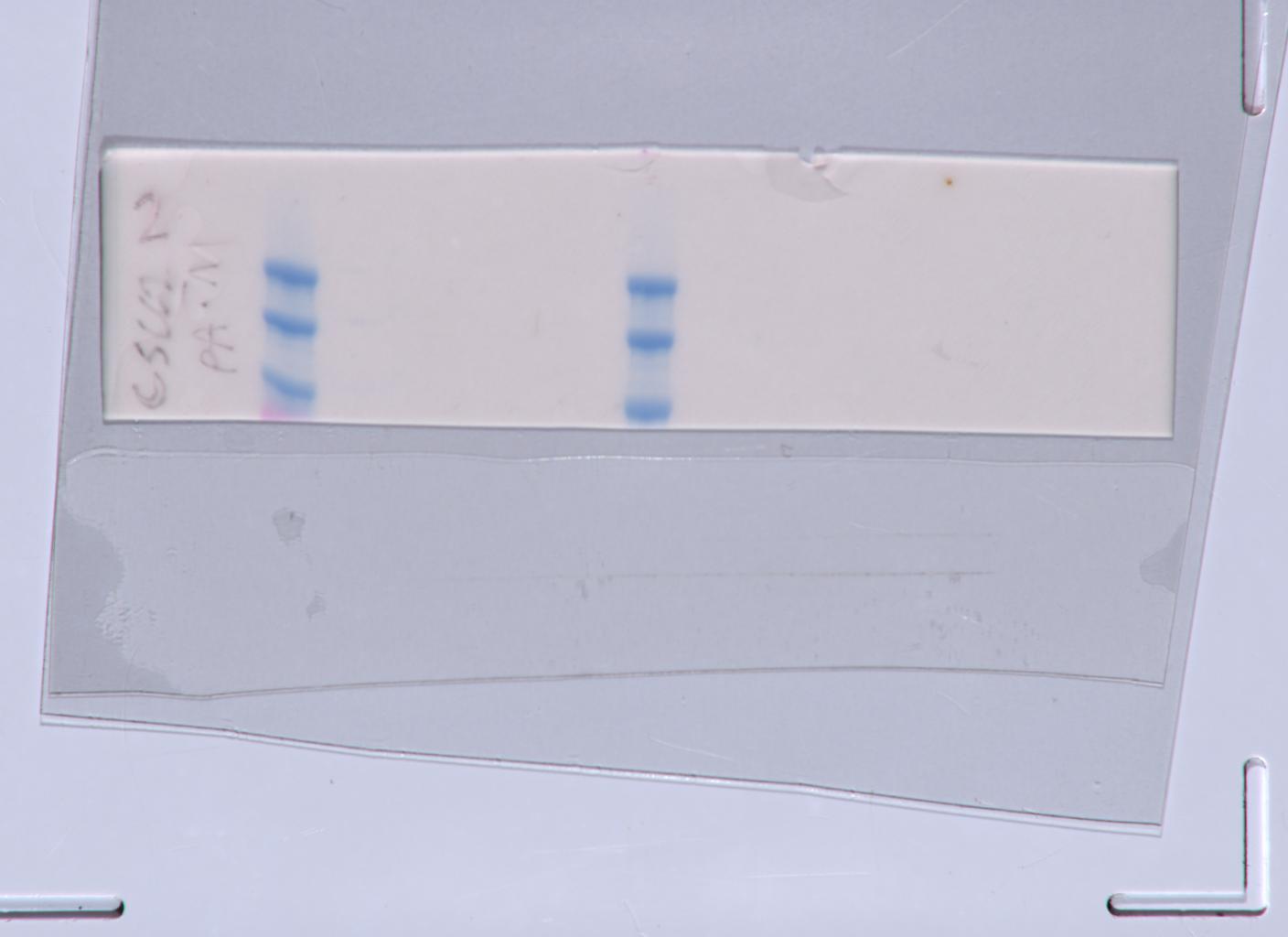

Supplement: Supplementary file 1 [file cancers-16-00370-s001.zip › JP pATM cs2 30-5 2mi 2022.05.30_11.08.17_Ch-Marker.jpg]

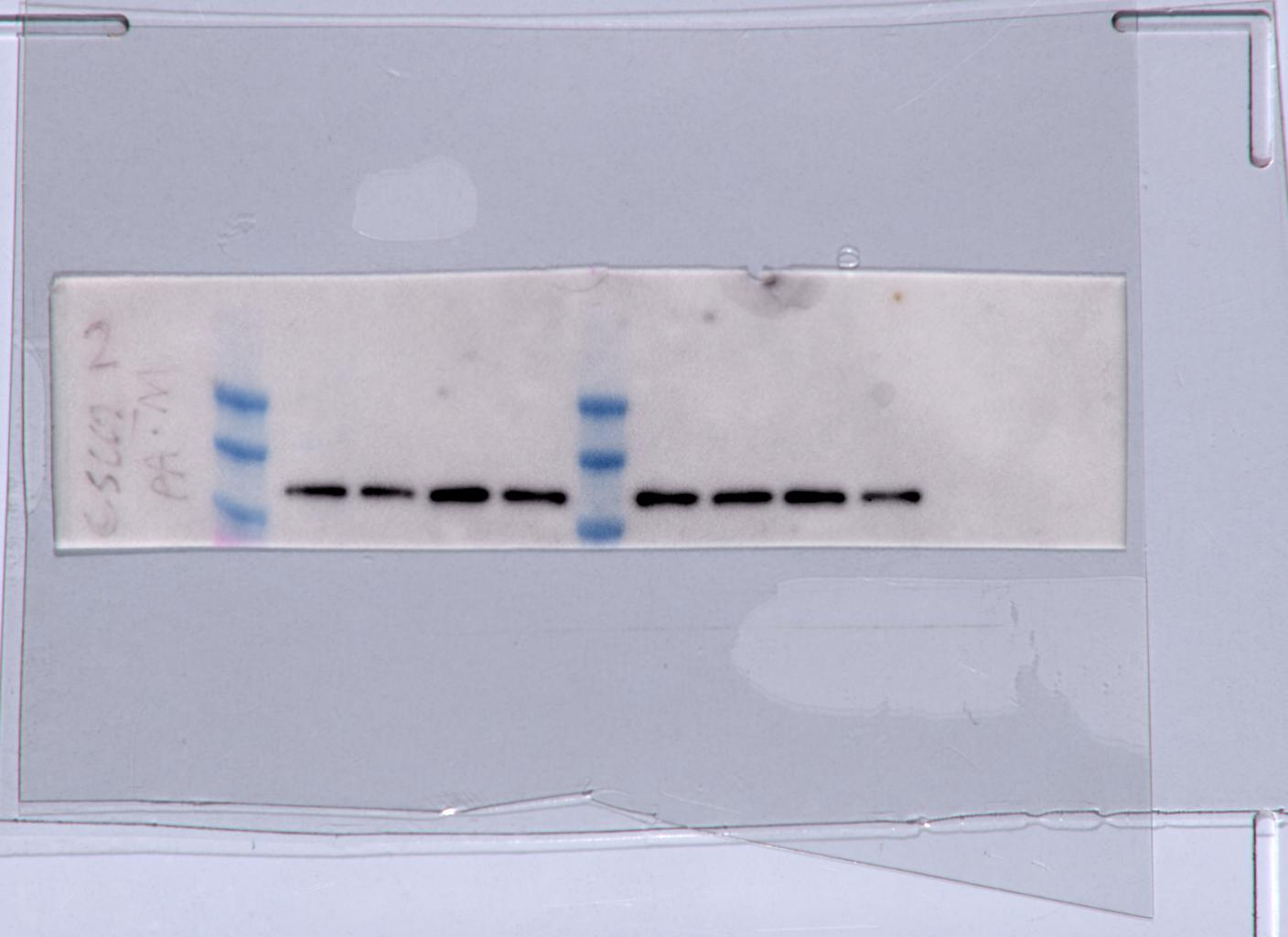

Supplement: Supplementary file 1 [file cancers-16-00370-s001.zip › JP pATM cs2 30-5 3mi 2022.05.30_14.11.04_Ch+Marker.jpg]

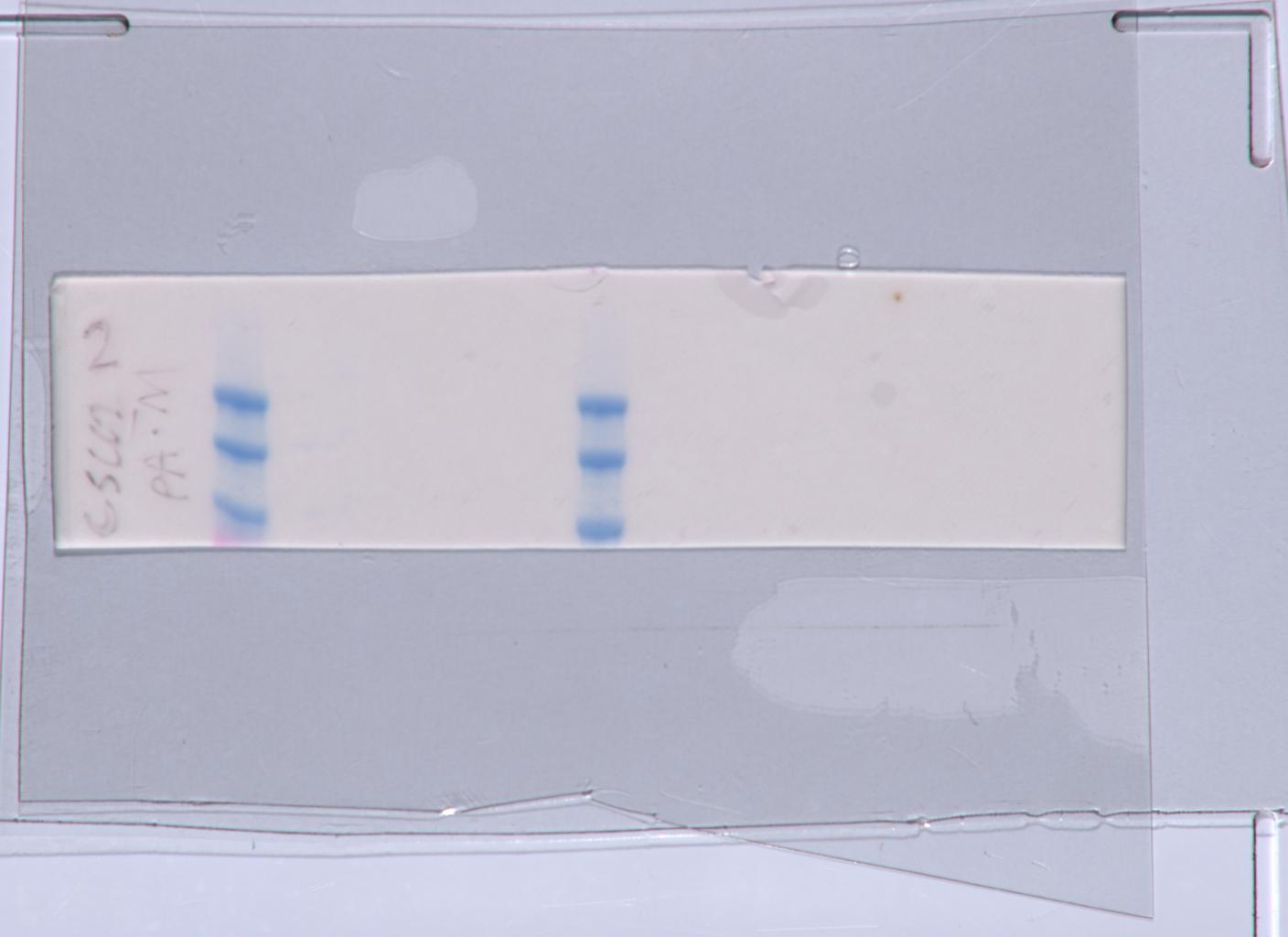

Supplement: Supplementary file 1 [file cancers-16-00370-s001.zip › JP pATM cs2 30-5 3mi 2022.05.30_14.11.04_Ch-Marker.jpg]

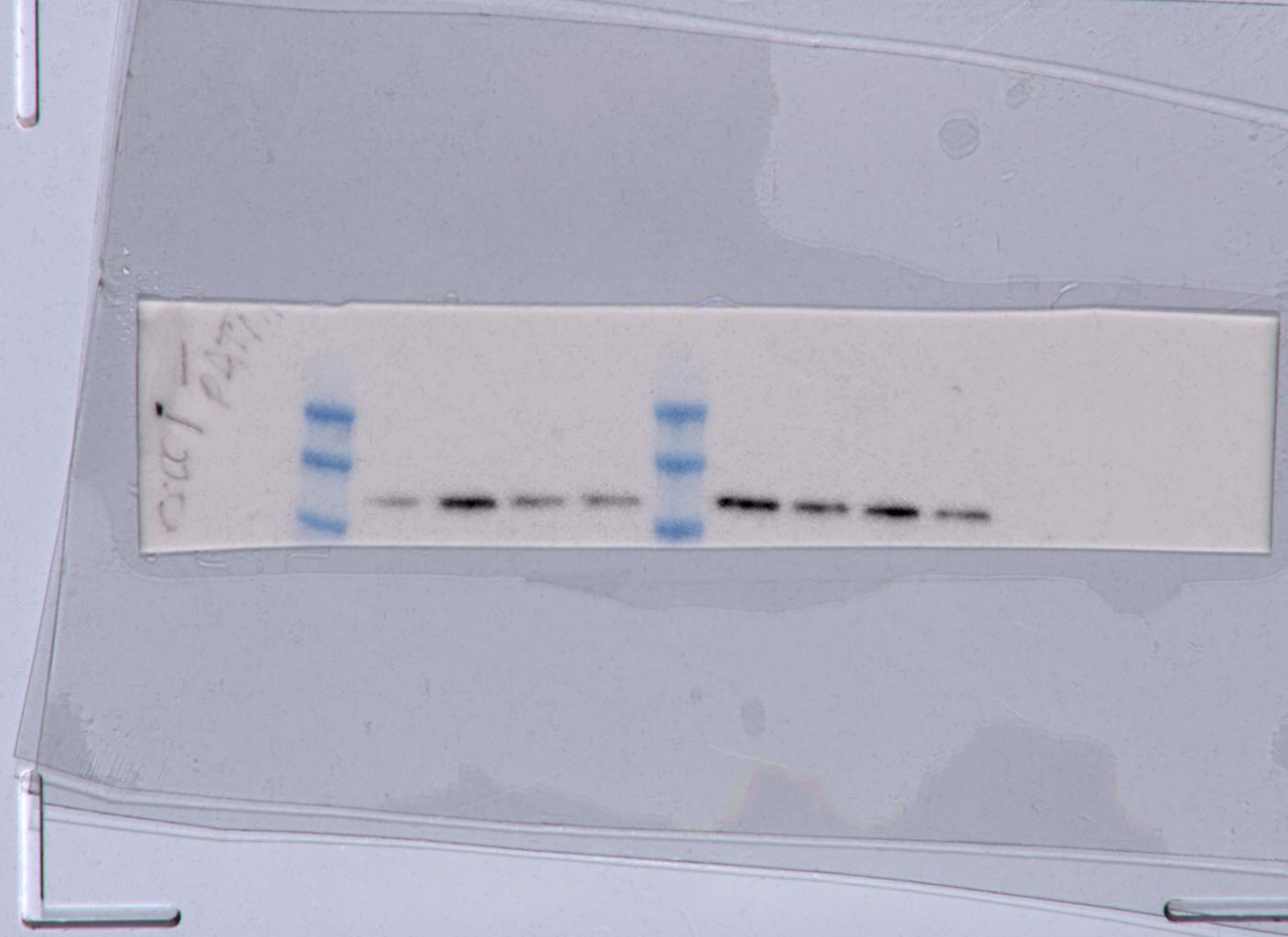

Supplement: Supplementary file 1 [file cancers-16-00370-s001.zip › JP pATM cscc1 30-5 2022.05.30_11.01.46_Ch+Marker.jpg]

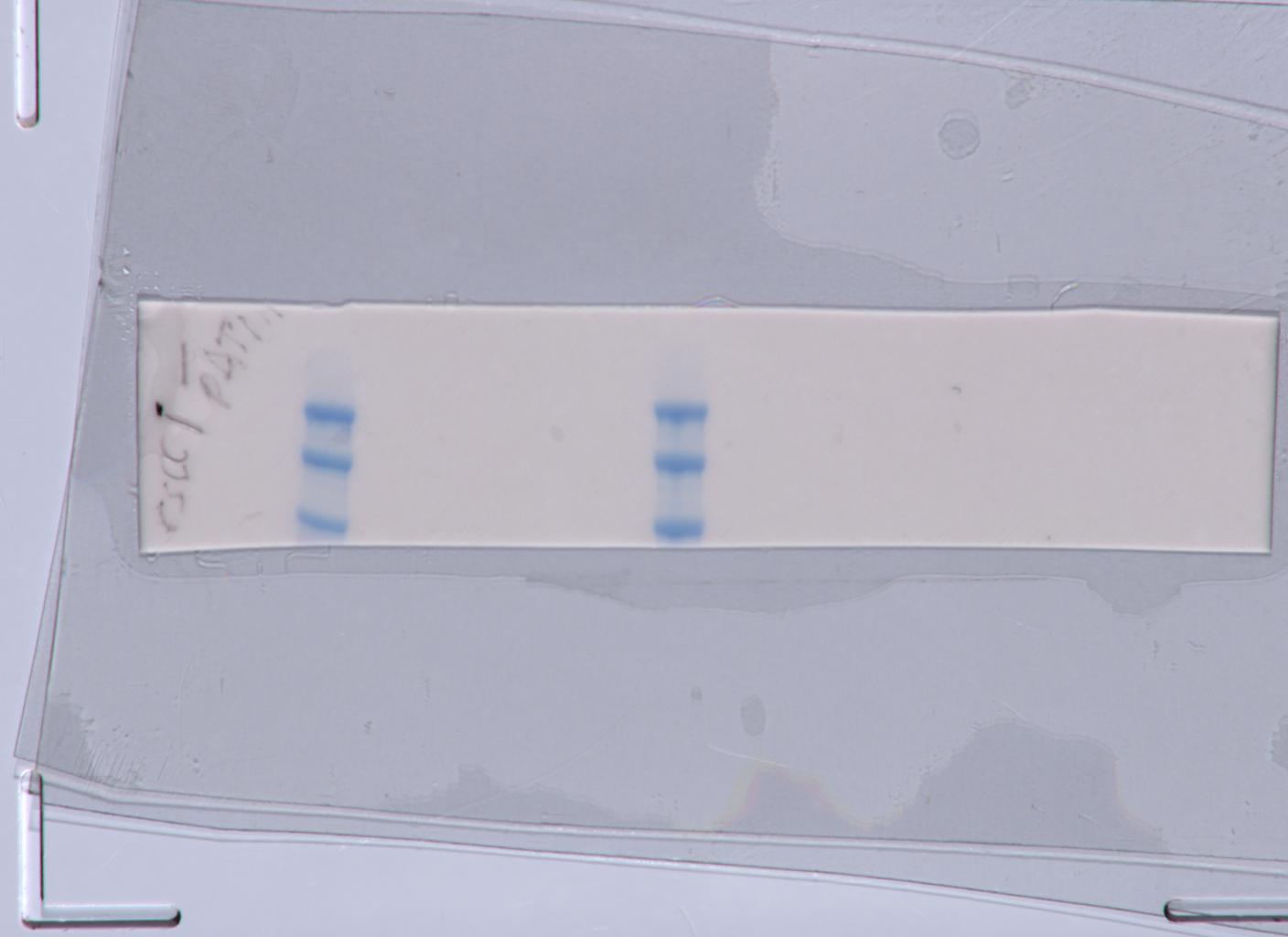

Supplement: Supplementary file 1 [file cancers-16-00370-s001.zip › JP pATM cscc1 30-5 2022.05.30_11.01.46_Ch-Marker.jpg]

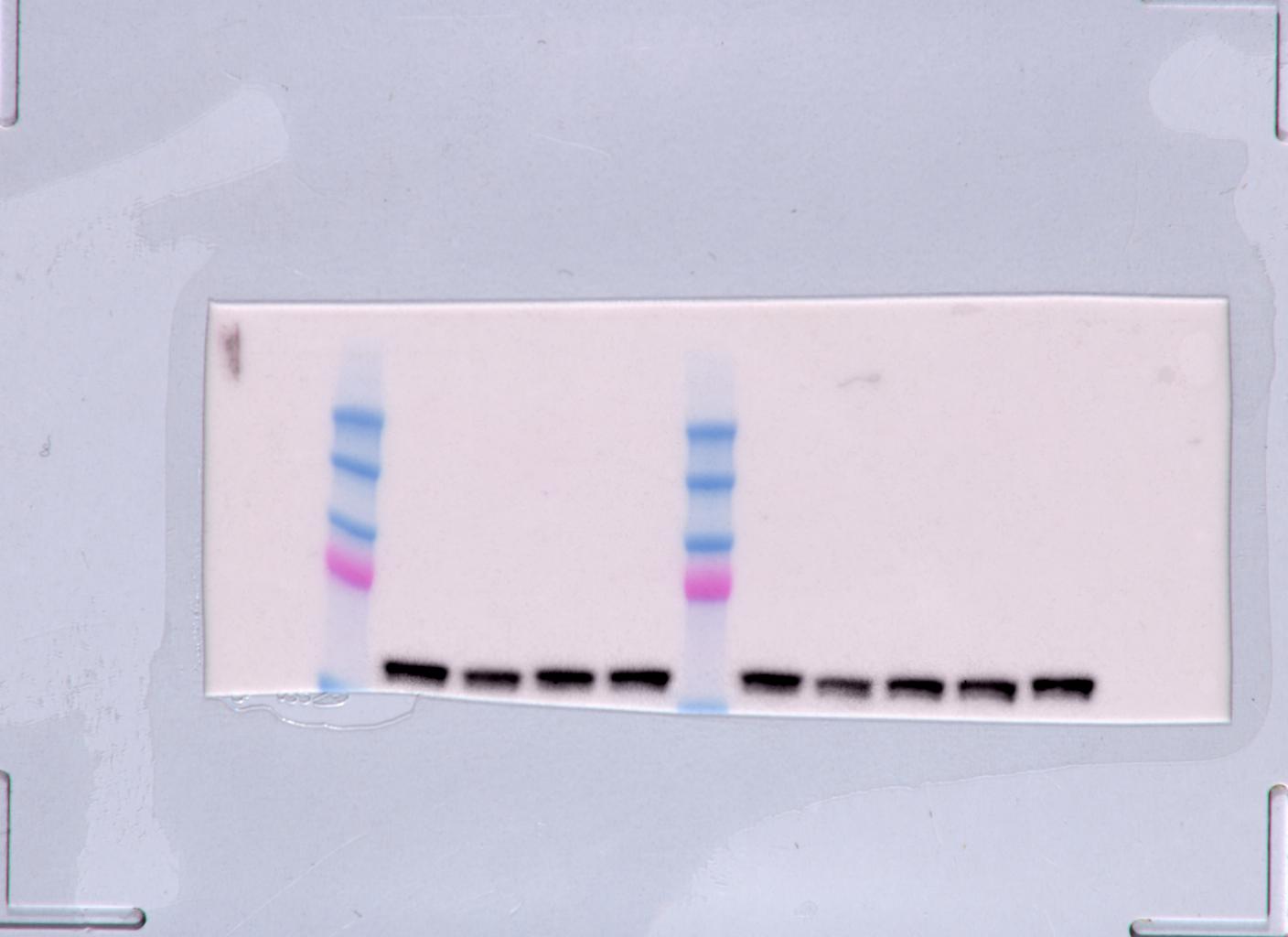

Supplement: Supplementary file 1 [file cancers-16-00370-s001.zip › JP PIKDIN gel1 1min 2022.03.25_15.14.47_Ch+Marker.jpg]

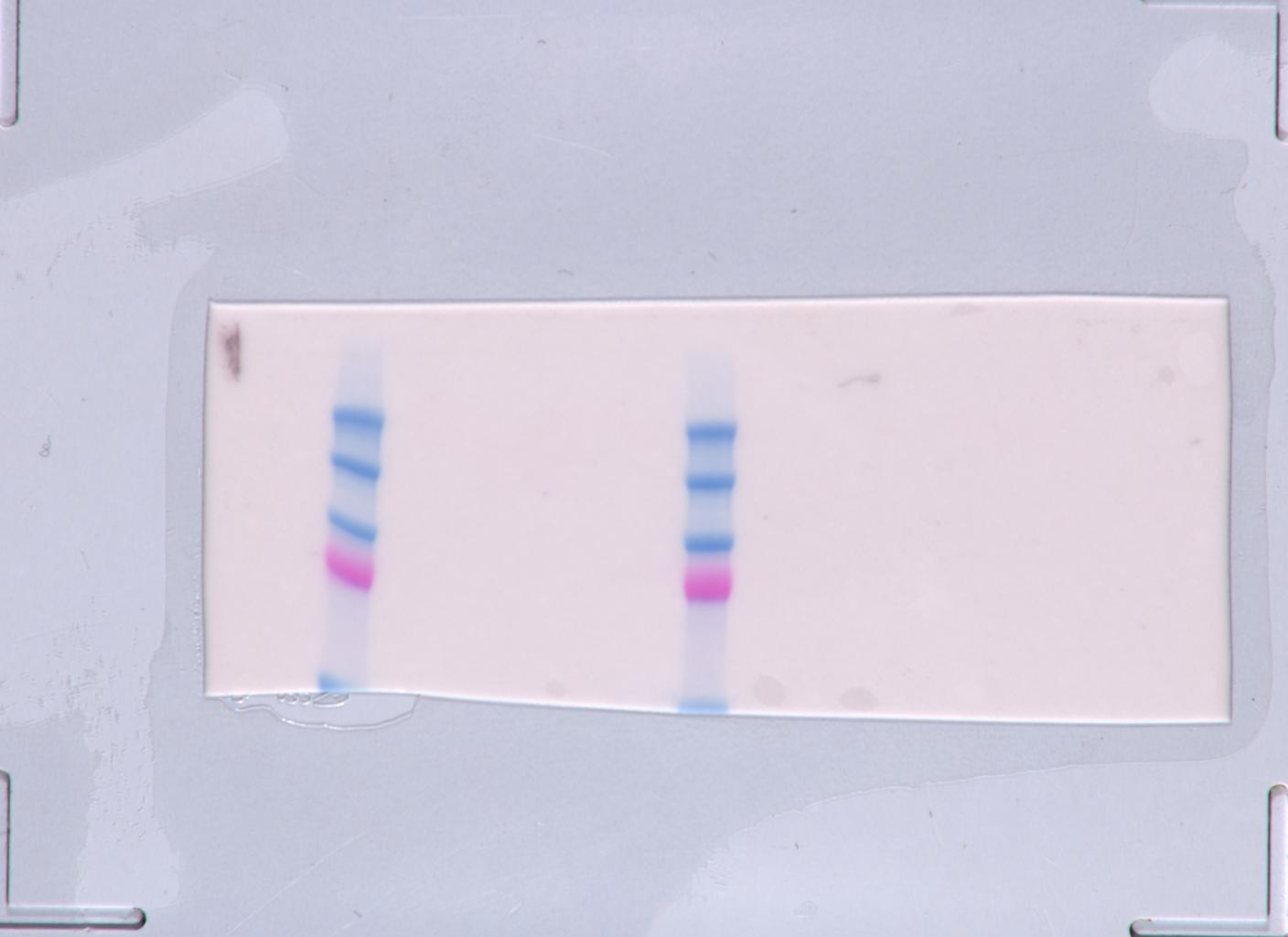

Supplement: Supplementary file 1 [file cancers-16-00370-s001.zip › JP PIKDIN gel1 1min 2022.03.25_15.14.47_Ch-Marker.jpg]

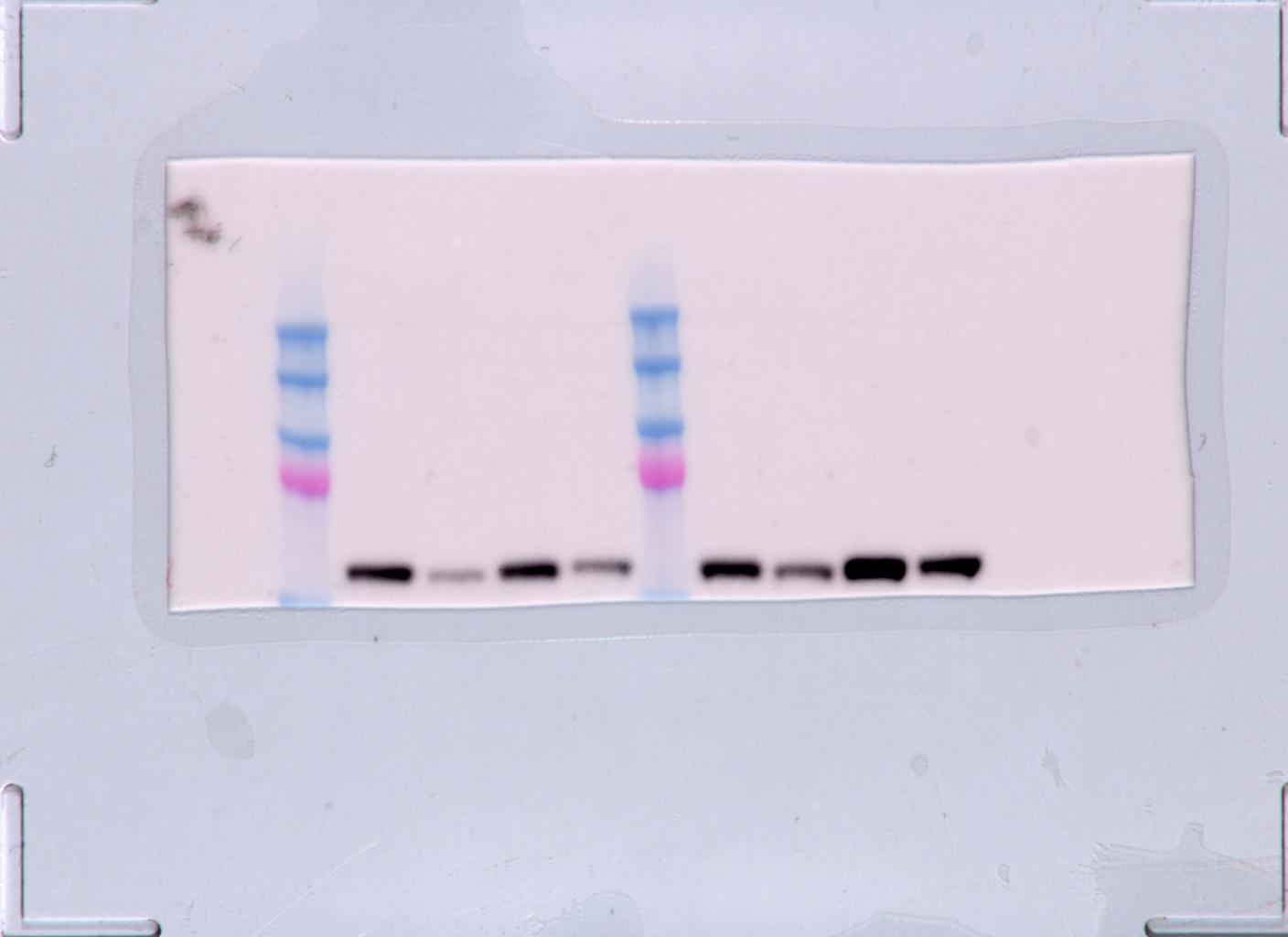

Supplement: Supplementary file 1 [file cancers-16-00370-s001.zip › JP PIKDIN gel2 2min 2022.03.25_15.19.17_Ch+Marker.jpg]

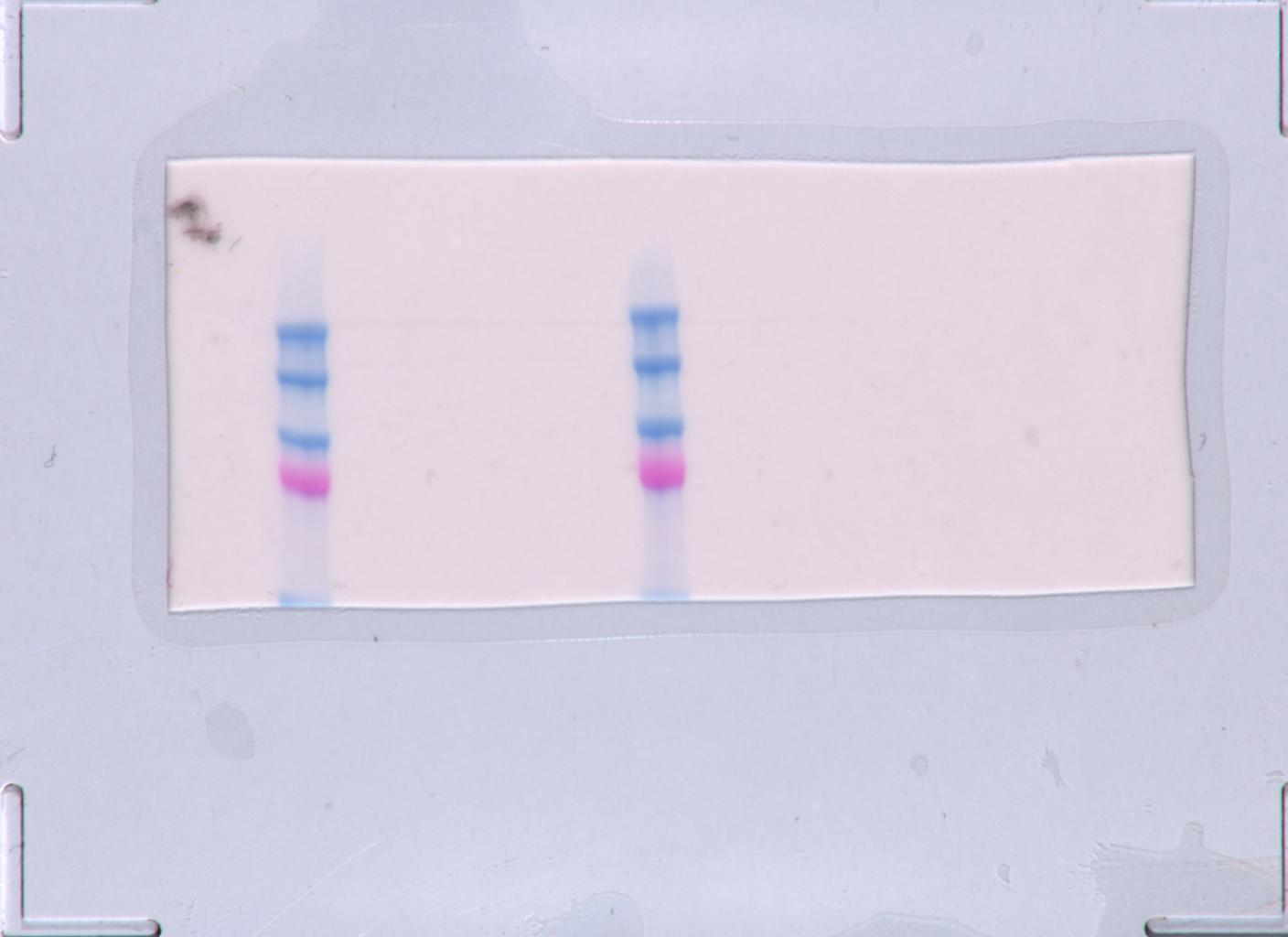

Supplement: Supplementary file 1 [file cancers-16-00370-s001.zip › JP PIKDIN gel2 2min 2022.03.25_15.19.17_Ch-Marker.jpg]

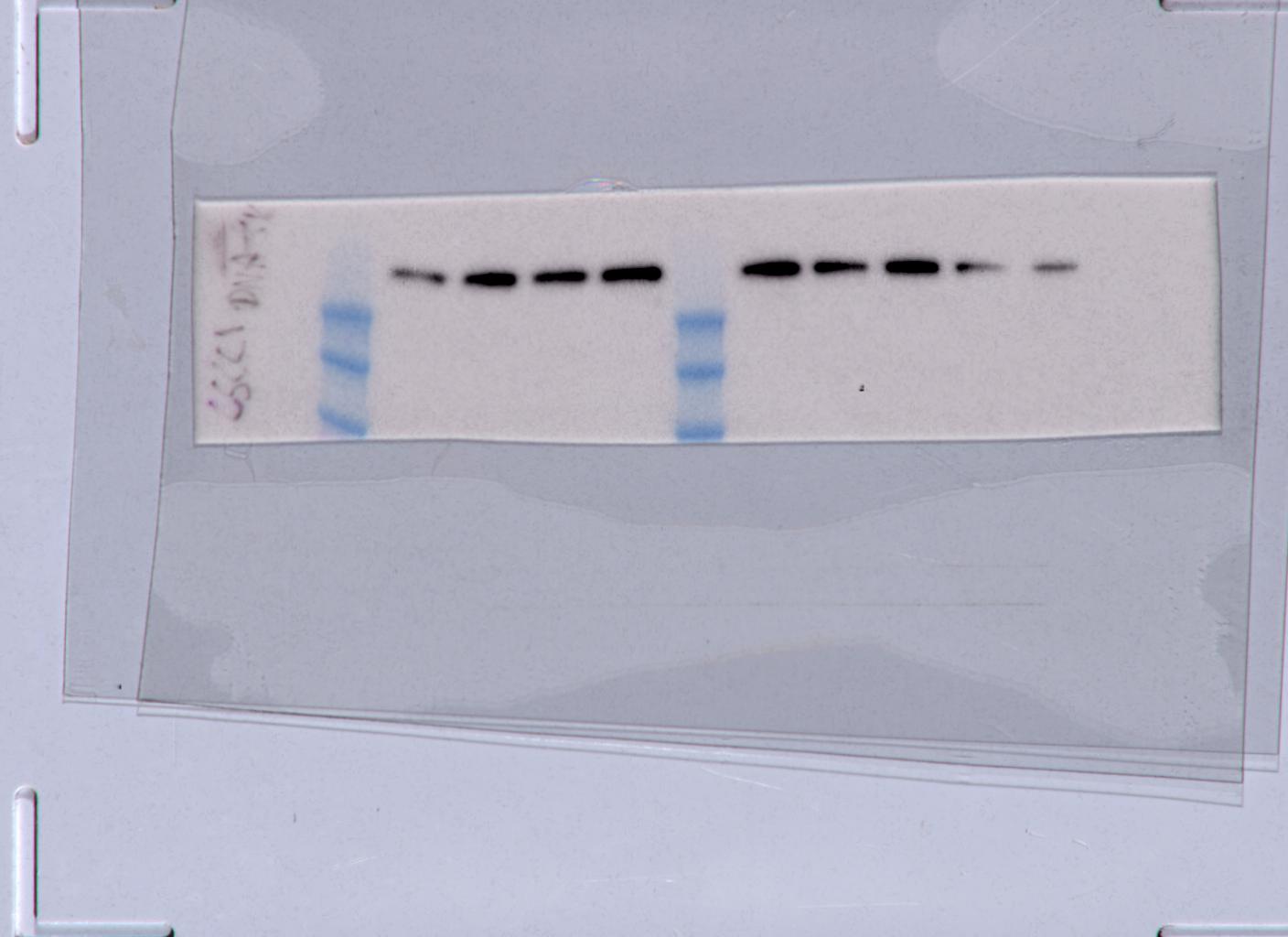

Supplement: Supplementary file 1 [file cancers-16-00370-s001.zip › JPDNAPK cs1 30-5 3m 2022.05.30_14.16.45_Ch+Marker.jpg]

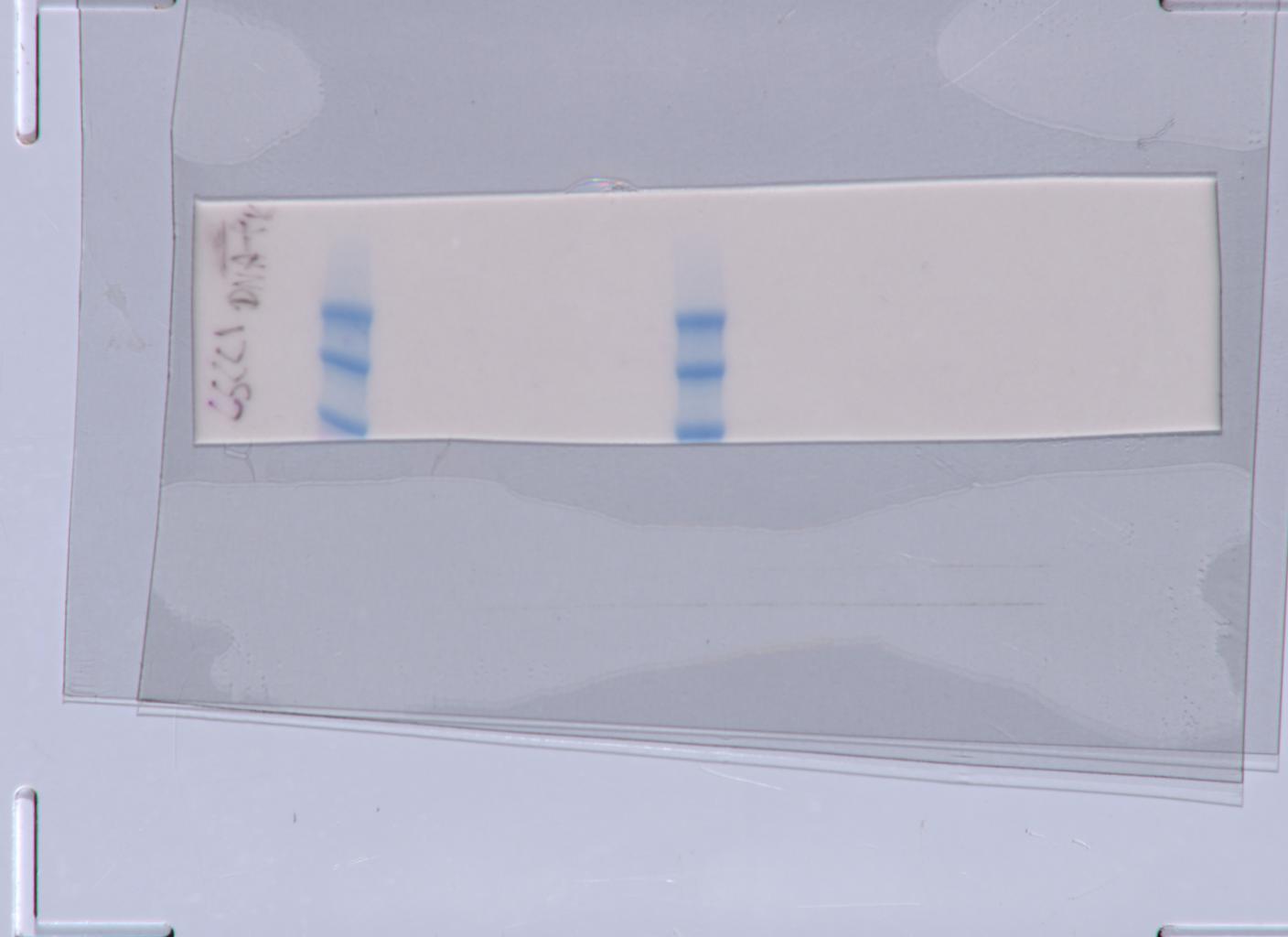

Supplement: Supplementary file 1 [file cancers-16-00370-s001.zip › JPDNAPK cs1 30-5 3m 2022.05.30_14.16.45_Ch-Marker.jpg]

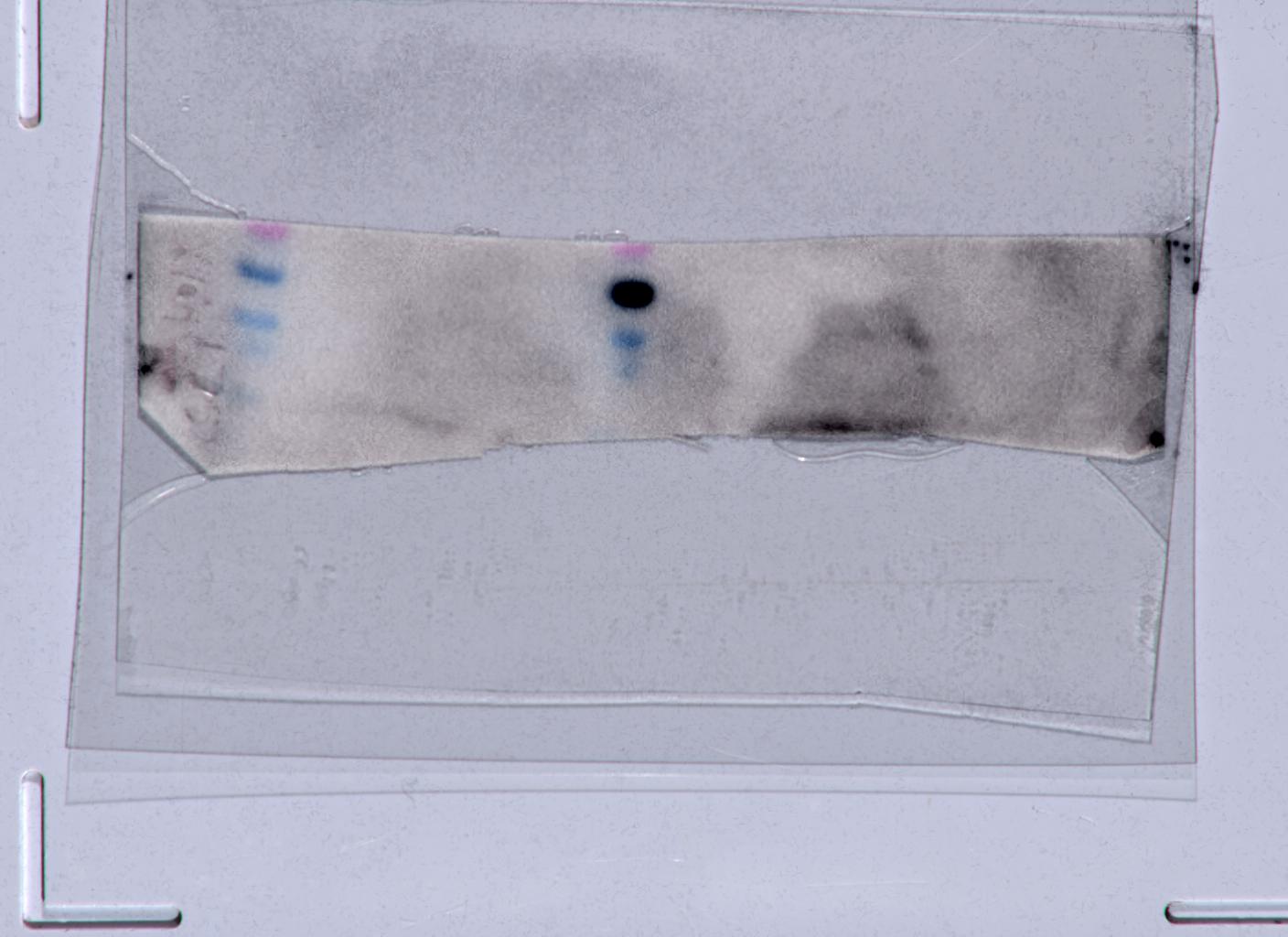

Supplement: Supplementary file 1 [file cancers-16-00370-s001.zip › JPh2ax cs1 30-5 2mfe 2022.05.30_14.47.48_Ch+Marker.jpg]

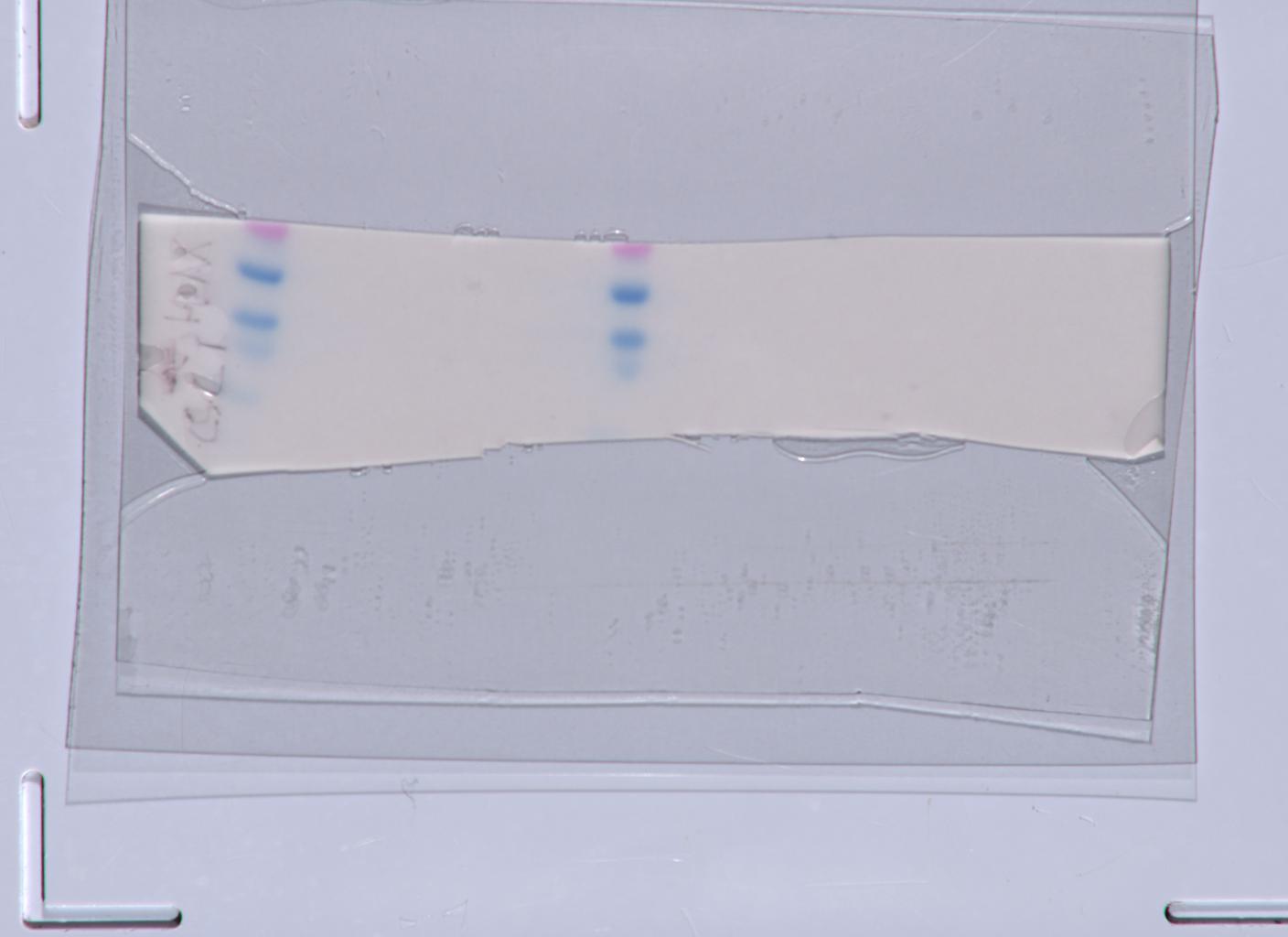

Supplement: Supplementary file 1 [file cancers-16-00370-s001.zip › JPh2ax cs1 30-5 2mfe 2022.05.30_14.47.48_Ch-Marker.jpg]

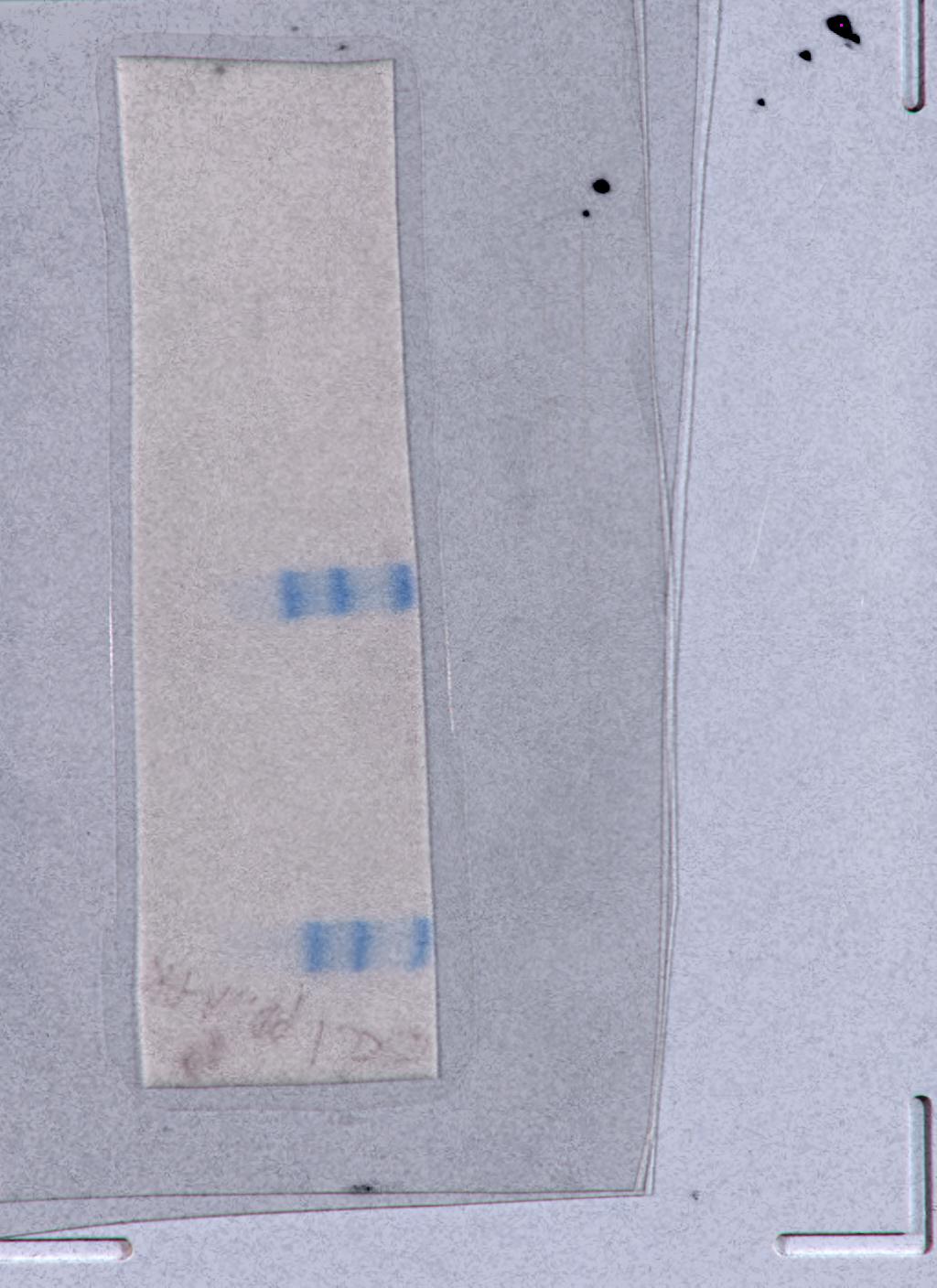

Supplement: Supplementary file 1 [file cancers-16-00370-s001.zip › JPpDNA cs1 30-5 10mf 2022.05.30_14.56.51_Ch+Marker.jpg]
